# Clustering systems of phylogenetic networks

**DOI:** 10.1007/s12064-023-00398-w

**Published:** 2023-08-12

**Authors:** Marc Hellmuth, David Schaller, Peter F. Stadler

**Affiliations:** 1 Department of Mathematics, Faculty of Science, Stockholm University, Albanovägen 28, 10691 Stockholm, Sweden; 2 Bioinformatics Group, Department of Computer Science and Interdisciplinary Center for Bioinformatics, Leipzig University, Härtelstraße 16-18, 04107 Leipzig, Germany; 3 Max Planck Institute for Mathematics in the Sciences, Inselstraße 22, 04103 Leipzig, Germany; 4 Department of Theoretical Chemistry, University of Vienna, Währingerstraße 17, 1090 Vienna, Austria; 5 Facultad de Ciencias, Universidad National de Colombia, Bogotá, Colombia; 6 Santa Fe Institute, 1399 Hyde Park Rd., Santa Fe, NM 87501 USA

**Keywords:** Compatibility, Level-*k*, Hybrid, Evolution, Cluster, Network phylogenetics, Least common ancestor

## Abstract

Rooted acyclic graphs appear naturally when the phylogenetic relationship of a set *X* of taxa involves not only speciations but also recombination, horizontal transfer, or hybridization that cannot be captured by trees. A variety of classes of such networks have been discussed in the literature, including phylogenetic, level-1, tree-child, tree-based, galled tree, regular, or normal networks as models of different types of evolutionary processes. Clusters arise in models of phylogeny as the sets ${{\,\mathrm{\texttt{C}}\,}}(v)$ of descendant taxa of a vertex *v*. The clustering system $\mathscr {C}_N$ comprising the clusters of a network *N* conveys key information on *N* itself. In the special case of rooted phylogenetic trees, *T* is uniquely determined by its clustering system $\mathscr {C}_T$. Although this is no longer true for networks in general, it is of interest to relate properties of *N* and $\mathscr {C}_N$. Here, we systematically investigate the relationships of several well-studied classes of networks and their clustering systems. The main results are correspondences of classes of networks and clustering systems of the following form: If *N* is a network of type $\mathbb {X}$, then $\mathscr {C}_N$ satisfies $\mathbb {Y}$, and conversely if $\mathscr {C}$ is a clustering system satisfying $\mathbb {Y},$ then there is network *N* of type $\mathbb {X}$ such that $\mathscr {C}\subseteq \mathscr {C}_N$.This, in turn, allows us to investigate the mutual dependencies between the distinct types of networks in much detail.

## Introduction

Networks used to model phylogenetic relationships typically are directed acyclic graphs (DAGs) with a single root, i.e., a unique vertex from which all other vertices can be reached from. As usual in phylogenetics, the subset *X* of vertices without descendants (the leaves of the network) represents the extant taxa, while the remaining vertices model their ancestors.

Phylogenetic trees and networks cannot be observed directly. Instead, they need to be inferred from measurable information such as dissimilarities or relational data encoding the relatedness of small subsets of taxa. Phylogenetic trees, for example, are determined by additive metric distances (Buneman 1974; Simões-Pereira 1969) (together with the knowledge of an outgroup to determine the root) as well as sets of rooted triples $ab\vert c$ recording that taxa *a* and *b* are more closely related with each other than with *c* (Aho et al. 1981). Similar results exist for certain types of networks, such as those determined by split-decomposable metrics and weakly compatible split systems (Bandelt and Dress 1989, 1992). Classes of phylogenetic networks are typically introduced by means of convenient graph-theoretical properties rather than their connection to readily available data. In most cases, it remains unknown whether the networks are uniquely determined by small building blocks. A notable exception are level-1 and level-2 networks, whose biconnected components have at most one or two minimal (hybrid) vertices, respectively, and so-called tree-child networks. These are encoded by their bi-nets and/or tri-nets, which can be seen as a generalization of rooted triples (Huber and Moulton 2013; van Iersel et al. 2017b; Van Iersel et al. 2022; Van Iersel and Moulton 2014; Semple and Toft 2021).

In this contribution, we are interested in particular in the relationships between the structure of networks *N* with leaf set *X* and their associated clustering systems $\mathscr {C}_N$, which contains, for each vertex *v* of *N*, the subset ${{\,\mathrm{\texttt{C}}\,}}(v)\subseteq X$ of leaves that can be reached from *v* (Nakhleh and Wang 2005; Huson and Rupp 2008). In the literature on phylogenetic networks, the sets $C\in \mathscr {C}_N$ are often called the “hardwired clusters” of *N*. Clustering systems $\mathscr {C}_N$ of a network are closely related to split systems (by associating $C\in \mathscr {C}_N$ with splits $C\vert (X\setminus C)\cup \{*\}$, where *X* is augmented by an additional outgroup $*$) (Dress 1997); on the other hand, clustering serves as a standard approach to analyze and interpret (dis)similarity data. Thus, it is of theoretical and practical interest to understand to what extent clustering systems determine the networks from which they derive.

As a special case, there is a well-known 1-to-1 correspondence between rooted phylogenetic trees *T* and hierarchies $\mathscr {C}$ (Semple and Steel 2003), i.e., clustering systems that do not contain pairs of overlapping clusters. Therefore, *T* is uniquely determined by $\mathscr {C}_T=\mathscr {C}$. This simple correspondence, however, is no longer true for (phylogenetic) networks. Nevertheless, it is not difficult to find some network *N* for a given clustering system $\mathscr {C}$ such that $\mathscr {C}_N= \mathscr {C}$: It suffices to determine the Hasse diagram $N = {\mathfrak {H}}[\mathscr {C}]$ of the inclusion partial order of the clustering system $\mathscr {C}$ to obtain such a network. For a phylogenetic tree *T*, the Hasse diagram ${\mathfrak {H}}[\mathscr {C}_T]$ and *T* are isomorphic. For general networks, however, the situation is much more complicated (Nakhleh and Wang 2005; Huson and Rupp 2008; Zhang 2019).

Phylogenetic networks can be seen as a superposition of multiple rooted trees that correspond to alternative explanations of the phylogenetic relationships of the leaves (Huson and Scornavacca 2011). This suggests to consider the union of the clusters of all trees contained in a network *N*, usually referred to as the softwired clusters of *N*. While the set $\mathscr {C}_N$ of all hardwired clusters of *N* is of linear size (i.e., in $O(\vert V(N)\vert )$, there may be exponentially many softwired clusters of *N*. In general, phylogenetic networks *N* interpreted in the softwired sense are computationally hard to work with and even just checking whether *N* contains a softwired cluster is NP-hard (Kanj et al. 2008; Huson and Scornavacca 2011). The construction of minimal networks from softwired clusters is fixed parameter tractable in the level *k* of the network (Kelk and Scornavacca 2014). From a practical point of view, however, it seems at least very difficult to estimate softwired clusters directly from data such as sequence similarities. Therefore, we consider exclusively the set of hardwired clusters $\mathscr {C}_N$ in this contribution.

A broad array of different types of networks have been studied in the literature in order to model different modes of non-tree-like evolution such as horizontal gene transfer, recombination, or hybridization, see Kong et al. (2022) for a current review. Naturally, the question arises how much information about the structure of *N* is contained in the clustering system $\mathscr {C}_N$. We will in particular be concerned with the following, inter-related questions

1. Which types of networks *N* satisfy $N\simeq {\mathfrak {H}}[\mathscr {C}_N]$?
2. What are necessary properties of the clustering systems $\mathscr {C}_N$ obtained for networks *N* of a given class?
3. Which types of networks *N*, if any, can be characterized in terms of properties of their clustering systems $\mathscr {C}_N$?
4. When is a network *N* uniquely determined by $\mathscr {C}_N$ or by the corresponding multiset of clusters $\mathscr {M}_N$?
5. When is a clustering system $\mathscr {C}$ compatible with a specified type of network *N* in the sense that there is network *N* of given type such that $\mathscr {C} \subseteq \mathscr {C}_N$?

While addressing these questions, we will also consider the implications between the defining properties of the various network classes. To help the reader navigate this contribution, we summarize the properties of interest in Table 1 and point to their formal definitions. Complementarily, properties of clustering systems are compiled in Table 2. Many of the results established here are summarized in Table 3 and Fig. 19 in  section “Summary”.

It is important to note that the literature on phylogenetic networks does not always utilize the same nomenclature. In particular, properties such as *binary*, *separated*, *conventional*, or *phylogenetic* are—more often than not—taken for granted in a given publication and explicitly or tacitly included in the definition of “phylogenetic network.” Here, we start from a very general setting of rooted DAGs, called “networks” throughout. All additional properties are made explicit throughout. We furthermore strive to prove all statements as general as possible. The reader will therefore on occasion find results that are well known in the field, although earlier proofs pertain to a more restrictive setting.

This paper is organized as follows. In section “Preliminaries”, we provide the basic terminology and definitions used throughout this paper. In section “Networks and clustering systems”, we start with a closer look at phylogenetic networks (“Basic Concepts” section) and related concepts which includes graph modifications such as arc-contractions or expansions (“Arc-expansion and arc-contraction” section) as well as the structural properties of non-trivial biconnected components (called blocks) in networks (“Blocks” section). We then continue in section “Clusters, Hasse diagrams, and regular networks” to characterize the structure of the Hasse diagram of clustering systems. In particular, we provide new characterizations of regular networks, that is, networks that are isomorphic to the Hasse diagram of some clustering system.

In section“Semi-regular networks”, we consider semi-regular networks, i.e., networks that do not contain so-called shortcuts and satisfy the path-cluster-comparability (PCC) property as introduced in section “Path-cluster comparability”. (PCC) simply ensures that one of the clusters ${{\,\mathrm{\texttt{C}}\,}}(u)$ and ${{\,\mathrm{\texttt{C}}\,}}(v)$ is a subset of the other one whenever the vertices *u* and *v* are connected by a directed path in *N*. Although this property does not seem to have been studied so far, it turns out to play a fundamental role in the relationships between networks and their clustering systems. Regular networks, as it turns out, are precisely the semi-regular networks that do not contain vertices with outdegree 1. In addition, we show how to obtain regular networks $N'$ from networks *N* that only satisfy (PCC) such that $\mathscr {C}_N = \mathscr {C}_{N'}$. In section “Separated networks and cluster networks”normal, and tree-based networks, we consider separated networks (networks for which each vertex with indegree greater than 1 has outdegree 1) and cluster networks in the sense of Huson and Rupp (2008) (whose definition is somewhat more involved). As we shall see, cluster networks are precisely the networks that are semi-regular, separated, and phylogenetic. We then show in section “Cluster multisets of semi-regular networks” that semi-regular networks are uniquely determined by their multisets of clusters and that, in turn, cluster networks, as a subclass of regular networks, are uniquely determined by their clustering systems. Section “Tree-child, normal, and tree-based networks” makes a short excursion to so-called tree-child, normal, and tree-based networks and their mutual relationships.

In “Least common ancestors and LCA-networks” section, we then have a closer look at the concept of least common ancestors (${{\,\textrm{lca}\,}}$) in networks. In contrast to rooted trees, the ${{\,\textrm{lca}\,}}$ of a pair of leaves (or more generally, a subset of leaves) is in general not uniquely defined. We introduce in “Basics” section several classes of networks in which the ${{\,\textrm{lca}\,}}$ is unique for at least certain subsets of leaves. This leads to the cluster-lca property (CL) which is satisfied by a network *N* whenever ${{\,\textrm{lca}\,}}({{\,\mathrm{\texttt{C}}\,}}(v))$ is uniquely determined for all $v\in V(N)$. We shall see that every network that satisfies (PCC), and this in particular includes all normal networks, also satisfy (CL). In “LCA-networks” section, we consider lca-networks, i.e., networks in which ${{\,\textrm{lca}\,}}(A)$ is uniquely determined for all leaf sets $A\subseteq X$. Among other results, we show that a clustering system $\mathscr {C}$ is closed (under intersection) if only if it is the clustering systems $\mathscr {C}_N$ of an lca-network. We then consider in “Strong LCA-networks and weak hierarchies” section the subclass of strong lca-networks, in which, for all $A\subseteq X$, it holds that ${{\,\textrm{lca}\,}}(A)={{\,\textrm{lca}\,}}(\{x,y\})$ for a suitably chosen pair of leaves *x* and *y*. These are closely related to weak hierarchies.

A very prominent role in phylogenetics is played by level-1 networks. Section “Level-1 networks” is devoted to establishing structural results for level-1 networks and their underlying clustering systems. After establishing basic results, we derive in section “Property (L)” a simple condition, called property (L), for clustering systems that is defined in terms of the intersection of its elements. As a main result of this contribution, we obtain in section “Characterization of clustering systems of level-1 networks” a simple characterization of the clustering systems of (phylogenetic, separated) level-1 network as the ones that are closed and satisfy (L). We then show in section “Compatibility of clustering systems and intersection closure” that property (L) is sufficient to ensure that clustering systems are “compatible” with a (phylogenetic, separated) level-1 network. Moreover, we provide a polynomial time algorithm to check if $\mathscr {C}$ is compatible with some level-1 network and, in the affirmative case, to construct such a network. We finally consider in section “Special subclasses of level-1 networks” several subclasses of level-1 networks as, e.g., galled trees or binary, conventional, separated, or quasi-binary level-1 networks, and characterize their clustering systems. Finally, we show that quasi-binary level-1 networks are encoded by their multisets of clusters. In section “Summary”, we provide a summary of the main results (see in particular Table 3 and Fig. 19).

## Preliminaries

The power set of a given set *X* is denoted by $2^X$. Two sets *A* and *B* overlap if $A\cap B\notin \{\emptyset , A,B\}$.

We consider graphs $G=(V,E)$ with finite vertex set $V(G){:}{=}V$ and arc set $E(G){:}{=}E$. A graph *G* is *undirected* if *E* is a subset of the set of two-element subsets of *V* and *G* is *directed* if $E\subseteq (V\times V){\setminus } \{(v,v)\mid v\in V \}$. Thus, arcs $e\in E$ in an undirected graph *G* are of the form $e=\{x,y\}$ and in directed graphs of the form $e=(x,y)$ with $x,y\in V$ being distinct. The *degree* of a vertex $v\in V$ in an undirected or directed graph *G*, denoted by $\deg _G(v)$, is the number of arcs that are incident with *v*. If *G* is directed, we furthermore distinguish the indegree ${{\,\textrm{indeg}\,}}_{G}(v) = \vert \left\{ u \mid (u,v)\in E\right\} \vert$ and the outdegree ${{\,\textrm{outdeg}\,}}_{G}(v) = \vert \{u \mid (v,u)\in E\}\vert$. A graph *H* is a *subgraph* of *G*, in symbols $H \subseteq G$, if $V(H)\subseteq V(G)$ and $E(H)\subseteq E(G)$. A subgraph $H\subseteq G$ is *induced* by some subset $W \subseteq V(G)$ if $V(H)=W$ and, additionally, $\{x,y\}\in E(G)$ (resp., $(x,y)\in E(G)$) and $x,y\in W$ implies that $\{x,y\}\in E(H)$ (resp., $(x,y)\in E(H)$). In the latter case, we write $H=G[W]$. Moreover, $G-v$ denotes the induced subgraph $G[V\setminus \{v\}]$.

A path *P* in an undirected (resp. directed) graph *G* is a subgraph consisting of $k\ge 1$ distinct vertices $\{v_1,\dots ,v_k\}$ and arcs $\{v_i,v_{i+1}\}\in E$ (resp. $(v_i,v_{i+1})\in E$) for all $1\le i\le k-1$. We call such paths also $v_1v_k$-paths. In case *G* is undirected, $v_1v_k$-paths are also $v_kv_1$-paths. However, if *G* is directed, the existence of a $v_1v_k$-paths does not imply that there is a $v_kv_1$-paths. We will often write *undirected path* for a subgraph *P* of a directed graph *G* that has vertices $\{v_1,v_2,\dots ,v_k\}$, $k\ge 1$, and the *forward arc* $(v_i,v_{i+1})$ or the corresponding *backward arc* $(v_{i+1},v_i)$ for all $1\le i\le k-1$. The vertices $v_1$ and $v_k$ in a directed or undirected path *P* are the *endpoints* of *P* and all other vertices (in *P*) are its *inner vertices*. A path *P* with vertices $\{v_1,v_2,\dots ,v_k\}$ in a directed graph G is *induced (in G)* if $(v_i,v_j) \in E(G)$ precisely if $j = i+1$, for all $i\in \{1,\dots ,k-1\}$.

A directed cycle *K* in a directed graph *G* is a subgraph with vertices $\{v_1,v_2,\dots ,v_k\}$, $k\ge 2$, and arcs $(v_i,v_{i+1})\in E$ for all $1\le i\le k-1$ and additionally $(v_k, v_1)\in E$. In analogy to undirected paths, an *undirected cycle* *K* in a directed graph *G* is a subgraph with $k\ge 3$ vertices that can be ordered in the form $\{v_1,v_2,\dots ,v_k\}$ such that the forward arc $(v_i,v_{i+1})$ or the corresponding backward arc $(v_{i+1},v_i)$ for $1\le i\le k-1$ as well as the forward arc $(v_k,v_1)$ or the backward arc $(v_1,v_k)$ are exactly the arcs of *K*.

An undirected graph $G=(V,E)$ is *bipartite* if there is a partition of *V* into subsets *W* and $W'$ such that every arc in *G* connects one vertex in *W* to one vertex in $W'$. If in addition, $x\in W$ and $x'\in W'$ imply $\{x,x'\}\in E$, then *G* is *complete bipartite*.

### Graph connectivity

An undirected graph is *connected* if, for every two vertices $u,v\in V$, there is a path connecting *u* and *v*. A directed graph is *connected* if its underlying undirected graph is connected. A connected component of *G* is a maximal induced subgraph that is connected. A vertex *v* is a cut vertex in a graph *G* if $G[V(G)\setminus \{v\}]$ consists of more connected components than *G*. Similarly, a directed or undirected arc (*u*, *v*) is a cut arc in *G* if the graph $G'$ with vertex set $V(G')=V(G)$ and arc set $E(G')=E(G)\setminus \{(u,v)\}$ consists of more connected components than *G*.

An undirected or directed graph is *biconnected* if it contains no vertex whose removal disconnects the graph. A *block* of an undirected or a directed graph is a maximal biconnected subgraph (Gambette et al. 2012). A block *B* is called *non-trivial* if it contains an (underlying undirected) cycle. Equivalently, a block is non-trivial if it is not a single vertex or a single arc. An arc that is at the same time a (trivial) block is a cut arc. For later reference, we state here the following observations that are immediate consequences of the fact that two distinct blocks in a graph share at most one vertex (West 2001, Proposition 4.1.19):

#### Observation 1

If two biconnected subgraphs share two vertices, then their union is contained in a common block.

#### Observation 2

If *B* and $B'$ are distinct blocks of a directed graph, then *B* and $B'$ are arc-disjoint.

The latter is justified by the fact that if blocks *B* and $B'$ share a common arc, then $B\cup B'$ is biconnected and thus $B=B'$ since blocks are always *maximal* biconnected subgraphs. We will frequently make use of

#### Theorem 1

(West 2001, Theorem 4.2.4) For a graph *G* with at least three vertices, the following statements are equivalent:

1. *G* is biconnected.
2. For all $x,y\in V(G)$, there are at least two internally vertex-disjoint (undirected) paths connecting *x* and *y*.
3. For all $x,y\in V(G)$, there is an (undirected) cycle containing *x* and *y*.

#### Corollary 1

Any two vertices of a non-trivial block *B* lie on a common (undirected) cycle in *B*.

The following well-known result will also be useful throughout:

#### Proposition 1

(Diestel 2017, Proposition 3.1.1) Let *H* be a biconnected subgraph of *G* and *P* be a path in *G* that only shares its endpoints with *H*. Then, the subgraph obtained by adding *P* to *H* is again biconnected.

### Directed acyclic graphs

A directed graph $G=(V,E)$ is *acyclic* if it does not contain a directed cycle. In particular, every undirected cycle in a directed acyclic graph (DAG) contains at least one forward and one backward arc. In a DAG *G*, a vertex $u\in V$ is called an *ancestor* of $v\in V$ and *v* a *descendant* of *u*, in symbols $v \preceq _G u$, if there is a directed path (possibly reduced to a single vertex) in *G* from *u* to *v*. We write $v \prec _G u$ if $v \preceq _G u$ and $u\ne v$. If $u \preceq _G v$ or $v \preceq _G u$, then *u* and *v* are $\preceq _G$-*comparable* and otherwise, $\preceq _G$-*incomparable*. Moreover, if $(u,v)\in E$, we say that *u* is a *parent* of *v*, $u\in {{\,\textrm{par}\,}}_{G}(v)$, and *v* is a *child* of *u*, $v\in {{\,\textrm{child}\,}}_{G}(u)$. Following Huber et al. (2019a), we call a vertex *v* that is $\preceq _{G}$-minimal in a block *B* a *terminal vertex* (of *B*). Note that every terminal vertex *v* of a non-trivial block *B* must always have indegree at least 2 since, by Corollary 1, *v* lies on some undirected cycle in *B* and, by $\preceq _{G}$-minimality of *v* in *B*, its two incident vertices on this cycle must both be in-neighbors. Below we will consider DAGs in which terminal vertices are a type of so-called hybrid vertices.

An arc (*u*, *w*) in a DAG *G* is a *shortcut* if there is a vertex $v\in {{\,\textrm{child}\,}}(u)\setminus \{w\}$ such that $w\prec _G v$ (or, equivalently, if there is a vertex $v'\in V(G)$ such that $w\prec _G v'\prec _G u$). In other words, an arc (*u*, *w*) of *N* is a shortcut if *G* has a directed path from *u* to *w* avoiding (*u*, *w*) (Linz and Semple 2020; Döcker et al. 2019). A DAG without shortcuts is *shortcut-free*.

#### Observation 3

Let *G* be a DAG. The following statements are equivalent:

1. *G* is shortcut-free.
2. For all $u\in V(G)$, $v,w\in {{\,\textrm{child}\,}}_G(u)$ are $\preceq _G$-comparable if and only if $v=w$.
3. For all $u\in V(G)$, $v,w\in {{\,\textrm{par}\,}}_G(u)$ are $\preceq _G$-comparable if and only if $v=w$.

## Networks and clustering systems

### Basic concepts

We define (phylogenetic) networks here as a slightly more general class of DAGs than what is customarily considered in most of the literature on the topic.

#### Definition 1

A *(rooted) network* is a directed acyclic graph $N=(V,E)$ such that

(N1): There is a unique vertex $\rho _N$, called the *root* of *N*, with ${{\,\textrm{indeg}\,}}(\rho _N)=0$.

A network is *phylogenetic* if

(N2): There is no vertex $v\in V$ with ${{\,\textrm{outdeg}\,}}(v)=1$ and ${{\,\textrm{indeg}\,}}(v)\le 1$.

A vertex with $v\in V$ is a *leaf* if ${{\,\textrm{outdeg}\,}}(v)=0$, a *hybrid vertex* if ${{\,\textrm{indeg}\,}}(v)>1$, and *tree vertex* if ${{\,\textrm{indeg}\,}}(v)\le 1$. The set of leaves is denoted by *X*.

*We emphasize that all networks considered here are rooted and thus we always use the term “network” instead of “rooted network.”*

We note that a leaf $x\in X$ is always either a hybrid vertex or a tree vertex. Networks without hybrid vertices are *trees*. The set of *inner vertices* of a network *N* is $V^0{:}{=}V^0(N){:}{=}V(N){\setminus } X$. A leaf $x\in X$ is a *strict descendant* of $v\in V$ if every directed path from the root $\rho _N$ to *x* contains *v*. In contrast to the even more general definition (Definition 3 Huson and Scornavacca 2011), we use the term “phylogenetic” here to mean that vertices with indegree 1 and outdegree 1 do not appear. Moreover, the root is either the single leaf or has ${{\,\textrm{outdeg}\,}}(\rho _N)\ge 2$. Rooted phylogenetic networks thus generalize rooted phylogenetic trees. Since the root is an ancestor of all vertices, *N* is connected.

For a vertex *v* of *N*, the *subnetwork* *N*(*v*) *of* *N* *rooted at* *v*, is the network obtained from the subgraph *N*[*W*] induced by the vertices in $W{:}{=}\{w\in V(N)\mid w\preceq _N v\}$ and by suppression of *w* if it has indegree 0 and outdegree 1 in *N*[*W*] or hybrid vertices of *N* that have in- and outdegree 1 in *N*[*W*].

**Table 1:** Summary of networks considered in this paper

Network *N* is/satisfies		References
Tree (level-0)	*N* does not contain hybrid vertices	
Shortcut-free	*N* does not contain shortcuts	
Phylogenetic	cf. Definition 1 (N2)	Definition 1
Separated	All hybrid vertices of *N* have outdegree 1	Definition 2
Binary	Every tree vertex *v* is either a leaf or has ${{\,\textrm{outdeg}\,}}(v)=2$, and every hybrid vertex *v* satisfies ${{\,\textrm{indeg}\,}}(v)=2$ and ${{\,\textrm{outdeg}\,}}(v)=1$	Definition 3
Level-*k*	Each block *B* of *N* contains at most *k* hybrid vertices (distinct from the “root” of *B*)	Definition 9
Regular	There is a prescribed isomorphism between *N* and the Hasse diagram ${\mathfrak {H}}[\mathscr {C}_N]$ of the clusters in *N*	Definition 11
Path-cluster-comparability (PCC)	For all $u,v\in V(N)$, *u* and *v* are $\preceq _N$-comparable if and only if ${{\,\mathrm{\texttt{C}}\,}}(u)\subseteq {{\,\mathrm{\texttt{C}}\,}}(v)$ or ${{\,\mathrm{\texttt{C}}\,}}(v)\subseteq {{\,\mathrm{\texttt{C}}\,}}(u)$	Definition 12
Semi-regular	*N* is shortcut-free and satisfies (PCC)	Definition 13
Cluster network	*N* satisfies (PCC), and, three additional properties based on the clusters and respective vertices in *N*	Definition 14
Tree-child	For every $v\in V^0(N)$, there is a “tree-child,” i.e., $u\in {{\,\textrm{child}\,}}(v)$ with ${{\,\textrm{indeg}\,}}(u)=1$	Definition 16
Normal	*N* is tree-child and shortcut-free	Definition 17
Tree-based	There is a base tree *T* of *N* that can be obtained from *N* in a prescribed manner	Definition 18
Cluster-lca (CL)	${{\,\textrm{lca}\,}}({{\,\mathrm{\texttt{C}}\,}}(v))$ is defined for all $v\in V(N)$	Definition 21
lca-network	${{\,\textrm{lca}\,}}(A)$ is well defined, i.e., if $\vert {{\,\textrm{LCA}\,}}(A)\vert =1$ for all non-empty subsets $A\subseteq X$	Definition 22
Strong lca-network	*N* is an lca-network and, for every non-empty subset $A\subseteq X$, there are $x,y\in A$ such that ${{\,\textrm{lca}\,}}(\{x,y\})={{\,\textrm{lca}\,}}(A)$	Definition 23
Galled tree	Every non-trivial block in *N* is an (undirected) cycle	Definition 26
Conventional	(i) All leaves have indegree at most 1 and (ii) Every hybrid vertex is contained in a unique non-trivial block	Definition 30
Quasi-binary	${{\,\textrm{indeg}\,}}_N(w)=2$ and ${{\,\textrm{outdeg}\,}}_{N}(w)=1$ for every hybrid vertex $w\in V(N)$ and, additionally, ${{\,\textrm{outdeg}\,}}_N(\max B) = 2$ for every non-trivial block *B* in *N*	Definition 32

**Table 2:** Properties of clustering systems considered in this paper

Clustering system $\mathscr {C}$ is/satisfies		References
Hierarchy	For all $C,C'\in \mathscr {C}$, it holds $C\cap C'\in \{\emptyset ,C,C'\}$	Definition 5
Closed	$\mathscr {C}$ is closed under intersection, i.e., $\bigcap _{C\in \mathscr {C}'} C \in \mathscr {C}{\cup \!\!\!\cdot }\,\,\{\emptyset \}$ holds for all $\mathscr {C}'\subseteq \mathscr {C}$	Definition 10
Pre-binary	For every pair $x,y\in X$, there is a unique inclusion-minimal cluster *C* such that $\{x,y\}\subseteq C$	Definition 20
Binary	Pre-binary and, for every $C\in \mathscr {C}$, there is a pair of vertices $x,y\in X$ such that *C* is the unique inclusion-minimal cluster containing *x* and *y*	Definition 24
Weak hierarchy	For all $C_1,C_2,C_3\in \mathscr {C}$, it holds $C_1\cap C_2\cap C_3 \in \{C_1\cap C_2,C_1\cap C_3, C_2\cap C_3\}$	Definition 24
(L)	$C_1\cap C_2=C_1\cap C_3$ for all $C_1,C_2,C_3\in \mathscr {C}$ where $C_1$ overlaps both $C_2$ and $C_3$	Definition 25
(N3O)	$\mathscr {C}$ contains no three distinct pairwise overlapping clusters	Definition 27
Paired hierarchy	Every $C\in \mathscr {C}$ overlaps with at most one other cluster in $\mathscr {C}$	Definition 29
(2-Inc)	For all clusters $C\in \mathscr {C}$, there are at most two inclusion-maximal clusters $A,B\in \mathscr {C}$ with $A,B\subsetneq C$ and at most two inclusion-minimal clusters $A,B\in \mathscr {C}$ with $C\subsetneq A,B$	Definition 31

A network *N* with leaf set *X* is often called a *network on* *X*. Two networks $N_1 =(V_1,E_1)$ and $N_2=(V_2,E_2)$ on *X* are *graph isomorphic*, in symbols $N_1\sim N_2$, if there is a *graph isomorphism between* $N_1$ *and* $N_2$, i.e., a bijection $\varphi :V_1\rightarrow V_2$ such that $(u,v)\in E_1$ if and only if $(\varphi (u),\varphi (v))\in E_2$ for all $u,v\in V_1$. Moreover, if additionally $N_1$ and $N_2$ are networks on the same leaf set *X*, we say that $N_1$ and $N_2$ are *isomorphic* in symbols $N_1\simeq N_2$ if $N_1\sim N_2$ (via the graph isomorphism $\varphi$) and $\varphi (x)=x$ for all $x\in X$. We say that a network *N* on *X* is *unique* w.r.t. some property (or some set of properties), if $N\simeq N'$ for every network $N'$ that also satisfies the desired property (or properties).

Many studies into phylogenetic networks require that reticulation events and speciation events are separated, i.e., ${{\,\textrm{outdeg}\,}}(v)=1$ for all hybrid vertices.

#### Definition 2

A network is *separated* if all its hybrid vertices have outdegree 1.

In particular, all leaves have indegree 1 in a separated network (or indegree zero if the network consists of a single vertex).

The properties *phylogenetic* and *separated* are part of the definition of networks in many publications in the field, see, e.g., Jetten and van Iersel (2018); Pons et al. (2019); Zhang (2019). However, we opted for the more general definition of networks for several reasons. On the one hand, we aim to explore which restrictions are actually needed to establish the relationship of different properties or classes of networks. On the other hand, separated networks do not include regular networks (Baroni et al. 2004), which are, as we shall see, a class of networks that is intimately linked with clustering systems.

An even more restrictive class of networks that is often considered are binary networks (Gambette and Huber 2012; Bordewich and Semple 2016; Kong et al. 2022):

#### Definition 3

A network is *binary* if every tree vertex *v* is either a leaf or has ${{\,\textrm{outdeg}\,}}(v)=2$, and every hybrid vertex *v* satisfies ${{\,\textrm{indeg}\,}}(v)=2$ and ${{\,\textrm{outdeg}\,}}(v)=1$.

By construction, binary networks are always phylogenetic and separated.

Throughout this paper, several other properties and distinct classes of networks are considered. For convenience, all these types are listed in Table 1. More formal definitions or more precise explanations are given in the remainder of the paper. A further essential ingredient to our paper are clusters and clustering systems as defined next.

#### Definition 4

Let *N* be a network with vertex set *V*, leaf set *X*, and partial order $\preceq _N$. Then, for each $v\in V$, the associated *cluster* is ${{\,\mathrm{\texttt{C}}\,}}(v){:}{=}{{\,\mathrm{\texttt{C}}\,}}_N(v){:}{=}\{x\in X\mid x\preceq _N v\}$. Furthermore, we write $\mathscr {C}{:}{=}\mathscr {C}_N{:}{=}\{ {{\,\mathrm{\texttt{C}}\,}}(v)\mid v\in V\}$.

Note that ${{\,\mathrm{\texttt{C}}\,}}(v)={{\,\mathrm{\texttt{C}}\,}}(w)$ may be possible for distinct $v,w\in V$. However, $\mathscr {C}$ is considered as a *set* and thus each cluster appears only once in $\mathscr {C}_N$. The clusters in $\mathscr {C}_N$ are usually called the *hardwired clusters* of *N*, see, e.g., Huson and Scornavacca (2011).

#### Definition 5

(Barthélemy and Brucker 2008; Semple and Steel 2003) A *clustering system* on *X* is a set $\mathscr {C}\subseteq 2^X$ such that (i) $\emptyset \notin \mathscr {C}$, (ii) $X\in \mathscr {C}$, and (iii) $\{x\}\in \mathscr {C}$ for all $x\in X$. A clustering system is a *hierarchy* if it does not contain pairwise overlapping sets.

**Fig. 1:**
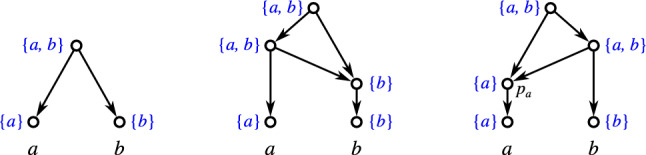
Three non-isomorphic (binary) level-1 networks (cf. Definition 9) with the same clustering system $\mathscr {C} = \{\{a\},\{b\},\{a,b\}\}$. While they are indistinguishable in terms of their clustering systems, they are encoded by their multisets of clusters, see Theorem 15, i.e., they are distinguished by the multiplicities of the clusters $\{a\}$, $\{b\}$, and $\{a,b\}$

We will mainly focus on clustering systems $\mathscr {C}_N$ of networks *N* (cf. Lemma 14). As shown in Fig. 1, the information conveyed by $\mathscr {C}_N$ is often insufficient to determine *N*, i.e., there are non-isomorphic networks *N* and $N'$ for which $\mathscr {C}_N = \mathscr {C}_{N'}$. A natural generalization is to consider the *multiset of clusters* $\mathscr {M}_N$, in which each cluster $C\in \mathscr {C}_N$ appears once for every vertex $v\in V(N)$ with ${{\,\mathrm{\texttt{C}}\,}}(v)=C$. We say that $\mathscr {M}_N$ *encodes* *N* within a given class $\mathbb {P}$ of networks if $N,N'\in \mathbb {P}$ and $\mathscr {M}_{N'}=\mathscr {M}_N$ implies $N'\simeq N$.

As for networks, we will also consider plenty of different types of clustering systems equipped with certain properties and, for convenience, list them in Table 2.

### Arc-expansion and arc-contraction

As mentioned above, often only separated networks are considered, stipulating that (1) leaves, i.e., vertices *v* with ${{\,\textrm{outdeg}\,}}(v)=0$ have ${{\,\textrm{indeg}\,}}(v)=1$; (2) hybrid vertices *v* have ${{\,\textrm{indeg}\,}}(v)\ge 2$ and ${{\,\textrm{outdeg}\,}}(v)=1$. Such networks are obtained from the ones in Definition 1 by means of a simple refinement operation that replaces every “offending” vertex by a pair of vertices connected by single arc. More precisely, we define the following operation on a network *N*, which is also part of (Alg. 6.4.2 Huson and Scornavacca 2011):

$${{\,\mathrm{\textsc {expd}}\,}}(v)$$
Create a new vertex $v'$, replace arcs (*u*, *v*) by $(u,v')$ for all $u\in {{\,\textrm{par}\,}}_N (v)$, and add the arc $(v',v)$.

**Fig. 2:**
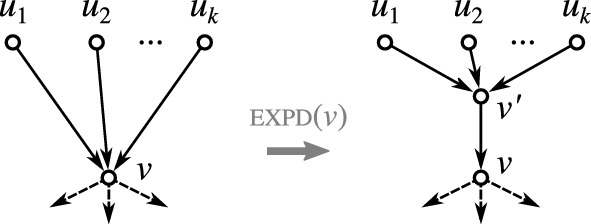
The expansion operation ${{\,\mathrm{\textsc {expd}}\,}}(v)$ introduces a new vertex $v'$ that becomes the single parent of *v* and the *k* original parents of *v* become parents of $v'$. Note that *v* may be an inner vertex or a leaf

The operation ${{\,\mathrm{\textsc {expd}}\,}}(v)$ is illustrated in Fig. 2. It will also useful to consider the reversed operation for arcs $(v',v)$ that are not shortcuts:

$${{\,\mathrm{\textsc {cntr}}\,}}(v',v)$$
Replace arcs $(u,v')$ by (*u*, *v*) for all $u\in {{\,\textrm{par}\,}}_N (v')\setminus {{\,\textrm{par}\,}}_N (v)$; replace arcs $(v',w)$ by (*v*, *w*) for all $w\in {{\,\textrm{child}\,}}_N (v')\setminus {{\,\textrm{child}\,}}_N (v)$; and finally delete $(v',v)$ and $v'$.

**Fig. 3:**
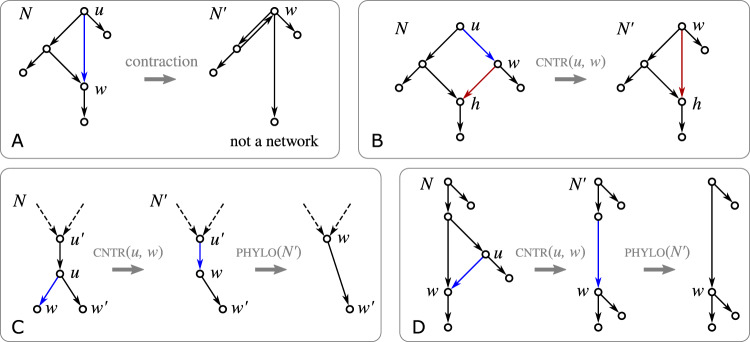
Complications arising in the contraction of arcs. The arcs to be contracted are highlighted in blue. **A** Contraction of a shortcut (*u*, *w*) introduced directed cycles. **B** Application of ${{\,\mathrm{\textsc {cntr}}\,}}(u,w)$ to a shortcut-free network *N* can result in a network $N'$ that contains a shortcut (r.h.s., shortcut indicated by the red arc). **C**, **D** Contraction of an arc (*u*, *w*) in a phylogenetic network *N* can yield a network $N'$ that is no longer phylogenetic. Application of ${{\,\mathrm{\textsc {phylo}}\,}}(N')$ can resolve this issue (color figure online)

The notation ${{\,\mathrm{\textsc {cntr}}\,}}$ is chosen in compliance with the literature where arc contraction is a commonly used operation. For our purpose, however, it will be useful to have this more formal definition in order to precisely keep track of the vertex sets upon execution of multiple operations. Observe that, e.g., since $(u,v'),(u,v)\in E$ is possible, applying first ${{\,\mathrm{\textsc {cntr}}\,}}(v',v)$ and then ${{\,\mathrm{\textsc {expd}}\,}}(v)$ does not necessarily yield a network that is isomorphic to the original network. Furthermore, we remark that the condition that $(v',v)$ is not a shortcut cannot be dropped since otherwise directed cycles are introduced (cf. Fig. 3A).

We are now in the position to define least-resolved networks:

#### Definition 6

A network *N* is *least-resolved* (w.r.t. its clustering system $\mathscr {C}{:}{=}\mathscr {C}_N$) if there is no network $N'$ with $\mathscr {C}_{N}=\mathscr {C}_{N'}$ that can be obtained from *N* by a non-empty series of shortcut removal and application of ${{\,\mathrm{\textsc {cntr}}\,}}(v',v)$ for some arc $(v',v)$ that is not a shortcut.

In many applications, phylogenetic networks are considered. However, ${{\,\mathrm{\textsc {cntr}}\,}}(w',w)$ applied on a phylogenetic network may result in a non-phylogenetic network. By way of example, see Fig. 3C, if *u* is a tree vertex with parent $u'$ and two children *w* and $w'$ which are leaves, then ${{\,\mathrm{\textsc {cntr}}\,}}(u,w)$ will “locally” result in a path with arcs $(u',w)$ and $(w,w')$, i.e., ${{\,\textrm{indeg}\,}}(w) = {{\,\textrm{outdeg}\,}}(w)=1$. Similarly, ${{\,\mathrm{\textsc {cntr}}\,}}(u,w)$ in a block that contains a shortcut can result in a network $N'$ that is not phylogenetic, see Fig. 3D. To circumvent this issue, we must “suppress” *w* to obtain a phylogenetic network. To this end, we define the following operation to make a network *N* phylogenetic:

$${{\,\mathrm{\textsc {phylo}}\,}}(N)$$
Repeatedly apply ${{\,\mathrm{\textsc {cntr}}\,}}(u,w)$ for an arc (*u*, *w*) such that ${{\,\textrm{outdeg}\,}}(u)=1$ and ${{\,\textrm{indeg}\,}}(u)\le 1$ until no such operation is possible.

Now, contraction of an arc $(v',v)$ that is not a shortcut and “suppression” of superfluous vertices can be combined in:

$${{\,\mathrm{\textsc {cntr}}\,}}^{\star }(v',v)$$
Apply ${{\,\mathrm{\textsc {cntr}}\,}}(v',v)$ to obtain $N'$ and then ${{\,\mathrm{\textsc {phylo}}\,}}(N')$.

The term “ancestor-preserving”—which is rigorously defined below—has been used in particular for mappings between certain network (Huber and Scholz 2020; Hellmuth et al. 2019). For our purposes, a slightly simplified version is sufficient.

#### Definition 7

Let *N* and $N'$ be networks such that $V(N')\subseteq V(N)$. Then, *N* and $N'$ are $(N,N')$-*ancestor-preserving* if for all $v,v'\in V(N')$, it holds that $v\preceq _{N} v'$ if and only if $v\preceq _{N'} v'$.

#### Lemma 1

Let *N* be a network on *X* and $(u,w)\in E(N)$ be a shortcut. Then, removal of (*u*, *w*) in *N* results in a network $N'$ with leaf set *X* and $V(N)=V(N')$. Moreover, *N* and $N'$ are $(N,N')$-ancestor-preserving and ${{\,\mathrm{\texttt{C}}\,}}_N(v)={{\,\mathrm{\texttt{C}}\,}}_{N'}(v)$ holds for all $v\in V(N')=V(N)$. In particular, it holds $\mathscr {C}_N=\mathscr {C}_{N'}$.

#### Proof

Let *N* be a network on *X* and (*u*, *w*) be a shortcut in *N*. Since (*u*, *w*) is a shortcut, there is a $w'\in {{\,\textrm{child}\,}}(u)\setminus \{w\}$ such that $w\prec _N w'$. Hence, there is a $w'w$-path *P* in *N*. Since *N* is acyclic and $w'\prec _N u$, *u* is not a vertex in *P* since otherwise $u\preceq _{N} w'$. Therefore, *w* has indegree larger than 1 in *N*. In particular, there is a *uw*-path $P'$ in $N'$ formed by the arc $(u,w')$ and $w'w$-path *P*. Since removal of (*u*, *w*) only decreases the indegree of *w* and ${{\,\textrm{indeg}\,}}_N(w)\ge 2$, $\rho _N=\rho _{N'}$ is still the only vertex with indegree 0 in $N'$. Moreover, removal of arcs clearly preserves acyclicity, and thus, $N'$ is a rooted network.

Now, let $v,v'\in V(N)=V(N')$. If $v \not \preceq _{N} v'$, then there is no $v'v$-path in *N*. Clearly, removal of arcs changes nothing about this and thus $v \not \preceq _{N'} v'$. Suppose now that $v \preceq _{N} v'$ and thus let $P_{v'v}$ be a $v'v$-path in *N*. If $P_{v'v}$ does not contain the arc (*u*, *w*), then $P_{v'v}$ is still a $v'v$-path in $N'$. Otherwise, the path obtained from $P_{v'v}$ by replacing (*u*, *w*) by the *uw*-path $P'$ is a $v'v$-path in $N'$. Hence, $v \preceq _{N} v'$ holds in both cases. In summary, we have $v\preceq _{N} v'$ if and only if $v\preceq _{N'} v'$.

By the latter arguments, $N'$ is a network with leaf set *X* and we have $x\preceq _{N} v$ if and only if $x\preceq _{N'} v$ for all $x\in X$ and all $v\in V(N)=V(N')$. Therefore, $x\in {{\,\mathrm{\texttt{C}}\,}}_N(v)$ if and only if $x\in {{\,\mathrm{\texttt{C}}\,}}_{N'}(v)$ for all $x\in X$ and all $v\in V(N)=V(N')$, and thus, ${{\,\mathrm{\texttt{C}}\,}}_N(v)={{\,\mathrm{\texttt{C}}\,}}_{N'}(v)$. Together with $V(N)=V(N')$, this implies $\mathscr {C}_N=\mathscr {C}_{N'}$. $\square$

Note that deletion of a shortcut from a phylogenetic network does not necessarily result in a phylogenetic network.

#### Lemma 2

If a network *N* is shortcut-free and has no vertex of outdegree 1, then for every vertex $w\in V(N)\setminus \{\rho _N\}$, there is a vertex $v\in {{\,\textrm{child}\,}}_N({{\,\textrm{par}\,}}_N(w))$ such that *v* and *w* are $\preceq _N$-incomparable. In this case, *N* is phylogenetic.

#### Proof

Since *N* is shortcut-free, siblings $v',v''\in {{\,\textrm{child}\,}}_N(u)$, $v'\ne v''$ are $\preceq _N$-incomparable. Thus, there is $v\in {{\,\textrm{child}\,}}_N({{\,\textrm{par}\,}}_N(w))$ that is $\preceq _N$-incomparable with *w* if and only if ${{\,\textrm{par}\,}}_N(w)\ne \emptyset$ and ${{\,\textrm{outdeg}\,}}({{\,\textrm{par}\,}}_N(w))>1$. Both conditions are satisfied by assumption. $\square$

#### Lemma 3

Let *N* be a network on *X* and $(u,w) \in E(N)$ be an arc that is not a shortcut. Then, ${{\,\mathrm{\textsc {cntr}}\,}}(u,w)$ applied on *N* results in a network $N'$ with leaf set *X* or $X\setminus \{w\}$ and $V(N')=V(N)\setminus \{u\}$. Moreover, for all $v,v'\in V(N')$,

1. $$v\preceq _N v'$$ implies $$v \preceq _{N'} v'$$, and
2. $v\preceq _{N'} v'$ implies (i) $v \preceq _{N} v'$ or (ii) $w\preceq _N v'$ and $v\preceq _N w'$ for some $w'\in {{\,\textrm{child}\,}}_N(u)\setminus \{w\}$ that is $\preceq _{N}$-incomparable with *w*.

In particular, $v\prec _{N'} v'$ always implies $v\prec _{N} v'$ or *v* and $v'$ are $\preceq _{N}$-incomparable.

#### Proof

The proof is rather lengthy and technical and is, therefore, placed to “Expansion, contraction, and blocks” section in “Appendix.” $\square$

#### Lemma 4

Let *N* be a network on *X* and $(u,w)\in E(N)$ such that ${{\,\textrm{outdeg}\,}}_{N}(u)=1$. Then, ${{\,\mathrm{\textsc {cntr}}\,}}(u,w)$ results in a network $N'$ with leaf set *X* and $V(N')=V(N)\setminus \{u\}$ that is $(N,N')$-ancestor-preserving. Moreover, ${{\,\mathrm{\texttt{C}}\,}}_N(v)={{\,\mathrm{\texttt{C}}\,}}_{N'}(v)$ for all $v\in V(N')=V(N)\setminus \{u\}$ and, in particular, $\mathscr {C}_N=\mathscr {C}_{N'}$.

#### Proof

Let *N* be a network on *X* and $(u,w)\in E(N)$ such that ${{\,\textrm{outdeg}\,}}_{N}(u)=1$. Since an arc $(u,w)\in E(N)$ with ${{\,\textrm{outdeg}\,}}_{N}(u)=1$ cannot be a shortcut and satisfies ${{\,\textrm{child}\,}}_N(u)\setminus \{w\}=\emptyset$, and thus, condition (ii) in Lemma 3 cannot occur, $N'$ is a network with leaf set *X* or $X{\setminus }\{w\}$ and $(N,N')$-ancestor-preserving. Moreover, since *w* is the only out-neighbor of *u*, we do not add any out-neighbors for *w*. Hence, $N'$ has leaf set *X*.

By the latter argument, $N'$ is a network with leaf set *X* and we have $x\preceq _{N} v$ if and only if $x\preceq _{N'} v$ for all $x\in X$ and all $v\in V(N')=V(N){\setminus }\{u\}$. Therefore, $x\in {{\,\mathrm{\texttt{C}}\,}}_N(v)$ if and only if $x\in {{\,\mathrm{\texttt{C}}\,}}_{N'}(v)$ holds for all $x\in X$ and all $v\in V(N')=V(N)\setminus \{u\}$. Hence, we have ${{\,\mathrm{\texttt{C}}\,}}_N(v)={{\,\mathrm{\texttt{C}}\,}}_{N'}(v)$ for all $v\in V(N')=V(N)\setminus \{u\}$. Moreover, since *w* is the unique out-neighbor of *u*, one can easily verify that ${{\,\mathrm{\texttt{C}}\,}}_N(u)={{\,\mathrm{\texttt{C}}\,}}_N(w)$ (cf. Observation 5 for further arguments) and thus ${{\,\mathrm{\texttt{C}}\,}}_N(u)={{\,\mathrm{\texttt{C}}\,}}_{N'}(w)\in \mathscr {C}_{N'}$. Taken together, we obtain $\mathscr {C}_N=\mathscr {C}_{N'}$. $\square$

As an immediate consequence of Lemma 1 and 4, we obtain

#### Corollary 2

Every least-resolved network *N* is shortcut-free and does not contain vertices *v* with ${{\,\textrm{outdeg}\,}}_N(v)=1$.

#### Lemma 5

Let *N* be a network and $N'$ be obtained from *N* by applying ${{\,\mathrm{\textsc {expd}}\,}}(w)$ for some $w\in V(N)$. Then, $N'$ is a network such that *N* and $N'$ are $(N',N)$-ancestor-preserving. Moreover, ${{\,\mathrm{\texttt{C}}\,}}_N(v)={{\,\mathrm{\texttt{C}}\,}}_{N'}(v)$ for all $v\in V(N)\subseteq V(N')$ and, in particular, it holds $\mathscr {C}_N=\mathscr {C}_{N'}$. Moreover, if *N* is phylogenetic, then $N'$ is phylogenetic if and only if *w* is a hybrid vertex and ${{\,\textrm{outdeg}\,}}_{N}(w)\ne 1$.

#### Proof

Let *N* be a network on *X*. We show first that $N'$ is a network. By construction, *w* is the only vertex in *N* whose in-neighborhood changes and it has the new vertex $w'$ as its unique in-neighbor in $N'$. If $w\ne \rho _N$, then *w* has at least one in-neighbor in *N*, which becomes an in-neighbor of $w'$. Hence, $\rho _N$ is still the only vertex with indegree 0 in $N'$. If $w=\rho _{N}$, then it has no in-neighbors in *N* and thus $w'$ has no in-neighbors in $N'$. Together with the fact that *w* no longer has indegree 0, $w'$ is the only vertex with indegree 0 in $N'$ in this case. Now, assume that $N'$ contains a directed cycle *K* comprising the vertices $v_1, v_2, \dots , v_k$, $k\ge 2$, in this order, i.e., $(v_i,v_{i+1})$, $1\le i\le k-1$ and $(v_k,v_1)$ are arcs in $N'$. If all arcs in *K* are in *N*, then *K* is a directed cycle in *N*, a contradiction. If *K* contains an arc that is not in *N*, then *K* must contain the new vertex $w'$ since all new arcs are incident with $w'$. Suppose w.l.o.g. that $w'=v_1$. Since $w'$ has a unique out-neighbor *w* and exactly the vertices of ${{\,\textrm{par}\,}}_N(w)$ as in-neighbors, we must have $v_2=w$ and $v_k\in {{\,\textrm{par}\,}}_N(w)$, respectively. In particular, this implies $v_2\ne v_k$ and $(v_k,v_2)\in E(N)$. Since $w'$ appears in *K* at most once, $(v_k, v_1)$ and $(v_1, v_2)$ are the only arcs in *K* that are incident with $w'$, and thus, all other arcs of *K* are also arcs in *N*. In particular, there is a $v_2 v_k$-path in *N*. Together with the fact that $(v_k,v_2)\in E(N)$, this implies that *N* contains a directed cycle, a contradiction. Therefore, $N'$ must be acyclic. Since moreover $N'$ has a unique root, it is a network.

The operation ${{\,\mathrm{\textsc {expd}}\,}}(w)$ on a network *N* creates a network $N'$ with an additional vertex $w'$ such that *w* is the unique out-neighbor of $w'$ and ${{\,\textrm{par}\,}}_{N'}(w') = {{\,\textrm{par}\,}}_N (w)$. Therefore, *N* is recovered from $N'$ by applying ${{\,\mathrm{\textsc {cntr}}\,}}(w',w)$. This observation together with Lemma. 4 implies that *N* and $N'$ are $(N',N)$-ancestor-preserving.

Suppose now that *N* is phylogenetic. Assume first that *w* is a hybrid vertex and ${{\,\textrm{outdeg}\,}}_{N}(w)\ne 1$. Then, by construction, the newly created vertex $w'$ satisfies ${{\,\textrm{indeg}\,}}_{N'}(w')={{\,\textrm{indeg}\,}}_{N}(w)\ge 2$ and, moreover, we have ${{\,\textrm{outdeg}\,}}_{N'}(w)={{\,\textrm{outdeg}\,}}_{N}(w)\ne 1$. The only other vertices whose neighborhoods are affected are the vertices $u\in {{\,\textrm{par}\,}}_N(w)$. More precisely, their out-neighbor *w* is replaced by an out-neighbor $w'$ and thus ${{\,\textrm{indeg}\,}}_{N'}(u)={{\,\textrm{indeg}\,}}_{N}(u)$ and ${{\,\textrm{outdeg}\,}}_{N'}(u)={{\,\textrm{outdeg}\,}}_{N}(u)$ for any $u\in {{\,\textrm{par}\,}}_N(w)$. Together with the fact that *N* is phylogenetic, the latter arguments imply that there is no vertex $v\in V(N')$ with ${{\,\textrm{outdeg}\,}}_{N'}(v)=1$ and ${{\,\textrm{indeg}\,}}_{N'}(v)\le 1$. Hence, $N'$ is phylogenetic. Now, assume that *w* is a not hybrid vertex or ${{\,\textrm{outdeg}\,}}_{N}(w)= 1$. If *w* is not a hybrid vertex, then ${{\,\textrm{indeg}\,}}_{N'}(w')={{\,\textrm{indeg}\,}}_{N}(w)\le 1$. Moreover, ${{\,\textrm{outdeg}\,}}_{N'}(w')=1$ holds by construction, and thus, $N'$ is not phylogenetic. If ${{\,\textrm{outdeg}\,}}_{N}(w)= 1$, then ${{\,\textrm{outdeg}\,}}_{N'}(w)= 1$ since the out-neighborhood of *w* does not change. In addition, $w'$ is the unique in-neighbor of *w* in $N'$ by construction. Hence, $N'$ is not phylogenetic. In summary, it holds that $N'$ is phylogenetic if and only if *w* is a hybrid vertex and ${{\,\textrm{outdeg}\,}}_{N}(w)\ne 1$.

By the latter arguments, $N'$ is a network with leaf set *X*. The newly created vertex $w'$ has a unique child *w*. The statement “${{\,\mathrm{\texttt{C}}\,}}_N(v)={{\,\mathrm{\texttt{C}}\,}}_{N'}(v)$ for all $v\in V(N)\subseteq V(N')$ and, in particular, $\mathscr {C}_N=\mathscr {C}_{N'}$” therefore follows immediately from Lemma 4 and the fact that *N* is recovered from $N'$ by applying ${{\,\mathrm{\textsc {cntr}}\,}}(w',w)$. $\square$

The following result shows that the expansion operation does not introduce shortcuts and is an immediate consequence of Lemma 72 in “Expansion, contraction, and blocks” section.

#### Corollary 3

Let *N* be a network and $N'$ be the network obtained from *N* by applying ${{\,\mathrm{\textsc {expd}}\,}}(w)$ for some $w\in V(N)$. Then, *N* is shortcut-free if and only if $N'$ is shortcut-free.

We remark that an analogue of Corollary 3 does not hold for the contraction operation ${{\,\mathrm{\textsc {cntr}}\,}}(u,w)$. Figure 3B shows an example where contraction introduces a shortcut.

### Blocks

The blocks of *N* will play a key role in the following. We first establish several technical results that will allow us efficiently reason about the block structure of a network.

#### Lemma 6

Let *N* be a network and $u,v\in V(N)$ be two $\preceq _N$-incomparable vertices. Then, *u* and *v* are connected by an undirected path *P* that contains at least 3 vertices and of which all inner vertices *w* satisfy $u\prec _N w$ or $v\prec _N w$. In addition, we have $w\not \preceq _N u$ and $w\not \preceq _N v$ for every such inner vertex *w*.

#### Proof

There are directed paths $P_u$ and $P_v$ from $\rho _N$ to both *u* and *v*, respectively. Let $w^*$ be the $\preceq _N$-minimal vertex of $P_u$ that is also a vertex of $P_v$, which exists since at least $\rho _N$ is contained in both paths. It must hold that $w^*\notin \{u,v\}$ since otherwise *u* and *v* would be $\preceq _N$-comparable. In particular, $u \prec _N w^*$ and $v \prec _N w^*$. Let $P'_u$ and $P'_v$ be the subpaths of $P_u$ and $P_v$ from $w^*$ to *u* and *v*, respectively. By construction, $P'_u$ and $P'_v$ only have vertex $w^*$ in common, which moreover is an outer vertex of both paths. Now, consider the path *P* that is the union of the underlying undirected version of $P'_u$ and $P'_v$. By construction, *P* contains at least the three vertices *u*, *v*, and $w^*$ and all of its inner vertices *w* satisfy $u\prec _N w$ or $v\prec _N w$. Assume, for contradiction, that $w\preceq _N u$ for some of these inner vertices. Since $u\prec _N w$ is not possible, we must have $v\prec _N w$. But then $v\prec _N w$ and $w\preceq _N u$ imply that *v* and *u* are $\preceq _N$-comparable, a contradiction. Hence, $w\not \preceq _N u$ must hold. One shows analogously that $w\not \preceq _N v$. $\square$

Paths of the form described in Lemma 6 connecting two leaves *u* and *v* are called “up-down-paths” in Bordewich and Semple (2016).

#### Lemma 7

Let *B* be a block in a network *N* and $u,v\in V(B)$ such that $v\preceq _{N} u$. Then, every *uv*-path in *N* is completely contained in *B*.

#### Proof

Let *P* be a *uv*-path in *N*, which exists since $v\preceq _{N} u$. The statement holds trivially if *B* is an isolated vertex, $v=u$, or *B* is the arc (*u*, *v*). Thus, suppose *B* is a non-trivial block. Suppose, for contradiction, there is a vertex $w\in V(P){\setminus } V(B)$. Let $w_a$ and $w_d$ be the $\preceq _{N}$-minimal ancestor and the $\preceq _{N}$-maximal descendant, resp., of *w* in *P* (both of which exist since $u,v\in V(P)$). Consider the subpath $P'$ of *P* from $w_a$ to $w_d$. By Proposition 1, the subgraph of *N* obtained by adding $P'$ to *B* is again biconnected. Together with $w\in V(P')\setminus V(B)$, this contradicts that *B* is a block. Hence, such a vertex cannot exist. Therefore, and since blocks are always induced subgraphs, the statement follows. $\square$

#### Lemma 8

Every block *B* in a network *N* has a unique $\preceq _N$-maximal vertex $\max B$. In particular, for every $v\in V(B)$, there is a directed path from $\max B$ to *v* and every such path is completely contained in *B*.

#### Proof

The statement is trivial for a block that consists only of a single vertex or arc. Otherwise, suppose there are two distinct $\preceq _N$-maximal vertices $v_1$ and $v_2$ in *B*. By assumption, $v_1$ and $v_2$ must be $\preceq _N$-incomparable. By Lemma 6, $v_1$ and $v_2$ are connected by an undirected path *P* that contains at least 3 vertices and of which all inner vertices *w* satisfy $v_1\prec _N w$ or $v_2\prec _N w$. By $\preceq _N$-maximality of $v_1$ and $v_2$, none of these inner vertices can be contained in *B*. By Proposition 1, adding *P* to *B* preserves biconnectivity, and thus, *B* is not a maximal biconnected subgraph, a contradiction. In particular, for every $v\in V(B)$, we have $v\preceq _{N}\max B$, i.e., there is a path from $\max B$ to *v* and by Lemma 7, each every such path is completely contained in *B*. $\square$

#### Corollary 4

If *B* is a non-trivial block in network *N*, then $\max B$ has at least two out-neighbors in *B*.

#### Proof

Since *B* is non-trivial, $\max B$ lies on an undirected cycle in *B* and thus is incident with two distinct vertices in *B*. By $\preceq _{N}$-maximality of $\max B$ in *B*, these must be out-neighbors of $\max B$. $\square$

#### Lemma 9

Let *N* be a network and suppose that $v\in V(N)$ is contained in the blocks *B* and $B'$ of *N*. If $v\notin \{\max B, \max B'\}$, then $B=B'$.

#### Proof

Assume that vertex *v* is contained in the blocks *B* and $B'$ of *N* but $v\notin \{\max B, \max B'\}$. By Lemma 8, there exists a directed path *P* in *B* from $\max B$ to *v*. Similarly, there is a directed path $P'$ in $B'$ from $\max B'$ to *v*. Since $v\notin \{\max B, \max B'\}$, both *P* and $P'$ contain at least one arc.

Assume first that *P* and $P'$ share an arc *e* and thus, that *B* and $B'$ share the arc *e*. In this case, contraposition of Observation 2 implies that $B=B'$. Hence, in the following we assume that *P* and $P'$ are arc-disjoint.

Consider first the case $\max B' \preceq _{N} \max B$. Let *u* be the unique $\preceq _N$-minimal vertex in *P* such that $\max B'\preceq _{N} u$. Together with $v\prec _N \max B'$, this implies that $u\ne v$. Let $P_{u,v}$ be the subpath of *P* from *u* to *v* and note that $P_{u,v}$ contains at least one arc. Since $\max B'\preceq _{N} u$, we can find a directed path $P_{u, \max B'}$ (possible only containing a single vertex $u=\max B'$) from *u* to $\max B'$. The paths $P_{u,v}$ and $P_{u, \max B'}$ only have vertex *u* in common since *u* is the unique $\preceq _N$-minimal vertex in *P* with $\max B'\preceq _{N} u$. Since *N* is acyclic, $P_{u, \max B'}$ and $P'$ are arc-disjoint. In summary, $P'$, $P_{u,v}$, and $P_{u, \max B'}$ are pairwise arc-disjoint. Hence, $\max B'$ and *v* are connected by two arc-disjoint undirected paths that correspond to $P'$ and the union of $P_{u,v}$ and $P_{u, \max B'}$. Therefore, $\max B'$ and *v* are contained in a common block $B''$. In particular, *B* and $B''$ share all arcs in $P_{u,v}$, and thus at least one arc. Similarly, $B'$ and $B''$ share all arcs in $P'$, and thus at least one arc. By Observation 2, it follows that $B=B''=B'$. Similarly, $\max B \preceq _{N} \max B'$ implies $B=B'$.

Suppose now that $\max B$ and $\max B'$ are $\preceq _N$-incomparable. Recall that *P* and $P'$ are arc-disjoint and each contain at least one arc. Let $\breve{P}$ be the undirected path corresponding to the union of *P* and $P'$ and observe that all of its inner vertices *w* that satisfy $w\preceq _N \max B$ or $w\preceq _N \max B'$. Since $\max B$ and $\max B'$ are $\preceq _N$-incomparable, Lemma 6 implies that they are connected by an undirected path ${\mathop {P}^{\frown }}$ that contains at least 3 vertices and of which all inner vertices $w'$ satisfy $w'\not \preceq _N \max B$ and $w'\not \preceq _N \max B'$. As a consequence, $\breve{P}$ and ${\mathop {P}^{\frown }}$ only have their endpoints $\max B$ and $\max B'$ in common. Hence, $\max B$ and $\max B'$ are contained in a common block $B''$. In particular, *B* and $B''$ share all arcs in *P*, and thus at least one arc. Similarly, $B'$ and $B''$ share all arcs in $P'$, and thus at least one arc. By Observation 2, it follows that $B=B''=B'$. $\square$

By definition, *N* is a tree if and only if it contains no undirected cycle, i.e., if all blocks are trivial. Thus, *N* is a tree if and only if there are no hybrid vertices.

#### Definition 8

Let *N* be a network and *B* a non-trivial block in *N* with terminal vertices $\{m_1,m_2,\dots , m_h\}$, $h\ge 1$. Then,

1 $$\begin{aligned} B^0 {:}{=}B\setminus \{ \max B, m_1, m_2,\dots m_h\} \end{aligned}$$

is the *interior* of *B*.

As an immediate consequence of Lemma 9, we have

#### Observation 4

Let $B_1$ and $B_2$ be two distinct blocks in *N*. Then, $B_1^0\cap B_2^0=\emptyset$.

#### Lemma 10

Let *N* be a network and $w\in V(N)$ be a hybrid vertex. Then, *w* and all of its in-neighbors are contained in a non-trivial block *B*.

#### Proof

Let *w* be a hybrid vertex, i.e., ${{\,\textrm{indeg}\,}}_N(w)\ge 2$, and let *v* and $v'$ be two distinct in-neighbors of *w*. If $v'\prec _N v$, then there is a directed path *P* from *v* to $v'$ that contains at least one arc. Moreover, *w* is not a vertex of *P* since otherwise $v'\preceq _{N} w$ would contradict $w\prec _N v'$. Therefore, *P* together with *w* and arcs *vw* and $v'w$ form an undirected cycle. An analogous argument applies if $v\prec _N v'$. If *v* and $v'$ are $\preceq _{N}$-incomparable, then Lemma 6 implies that they are connected by an undirected path *P* that contains at least 3 vertices and of which all inner vertices $w'$ satisfy $w'\not \preceq _N v$ and $w'\not \preceq _N v'$. Together with $w\prec _N v,v'$, this implies that *w* is not contained in *P*. Therefore, *P* together with *w* and arcs *vw* and $v'w$ form an undirected cycle. In summary, in all cases, *w* is contained in a non-trivial block $B_{v'}$ that, in particular, also contains *v*, $v'$, and the arc *vw*. Since $v'$ was chosen arbitrarily among the in-neighbors of *w* that are distinct from *v* and the blocks $B_{v'}$ for all of these vertices share the arc *vw*, Observation 1 implies that *w* and all of its in-neighbors are contained in a non-trivial block *B*. $\square$

A hybrid vertex *w* is *properly contained* in a block *B* if $w\in V(B)$ and all of its in-neighbors are also contained in *B*. As an immediate consequence of Lemma 10, every hybrid vertex is properly contained in exactly one block.

#### Lemma 11

Let *N* be a network, *w* a hybrid vertex in *N*, and *B* be a block of *N*. Then, the following statements are equivalent:

1. *w* is properly contained in *B*, i.e., *w* and all of its parents are contained in *B*.
2. *w* and one of its parents *u* are contained in *B*.
3. $$w\in V(B)\setminus \{ \max B \}$$.

#### Proof

(3) $\implies$ (2). Since $\max B$ is the unique $\preceq _{N}$-maximal vertex in *B*, we have $w\prec _N \max B$. By Lemma 8, there is a directed path from $\max B$ to *w* that is completely contained in *B*. Clearly, *P* contains a parent of *w*, which is there also contained in *B*. *(2)* $\implies$ (1). By Lemma 10, *w* and all of its parents are contained in a non-trivial block $B'$. Hence, *B* and $B'$ share the distinct vertices *w* and *u*. By Observation 1, $B=B'$. *(1)* $\implies$ (3). If *w* and all of its (at least two) parents are contained in *B*, then clearly $w\in V(B)\setminus \{ \max B \}$. $\square$

As a consequence, if a hybrid vertex *w* is contained in a block *B* but not properly contained, then it must hold $w=\max B$. This motivates the following definition of level-*k* networks:

#### Definition 9

A network *N* is *level*-*k* if each block *B* of *N* contains at most *k* hybrid vertices distinct from $\max B$.

**Fig. 4:**
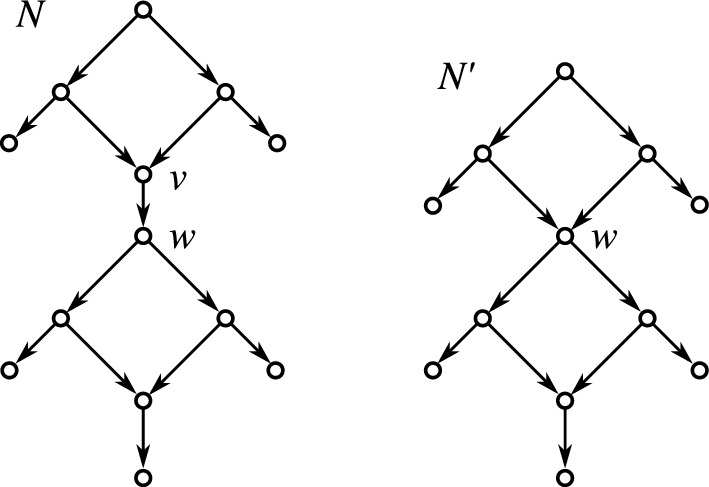
The network *N* contains a hybrid vertex *v* with ${{\,\textrm{outdeg}\,}}(v)=1$. Network $N'$ is obtained from *N* by contraction of the arc (*v*, *w*), i.e., the operation ${{\,\mathrm{\textsc {cntr}}\,}}(v,w)$ which preserves vertex *w*. Vertex *w* is now a hybrid vertex that is contained in two blocks of $N'$. However, only the upper block properly contains it

Equivalently, by Lemma 10, *N* is level-*k* if each block *B* of *N* properly contains at most *k* hybrid vertices. In Choy et al. (2004), level-*k* networks are simply defined by having no more than *k* hybrid vertices within any given block. We note that this is equivalent to our definition in a setting where hybrid vertices are restricted to having outdegree 1. Definition 9 also accommodates the contraction of out-arcs of hybrid vertices *v* with ${{\,\textrm{outdeg}\,}}(v)=1$, see Fig. 4.

The following two lemmas show that neither arc contraction nor expansion increases the level of a network. Since their proofs are rather lengthy and technical, they are given in “Expansion, contraction, and blocks” section in “Appendix.”

#### Lemma 12

Let *N* be a network, $(w',w) \in E(N)$ be an arc that is not a shortcut, and $N'$ be the network obtained from *N* by applying ${{\,\mathrm{\textsc {cntr}}\,}}(w',w)$. If *N* is level-*k*, then $N'$ is also level-*k*.

#### Proof

See “Expansion, contraction, and blocks” section in “Appendix.” $\square$

**Fig. 5:**
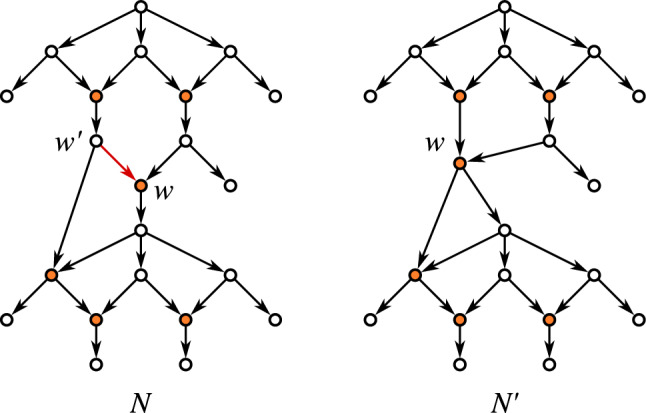
The level-3 network $N'$ is obtained from the level-6 network by application of ${{\,\mathrm{\textsc {cntr}}\,}}(w',w)$. The hybrid vertices are highlighted in orange (color figure online)

The converse of Lemma 12, however, is not true as shown by the example in Fig. 5. This example also shows that even mitigated versions “*if* $N'$ *is level*-*k*, *then* *N* *is level*-$(k+1)$” do not hold. As an immediate consequence of the definition of ${{\,\mathrm{\textsc {phylo}}\,}}(N)$, Lemma 4 and 12, we obtain

#### Corollary 5

Let *N* be a level-*k* network. Then, the network $N'$ obtained by operation ${{\,\mathrm{\textsc {phylo}}\,}}(N)$ is a phylogenetic level-*k* network such that $\mathscr {C}_{N}=\mathscr {C}_{N'}$.

#### Lemma 13

Let *N* be a network and $N'$ be the network obtained from *N* by applying ${{\,\mathrm{\textsc {expd}}\,}}(w)$ for some $w\in V(N)$. Then, *N* is level-*k* if and only if $N'$ is level-*k*.

#### Proof

See “Expansion, contraction, and blocks” section in “Appendix.” $\square$

The definition of ${{\,\mathrm{\textsc {phylo}}\,}}(N)$ and ${{\,\mathrm{\textsc {cntr}}\,}}^{\star }(v',v)$ and Lemma 12 yield

#### Corollary 6

Let *N* be a level-*k* network. If $N'$ is the network obtained from *N* by applying ${{\,\mathrm{\textsc {phylo}}\,}}(N)$ or ${{\,\mathrm{\textsc {cntr}}\,}}^{\star }(v',v)$ for some arc $(v',v) \in E(N)$ that is not a shortcut, then $N'$ is phylogenetic and level-*k*.

### Clusters, Hasse diagrams, and regular networks

In this section, we consider general properties of the set of clusters $\mathscr {C}_N$ of a phylogenetic network as specified in Definition 4.

#### Lemma 14

For all networks *N* on *X*, the set $\mathscr {C}_N$ is a clustering system.

#### Proof

Every non-leaf vertex $v\in V\setminus X$ has at least one out-neighbor and *N* is acyclic and finite. Thus, every directed path in *N* can be extended to a directed path that eventually ends in a leaf, implying ${{\,\mathrm{\texttt{C}}\,}}(v)\ne \emptyset$. Since ${{\,\mathrm{\texttt{C}}\,}}(v)\ne \emptyset$ for all $v\in V$ and since *N* contains at least a root $\rho _N$ as a vertex, we have $\emptyset \notin \mathscr {C}_N$ and thus Condition (i) holds. Since $v\preceq _N \rho _N$ for all $v\in V$, we have ${{\,\mathrm{\texttt{C}}\,}}(\rho _N)=X$ and (ii) is satisfied. To see that Condition (iii) holds, observe that for all $x\in X$, we have ${{\,\textrm{outdeg}\,}}(x)=0$ and thus ${{\,\mathrm{\texttt{C}}\,}}(x)=\{x\}$. $\square$

This simple observation connects phylogenetic networks to a host of the literature on clustering systems, which have been studied with motivations often unrelated to evolution or phylogenetics (Jardine and Sibson 1971; Barthélemy and Brucker 2008; Janowitz 2010).

A particular difficulty in the characterization of certain types of networks by means of their clustering systems is that even rather simple clustering systems such as hierarchies can be explained by very complex networks.

#### Lemma 15

Let *n* be a positive integer. Then, for all $k\in \{0,2,\dots ,n\}$, there is a phylogenetic, shortcut-free level-*k* network *N* on *n* leaves that is not level-$(k-1)$ such that $\mathscr {C}_N$ is a hierarchy.

#### Proof

**Fig. 6:**
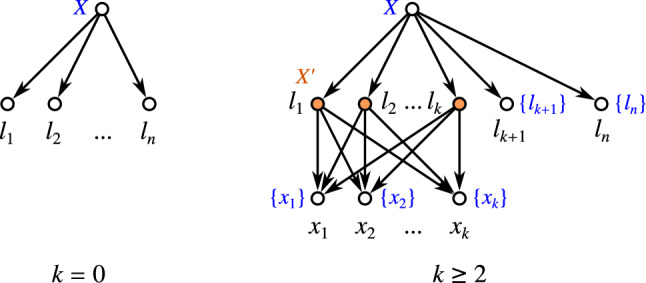
A generic framework that shows that, for every $n\ge 2$ and $k\in \{0,2,\dots ,n\}$, there is a phylogenetic, shortcut-free level-*k* network *N* on *n* leaves that is not level-$(k-1)$ and where $\mathscr {C}_N$ is a hierarchy. The network shown left is level-0 and its clustering system is trivially a hierarchy. The clustering system $\mathscr {C}_N$ of the level-*k* network *N* shown right consists of the clusters $X'{:}{=}\{x_1,\dots ,x_k\}$ (for which the corresponding vertices are highlighted in orange), $X = X'\cup \{l_{k+1}, \dots , l_n\}$, and the singletons and, therefore, $\mathscr {C}_N$ is a hierarchy (color figure online)

If $n=1$, then $k=0$ and the single vertex graphs serves as an example (since a network contains at least one vertex and thus a level-$(-1)$ cannot exist by definition). Let $n\ge 2$. For $k=0$, simply take a tree whose root is adjacent to the *n* leaves only. Again, this tree is level-0 but not level-$(-1)$. We refer to this tree as a star tree. For $k\ge 2$, take a star tree *T* and randomly collect *k* of its leaves $l_1,\dots l_k$. Now, add new leaves $x_1,\dots ,x_k$ and edges such that the induced subgraph $N[\{l_1,\dots l_k,x_1,\dots ,x_k\}]$ is graph isomorphic to a complete bipartite graphs where one part of the bipartition contains all $l_1,\dots ,l_k$ and the other part all $x_1,\dots ,x_k$ (see Fig. 6 for a generic example). It is easy to verify that *N* is shortcut-free, phylogenetic, level-*k* but not level-$(k-1)$. In all cases, $\mathscr {C}_N$ just consist of the clusters $\{x_1,\dots ,x_k\}$, *X*, and the singletons $\{x\}$, $x\in X,$ are, therefore, a hierarchy. $\square$

As we shall see in Lemma 45 there is no phylogenetic shortcut-free level-1 network *N* (that is not a tree) for which $\mathscr {C}_N$ is a hierarchy.

For a clustering system $\mathscr {C}$ on *X* and a subset $A\subseteq 2^X$, we define the *closure operator* as the map ${{\,\textrm{cl}\,}}:2^X\rightarrow 2^X$ defined by

2 $$\begin{aligned} {{\,\textrm{cl}\,}}(A){:}{=}\bigcap _{\begin{array}{c} C\in \mathscr {C}\\ A \subseteq C \end{array}} C. \end{aligned}$$

It is well defined, isotonic [$A\subseteq B \implies {{\,\textrm{cl}\,}}(A)\subseteq {{\,\textrm{cl}\,}}(B)$], enlarging [$A\subseteq {{\,\textrm{cl}\,}}(A)$], and idempotent ${{\,\textrm{cl}\,}}({{\,\textrm{cl}\,}}(A))={{\,\textrm{cl}\,}}(A)$. For $\vert X\vert >1$, we have ${{\,\textrm{cl}\,}}(\emptyset )=\emptyset$.

#### Definition 10

A clustering system $\mathscr {C}$ is *closed* if, for all *non-empty* $A\in 2^X$, the following condition holds: ${{\,\textrm{cl}\,}}(A)=A \iff A\in \mathscr {C}$.

The following result is well known in the clustering literature.

#### Lemma 16

A clustering system $\mathscr {C}$ is closed if and only if $A,B \in \mathscr {C}$ and $A\cap B\ne \emptyset$ implies $A\cap B\in \mathscr {C}$.

#### Proof

For completeness, a proof is provided in “Closed clustering systems” section in “Appendix.” $\square$

We continue with three simple observations concerning the clusters of networks.

#### Lemma 17

Let *N* be a network. Then, $v\preceq _N w$ implies ${{\,\mathrm{\texttt{C}}\,}}(v)\subseteq {{\,\mathrm{\texttt{C}}\,}}(w)$.

#### Proof

By construction, $x\in {{\,\mathrm{\texttt{C}}\,}}(v)$ if and only if $x\in X$ and *v* lies on a directed path from the root $\rho _N$ to *x*. Furthermore, $v\preceq _N w$ implies that *w* lies on a directed path from $\rho _N$ to *v*. By (N1) and since *N* is a DAG, there is directed path from $\rho _N$ to *x* that contains *w*, and thus $x\preceq _N w$, i.e., $x\in {{\,\mathrm{\texttt{C}}\,}}(w)$. $\square$

We note in passing that the converse of Lemma 17 is not always satisfied (even in level-1 networks): If *v* is a hybrid vertex with unique child *w*, we have ${{\,\mathrm{\texttt{C}}\,}}(v) = {{\,\mathrm{\texttt{C}}\,}}(w)$ and thus ${{\,\mathrm{\texttt{C}}\,}}(v)\subseteq {{\,\mathrm{\texttt{C}}\,}}(w)$, but $v\not \preceq _N w$, (cf. the network *N* in Fig. 4). A result similar to Lemma 6 ensures the existence of a path *P* connecting $\preceq _N$-incomparable vertices $u,v\in V(N)$ that contains only vertices that are below *u* or *v*. However, it requires that *u* and *v* have at least one descendant leaf in common, i.e., that ${{\,\mathrm{\texttt{C}}\,}}(u)\cap {{\,\mathrm{\texttt{C}}\,}}(v)\ne \emptyset$:

#### Lemma 18

Let *N* be a network and $u,v\in V(N)$ be two $\preceq _N$-incomparable vertices such that ${{\,\mathrm{\texttt{C}}\,}}(u)\cap {{\,\mathrm{\texttt{C}}\,}}(v)\ne \emptyset$. Then, for every $x\in {{\,\mathrm{\texttt{C}}\,}}(u)\cap {{\,\mathrm{\texttt{C}}\,}}(v)$, *u* and *v* are connected by an undirected path $P=(w_1{:}{=}u, \dots , w_h,\dots , w_k{:}{=}v)$, $1<h<k$, such that

(i) $(w_i, w_{i+1})\in E(N)$ for all $1\le i< h$, $(w_{i+1}, w_i)\in E(N)$ for all $h\le i< k$, and $w_h$ is a hybrid vertex satisfying $w_h\prec _N u$ and $w_h\prec _N v$.
(ii) $$x\in {{\,\mathrm{\texttt{C}}\,}}(w_h)$$,

In particular, $k\ge 3$, all inner vertices $w_i$ of *P* satisfy $w_i\prec _N u$ or $w_i\prec _N v$, and *P* is a subgraph of a non-trivial block.

#### Proof

There are directed paths $P_u$ and $P_v$ from *u* and *v*, respectively, to the leaf *x*. Let $w^*$ be the $\preceq _N$-maximal vertex of $P_u$ that is also a vertex of $P_v$, which exists since at least *x* is contained in both paths. It must hold that $w^*\notin \{u,v\}$ since otherwise *u* and *v* would be $\preceq _N$-comparable. In particular, $w^*\prec _N u$ and $w^*\prec _N v$. Let $P'_u$ and $P'_v$ be the subpaths of $P_u$ and $P_v$ from *u* and *v*, respectively, to $w^*$. By construction, $w^*$ must be a hybrid vertex, $x\in {{\,\mathrm{\texttt{C}}\,}}(w^*)$, and $P'_u$ and $P'_v$ only have vertex $w^*$ in common, which moreover is an outer vertex of both paths. Now, consider the path $P=(w_1{:}{=}u, \dots , w_h{:}{=}w^*,\dots , w_k{:}{=}v)$, that is the union of the underlying undirected version of $P'_u$ and $P'_v$. It is now easy to verify that *P* satisfies all of the desired properties. Two see that *P* is a subgraph of a non-trivial block *B*, observe that, by Lemma 6, the two $\preceq _N$-incomparable vertices *u* and *v* are connected by an undirected path ${\mathop {P}^{\frown }}$ that contains at least 3 vertices and of which all inner vertices $w'$ satisfy $w'\not \preceq _N u$ and $w'\not \preceq _N v$. Hence, *P* and ${\mathop {P}^{\frown }}$ cannot have any inner vertices in common. Therefore, *u* and *v* are connected by two distinct paths that both have at least 3 vertices and that only have the endpoints *u* and *v* in common. Hence, *u* and *v* lie on a cycle *K* and thus in a common block *B* of *N*. In particular, *P* is a subgraph of *K* and thus of *B*. $\square$

#### Lemma 19

Let *N* be a network and $u, v\in V(N)$. If ${{\,\mathrm{\texttt{C}}\,}}_N(u)$ and ${{\,\mathrm{\texttt{C}}\,}}_N(v)$ overlap, then *u* and *v* are $\preceq _{N}$-incomparable and $u,v\in B^0$ for a non-trivial block *B* of *N*.

#### Proof

Let $u, v\in V(N)$ be distinct vertices such that their two clusters ${{\,\mathrm{\texttt{C}}\,}}_N(u)$ and ${{\,\mathrm{\texttt{C}}\,}}_N(v)$ overlap. In this case, Lemma 17 implies that *u* and *v* are $\preceq _{N}$-incomparable. Lemma 18 implies that *u* and *v* are contained in a common non-trivial block *B* of *N*. Since ${{\,\mathrm{\texttt{C}}\,}}(u)\subseteq {{\,\mathrm{\texttt{C}}\,}}(\max B)$ for all $u\in B$, ${{\,\mathrm{\texttt{C}}\,}}(\max B)$ does not overlap any cluster ${{\,\mathrm{\texttt{C}}\,}}(w)$ with $w\in B$. Consequently, $u,v\ne \max B$. Since ${{\,\mathrm{\texttt{C}}\,}}_N(u)\cap {{\,\mathrm{\texttt{C}}\,}}_N(v)\ne \emptyset$, we can apply Lemma 18 and conclude that there is a hybrid vertex $w_h$ with $w_h\prec _N v$ and $w_h\prec _N u$. In particular, Lemma 18 implies that $w_h$ is contained in the block *B*. Thus, neither *u* nor *v* is a terminal vertex. In summary, $u,v\in B^0$. $\square$

Clusters of outdegree 1 vertices *w* are redundant in the sense that every directed path from *w* to one of its descendant leaves necessarily passes through the unique child *v* of *w*. Thus, we have ${{\,\mathrm{\texttt{C}}\,}}(w)\subseteq {{\,\mathrm{\texttt{C}}\,}}(v)$. Moreover, $v\prec _N w$ and Lemma 17 imply ${{\,\mathrm{\texttt{C}}\,}}(v)\subseteq {{\,\mathrm{\texttt{C}}\,}}(w)$, and thus, ${{\,\mathrm{\texttt{C}}\,}}(v)={{\,\mathrm{\texttt{C}}\,}}(w)$. Hence, we have

#### Observation 5

Let *N* be a network. If *v* is the unique child of *w* in *N*, then ${{\,\mathrm{\texttt{C}}\,}}(v)={{\,\mathrm{\texttt{C}}\,}}(w)$.

#### Lemma 20

Let *N* be a network, *B* a block in *N* and $u,v\in V(B)$. Moreover, let *H* be the set of hybrid vertices *h* that are properly contained in *B* and satisfy $h\preceq _{N} u,v$. Then, it holds ${{\,\mathrm{\texttt{C}}\,}}(u)\cap {{\,\mathrm{\texttt{C}}\,}}(v)\in \{{{\,\mathrm{\texttt{C}}\,}}(u),{{\,\mathrm{\texttt{C}}\,}}(v), \bigcup _{h\in H} {{\,\mathrm{\texttt{C}}\,}}(h)\}$.

#### Proof

It suffices to show that ${{\,\mathrm{\texttt{C}}\,}}(u)\cap {{\,\mathrm{\texttt{C}}\,}}(v)\notin \{{{\,\mathrm{\texttt{C}}\,}}(u),{{\,\mathrm{\texttt{C}}\,}}(v)\}$ implies ${{\,\mathrm{\texttt{C}}\,}}(u)\cap {{\,\mathrm{\texttt{C}}\,}}(v)= \bigcup _{h\in H} {{\,\mathrm{\texttt{C}}\,}}(h) {=}{:}C$. Hence, suppose ${{\,\mathrm{\texttt{C}}\,}}(u)\cap {{\,\mathrm{\texttt{C}}\,}}(v)\notin \{{{\,\mathrm{\texttt{C}}\,}}(u),{{\,\mathrm{\texttt{C}}\,}}(v)\}$. Then, Lemma 17 implies that *u* and *v* are $\preceq _{N}$-incomparable. If $x\in C$, then $x\in {{\,\mathrm{\texttt{C}}\,}}(h)$ for some $h\in H$. Since $h\preceq _{N} u,v$, Lemma 17 implies ${{\,\mathrm{\texttt{C}}\,}}(h)\subseteq {{\,\mathrm{\texttt{C}}\,}}(u)$ and ${{\,\mathrm{\texttt{C}}\,}}(h)\subseteq {{\,\mathrm{\texttt{C}}\,}}(v)$ and thus, $x\in {{\,\mathrm{\texttt{C}}\,}}(h)\subseteq {{\,\mathrm{\texttt{C}}\,}}(u)\cap {{\,\mathrm{\texttt{C}}\,}}(v)$. Now, suppose $x\in {{\,\mathrm{\texttt{C}}\,}}(u)\cap {{\,\mathrm{\texttt{C}}\,}}(v)$. By Lemma 18, the $\preceq _{N}$-incomparable vertices *u* and *v* are connected by an undirected path *P* which contains a hybrid vertex $h \prec _N u,v$ with $x\in {{\,\mathrm{\texttt{C}}\,}}(h)$ and is a subgraph of a non-trivial block $B'$ of *N*. Since *B* and $B'$ share the two distinct vertices *u* and *v*, Observation 1 implies $B=B'$. In particular, $u,v,h\in V(B)$ and $h \prec _N u,v$ imply $h\ne \max B$, and thus, *h* must be properly contained in *B* by Lemma 10. Hence, we have $h\in H$ and thus $x\in {{\,\mathrm{\texttt{C}}\,}}(h) \subseteq C$. In summary, we have $x\in {{\,\mathrm{\texttt{C}}\,}}(u)\cap {{\,\mathrm{\texttt{C}}\,}}(v)$ if and only if $x\in C$, and thus, ${{\,\mathrm{\texttt{C}}\,}}(u)\cap {{\,\mathrm{\texttt{C}}\,}}(v)=C$. $\square$

Note that $H=\emptyset$ and thus $C=\bigcup _{h\in H} {{\,\mathrm{\texttt{C}}\,}}(h)=\emptyset$ in Lemma 20 is possible.

The *Hasse diagram* ${\mathfrak {H}}{:}{=}{\mathfrak {H}}[\mathscr {C}]$ of $\mathscr {C}$ w.r.t. to set inclusion is a DAG whose vertices are the clusters in $\mathscr {C}$. There is a directed arc $(C,C')\in {\mathfrak {H}}$ if $C'\subsetneq C$ and there is no $C''\in \mathscr {C}$ with $C'\subsetneq C''\subsetneq C$. Since $X\in \mathscr {C}$, the Hasse diagram is connected and has *X* as its unique root. The singletons $\{x\}$, $x\in X$, are exactly the inclusion-minimal vertices in $\mathscr {C},$ and thus, they have outdegree 0 but not necessarily indegree 1 in ${\mathfrak {H}}$. Another simple property of ${\mathfrak {H}}$ is the following:

#### Lemma 21

Let $\mathscr {C}$ be a clustering system on *X*. Then, every non-singleton set $C\in \mathscr {C}$ satisfies ${{\,\textrm{outdeg}\,}}_{{\mathfrak {H}}}(C)\ge 2$ in the Hasse diagram ${\mathfrak {H}}$ of $\mathscr {C}$.

#### Proof

Let $C\in \mathscr {C}$ be a non-singleton set, i.e., $\vert C\vert \ge 2$. Therefore, and since $\{x\}\in \mathscr {C}$ for all $x\in X$, there is a directed path in ${\mathfrak {H}}$ from *C* to some singleton set $\{x'\}\in \mathscr {C}$. In particular, this path contains at least the two distinct clusters *C* and $\{x'\}$, and thus, *C* has a child $C'$ in ${\mathfrak {H}}$ with $\{x'\}\subseteq C'\subsetneq C$. Now, pick an element $x''\in C{\setminus } C'\ne \emptyset$. Since $\{x''\}\in \mathscr {C}$ and $\{x''\}\subseteq C$, we can argue similarly as before to conclude that *C* has a child $C''$ in ${\mathfrak {H}}$ with $\{x''\}\subseteq C''\subsetneq C$. Since $x''\notin C'$, we have $C'\ne C''$ and thus, *C* satisfies ${{\,\textrm{outdeg}\,}}_{{\mathfrak {H}}}(C)\ge 2$. $\square$

#### Lemma 22

Let $\mathscr {C}$ be a clustering system on *X* with corresponding Hasse diagram ${\mathfrak {H}}$. Then, ${\mathfrak {H}}$ is a phylogenetic network with leaf set $X_{{\mathfrak {H}}}{:}{=}\{ \{x\} \mid x \in X \}$.

#### Proof

Clearly, ${\mathfrak {H}}$ is a DAG. Since $X\in \mathscr {C}$ and $C\subseteq X$ for all $C\in \mathscr {C}$, *X* is the unique cluster in $\mathscr {C}$ with indegree 0, i.e., *X* is the root in ${\mathfrak {H}}$ and ${\mathfrak {H}}$ satisfies (N1). By definition of clustering systems, we have $X_{{\mathfrak {H}}}\subseteq \mathscr {C}$. Now, consider a cluster $\{x\}\in X_{{\mathfrak {H}}}$. Since $\emptyset \notin \mathscr {C}$, $\{x\}$ has outdegree zero in ${\mathfrak {H}}$. Lemma 21 implies ${{\,\textrm{outdeg}\,}}_{{\mathfrak {H}}}(C)\ge 2$ for all $C\in \mathscr {C}$ with $\vert C\vert >1$, i.e., for all $C\in \mathscr {C}{\setminus } X_{{\mathfrak {H}}}$. Taken together, the latter arguments imply that the elements in $X_{{\mathfrak {H}}}$ are exactly the leaves of ${\mathfrak {H}}$ and that (N2) is satisfied. $\square$

For a given a clustering system $\mathscr {C}$ and a cluster $C\in \mathscr {C}$, we will moreover make use of the subsets

3 $$\begin{aligned} \mathcal {D}(C){:}{=}\{D\in \mathscr {C}\mid D\subsetneq C\} \qquad \text {and} \qquad \overline{\mathcal {D}}(C){:}{=}\{D\in \mathscr {C}\mid D \not \subseteq C\}. \end{aligned}$$

Note that, by definition, we have $\mathcal {D}(C){\cup \!\!\!\cdot }\,\,\overline{\mathcal {D}}(C){\cup \!\!\!\cdot }\,\,\{C\}=\mathscr {C}$ for all $C\in \mathscr {C}$, $\mathcal {D}(C)=\emptyset$ if and only if *C* is a singleton, and $\overline{\mathcal {D}}(C)=\emptyset$ if and only if $C=X$.

#### Lemma 23

Let ${\mathfrak {H}}$ be the Hasse diagram of a clustering system $\mathscr {C}$ and $C\in \mathscr {C}$ such that *C* does not overlap any other set. Then, there is no undirected cycle in ${\mathfrak {H}}$ that intersects both $\mathcal {D}(C)$ and $\overline{\mathcal {D}}(C)$. In particular, if $C\ne X$ and $\vert C\vert >1$, then *C* is a cut vertex in ${\mathfrak {H}}$.

#### Proof

Suppose that *C* does not overlap with any other cluster. If $C=X$ then, $\overline{\mathcal {D}}(C) = \emptyset$ and if $\vert C\vert =1$ then $\mathcal {D}(C) = \emptyset$ and thus, for any cycle *K* in ${\mathfrak {H}}$ we have $K\cap \overline{\mathcal {D}}(C) = \emptyset$ or $K\cap {\mathcal {D}}(C) = \emptyset$. Hence, *K* cannot intersect both. Now, assume that $C\ne X$ and $\vert C\vert >1$. Since *C* is neither a singleton nor *X*, both $\mathcal {D}(C)$ and $\overline{\mathcal {D}}(C)$ are non-empty. Furthermore, $\mathcal {D}(C)\cup \overline{\mathcal {D}}(C)= \mathscr {C}{\setminus }\{C\}$. Let $C_1\in \mathcal {D}(C)$ and $C_2\in \overline{\mathcal {D}}(C)$. By assumption, we have $C_1\subsetneq C$ and either (i) $C_2\cap C=\emptyset$ or (ii) $C\subsetneq C_2$. In case (i), we have $C_1\cap C_2=\emptyset$ and in case (ii), it holds $C_1\subsetneq C\subsetneq C_2$. Therefore, and since $C_1\in \mathcal {D}(C)$ and $C_2\in \overline{\mathcal {D}}(C)$ were chosen arbitrarily, ${\mathfrak {H}}$ contains no arc connecting a cluster in $\mathcal {D}(C)$ and a cluster in $\overline{\mathcal {D}}(C)$. Together with $\mathcal {D}(C)\cup \overline{\mathcal {D}}(C)=\mathscr {C}\setminus \{C\}$, this implies that the subgraph of ${\mathfrak {H}}$ obtained by removing *C* is disconnected and thus *C* is a cut vertex. In particular, every undirected path connecting a cluster in $\mathcal {D}(C)$ and a cluster in $\overline{\mathcal {D}}(C)$ has to pass through *C* and thus the second statement of the lemma follows as an immediate consequence. $\square$

**Fig. 7:**
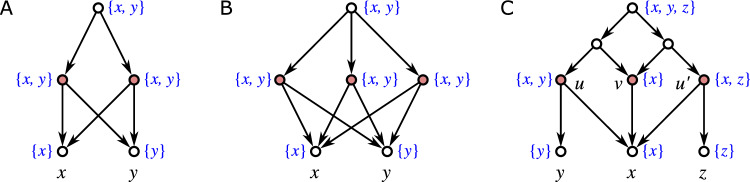
Both the rooted $K_{2,3}$ (**A**) and $K_{3,3}$ (**B**) are a phylogenetic networks that have only two leaves, denoted *x* and *y* here. The clustering system therefore consists only of $X=\{x,y\}$ and the two singletons $\{x\}$ and $\{y\}$. The same clustering system can be represented by a rooted tree with a single root that is adjacent to the two leaves *x* and *y*. In particular, both networks do not satisfy (PCC) since the highlighted vertices are $\preceq _{N}$-incomparable but share the cluster $\{x,y\}$. **C** A network showing that ${{\,\mathrm{\texttt{C}}\,}}(v)\subsetneq {{\,\mathrm{\texttt{C}}\,}}(u)$ is also possible for two $\preceq$-incomparable vertices *u* and *v*

Every phylogenetic tree *T* is isomorphic to the Hasse diagram of its clustering systems $\mathscr {C}$ by virtue of the map $\varphi :V(T)\rightarrow \mathscr {C},\, v\mapsto {{\,\mathrm{\texttt{C}}\,}}(v)$, see, e.g., Semple and Steel (2003). Figure 7 shows that this is not the case for phylogenetic networks in general. The rooted networks that share this property with phylogenetic trees have been introduced and studied in Baroni et al. (2004); Baroni and Steel (2006); Willson (2010).

#### Definition 11

(Baroni et al. 2004) A network $N=(V,E)$ is *regular* if the map $\varphi :V\rightarrow V({\mathfrak {H}}[\mathscr {C}_N]):v\mapsto {{\,\mathrm{\texttt{C}}\,}}(v)$ is a graph isomorphism between *N* and ${\mathfrak {H}}[\mathscr {C}_N]$.

The graph isomorphism in Definition 11 is quite constrained. In particular, it is not obvious that an arbitrary graph isomorphism $\varphi$ between the two networks *N* and ${\mathfrak {H}}[\mathscr {C}_N]$ implies that *N* is regular, as $\varphi (v)\ne {{\,\mathrm{\texttt{C}}\,}}(v)$ may be possible. As we shall see in Corollary 11, however, $N\sim {\mathfrak {H}}[\mathscr {C}_N]$ if and only if *N* is regular. Even more, as noted without proof in Baroni et al. (2004), a rooted network *N* with leaf set *X* is regular if and only if it is graph isomorphic to the Hasse diagram ${\mathfrak {H}}[\mathscr {C}]$ for some clustering system $\mathscr {C}\subseteq 2^X$. This result will be an immediate consequence of the results established below and will be summarized and proven in Proposition 4.

#### Proposition 2

For every clustering system $\mathscr {C}$, there is a unique regular network *N* with $\mathscr {C}_N=\mathscr {C}$.

#### Proof

Let $\mathscr {C}$ be a clustering system on *X*. By Lemma 22, ${\mathfrak {H}}[\mathscr {C}]$ is a network with leaf set $X_{{\mathfrak {H}}}{:}{=}\{ \{x\} \mid x \in X \}$. Replacing all leaves $\{x\}$ in ${\mathfrak {H}}[\mathscr {C}]$ with the single element *x* that they contain clearly yields a network *N* such that $\mathscr {C}_{N}=\mathscr {C}$ and $\varphi :V(N)\rightarrow V({\mathfrak {H}}[\mathscr {C}]):v\mapsto {{\,\mathrm{\texttt{C}}\,}}_{N}(v)$ is an isomorphism between *N* and ${\mathfrak {H}}[\mathscr {C}]$. By definition, *N* is regular.

Now, let $N'$ be a regular network with $\mathscr {C}_{N'}=\mathscr {C}$, i.e., there is an isomorphism $\varphi ':V(N')\rightarrow V({\mathfrak {H}}[\mathscr {C}]):v\mapsto {{\,\mathrm{\texttt{C}}\,}}_{N'}(v)$ between $N'$ and ${\mathfrak {H}}[\mathscr {C}]$. In particular, we have $\varphi (x)=\varphi '(x)=\{x\}$ for all $x\in X$ and thus $\varphi '^{-1}(\varphi (x))=x$. Hence, $\varphi '^{-1} \circ \varphi$ is an isomorphism between *N* and *N* that is the identity on *X*. Hence, *N* is the unique regular network with $\mathscr {C}_N=\mathscr {C}$. $\square$

#### Remark 1

By a slight abuse of notation, we also write ${\mathfrak {H}}[\mathscr {C}]$ for the unique regular network of a clustering system $\mathscr {C}$ since it is obtained from the Hasse diagram by relabeling all leaves $\{x\}$ with *x*.

The following characterization is a slight rephrasing of Proposition 4.1 in Baroni et al. (2004):

#### Proposition 3

A network *N* is regular if and only if

(i) ${{\,\mathrm{\texttt{C}}\,}}(u)\subseteq {{\,\mathrm{\texttt{C}}\,}}(v) \iff u\preceq _N v$ for all $u,v\in V$, and
(ii) *N* is shortcut-free.

#### Proof

Proposition 4.1 in Baroni et al. (2004) states that *N* is regular if and only if the following three conditions hold: (a) $u\ne v$ implies ${{\,\mathrm{\texttt{C}}\,}}(u)\ne {{\,\mathrm{\texttt{C}}\,}}(v)$, i.e., ${{\,\mathrm{\texttt{C}}\,}}(u)={{\,\mathrm{\texttt{C}}\,}}(v)\implies u=v$; (b) if ${{\,\mathrm{\texttt{C}}\,}}(u)\subsetneq {{\,\mathrm{\texttt{C}}\,}}(v)$, then there is a directed path from *v* to *u*, i.e., $u\prec _N v$; and (c) if there are two distinct directed paths connecting *u* and *v*, then neither path consists of a single arc, i.e., (*u*, *v*) is not a shortcut. Clearly, conditions (ii) and (c) are equivalent. It therefore suffices to show that condition (i) holds if and only if conditions (a) and (b) are satisfied. Together, (a), (b) and Lemma 17 obviously imply (i). Now, suppose (i) is satisfied. Then, ${{\,\mathrm{\texttt{C}}\,}}(u)={{\,\mathrm{\texttt{C}}\,}}(v)$ implies ${{\,\mathrm{\texttt{C}}\,}}(u)\subseteq {{\,\mathrm{\texttt{C}}\,}}(v)$ and ${{\,\mathrm{\texttt{C}}\,}}(v)\subseteq {{\,\mathrm{\texttt{C}}\,}}(u)$ and thus we have both $u\preceq _N v$ and $v\preceq _N u$, and hence $u=v$, i.e., (a) holds. Assuming ${{\,\mathrm{\texttt{C}}\,}}(u)\subsetneq {{\,\mathrm{\texttt{C}}\,}}(v)$, i.e., ${{\,\mathrm{\texttt{C}}\,}}(u)\subseteq {{\,\mathrm{\texttt{C}}\,}}(v)$ and ${{\,\mathrm{\texttt{C}}\,}}(u)\ne {{\,\mathrm{\texttt{C}}\,}}(v)$ implies $u\preceq _N v$ by (i) and $u\ne v$ by (a), and thus, $u\prec _N v$, i.e., (b) holds as well. $\square$

Proposition 2 and 3 imply

#### Corollary 7

For every clustering system $\mathscr {C}$ there is a network *N* with $\mathscr {C}_N = \mathscr {C}$ such that ${{\,\mathrm{\texttt{C}}\,}}(u)\subseteq {{\,\mathrm{\texttt{C}}\,}}(v) \iff u\preceq _N v$ for all $u,v\in V$.

#### Corollary 8

Every regular network is least-resolved.

#### Proof

Suppose, for contradiction, that the regular network *N* is not least-resolved, i.e., there is a network $N'$ with $\mathscr {C}_N=\mathscr {C}_{N'}$ that can be obtained from *N* by a non-empty sequence of shortcut removals and application of ${{\,\mathrm{\textsc {cntr}}\,}}(v',v)$. By Proposition 3, *N* is shortcut-free and, therefore, the operation that is applied first must be a contraction. Therefore, and since no new vertices are introduced, it must hold $\vert V(N')\vert <\vert V(N)\vert$. Since *N* is regular, it holds $\vert V(N)\vert =\vert \mathscr {C}_N\vert$. Hence, we have $\vert V(N')\vert <\vert \mathscr {C}_N\vert =\vert \mathscr {C}_{N'} \vert$, a contradiction. $\square$

**Fig. 8:**
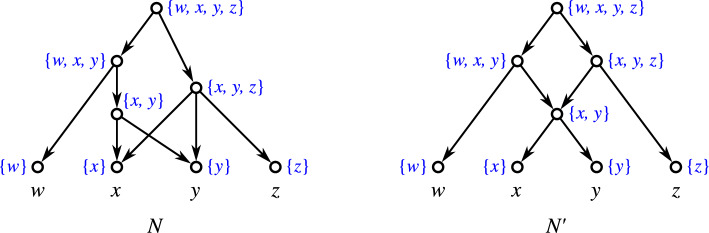
The network *N* is least-resolved but not isomorphic to $N'\sim {\mathfrak {H}}[\mathscr {C}_N]$. Consequently, *N* is not regular

The converse of Corollary 8, however, is not satisfied, see Fig. 8. The network *N* is shortcut-free and satisfies $\vert V(N) \vert = \vert \mathscr {C}_N\vert$. Hence, $\mathscr {C}_N$ is least-resolved. The unique regular network $N'$ for $\mathscr {C}_N$ on the r.h.s. of Fig. 8 is not isomorphic to *N*. Hence, we obtain

#### Observation 6

Not every least-resolved network is regular.

Nevertheless, for level-1 networks the terms least-resolved and regular coincide as shown in Corollary 23.

A clustering system $\mathscr {C}$, by definition, is a hierarchy if and only if, for all $C,C'\in \mathscr {C}$ holds $C\cap C'\in \{\emptyset ,C,C'\}$.

#### Corollary 9

A clustering system $\mathscr {C}$ on *X* is a hierarchy if and only if ${\mathfrak {H}}[\mathscr {C}]$ is a phylogenetic tree. Moreover, *N* is a phylogenetic tree if and only if ${\mathfrak {H}}[\mathscr {C}_N]\sim N$ and $\mathscr {C}_N$ is a hierarchy.

#### Proof

The 1-to-1 correspondence of hierarchies and phylogenetic trees is well known, see, e.g., (Theorem 3.5.2 Semple and Steel 2003). $\square$

## Semi-regular networks

### Path-cluster comparability

Regularity as characterized in Proposition 3 is a bit too restrictive for our purposes. We therefore consider a slightly weaker condition, which we will call *semi-regularity*. More precisely, we relax condition (i) in Proposition 3:

#### Definition 12

A network *N* has the *path-cluster-comparability (PCC)* property if it satisfies, for all $u,v\in V(N)$,

(PCC): *u* and *v* are $\preceq _N$-comparable if and only if ${{\,\mathrm{\texttt{C}}\,}}(u)\subseteq {{\,\mathrm{\texttt{C}}\,}}(v)$ or ${{\,\mathrm{\texttt{C}}\,}}(v)\subseteq {{\,\mathrm{\texttt{C}}\,}}(u)$.

Definition 12 together with Corollary 7 implies

#### Lemma 24

For every clustering system $\mathscr {C}$ there is a network *N* with $\mathscr {C}_N = \mathscr {C}$ that satisfies (PCC).

We emphasize that (PCC) is a quite restrictive property. For instance, the rooted $K_{3,3}$ in Fig. 7B violates (PCC). The example in Fig. 7C shows that even ${{\,\mathrm{\texttt{C}}\,}}(v)\subsetneq {{\,\mathrm{\texttt{C}}\,}}(u)$ is possible for two $\preceq _{N}$-incomparable vertices *u* and *v*.

#### Observation 7

Let *N* be a network satisfying (PCC) and $u, v\in V(N)$. Then, ${{\,\mathrm{\texttt{C}}\,}}(u)\subsetneq {{\,\mathrm{\texttt{C}}\,}}(v)$ implies $u\prec _N v$.

#### Proof

Suppose ${{\,\mathrm{\texttt{C}}\,}}(u)\subsetneq {{\,\mathrm{\texttt{C}}\,}}(v)$. Then, (PCC) implies that *u* and *v* are $\preceq _{N}$-comparable. If $v\preceq _N u$, then Lemma 17 implies ${{\,\mathrm{\texttt{C}}\,}}(v)\subseteq {{\,\mathrm{\texttt{C}}\,}}(u)$, a contradiction. Hence, only $u\prec _N v$ is possible. $\square$

#### Lemma 25

Let *N* be a network. Then, ${{\,\mathrm{\texttt{C}}\,}}(u)={{\,\mathrm{\texttt{C}}\,}}(v)$ and $u\prec _N v$ imply that there is $w\in {{\,\textrm{child}\,}}_N(v)$ such that $u\preceq _N w \prec _N v$ and ${{\,\mathrm{\texttt{C}}\,}}(w)={{\,\mathrm{\texttt{C}}\,}}(v)$.

#### Proof

Since $u\prec _N v$, there is a *vw*-path which passes through some child $w\in {{\,\textrm{child}\,}}_N(v)$. Hence, we have $u\preceq _N w \prec _N v$. Lemma 17 implies ${{\,\mathrm{\texttt{C}}\,}}(u)\subseteq {{\,\mathrm{\texttt{C}}\,}}(w)\subseteq {{\,\mathrm{\texttt{C}}\,}}(v)$. Together with ${{\,\mathrm{\texttt{C}}\,}}(u)={{\,\mathrm{\texttt{C}}\,}}(v)$, this yields ${{\,\mathrm{\texttt{C}}\,}}(v)={{\,\mathrm{\texttt{C}}\,}}(w)={{\,\mathrm{\texttt{C}}\,}}(u)$. $\square$

Property (PCC) still allows that distinct vertices are associated with the same clusters. It requires, however, that such vertices lie along a common directed path.

#### Definition 13

A network is *semi-regular* if it is shortcut-free and satisfies (PCC).

We introduce the term “semi-regular” because, as we shall see in Theorem 2, it is a moderate generalization of regularity that preserves many of useful properties of regular networks.

#### Lemma 26

Let *N* be a semi-regular network and let $v\in V(N)$. Then, there is a vertex $u\in V(N)$ with ${{\,\mathrm{\texttt{C}}\,}}(u)={{\,\mathrm{\texttt{C}}\,}}(v)$ and $u\prec _N v$ if and only if ${{\,\textrm{outdeg}\,}}(v)=1$. If, moreover, *N* is phylogenetic, then *u* is the unique child of *v* in this case.

#### Proof

Suppose first that ${{\,\textrm{outdeg}\,}}(v)=1$ and thus let *u* be the unique child of *v*. Thus, it holds $u\prec _N v$ and, by Observation 5, we have ${{\,\mathrm{\texttt{C}}\,}}(u)={{\,\mathrm{\texttt{C}}\,}}(v)$. Conversely, suppose that ${{\,\mathrm{\texttt{C}}\,}}(u)={{\,\mathrm{\texttt{C}}\,}}(v)$ and $u\prec _N v$. Lemma 25 implies that there is $w\in {{\,\textrm{child}\,}}(v)$ with $u\preceq _N w \prec _N v$ and ${{\,\mathrm{\texttt{C}}\,}}(w)={{\,\mathrm{\texttt{C}}\,}}(v)$. Suppose there is another child $w'\in {{\,\textrm{child}\,}}(v)$ with $w'\ne w$. By Lemma 17, we have ${{\,\mathrm{\texttt{C}}\,}}(w')\subseteq {{\,\mathrm{\texttt{C}}\,}}(v)={{\,\mathrm{\texttt{C}}\,}}(w)$. Hence, (PCC) implies that *w* and $w'$ are $\preceq _{N}$-comparable. But then Observation 3 and *N* being shortcut-free imply $w=w'$, a contradiction. Hence, *w* is the unique child of *v*.

Now, suppose, in addition, that *N* is phylogenetic and assume, for contradiction, that $u\ne w$ and thus $u\prec _N w$. We can apply similar arguments as before to conclude that *w* has a unique child. Therefore, and since *N* is phylogenetic, there must be a vertex $v'\in {{\,\textrm{par}\,}}_N(w)\setminus \{v\}$ since otherwise ${{\,\textrm{indeg}\,}}(w)={{\,\textrm{outdeg}\,}}(w)=1$. By Lemma 17, we have ${{\,\mathrm{\texttt{C}}\,}}(v)={{\,\mathrm{\texttt{C}}\,}}(w)\subseteq {{\,\mathrm{\texttt{C}}\,}}(v')$. This together with (PCC) implies that *v* and $v'$ are $\preceq _{N}$-comparable. But then Observation 3 and *N* being shortcut-free imply $v=v'$, a contradiction. Therefore, $u=w$ is the unique child of *v*. $\square$

As a consequence of Lemmas 17 and 26, we have

#### Corollary 10

Let *N* be a semi-regular network and let $v\in V^0$. Then, ${{\,\mathrm{\texttt{C}}\,}}(u)\subsetneq {{\,\mathrm{\texttt{C}}\,}}(v)$ for all $u\in {{\,\textrm{child}\,}}_N(v)$ if and only if ${{\,\textrm{outdeg}\,}}(v)\ge 2$.

#### Lemma 27

Let *N* be a semi-regular network, $u\in V(N)$, and let $Q(u){:}{=}\{u'\in V(N) \mid {{\,\mathrm{\texttt{C}}\,}}(u')={{\,\mathrm{\texttt{C}}\,}}(u)\}$. Then, the vertices of *Q*(*u*) are pairwise $\preceq _N$-comparable and lie consecutively along an induced directed path in *N*. Moreover, if $\vert Q(u)\vert >1$, either *Q*(*u*) is contained in a non-trivial block, or adjacent pairs of vertices in *Q*(*u*) form a trivial block.

#### Proof

By (PCC), ${{\,\mathrm{\texttt{C}}\,}}(v)={{\,\mathrm{\texttt{C}}\,}}(w)$ and $v\ne w$ imply $v\prec _N w$ or $w\prec _N v$ for $v,w\in Q(u)$, i.e., the vertices in *Q*(*u*) are pairwise $\preceq _N$-comparable and thus linearly ordered w.r.t. $\preceq _N$. Using Lemma 25, one easily verifies that *Q*(*u*) forms a directed path in *N* and there are unique $\preceq _{N}$-minimal and $\preceq _{N}$-maximal vertices $\min Q(u)$ and $\max Q(u)$. In particular, since *N* is acyclic and shortcut-free, this path must be an induced subgraph.

Now, suppose *u* is a hybrid vertex and suppose there is $v\in Q(u)$ with $(v,u)\in E(N)$, i.e., *u* has a parent in *Q*(*u*). Then, there is $v'\in {{\,\textrm{par}\,}}(u)\setminus \{v\}$. By Lemma 17, ${{\,\mathrm{\texttt{C}}\,}}(u)\subseteq {{\,\mathrm{\texttt{C}}\,}}(v')$, and thus, by (PCC), *v* and $v'$ are $\preceq _N$-comparable. However, since *N* is shortcut-free, *v* and $v'$ are $\preceq _N$-incomparable by Observation 3, a contradiction. Thus, only $\max Q(u)$ can be a hybrid vertex in *Q*(*u*). By similar argument, only $\min Q(u)$ can have outdegree greater that one. Hence, all inner vertices in the directed path *P* formed by *Q*(*u*) have degree 2. Therefore, if one arc in *P* is contained in an undirected cycle, then all arcs in *P* are contained in this cycle, in which case *Q*(*u*) is contained in a non-trivial block. Otherwise *Q*(*u*) consists of a sequence of consecutive cut-arcs. $\square$

The examples in Fig. 7A, B show that (PCC) is necessary in Lemma 27. We note, moreover, that the cardinality $\vert Q(u)\vert$ equals the multiplicity of the cluster ${{\,\mathrm{\texttt{C}}\,}}(u)$ in $\mathscr {M}_N$.

#### Theorem 2

A network is regular if and only if it is semi-regular and there is no vertex with outdegree 1.

#### Proof

If *N* is regular, it is in particular also semi-regular and thus satisfies (PCC). Furthermore, then ${{\,\mathrm{\texttt{C}}\,}}(u)={{\,\mathrm{\texttt{C}}\,}}(v)$ implies $u=v$ and thus there are no two vertices *u*, *v* satisfying ${{\,\mathrm{\texttt{C}}\,}}(u)={{\,\mathrm{\texttt{C}}\,}}(v)$ and $u\prec _N v$. Lemma 26 thus implies that there is no vertex with outdegree 1. Conversely, assume that *N* is semi-regular (and thus shortcut-free) and suppose there is not vertex with outdegree 1. Then, Lemma 26 implies that there is no pair of vertices *u*, *v* with ${{\,\mathrm{\texttt{C}}\,}}(u)={{\,\mathrm{\texttt{C}}\,}}(v)$ and $u\prec _N v$ or $v\prec _N u$, i.e., ${{\,\mathrm{\texttt{C}}\,}}(u)={{\,\mathrm{\texttt{C}}\,}}(v)$ implies $u=v$. Therefore and by Lemma 17, we have ${{\,\mathrm{\texttt{C}}\,}}(u)\subseteq {{\,\mathrm{\texttt{C}}\,}}(v) \iff u\preceq _N v$. By Proposition 3, therefore, *N* is regular. $\square$

#### Proposition 4

Let *N* be a network on *X*. Then, $N\sim {\mathfrak {H}}[\mathscr {C}]$ for some clustering system $\mathscr {C}\subseteq 2^X$ if and only if *N* is regular.

#### Proof

Assume first that $N\sim {\mathfrak {H}}[\mathscr {C}]$. In this case, *N* is shortcut-free, satisfies (PCC), and has no vertex with outdegree 1 (since ${\mathfrak {H}}[\mathscr {C}]$ has these properties). By Theorem 2, *N* is regular. Assume now that *N* is regular. By Definition 11, $N\sim {\mathfrak {H}}[\mathscr {C}_N]$. Thus, we can put $\mathscr {C} = \mathscr {C}_N$ and obtain $N\sim {\mathfrak {H}}[\mathscr {C}]$. $\square$

It should be noted that $N\sim {\mathfrak {H}}[\mathscr {C}]$ does not necessarily imply that $\mathscr {C}_N = \mathscr {C}$. By way of example, consider a binary phylogenetic rooted tree *T* on *X* with $\mathscr {C}_T = \{\{1\}, \{2\}, \{3\}, \{1,2\}, \{1,2,3\}\}$ and the clustering system $\mathscr {C} = \{\{1\}, \{2\}, \{3\}, \{2,3\}, \{1,2,3\}\}$. It can easily be verified that $T\sim {\mathfrak {H}}[\mathscr {C}]$ although $\mathscr {C}_T\ne \mathscr {C}$. Nevertheless, Proposition 4 together with Definition 11 immediately implies

#### Corollary 11

$N\sim {\mathfrak {H}}[\mathscr {C}_N]$ if and only if *N* is regular.

A crucial link between a network and its clustering systems is the ability to identify the non-trivial blocks. The following result shows that, at least in semi-regular networks, key information is provided by the overlaps of clusters.

#### Lemma 28

Let *B* be a non-trivial block in a semi-regular network *N*. Then for every $u\in B^0$ there is a $v\in B^0$ such that ${{\,\mathrm{\texttt{C}}\,}}(u)$ and ${{\,\mathrm{\texttt{C}}\,}}(v)$ overlap.

#### Proof

Suppose $u\in B^0$ and consider the two disjoint sets $A{:}{=}\{w\in V(B) \mid u\prec _{N} w\}$ and $D{:}{=}\{w\in V(B) \mid w\prec _{N} u\}$, i.e., the ancestors and descendants, resp., of *u* in *B*. Note that both sets are non-empty since $u\in B^0$. There is no arc connecting a vertex in *A* with a vertex in *D*. To see this, consider $a\in A$ and $d\in D$. Since $d\prec _{N} u \prec _{N} a$ and *N* is acyclic, there is a directed path from *a* to *d* passing through *u*. Thus, an arc (*a*, *d*) would be a shortcut, contradicting that *N* is semi-regular, and an arc (*d*, *a*) would imply $a\prec _{N} d$ contradicting $d\prec _{N} u \prec _{N} a$. Thus, an arc connecting a vertex in *A* with a vertex in *D* cannot exist. Since $a,d\in V(B)$ and *B* is a non-trivial block, *a* and *d* lie on an undirected cycle *K* in *B*. In particular, they are connected by two undirected paths that do not share any inner vertices. Thus, there is an undirected path $P=(d=v_1, v_2,\dots ,a=v_k)$ that does not contain *u*. Let $v_i$ be the unique vertex in *P* such that $v_i\in D$ and there is no vertex $v_j\in D$ with $i<j\le k$. Such a vertex exists since $v_1\in D$. Moreover, $v_k=a\in A$ implies $i< k$. Thus, consider the vertex $v{:}{=}v_{i+1}$. We have $v\notin D$ by construction and $v\notin A$ since $v_i\in D$ is not adjacent to any vertex in *A*. Since *P* does not contain *u* and $v\in V(B){\setminus } (A{\cup \!\!\!\cdot }\,\,D)$, we see that *u* and *v* are $\preceq _{N}$-incomparable. Since $v_i\in D$, we have $v_i\prec _N u$. Hence, $(v_i,v_{i+1})$ cannot be an arc in *N* since otherwise $v_{i+1}\prec _{N} v_i\prec _N u$ would imply $v_{i+1}\in D$, a contradiction. Therefore, it have $(v, v_i)=(v_{i+1}, v_i)\in E(N)$ and thus $v_i\prec _{N} u,v$. By Lemma 17, this implies $\emptyset \ne {{\,\mathrm{\texttt{C}}\,}}_{N}(v_i)\subseteq {{\,\mathrm{\texttt{C}}\,}}_{N}(u)\cap {{\,\mathrm{\texttt{C}}\,}}_{N}(v)$. Together with (PCC) and the fact that *u* and *v* are $\preceq _{N}$-incomparable, this yields that ${{\,\mathrm{\texttt{C}}\,}}(u)$ and ${{\,\mathrm{\texttt{C}}\,}}(v)$ overlap. In particular, $v\ne \max B$ since *u* and *v* are $\preceq _{N}$-incomparable and *v* is not a terminal vertex since $v_{i}\prec _{N} v$. Therefore, we have $v\in B^0$. $\square$

We note that semi-regularity cannot be omitted in Lemma 28, since the statement is not true for the rooted $K_{3,3}$ of Fig. 7. Lemma 19 and Lemma 28 together show that in semi-regular networks all vertices in the interior of non-trivial blocks are identified by the fact that their clusters overlap. It remains an open question, however, whether the information of cluster overlaps is sufficient to identify the non-trivial blocks.

We continue by showing that whenever (PCC) is satisfied, least-resolved networks are precisely the regular network, To this end, we consider first the implications given by the operations ${{\,\mathrm{\textsc {expd}}\,}}$ and ${{\,\mathrm{\textsc {cntr}}\,}}$, and by the removal of shortcuts, respectively.

#### Lemma 29

Let *N* be a network, $w\in V(N)$, and $N'$ the network obtained from *N* by applying ${{\,\mathrm{\textsc {expd}}\,}}(w)$. Then, *N* satisfies (PCC) if and only if $N'$ satisfies (PCC).

#### Proof

By Lemma 5, *N* and $N'$ are $(N',N)$-ancestor-preserving, i.e., $v\preceq _{N} v'$ if and only if $v\preceq _{N} v'$ holds for all $v,v'\in V(N)$. By Lemma 5, ${{\,\mathrm{\texttt{C}}\,}}_N(v)={{\,\mathrm{\texttt{C}}\,}}_{N'}(v)$ for all $v\in V(N)\subseteq V(N')$. Let $w'$ be the unique vertex in $V(N')\setminus V(N)$. By construction, *w* is the unique child of $w'$ in $N'$ and thus, by Observation 5, ${{\,\mathrm{\texttt{C}}\,}}_{N}(w)={{\,\mathrm{\texttt{C}}\,}}_{N'}(w)={{\,\mathrm{\texttt{C}}\,}}_{N'}(w')$.

Suppose first $N'$ satisfies (PCC), i.e., for all $u, v\in V(N')$, it holds that *u* and *v* are $\preceq _{N'}$-comparable if and only if ${{\,\mathrm{\texttt{C}}\,}}_{N'}(u)\subseteq {{\,\mathrm{\texttt{C}}\,}}_{N'}(v)$ or ${{\,\mathrm{\texttt{C}}\,}}_{N'}(v)\subseteq {{\,\mathrm{\texttt{C}}\,}}_{N'}(u)$. To see that *N* also satisfies (PCC), consider $u, v\in V(N)\subseteq V(N')$. Suppose *u* and *v* are $\preceq _{N}$-comparable. Hence, *u* and *v* are also $\preceq _{N'}$-comparable, and thus, ${{\,\mathrm{\texttt{C}}\,}}_{N}(u)={{\,\mathrm{\texttt{C}}\,}}_{N'}(u)\subseteq {{\,\mathrm{\texttt{C}}\,}}_{N'}(v)={{\,\mathrm{\texttt{C}}\,}}_{N}(v)$ or ${{\,\mathrm{\texttt{C}}\,}}_{N}(v)={{\,\mathrm{\texttt{C}}\,}}(v)_{N'}\subseteq {{\,\mathrm{\texttt{C}}\,}}_{N'}(u)={{\,\mathrm{\texttt{C}}\,}}_{N}(u)$. Conversely, if ${{\,\mathrm{\texttt{C}}\,}}_{N}(u)\subseteq {{\,\mathrm{\texttt{C}}\,}}_{N}(v)$ or ${{\,\mathrm{\texttt{C}}\,}}_{N}(v)\subseteq {{\,\mathrm{\texttt{C}}\,}}_{N}(u)$, then also ${{\,\mathrm{\texttt{C}}\,}}_{N'}(u)\subseteq {{\,\mathrm{\texttt{C}}\,}}_{N'}(v)$ or ${{\,\mathrm{\texttt{C}}\,}}_{N'}(v)\subseteq {{\,\mathrm{\texttt{C}}\,}}_{N'}(u)$. Hence, *u* and *v* are $\preceq _{N'}$-comparable and thus also $\preceq _{N}$-comparable.

Now, suppose *N* satisfies (PCC). By similar argument as above, it holds, for all $u, v\in V(N)=V(N')\setminus \{w'\}$ that *u* and *v* are $\preceq _{N'}$-comparable if and only if ${{\,\mathrm{\texttt{C}}\,}}_{N'}(u)\subseteq {{\,\mathrm{\texttt{C}}\,}}_{N'}(v)$ or ${{\,\mathrm{\texttt{C}}\,}}_{N'}(v)\subseteq {{\,\mathrm{\texttt{C}}\,}}_{N'}(u)$. To show that $N'$ satisfies (PCC), it therefore only remains to consider $w'$ and some vertex $v\in V(N)$. It holds that *v* and $w'$ are $\preceq _{N'}$-comparable if and only if *v* and *w* are $\preceq _{N'}$-comparable. To see this, suppose first *v* and $w'$ are $\preceq _{N'}$-comparable. If $v\prec _{N'} w'$, then $v\preceq _{N'} w$ since *w* is the unique child of $w'$. If $w'\prec _{N'} v$, then $w\prec _{N'} w'$ implies $w\prec _{N'} v$. Now, suppose *v* and *w* are $\preceq _{N'}$-comparable. If $v\preceq _{N'} w$, then $w\prec _{N'} w'$ implies $v\prec _{N'} w'$. If $w\prec _{N'} v$, then $w'\preceq _{N'} v$ (and thus $w'\prec _{N'} v$) since $w'$ is the unique parent of *w* in $N'$. Taken together and since $v,w\in V(N)$, the arguments so far imply that *v* and $w'$ are $\preceq _{N'}$-comparable if and only if *v* and *w* are $\preceq _{N'}$-comparable if and only if ${{\,\mathrm{\texttt{C}}\,}}_{N'}(v)\subseteq {{\,\mathrm{\texttt{C}}\,}}_{N'}(w)={{\,\mathrm{\texttt{C}}\,}}_{N'}(w')$ or ${{\,\mathrm{\texttt{C}}\,}}_{N'}(w')={{\,\mathrm{\texttt{C}}\,}}_{N'}(w)\subseteq {{\,\mathrm{\texttt{C}}\,}}_{N'}(v)$ In summary, therefore, $N'$ satisfies (PCC). $\square$

Corollary 3 and Lemma 29 imply that semi-regularity is preserved by ${{\,\mathrm{\textsc {expd}}\,}}$ applied on arbitrary vertices and ${{\,\mathrm{\textsc {cntr}}\,}}(u,w)$ applied to arcs (*u*, *w*) where ${{\,\textrm{outdeg}\,}}(u)=1$.

#### Corollary 12

Let *N* be a network, $w\in V(N)$, and $N'$ the network obtained from *N* by applying ${{\,\mathrm{\textsc {expd}}\,}}(w)$. Then, *N* is semi-regular if and only if $N'$ is semi-regular.

#### Proposition 5

Let *N* be a network satisfying (PCC). The unique regular network ${\mathfrak {H}}[\mathscr {C}_N]$ is obtained from *N* by repeatedly executing one of the following operations (1) and (2) until neither of them is possible:

1. remove a shortcut (*u*, *w*), and
2. apply ${{\,\mathrm{\textsc {cntr}}\,}}(u,w)$ for an arc (*u*, *w*) with ${{\,\textrm{outdeg}\,}}(u)=1$.

#### Proof

Let $N'$ be the network obtained by applying one of the following operations until neither of them is possible. By construction, we have $V(N')\subseteq V(N)$. We can repeatedly apply Lemmas 1 and 4 to conclude that $N'$ is a network with leaf set *X* and that, for all $v,v'\in V(N')$, it holds $v\preceq _{N} v'$ if and only if $v\preceq _{N'} v'$. Similarly, Lemmas 1 and 4 imply that ${{\,\mathrm{\texttt{C}}\,}}_N(v)={{\,\mathrm{\texttt{C}}\,}}_{N'}(v)$ holds for all $v\in V(N')$ and, in particular, $\mathscr {C}_N=\mathscr {C}_{N'}$.

By assumption, *N* satisfies (PCC), i.e., for all $u, v\in V(N)$, it holds that *u* and *v* are $\preceq _N$-comparable if and only if ${{\,\mathrm{\texttt{C}}\,}}_N(u)\subseteq {{\,\mathrm{\texttt{C}}\,}}_N(v)$ or ${{\,\mathrm{\texttt{C}}\,}}_N(v)\subseteq {{\,\mathrm{\texttt{C}}\,}}_N(u)$. To see that $N'$ also satisfies (PCC), consider $u, v\in V(N')$. Suppose *u* and *v* are $\preceq _{N'}$-comparable. Hence, *u* and *v* are also $\preceq _{N}$-comparable, and thus, ${{\,\mathrm{\texttt{C}}\,}}_{N'}(u)={{\,\mathrm{\texttt{C}}\,}}_N(u)\subseteq {{\,\mathrm{\texttt{C}}\,}}_N(v)={{\,\mathrm{\texttt{C}}\,}}_{N'}(v)$ or ${{\,\mathrm{\texttt{C}}\,}}_{N'}(v)={{\,\mathrm{\texttt{C}}\,}}_N(v)\subseteq {{\,\mathrm{\texttt{C}}\,}}_N(u)={{\,\mathrm{\texttt{C}}\,}}_{N'}(u)$. Conversely, if ${{\,\mathrm{\texttt{C}}\,}}_{N'}(u)\subseteq {{\,\mathrm{\texttt{C}}\,}}_{N'}(v)$ or ${{\,\mathrm{\texttt{C}}\,}}_{N'}(v)\subseteq {{\,\mathrm{\texttt{C}}\,}}_{N'}(u)$, then also ${{\,\mathrm{\texttt{C}}\,}}_{N}(u)\subseteq {{\,\mathrm{\texttt{C}}\,}}_{N}(v)$ or ${{\,\mathrm{\texttt{C}}\,}}_{N}(v)\subseteq {{\,\mathrm{\texttt{C}}\,}}_{N}(u)$. Hence, *u* and *v* are $\preceq _N$-comparable and thus also $\preceq _{N'}$-comparable. Therefore, $N'$ satisfies (PCC). Since moreover $N'$ is shortcut-free by construction, $N'$ is semi-regular. This together with Theorem 2 and the fact that, by construction, there is no vertex *v* with ${{\,\textrm{outdeg}\,}}_{N'}(v)=1$ implies that $N'$ is regular. This, together with $\mathscr {C}_N=\mathscr {C}_{N'}$, implies that $N'$ is the unique regular network ${\mathfrak {H}}[\mathscr {C}_N]$. $\square$

#### Proposition 6

Let *N* be a least-resolved network satisfying (PCC). Then, *N* is uniquely determined by $\mathscr {C}_N$ and, in particular, regular so that $N \sim {\mathfrak {H}}({\mathscr {C}}_N )$.

#### Proof

By Corollary 2, *N* is shortcut-free (and thus, semi-regular) and contains no vertex with outdegree 1. By Theorem 2, therefore, *N* is regular. $\square$

As a consequence of Corollary 8, Theorem 2, and Proposition 6, we obtain

#### Theorem 3

A network *N* is regular if and only if *N* is least-resolved and satisfies (PCC).

### Separated networks and cluster networks

Recall that a network *N* is *separated* if all hybrid vertices have outdegree 1. We have already seen above that the Hasse diagrams of the clustering systems cannot produce separated networks with hybrid vertices because ${{\,\textrm{outdeg}\,}}(v)=1$ implies that ${{\,\mathrm{\texttt{C}}\,}}(v)={{\,\mathrm{\texttt{C}}\,}}(u)$ whenever *u* is the only child of *v*. By Theorem 2, furthermore, a regular network does not have any vertex with outdegree 1. Therefore, a regular network cannot be separated whenever it contains at least one hybrid vertex and *vice versa*.

The *Cluster-popping* algorithm (Huson and Rupp 2008) constructs a separated network for a given clustering system $\mathscr {C}$ by first constructing the Hasse diagram, and thus the unique regular network ${\mathfrak {H}}[\mathscr {C}]$, and then applying ${{\,\mathrm{\textsc {expd}}\,}}(w)$ to all hybrid vertices $w\in V({\mathfrak {H}}[\mathscr {C}])$. In particular, the resulting network is a so-called *cluster network* (Huson and Rupp 2008; Huson and Scornavacca 2011):

#### Definition 14

A network *N* is a *cluster network* if (i) it satisfies (PCC), and, for all $u,v\in V(N)$, it holds

(b) ${{\,\mathrm{\texttt{C}}\,}}_{N}(u)= {{\,\mathrm{\texttt{C}}\,}}_{N}(v)$ if and only if $u= v$ or *v* is a hybrid vertex and parent of *u* or *vice versa*,
(c) if *u* is a child of *v*, then there exists no node *w* with ${{\,\mathrm{\texttt{C}}\,}}_{N}(u) \subsetneq {{\,\mathrm{\texttt{C}}\,}}_{N}(w) \subsetneq {{\,\mathrm{\texttt{C}}\,}}_{N}(v)$, and
(d) every hybrid vertex *v* has exactly one child, which is a tree node.

We note that the definition of cluster networks usually is expressed using the following condition instead of (PCC):

(i’): ${{\,\mathrm{\texttt{C}}\,}}_{N}(u)\subseteq {{\,\mathrm{\texttt{C}}\,}}_{N}(v)$ if and only if $u\preceq _{N} v$ for all $u,v\in V(N)$ (Huson and Rupp 2008; Zhang 2019).

However, this contradicts the existence of hybrid vertices *v*, which are required to have exactly one child *u* by (iv). To see this, observe that $u\prec _{N} v$ and, by Observation 5, we have ${{\,\mathrm{\texttt{C}}\,}}_{N}(u)= {{\,\mathrm{\texttt{C}}\,}}_{N}(v)$. The latter means that ${{\,\mathrm{\texttt{C}}\,}}_{N}(v)\subseteq _{N} {{\,\mathrm{\texttt{C}}\,}}_{N}(u)$ is satisfied and thus (i’) implies $v\preceq _{N} u$, a contradiction.

We can rephrase conditions (i)-(iv), and thus the definition of cluster networks, as follows:

#### Theorem 4

A network *N* is a cluster network if and only if it is semi-regular, separated, and phylogenetic.

#### Proof

Suppose first that *N* is a cluster network, i.e., it satisfies conditions (i)-(iv) in Definition 14. By condition (i) and (iv), resp., *N* satisfies (PCC) and is separated. Hence, it remains to show that *N* is shortcut-free and phylogenetic. Suppose, for contradiction, that (*v*, *u*) is a shortcut in *N*. Then, there is $w\in {{\,\textrm{child}\,}}_{N}(v)\setminus \{u\}$ and $w'\in {{\,\textrm{par}\,}}_{N}(u)\setminus \{v\}$ such that $u\prec _{N}w'\preceq _{N} w$. By Lemma 17, ${{\,\mathrm{\texttt{C}}\,}}_{N}(u)\subseteq {{\,\mathrm{\texttt{C}}\,}}_{N}(w') \subseteq {{\,\mathrm{\texttt{C}}\,}}_{N}(w) \subseteq {{\,\mathrm{\texttt{C}}\,}}_{N}(v)$. If ${{\,\mathrm{\texttt{C}}\,}}_{N}(w)={{\,\mathrm{\texttt{C}}\,}}_{N}(v)$, then conditions (ii) and (iv) imply that *v* is a hybrid vertex with exactly one child, a contradiction. Therefore, ${{\,\mathrm{\texttt{C}}\,}}_{N}(w)\subsetneq {{\,\mathrm{\texttt{C}}\,}}_{N}(v)$ must hold. If ${{\,\mathrm{\texttt{C}}\,}}_{N}(u)={{\,\mathrm{\texttt{C}}\,}}_{N}(w')$, then conditions (ii) and (iv) imply that $w'$ is a hybrid vertex and its unique child *u* is a tree node, contradicting that $w',v\in {{\,\textrm{par}\,}}_{N}(u)$. Hence, we have ${{\,\mathrm{\texttt{C}}\,}}_{N}(u)\subsetneq {{\,\mathrm{\texttt{C}}\,}}_{N}(w') \subseteq {{\,\mathrm{\texttt{C}}\,}}_{N}(w) \subsetneq {{\,\mathrm{\texttt{C}}\,}}_{N}(v)$, which contradicts (iii). Therefore, *N* must be shortcut-free and thus semi-regular. Suppose, for contradiction, that *N* is not phylogenetic. Hence, there is a vertex *v* with exactly one child *u* and ${{\,\textrm{indeg}\,}}_{N}(v)\le 1$. By Observation 5, we have ${{\,\mathrm{\texttt{C}}\,}}_{N}(u)= {{\,\mathrm{\texttt{C}}\,}}_{N}(v)$ and thus *v* must be a hybrid vertex by (ii), a contradiction.

Conversely, suppose *N* is semi-regular, separated, and phylogenetic. Hence, *N* satisfies (PCC), i.e., condition (i). Condition (iii) must be satisfied since an arc (*v*, *u*) with ${{\,\mathrm{\texttt{C}}\,}}_{N}(u) \subsetneq {{\,\mathrm{\texttt{C}}\,}}_{N}(w) \subsetneq {{\,\mathrm{\texttt{C}}\,}}_{N}(v)$ for some $w\in V(N)$ would be a shortcut by Observation 7. We continue with showing (ii). Suppose ${{\,\mathrm{\texttt{C}}\,}}_{N}(u)= {{\,\mathrm{\texttt{C}}\,}}_{N}(v)$ and $u\ne v$. By (PCC), it holds $u\prec _{N} v$ or $v\prec _{N} u$. Suppose w.l.o.g. that $u\prec _{N} v$. Then, Lemma 26 implies that ${{\,\textrm{outdeg}\,}}_{N}(v)=1$ and *u* is the unique child of *v*. Since *N* is phylogenetic, *v* must be a hybrid vertex. Conversely, a hybrid vertex *v* in a separated network has exactly one child *u* implying ${{\,\mathrm{\texttt{C}}\,}}_{N}(u)= {{\,\mathrm{\texttt{C}}\,}}_{N}(v)$ by Observation 5. Since *N* is separated, it remains to show that the unique child *u* of a hybrid vertex *v* is a tree node. Suppose for contradiction that *u* is a hybrid node. Then, *u* again has a unique child *w*. Hence, we have $w\prec _{N} u\prec _{N} v$ and, by Observation 5, it holds ${{\,\mathrm{\texttt{C}}\,}}_{N}(w)={{\,\mathrm{\texttt{C}}\,}}_{N}(u)= {{\,\mathrm{\texttt{C}}\,}}_{N}(v)$. By (ii), this implies that (*v*, *w*) is an arc in *N* and, in particular, a shortcut, a contradiction. Therefore, condition (iv) is also satisfied. $\square$

We shall see in Theorem 6 in the following section that cluster networks are uniquely determined by their cluster sets. To obtain this result, it will be convenient to make use of the fact that the semi-regular networks are encoded by their multisets of clusters.

### Cluster multisets of semi-regular networks

#### Lemma 30

Let *N* be a semi-regular phylogenetic network. Then, the multiplicity of each cluster $C\in \mathscr {C}$ in the cluster multiset $\mathscr {M}_N$ is either one or two. In the latter case, the two distinct vertices $u,v\in V(N)$ with ${{\,\mathrm{\texttt{C}}\,}}_{N}(u)={{\,\mathrm{\texttt{C}}\,}}_{N}(v)=C$ are adjacent.

#### Proof

Recall that a semi-regular network *N* satisfies (PCC) and is shortcut-free. Let $C\in \mathscr {C}$. Thus, there is at least one vertex $v\in V(N)$ with ${{\,\mathrm{\texttt{C}}\,}}_{N}(v)=C$. Now, suppose there is $u\in V(N){\setminus } \{v\}$ with ${{\,\mathrm{\texttt{C}}\,}}_{N}(u)=C$. By (PCC), it holds $u\prec _{N} v$ or $v\prec _{N} u$. Suppose that $u\prec _{N} v$. Then, Lemma 26 implies that ${{\,\textrm{outdeg}\,}}_{N}(v)=1$ and *u* is the unique child of *v*. Suppose, for contradiction, there is a third vertex $w\in V(N)\setminus \{u,v\}$. By (PCC), it holds $w\prec _{N} v$ or $v\prec _{N} w$. If $w\prec _{N} v$, then Lemma 26 implies that *w* is the unique child of *v*, a contradiction. If $v\prec _{N} w$, then we have also $u\prec _{N} v\prec _{N} w$. By Lemma 26, therefore, *v* and *u* are both the unique child of *w*, which is not possible since $u\ne v$. One argues similarly if $v\prec _{N} u$. In particular, *u* and *v* are adjacent in both cases. $\square$

As we shall see in Theorem 5, the property of being phylogenetic, however, is not necessary for semi-regular networks to be identified by their cluster multisets. In order to prove this, the following map will be of useful.

#### Definition 15

Let *N* and $\tilde{N}$ be two networks satisfying (PCC) and $\mathscr {M}_N=\mathscr {M}_{\tilde{N}}$. Then, the map $\varphi _{PCC}:V(N)\rightarrow V(\tilde{N})$ is given by the following steps for all $C\in \mathscr {C}_N=\mathscr {C}_{\tilde{N}}$:

(i) sort the $k\ge 1$ vertices in *N* with cluster *C* such that $v_1 \prec _N \dots \prec _N v_k$,
(ii) sort the *k* vertices in $\tilde{N}$ with cluster *C* such that $\tilde{v}_1 \prec _{\tilde{N}} \dots \prec _{\tilde{N}} \tilde{v}_k$, and
(iii) set $\varphi (v_i)=\tilde{v}_i$ for all $1\le i \le k$.

In other words, we map the $\preceq _{N}$-larger vertices with cluster *C* in *N* to $\preceq _{\tilde{N}}$-larger vertices with cluster *C* in $\tilde{N}$, which is possible since, by (PCC), these vertices are totally ordered w.r.t. $\preceq _{N}$ and $\preceq _{\tilde{N}}$, respectively.

#### Lemma 31

Let *N* and $\tilde{N}$ be two networks satisfying (PCC) and $\mathscr {M}_N=\mathscr {M}_{\tilde{N}}$. Then, $\varphi _{PCC}$ is a bijection between *V*(*N*) and $V(\tilde{N})$ that is the identity on the common leaf set *X*. Writing $\tilde{v}{:}{=}\varphi _{PCC}(v)$, it moreover holds

1. $${{\,\mathrm{\texttt{C}}\,}}_{N}(v)={{\,\mathrm{\texttt{C}}\,}}_{\tilde{N}}(\tilde{v})$$ for all $$v\in V(N)$$,
2. *v* is a leaf if and only if $\tilde{v}$ is a leaf for all $v\in V(N)$, and
3. $u\prec _{N} v$ if and only if $\tilde{u}\prec _{\tilde{N}} \tilde{v}$ for all $u,v\in V(N)$.

#### Proof

Since $\mathscr {M}_N=\mathscr {M}_{\tilde{N}}$, the multiplicity of every cluster $C\in \mathscr {C}{:}{=}\mathscr {C}_N=\mathscr {C}_{\tilde{N}}$ is equal in $\mathscr {M}_N$ and $\mathscr {M}_{\tilde{N}}$, i.e., there are $k\ge 1$ vertices with cluster *C* in *N* and *k* vertices with cluster *C* in $\tilde{N}$. One easily verifies that, by construction, $\varphi _{PCC}$ is a bijection between *V*(*N*) and $V(\tilde{N})$ that is the identity on the common leaf set *X* and satisfies ${{\,\mathrm{\texttt{C}}\,}}_{N}(v)={{\,\mathrm{\texttt{C}}\,}}_{\tilde{N}}(\tilde{v})$ for all $v\in V(N)$. To see that $u\prec _{N} v$ if and only if $\tilde{u}\prec _{\tilde{N}} \tilde{v}$, suppose $u\prec _{N} v$. By Lemma 17, this implies ${{\,\mathrm{\texttt{C}}\,}}_{N}(u)\subseteq {{\,\mathrm{\texttt{C}}\,}}_N(v)$. If ${{\,\mathrm{\texttt{C}}\,}}_{N}(u)\subsetneq {{\,\mathrm{\texttt{C}}\,}}_N(v)$ (and thus ${{\,\mathrm{\texttt{C}}\,}}_{\tilde{N}}(\tilde{u})\subsetneq {{\,\mathrm{\texttt{C}}\,}}_{\tilde{N}}(\tilde{v})$), then Observation 7 implies $\tilde{u}\prec _{\tilde{N}} \tilde{v}$. If, on the other hand, ${{\,\mathrm{\texttt{C}}\,}}_{N}(u)= {{\,\mathrm{\texttt{C}}\,}}_N(v)$, then $\tilde{u}\prec _{\tilde{N}} \tilde{v}$ holds by construction of $\varphi$. Analogously, $\tilde{u}\prec _{\tilde{N}} \tilde{v}$ implies $u\prec _{N} v$. In particular, this implies that, for every $v\in V(N)$, *v* is a leaf if and only if $\tilde{v}$ is a leaf. $\square$

#### Theorem 5

Let *N* be a semi-regular network. Then, *N* is the unique semi-regular network whose cluster multiset is $\mathscr {M}_N$.

#### Proof

Suppose *N* and $\tilde{N}$ are semi-regular networks with $\mathscr {M}_N=\mathscr {M}_{\tilde{N}}$. By assumption, both *N* and $\tilde{N}$ are shortcut-free and satisfy (PCC). We continue with showing that $\varphi _{PCC}:V(N)\rightarrow V(\tilde{N})$ is a graph isomorphism. By Lemma 31, $\varphi _{PCC}$ is a bijection that is the identity on the common leaf set *X*. In the following, we write $\tilde{v}{:}{=}\varphi _{PCC}(v)$ for all $v\in V(N)$. Suppose that $(v,u)\in E(N)$. Thus, we have $u\prec _{N} v$ which implies $\tilde{u}\prec _{\tilde{N}} \tilde{v}$ by Lemma 31(3). Assume, for contradiction, that $(\tilde{v},\tilde{u})\notin E(\tilde{N})$. Then, there must be $\tilde{z}\in V(\tilde{N})$ such that $\tilde{u}\prec _{\tilde{N}} \tilde{z} \prec _{\tilde{N}} \tilde{v}$. By Lemma 31(3), we have $u\prec _{N} z\prec _{N} v$. Hence, the arc (*v*, *u*) must be a shortcut in *N*, a contradiction. Therefore, $(\tilde{v},\tilde{u})\in E(\tilde{N})$. By analogous arguments, $(\tilde{v},\tilde{u})\in E(\tilde{N})$ implies $(v,u)\in E(N)$. Hence, $\varphi _{PCC}$ is a graph isomorphism that is the identity on *X* and thus $N\simeq \tilde{N}$. Therefore, *N* is the unique semi-regular network whose cluster multiset is $\mathscr {M}_N$. $\square$

**Fig. 9:**
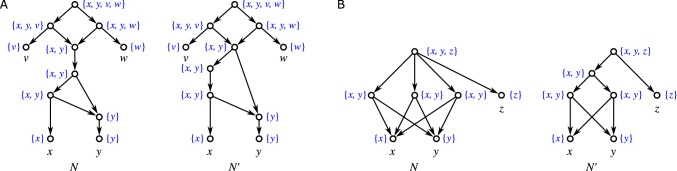
Two pairs of non-isomorphic (phylogenetic) networks *N* and $N'$ for which $\mathscr {M}_{N} = \mathscr {M}_{N'}$. **A** *N* and $N'$ satisfy (PCC) but are not shortcut-free. **B** *N* and $N'$ are shortcut-free but do not satisfy (PCC)

We emphasize that none of the two conditions (PCC) and shortcut-free that define semi-regular networks can be omitted in Theorem 5 as shown by the examples in Fig. 9.

**Fig. 10:**
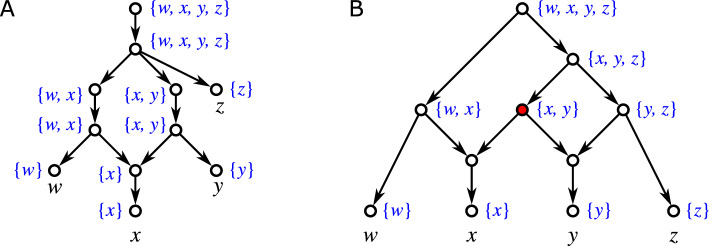
**A** Example of a network that is semi-regular and tree-child but not phylogenetic. **B** Example of a cluster network, thus satisfying (PCC), that is not tree-child. The central node marked in red does not have a tree-child. One easily checks that it satisfies (PCC) since vertices associated with overlapping pairs of clusters are incomparable (color figure online)

It is an easy task to verify that semi-regular networks *N* (as any other network for which the property of being phylogenetic has been left out) are not determined by their clustering systems $\mathscr {C}_N$. The network *N* in Fig. 10A, for example, is not phylogenetic (but semi-regular). Suppression of any vertex with in- and outdegree 1 yields a network $N'$ with $N\not \simeq N'$ but $\mathscr {C}_N = \mathscr {C}_{N'}$. We will see in the following that there is a 1-to-1 correspondence between clustering systems and cluster networks. To show this, we will need the following technical result that relates the occurrence of vertices with equal clusters in such networks to the structure of the Hasse diagram.

#### Lemma 32

Let *N* be a cluster network with clustering system $\mathscr {C}$. Then, for every cluster $C\in \mathscr {C}$, there is either exactly one vertex $v\in V(N)$ with ${{\,\mathrm{\texttt{C}}\,}}_{N}(v)=C$ or there are exactly two vertices $u,v\in V(N)$ with ${{\,\mathrm{\texttt{C}}\,}}_{N}(u)={{\,\mathrm{\texttt{C}}\,}}_{N}(v)=C$. The latter case occurs if and only if *C* has indegree at least 2 in ${\mathfrak {H}}[\mathscr {C}]$. Moreover, in this case, *u* and *v* are adjacent in *N*.

#### Proof

By Theorem 4, *N* is phylogenetic, separated, and semi-regular, i.e., it satisfies (PCC) and is shortcut-free. By Lemma 30, it only remains to show that there are two distinct vertices $u,v\in V(N)$ with ${{\,\mathrm{\texttt{C}}\,}}_{N}(u)={{\,\mathrm{\texttt{C}}\,}}_{N}(v)=C$ if and only if *C* has indegree at least 2 in ${\mathfrak {H}}[\mathscr {C}]$.

Suppose $C={{\,\mathrm{\texttt{C}}\,}}_{N}(v)={{\,\mathrm{\texttt{C}}\,}}_{N}(u)$ for two distinct vertices $v,u\in V(N)$ and assume w.l.o.g. that $u\prec _{N} v$. By Lemma 26, *u* must be the unique child of *v*. Since *N* is phylogenetic, this implies that ${{\,\textrm{indeg}\,}}_{N}(v)\ge 2$. Thus, let $v_1$ and $v_2$ be two distinct parents of *v*. Since *N* is shortcut-free and by Observation 3, $v_1$ and $v_2$ are $\prec _{N}$-incomparable. Using (PCC), we conclude that $C_1{:}{=}{{\,\mathrm{\texttt{C}}\,}}_{N}(v_1)$ and $C_2{:}{=}{{\,\mathrm{\texttt{C}}\,}}_{N}(v_2)$ are distinct and none of them is contained in the other. Moreover, we have $C\subseteq C_1\cap C_2$ by Lemma 17, and thus, and $C\subsetneq C_1,C_2$. Suppose there is $C'\in \mathscr {C}$ such that $C\subsetneq C'\subsetneq C_1$. Let $v'\in V(N)$ be a vertex with ${{\,\mathrm{\texttt{C}}\,}}_{N}(v')=C'$. By Observation 7, we have $v\prec _{N} v'\prec _{N}v_1$. Therefore, $(v_1,v)\in V(N)$ must be a shortcut, a contradiction. Hence, there is no $C'\in \mathscr {C}$ with $C\subsetneq C'\subsetneq C_1$ and $C_1$ must be a parent of *C* in ${\mathfrak {H}}[\mathscr {C}]$. By similar arguments, $C_2$ is a parent of *C* in ${\mathfrak {H}}[\mathscr {C}]$, which together with $C_1\ne C_2$ implies that *C* has indegree at least 2 in ${\mathfrak {H}}[\mathscr {C}]$.

Conversely, suppose *C* has at least two distinct parents $C_1$ and $C_2$ in ${\mathfrak {H}}[\mathscr {C}]$. Hence, it holds $C\subsetneq C_1$ and $C\subsetneq C_2$ and none of $C_1$ and $C_2$ is contained in the other. Let $v, v_1, v_2\in V(N)$ be vertices with $C= {{\,\mathrm{\texttt{C}}\,}}_{N}(v)$, $C_1= {{\,\mathrm{\texttt{C}}\,}}_{N}(v_1)$, and $C_2= {{\,\mathrm{\texttt{C}}\,}}_{N}(v_2)$. Clearly, *v*, $v_1$, and $v_2$ are pairwise distinct. By Observation 7, we have $v \prec _{N} v_1$ and $v \prec _{N} v_2$, i.e., there are a $v_1 v$-path $P_1$ and a $v_2 v$-path $P_2$ in *N*. Let $v'$ be the $\preceq _{N}$-maximal vertex in $P_1$ that is also a vertex in $P_2$. We distinguish the two cases $v'= v$ and $v'\ne v$. If $v'= v$, then *v* has a parent in each of $P_1$ and $P_2$ which are distinct by construction. Thus, *v* is a hybrid vertex. Since moreover *N* is separated, *v* has a unique child *u*. By Observation 5, this implies ${{\,\mathrm{\texttt{C}}\,}}_{N}(u)={{\,\mathrm{\texttt{C}}\,}}_{N}(v)=C$. Now, suppose $v'\ne v$. Lemma 17 and $v\prec _{N} v'\preceq _{N} v_1, v_2$ imply $C\subseteq C'{:}{=}{{\,\mathrm{\texttt{C}}\,}}_{N}(v')$, $C'\subseteq C_1$ and $C'\subseteq C_2$. Since none of $C_1$ and $C_2$ is contained in the other, we must have $C\subseteq C'\subsetneq C_1$ and $C\subseteq C'\subsetneq C_2$. Since $C_1$ and $C_2$ are parents of *C* in ${\mathfrak {H}}[\mathscr {C}]$, the latter is only possible if $C=C'$. Hence, in both cases, there are two distinct vertices in *N* with cluster *C*. $\square$

#### Theorem 6

For every clustering system $\mathscr {C}$, there is a unique cluster network *N* with $\mathscr {C}=\mathscr {C}_N$. It is obtained from the unique regular network ${\mathfrak {H}}[\mathscr {C}]$ of $\mathscr {C}$ by applying ${{\,\mathrm{\textsc {expd}}\,}}(v)$ to all hybrid vertices. In particular, *N* is the unique semi-regular separated phylogenetic network with clustering system $\mathscr {C}$.

#### Proof

By Theorem 2, the unique regular network ${\mathfrak {H}}[\mathscr {C}]$ is shortcut-free, satisfies (PCC), and has no vertex with outdegree 1. In particular, ${\mathfrak {H}}[\mathscr {C}]$ is phylogenetic. Now, let *N* be the network obtained from ${\mathfrak {H}}[\mathscr {C}]$ by repeatedly applying ${{\,\mathrm{\textsc {expd}}\,}}(w)$ to some hybrid vertex whose outdegree is not 1 until no such vertex exists. Clearly this is achieved by applying ${{\,\mathrm{\textsc {expd}}\,}}(w)$ to all hybrid vertices $w\in V({\mathfrak {H}}[\mathscr {C}])$ since they all satisfy ${{\,\textrm{outdeg}\,}}_{{\mathfrak {H}}[\mathscr {C}]}(w)\ne 1$ and, moreover, no expansion step introduces new such vertices but reduces their number by 1. We can repeatedly (i.e., in each expansion step) apply Lemma 5 to conclude that the resulting digraph *N* is a phylogenetic network that satisfies $\mathscr {C}_{N}=\mathscr {C}_{{\mathfrak {H}}[\mathscr {C}]}=\mathscr {C}$, and Corollary 12 to conclude that *N* is semi-regular. In particular, by construction, all hybrid vertices in *N* have outdegree 1, i.e., *N* is separated. By Theorem 4, *N* is a cluster network.

It remains to show that *N* is the unique cluster network with clustering system $\mathscr {C}$. To this end, let $\tilde{N}$ be a cluster network with $\mathscr {C}=\mathscr {C}_{\tilde{N}}$. By Theorem 4, both *N* and $\tilde{N}$ are semi-regular, shortcut-free, and phylogenetic. Moreover, by Lemma 32, for every cluster $C\in \mathscr {C}$, *C* has multiplicity 2 in $\mathscr {M}_N$ if and only if *C* has indegree at least 2 in ${\mathfrak {H}}[\mathscr {C}]$ if and only if *C* has multiplicity 2 in $\mathscr {M}_{\tilde{N}}$; and multiplicity 1 in both $\mathscr {M}_N$ and $\mathscr {M}_{\tilde{N}}$ otherwise. Hence, we have $\mathscr {M}_N=\mathscr {M}_{\tilde{N}}$. By Theorem 5, we conclude that $N\simeq \tilde{N}$, and thus, *N* is the unique cluster network. In particular, by Theorem 4, *N* is the unique semi-regular separated phylogenetic network with clustering system $\mathscr {C}$. $\square$

The uniqueness of cluster networks for a given clustering system $\mathscr {C}$ has been proved in the framework of *reticulate networks* in (Theorem 3.9 Alcalà et al. 2014), using alternative arguments. A network *N* is *reticulate* in the sense of (Definition 2.3 Alcalà et al. 2014) if (a) every hybrid vertex has exactly one child which, moreover, must be a tree vertex, and (b) if a vertex *v* has ${{\,\textrm{outdeg}\,}}_{N}(v)=1$ and ${{\,\textrm{indeg}\,}}_{N}(v)\le 1$ then *v* has a unique child and parent, both of which are hybrid vertices. The following result shows that the additional condition that *N* is reticulate does not affect cluster networks, and thus, Theorem 6 and (Theorem 3.9 Alcalà et al. 2014) are equivalent.

#### Proposition 7

If *N* is a cluster network, then *N* is reticulate.

#### Proof

By Theorem 4, *N* is semi-regular, phylogenetic, and separated. Since *N* is phylogenetic, it does not contain a vertex satisfying condition (b), and hence (b) is satisfied trivially. Since *N* is separated, every hybrid vertex *v* has exactly one child *u*. By Obs 5, we have ${{\,\mathrm{\texttt{C}}\,}}(v)={{\,\mathrm{\texttt{C}}\,}}(u)$. Suppose that *u* is also a hybrid vertex, i.e., there is $v'\in {{\,\textrm{par}\,}}_{N}(u)\setminus \{v\}$. Since *N* is shortcut-free, *v* and $v'$ are $\preceq _{N}$-incomparable. However, by Lemma 17, we have ${{\,\mathrm{\texttt{C}}\,}}(v)={{\,\mathrm{\texttt{C}}\,}}(u) \subseteq {{\,\mathrm{\texttt{C}}\,}}(v')$ and thus (PCC) implies that *v* and $v'$ must be $\preceq _{N}$-comparable, a contradiction. Hence, *N* also satisfies condition (a). In summary, every cluster network is reticulate. $\square$

## Tree-child, normal, and tree-based networks

### Definition 16

(Cardona et al. 2009) A network *N* has the *tree-child* property if, for every $v\in V^0$, there is a “tree-child,” i.e., $u\in {{\,\textrm{child}\,}}(v)$ with ${{\,\textrm{indeg}\,}}(u)=1$.

Tree-child networks are not necessarily phylogenetic, see Fig. 10A for an example. As shown in (Lemma 2 Cardona et al. 2009), *N* is a tree-child network if and only if every vertex $v\in V$ has a strict descendant, i.e., a leaf $x\in X$ such that every directed path from the root $\rho _N$ to *x* contains *v*.

### Lemma 33

Suppose *N* is tree-child and *u* and *v* are $\preceq _N$-incomparable. Then, there is a vertex $x\in {{\,\mathrm{\texttt{C}}\,}}(v)$ such that $x\notin {{\,\mathrm{\texttt{C}}\,}}(u)$ and $y\in {{\,\mathrm{\texttt{C}}\,}}(u)$ such that $y\notin {{\,\mathrm{\texttt{C}}\,}}(v)$.

### Proof

Since *N* is tree-child, there is a strict descendant *x* of *v*. It satisfies $x\in {{\,\mathrm{\texttt{C}}\,}}(v)$ and every path from the root $\rho _N$ to *x* runs through *v*. Now, suppose $x\in {{\,\mathrm{\texttt{C}}\,}}(u)$. Then, there is a directed path from $\rho _N$ to *x* that contains *u*. Since any such path also contains *v*, the vertices *u* and *v* must be $\preceq _N$-comparable, a contradiction. Thus, $x\notin {{\,\mathrm{\texttt{C}}\,}}(u)$. The same argument shows that there is $y\in {{\,\mathrm{\texttt{C}}\,}}(v)$ with $y\notin {{\,\mathrm{\texttt{C}}\,}}(u)$. $\square$

### Corollary 13

Every tree-child network satisfies (PCC).

### Proof

Let *N* be a tree-child network and $u,v\in V(N)$. By Lemma 17, $u\preceq _N v$ implies ${{\,\mathrm{\texttt{C}}\,}}(u)\subseteq {{\,\mathrm{\texttt{C}}\,}}(v)$. On the other hand, if *u* and *v* are $\preceq _N$-incomparable, then Lemma 33 implies that either ${{\,\mathrm{\texttt{C}}\,}}(u)\cap {{\,\mathrm{\texttt{C}}\,}}(v)=\emptyset$ or ${{\,\mathrm{\texttt{C}}\,}}(u)$ and ${{\,\mathrm{\texttt{C}}\,}}(v)$ overlap and thus neither ${{\,\mathrm{\texttt{C}}\,}}(u)\subseteq {{\,\mathrm{\texttt{C}}\,}}(v)$ nor ${{\,\mathrm{\texttt{C}}\,}}(v)\subseteq {{\,\mathrm{\texttt{C}}\,}}(u)$ is satisfied. $\square$

The converse is not true. Figure 10B shows an example of a network that satisfies (PCC) but does not have the tree-child property.

### Definition 17

A network is *normal* if it is tree-child and shortcut-free.

Willson (2010) studies normal networks in a somewhat different setting, in which *X* comprises not only the leaves in our sense but also the root and all vertices with outdegree 1. Under this assumption, (Theorem 3.4 Willson 2010) states that “*N* is regular whenever it is normal.” The absence of vertices with outdegree 1 can be included as an extra condition. The analog of Willson’s result in our setting follows immediately from Corollary 13, Theorem 2, and the absence of shortcuts:

### Corollary 14

Let *N* be a network. If *N* is normal, then *N* is semi-regular. If, in addition, there are no vertices with outdegree 1, then *N* is regular.

The converse is not true, there are (semi-)regular networks that are not normal, see Fig. 1 of Willson (2010) and also Fig. 10B for a semi-regular example. Hence, we have

### Remark 2

Not every semi-regular network, and in particular not every cluster network, is normal.

### Proposition 8

Let $\mathscr {C}$ be a clustering system. If there is a phylogenetic, separated, normal network *N* with $\mathscr {C}=\mathscr {C}_N$, then *N* is unique w.r.t. these properties. In particular, *N* is the unique cluster network with $\mathscr {C}=\mathscr {C}_N$.

### Proof

Suppose *N* is phylogenetic, separated, and normal and satisfies $\mathscr {C}=\mathscr {C}_N$. By Cor 14, *N* is semi-regular and thus, by Theorem 4, a cluster network. By Theorem 6, *N* is unique. $\square$

From Proposition 8 and the definition of binary networks, we immediately obtain

### Corollary 15

Let *N* be a binary normal network. Then, *N* is the unique binary normal network whose cluster set is $\mathscr {C}_N$. In particular, *N* is a cluster network.

The following result appears to be well known, see, e.g., Murakami et al. (2019). An argument for binary tree-child and level-1 networks can be found Huber et al. (2019b). A direct proof for our more general setting is included here for completeness.

### Proposition 9

Every phylogenetic level-1 network is tree-child.

### Proof

Let *N* be a phylogenetic level-1 network. If *v* is a hybrid vertex, then, by Lemma 10, there is a non-trivial block $B_v$ that contains *v* and all its parents. Suppose, for contradiction, there is a non-leaf vertex *v* whose children are all hybrid vertices. Suppose first that ${{\,\textrm{outdeg}\,}}(v)=1$. Since *N* is phylogenetic, this implies that *v* is hybrid vertex. Let *u* be the unique child of *v*, which is a hybrid vertex by assumption. The hybrid vertices *u* and *v* are contained in a common non-trivial block $B_u$. In particular, $u\prec _{N} v$ implies $u\ne \max B_u$. Additionally, $v\ne \max B_u$ since ${{\,\textrm{outdeg}\,}}(v)=1$ but ${{\,\textrm{outdeg}\,}}(\max B_u)>1$ by Lemma 4; contradicting that *N* is level-1. Now, suppose that ${{\,\textrm{outdeg}\,}}(v)\ge 2$. Since all $u_i\in {{\,\textrm{child}\,}}(v)$ are hybrid vertices, *v* and $u_i$ are contained in blocks $B_i{:}{=}B_{u_i}$. Note that $u_i\ne \max B_i$. If there are two distinct $u_i,u_j\in {{\,\textrm{child}\,}}(v)$ such that $v\notin \{\max B_i, \max B_j\}$, then Lemma 9 implies $B_i=B_j$ and thus $B_i$ contains the hybrid vertices $u_i,u_j\ne \max B_i$; this contradicts that *N* is level-1. Otherwise, there is at least one $u_i\in {{\,\textrm{child}\,}}(v)$ such that $v=\max B_i$. Since $u_i$ is a hybrid vertex, there is $w_i\in {{\,\textrm{par}\,}}_{N}(u_i){\setminus } \{v\}$, which is also contained in $B_i$. Hence, we have $u_i\prec _{N} w_i\prec _{N} \max B_i = v$. Therefore, and because *N* is acyclic, there is $u_j\in {{\,\textrm{child}\,}}_{N}(v)$ such that $w_i\preceq _{N} u_j\prec _{N} v$. By assumption, $u_j$ is a hybrid vertex and moreover $u_j\notin \{u_i, v=\max B_i \}$. By Lemma 7, $w_i\preceq _{N} u_j\prec _{N} v$ and $w_i,v\in V(B_i)$ imply $u_j\in V(B_i)$. Hence, $B_i$ contains two distinct hybrid vertices $u_i, u_j\ne \max B_i$, contradicting that *N* is level-1. $\square$

Note that “phylogenetic” cannot be omitted in Proposition 9: Consider a tree vertex *v* with a hybrid child $u\in {{\,\textrm{child}\,}}(v)$. Subdivision of the arc (*v*, *u*) creates a new tree vertex $u'\in {{\,\textrm{child}\,}}(v)$ with the hybrid *u* as its only child. The modified network is still level-1 but no longer tree-child.

Next we consider the overlapping clusters in tree-child networks in some more detail:

### Lemma 34

Let *N* be a tree-child network and suppose *u* and *v* are $\preceq _N$-incomparable. Then, either ${{\,\mathrm{\texttt{C}}\,}}(u)\cap {{\,\mathrm{\texttt{C}}\,}}(v)={{\,\mathrm{\texttt{C}}\,}}(h)$ for some hybrid vertex $h\in V(N)$ or ${{\,\mathrm{\texttt{C}}\,}}(u)\cap {{\,\mathrm{\texttt{C}}\,}}(v)\notin \mathscr {C}_N$.

### Proof

By Corollary 13, *N* satisfies (PCC) and thus either ${{\,\mathrm{\texttt{C}}\,}}(u)\cap {{\,\mathrm{\texttt{C}}\,}}(v)=\emptyset$, in which case the assertion is obviously true, or ${{\,\mathrm{\texttt{C}}\,}}(u)$ and ${{\,\mathrm{\texttt{C}}\,}}(v)$ overlap. In the latter case, Lemma 18 implies that *u*, *v* are contained in a common non-trivial block *B*. Set $A{:}{=}{{\,\mathrm{\texttt{C}}\,}}(u)\cap {{\,\mathrm{\texttt{C}}\,}}(v)$ and assume $A\ne {{\,\mathrm{\texttt{C}}\,}}(h)$ for any hybrid vertex $h\in V(N)$. Lemma 20 implies that $A=\bigcup _{h\in H} {{\,\mathrm{\texttt{C}}\,}}(h)$ for some set *H* of hybrid vertices. Since *A* is non-empty and, by assumption, $A\ne {{\,\mathrm{\texttt{C}}\,}}(h)$ for all hybrid vertices *h*, we must have $\vert H\vert \ge 2$. In particular, therefore, it holds that ${{\,\mathrm{\texttt{C}}\,}}(h)\subsetneq A$ for all $h\in H$. Now, suppose, for contradiction, that there is a non-hybrid vertex $w\in V(N)$ such that ${{\,\mathrm{\texttt{C}}\,}}(w)=\bigcup _{h\in H} {{\,\mathrm{\texttt{C}}\,}}(h)$. Then, for all $h\in H$, we have ${{\,\mathrm{\texttt{C}}\,}}(h)\subsetneq {{\,\mathrm{\texttt{C}}\,}}(w)$, which together with Corollary 7 implies $h\prec _N w$. Moreover, all elements in ${{\,\mathrm{\texttt{C}}\,}}(w)$ are descendants of one of the hybrid vertices in *H*. Since *N* is tree-child, there is a leaf *x* that is reachable from *w* along a path consisting entirely of tree vertices and thus cannot be a descendant of any hybrid vertex $h\prec _N w$. That is, there is $x\in {{\,\mathrm{\texttt{C}}\,}}(w)$ and $x\notin {{\,\mathrm{\texttt{C}}\,}}(h)$ for all $h\in H$, a contradiction. Therefore, $A\notin \mathscr {C}_N$. $\square$

**Fig. 11:**
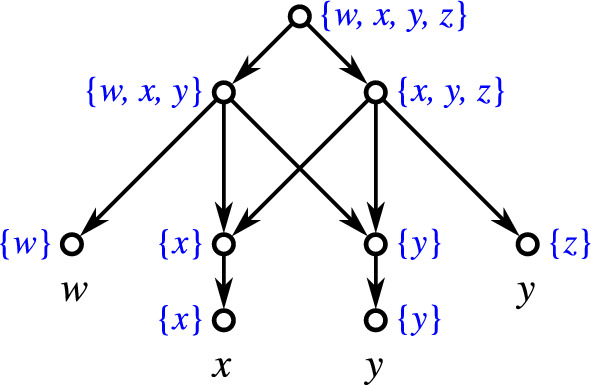
A tree-child network whose clustering system $\mathscr {C}$ is not closed since $\{w,x,y\}\cap \{x,y,z\}=\{x,y\}\notin \mathscr {C}$

In particular, the case ${{\,\mathrm{\texttt{C}}\,}}(u)\cap {{\,\mathrm{\texttt{C}}\,}}(v)\notin \mathscr {C}_N$ in Lemma 34 can indeed occur even if ${{\,\mathrm{\texttt{C}}\,}}(u)\cap {{\,\mathrm{\texttt{C}}\,}}(v)\ne \emptyset$ as the example in Fig. 11 shows. Hence, the clustering system $\mathscr {C}_N$ of a tree-child network *N* is not necessarily closed.

### Corollary 16

Let *N* be a tree-child network with clustering system $\mathscr {C}$ and let $C_1,C_2\in \mathscr {C}$ be a pair of overlapping clusters. Then, $C_1\cap C_2\in \mathscr {C}$ if and only if there is a hybrid vertex $h\in V(N)$ such that $C_1\cap C_2={{\,\mathrm{\texttt{C}}\,}}(h)$.

Another class of networks that has received considerable attention in the last decade are tree-based networks (Francis and Steel 2015; Zhang 2016; Jetten and van Iersel 2018; Pons et al. 2019). They capture the idea that networks can be obtained from (the subdivision of) a tree by inserting additional arcs:

### Definition 18

A network *N* is called *tree-based* with base tree *T* if *N* can be obtained from *T* by (a) subdividing the arcs of *T* by introducing vertices with in- and outdegree 1 (called *attachment points*), and (b) adding arcs (called *linking arcs*) between pairs of vertices, so that *N* remains acyclic.

This definition further generalizes the original one for non-binary tree-based networks in Jetten and van Iersel (2018) in the sense that we do not require the two properties phylogenetic and separated and that we allow to have additional arcs between non-attachment points and attachment points may have in- and outdegree greater than one. This generalization ensures that all trees, i.e., in particular non-phylogenetic trees, remain tree-based.

Equivalently, a network *N* on *X* is tree-based if and only if there is a rooted (not necessarily phylogenetic) spanning tree *T* with leaf set *X*, i.e., there are no *dummy leaves* in *T* that correspond to inner vertices in *N*. Clearly, the unique incoming arc (*u*, *v*) of a tree vertex *v* must be contained in every rooted spanning tree *T* of a network *N* and thus *u* cannot be a dummy leaf in *T*. As an immediate consequence, the well-known fact that tree-child network are always tree-based (Pons et al. 2019) remains also true in our generalized setting: and does in particular, not require the properties phylogenetic and separated:

### Observation 8

Every tree-child network is tree-based.

**Fig. 12:**
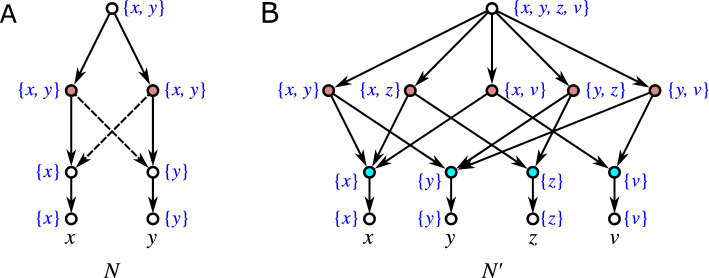
**A** A network that is tree-based (with a possible base tree indicated by the solid-line arcs) but that does not satisfy (PCC). **B** A cluster network (thus satisfying (PCC)) that is not tree-based, see details in the text (color figure online)

Figure 12A shows a network *N* that is tree-based since removal of the dashed arcs results in a rooted spanning tree whose leaves are exactly the leaves of *N*. However, *N* does not satisfy (PCC) since the two vertices highlighted in orange correspond to the same cluster but are $\preceq _{N}$-incomparable. Conversely, the example in Fig. 12B shows that (PCC), or even semi-regularity, does not imply that a network is tree-based. In particular, the network $N'$ is the cluster network for $\mathscr {C}= \{ \{x\}, \{y\}, \{z\}, \{v\}, \{x,y\}, \{x,z\}, \{x,v\}, \{y,z\}, \{y,v\}, \{x,y,z,v\} \}$. To see that $N'$ is not tree-based, consider the set *U* of all inner vertices whose children are all hybrid vertices, called *omnians* in Jetten and van Iersel (2018). These vertices are highlighted in orange. In any rooted spanning tree, each of the four hybrid vertices (highlighted in cyan) has exactly one parent. Since, in addition, none of the five omnians has a child that is not one of the four hybrid vertices, one easily verifies that at least one omnian must be a dummy leaf in every spanning tree. Therefore, $N'$ is not tree-based. The latter observation is in line with Corollary 3.6 in Jetten and van Iersel (2018), which holds for a more restricted definition of tree-based and states that a (phylogenetic, separated) network is tree-based if and only if, for all $S\subseteq U$, the number of different children of the vertices in *S* is greater than or equal to $\vert S\vert$. Clearly, the latter is not satisfied for $S=U$ in the example. We summarize the latter findings in

### Observation 9

Not every tree-based network satisfies (PCC). Moreover, there are cluster networks and thus phylogenetic separated networks that do not satisfy (PCC).

## Least common ancestors and LCA-networks

### Basics

#### Definition 19

Bender et al. (2001) A *least common ancestor* (LCA) of a subset $Y\subseteq V$ in a DAG *N* is an ancestor of all vertices in *Y* that is $\preceq _N$-minimal w.r.t. this property.

In general DAGs *N*, an LCA does not necessarily exist for a given vertex set. Moreover, an LCA is not unique in general. We write ${{\,\textrm{LCA}\,}}(Y)$ for the (possibly) empty set of $\preceq _{N}$-minimal ancestors of the elements in *Y*. In a (phylogenetic) network *N*, the root $\rho _N$ is an ancestor of all vertices in *V*(*N*), and thus, a least common ancestor exists for all $Y\subseteq V(N)$. The LCA sets retain key information on the partial order $\preceq _N$:

#### Observation 10

Let *N* be a network and $Z\subseteq Y\subseteq V(N)$. Then, for every $y\in {{\,\textrm{LCA}\,}}(Y)$ there is $z\in {{\,\textrm{LCA}\,}}(Z)$ such that $z\preceq _N y$.

#### Proof

Consider $y\in {{\,\textrm{LCA}\,}}(Y)$. Then, *y* is also an ancestor of all vertices in *Z* and thus there is a $\preceq _N$-minimal descendant $z\preceq _N y$ that is an ancestor of all vertices in *Z*, i.e., $z\in {{\,\textrm{LCA}\,}}(Z)$. $\square$

If ${{\,\textrm{LCA}\,}}(Y) = \{u\}$ consists of a single element *u* only, we write ${{\,\textrm{lca}\,}}(Y)=u$. In other words, ${{\,\textrm{lca}\,}}(Y) = u$ always implies that the $\preceq _{N}$-minimal ancestor of the elements in *Y* exists and is uniquely determined. We leave ${{\,\textrm{lca}\,}}(Y)$ undefined for all *Y* with $\vert {{\,\textrm{LCA}\,}}(Y)\vert \ne 1$.

In Huber and Scholz (2018), least common ancestors *u* are defined in terms of the fact that no child of *u* is an ancestor of all vertices in *Y*. These definitions are equivalent:

#### Lemma 35

Let *N* be a network and $\emptyset \ne Y \subseteq V(N)$. Then, $u\in V(N)$ is a least common ancestor of *Y* if and only if *u* is an ancestor of all vertices in *Y* but there is no $v\in {{\,\textrm{child}\,}}_N(u)$ that is an ancestor of all vertices in *Y*.

#### Proof

By definition, $u\in V(N)$ is a least common ancestor of *Y* if it is ancestor of all vertices in *Y* and $\preceq _{N}$-minimal w.r.t. this property. Thus, the *only if*-part of the statement follows immediately. Conversely, suppose *u* is an ancestor of all vertices in *Y* but there is no $v\in {{\,\textrm{child}\,}}_N(u)$ with this property. Writing $D_x$ for the set of descendants of a vertex $x\in V(N)$, suppose conversely that *u* is an ancestor of all vertices in *Y* but $Y\not \subseteq D_v$ for each $v\in {{\,\textrm{child}\,}}_N(u)$. For every vertex $w\in V(N)$ with $w\prec _N u$, there is a directed path passing through some child $v\in {{\,\textrm{child}\,}}_N(u)$, i.e., $w\preceq _N v$. Hence, we have $D_w\subseteq D_v$. Together with $Y\not \subseteq D_v$, this implies $Y\not \subseteq D_w$. Hence, *u* is a least common ancestor of *Y*. $\square$

We will in particular be concerned here with LCAs of leaves, i.e., the sets ${{\,\textrm{LCA}\,}}(A)$ for non-empty subsets $A\subseteq X$. We can then express LCAs in terms of clusters:

#### Observation 11

$v\in {{\,\textrm{LCA}\,}}(A)$ if and only if $A\subseteq {{\,\mathrm{\texttt{C}}\,}}(v)$ and there is no vertex $u\prec _N v$ such that $A\subseteq {{\,\mathrm{\texttt{C}}\,}}(u)$.

Suppose ${{\,\textrm{lca}\,}}(A){=}{:}q$ is defined for some non-empty $A\subseteq X$. Then, by assumption, every vertex *v* with $A\subseteq {{\,\mathrm{\texttt{C}}\,}}(v)$ satisfies $q\preceq _N v$ and thus ${{\,\mathrm{\texttt{C}}\,}}(q)\subseteq {{\,\mathrm{\texttt{C}}\,}}(v)$ by Lemma 17. Since every vertex $v'$ for which ${{\,\mathrm{\texttt{C}}\,}}(q)\subseteq {{\,\mathrm{\texttt{C}}\,}}(v')$ in particular also satisfies $A\subseteq {{\,\mathrm{\texttt{C}}\,}}(v')$, we conclude that ${{\,\textrm{lca}\,}}({{\,\mathrm{\texttt{C}}\,}}(q))=q$. Thus, we have

#### Observation 12

Let *N* be a network, $\emptyset \ne A\subseteq X$, and suppose ${{\,\textrm{lca}\,}}(A)$ is defined. Then, the following is satisfied:

(i) ${{\,\textrm{lca}\,}}(A)\preceq _{N} v$ for all *v* with $A\subseteq {{\,\mathrm{\texttt{C}}\,}}(v)$.
(ii) ${{\,\mathrm{\texttt{C}}\,}}({{\,\textrm{lca}\,}}(A))$ is the unique inclusion-minimal cluster in $\mathscr {C}_N$ containing *A*.
(iii) $${{\,\textrm{lca}\,}}({{\,\mathrm{\texttt{C}}\,}}({{\,\textrm{lca}\,}}(A)))={{\,\textrm{lca}\,}}(A)$$.

In much of the literature on least common ancestors in DAGs, only pairwise LCAs are considered. Networks with unique pairwise LCAs are of interest because of a close connection with so-called binary clustering systems (Barthélemy and Brucker 2008) and monotone transit functions (Changat et al. 2019, 2022).

#### Definition 20

(Barthélemy and Brucker 2008) A clustering system $\mathscr {C}$ on *X* is *pre-binary* if, for every pair $x,y\in X$, there is a unique inclusion-minimal cluster *C* such that $\{x,y\}\subseteq C$.

From Observation 12(ii), we immediately obtain

#### Observation 13

If *N* is a network on *X* such that ${{\,\textrm{lca}\,}}(\{x,y\})$ is defined for all $x,y\in X$, then $\mathscr {C}_N$ is pre-binary.

**Fig. 13:**
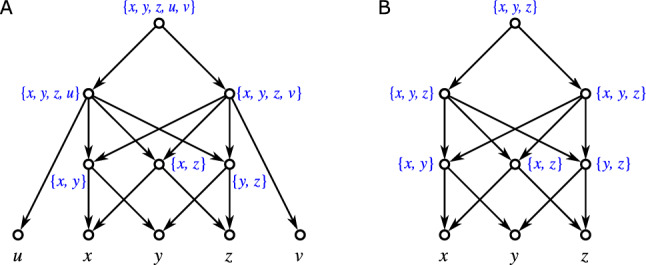
**A** A regular network that is not an lca-network even though all ${{\,\textrm{lca}\,}}(\{x,y\})$ is defined for all pairs. For $A=\{x,y,z\}$ both children of the root are contained in ${{\,\textrm{LCA}\,}}(A)$. Here, *A* is not a cluster. **B** The existence of pairwise LCAs is also insufficient to ensure that property (CL) is satisfied, i.e., that ${{\,\textrm{lca}\,}}({{\,\mathrm{\texttt{C}}\,}}(v))$ is defined for all $v\in V(N)$ since *A* is a cluster in this example

The first example in Fig. 13 shows, however, that unique pairwise LCAs are not sufficient to ensure that ${{\,\textrm{lca}\,}}(A)$ is also defined for larger sets. The second example shows that unique pairwise LCAs also do not ensure that all clusters ${{\,\mathrm{\texttt{C}}\,}}(v)$ have a unique LCA.

(Theorem 3.3 Willson 2010) showed that ${{\,\textrm{lca}\,}}({{\,\mathrm{\texttt{C}}\,}}(v))$ (there called “mrca”) is well defined for all vertices of a normal network. However, Willson (2010) uses a different definition of *X* as the “base set” comprising the root, leaves, and all vertices with outdegree 1. We therefore adapt Theorem 3.3 of Willson (2010) to our setting and include a proof for completeness.

#### Definition 21

A network *N* has the *cluster-lca* property (CL) if

(CL): For every $v\in V(N)$, ${{\,\textrm{lca}\,}}({{\,\mathrm{\texttt{C}}\,}}(v))$ is defined.

#### Lemma 36

Suppose *N* has property (CL). Then, for all $v\in V(N)$, it holds that ${{\,\textrm{lca}\,}}({{\,\mathrm{\texttt{C}}\,}}(v))\preceq _N v$ and ${{\,\mathrm{\texttt{C}}\,}}({{\,\textrm{lca}\,}}({{\,\mathrm{\texttt{C}}\,}}(v)))={{\,\mathrm{\texttt{C}}\,}}(v)$.

#### Proof

If $v\notin {{\,\textrm{LCA}\,}}({{\,\mathrm{\texttt{C}}\,}}(v)),$ then there is a descendant $v'\preceq v$ such that ${{\,\mathrm{\texttt{C}}\,}}(v)={{\,\mathrm{\texttt{C}}\,}}(v')$, and every $\preceq _N$-minimal descendant $v'$ with this property satisfies $v'\in {{\,\textrm{LCA}\,}}({{\,\mathrm{\texttt{C}}\,}}(v))$. By property (CL), ${{\,\textrm{LCA}\,}}({{\,\mathrm{\texttt{C}}\,}}(v))$ contains only a single vertex ${{\,\textrm{lca}\,}}({{\,\mathrm{\texttt{C}}\,}}(v))$, which therefore must coincide either with *v* or one of its descendants. The second statement now follows directly from the definition. $\square$

In Lemma 27, we saw that $Q(u){:}{=}\{u'\in V(N) \mid {{\,\mathrm{\texttt{C}}\,}}(u')={{\,\mathrm{\texttt{C}}\,}}(u)\}$ forms an induced path in semi-regular networks. Property (CL) imposes a weaker structure.

#### Lemma 37

Let *N* be a network satisfying (CL). Then, (i) *Q*(*u*) has a unique $\preceq _N$-minimal element, namely ${{\,\textrm{lca}\,}}({{\,\mathrm{\texttt{C}}\,}}(u))=\min Q(u)$, and (ii) if $u,v\in Q(u)$ and *w* is contained in a directed path from *u* to *v*, then $w\in Q(u)$.

#### Proof

The second statement in Lemma 36 implies that $q{:}{=}{{\,\textrm{lca}\,}}({{\,\mathrm{\texttt{C}}\,}}(u))\in Q(u)$. By definition *q* is the unique $\preceq _N$-minimal vertex that has all leaves in ${{\,\mathrm{\texttt{C}}\,}}(q)$ as its descendants and thus $q\preceq u'$ for all $u'\in Q(u)$, establishing statement (i). Statement (ii) is a direct consequence of Lemma 17. $\square$

**Fig. 14:**
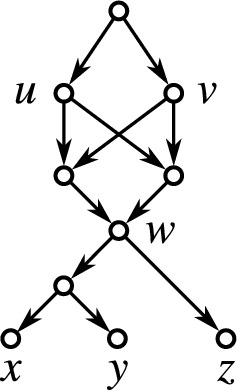
The network *N* satisfies (CL) but not (PCC). In addition to the singletons, only $\{x,y\}$ and $A=\{x,y,z\}$ appear as clusters. Both have unique last common ancestors, and hence, (CL) is satisfied. In particular, ${{\,\textrm{lca}\,}}(A)=w$. However, we have $A={{\,\mathrm{\texttt{C}}\,}}_{N}(u)={{\,\mathrm{\texttt{C}}\,}}_{N}(v)$ for the two $\preceq _{N}$-incomparable vertices *u* and *v*. Hence, *N* does not satisfy (PCC)

#### Lemma 38

If a network *N* satisfies (PCC), then it satisfies (CL).

#### Proof

Suppose that *N* satisfies (PCC). Given a cluster ${{\,\mathrm{\texttt{C}}\,}}(u)$, we consider the non-empty set $W{:}{=}\{w\in V \mid {{\,\mathrm{\texttt{C}}\,}}(u)={{\,\mathrm{\texttt{C}}\,}}(w)\}\subseteq V$. (PCC) implies that the elements of *W* are pairwise $\preceq _N$-comparable, and thus, there is a unique $\preceq _N$-minimal element $w\in W$. Furthermore, ${{\,\mathrm{\texttt{C}}\,}}(u)={{\,\mathrm{\texttt{C}}\,}}(w)\subsetneq {{\,\mathrm{\texttt{C}}\,}}(v)$ implies $w\prec _N v$ by Observation 7. Therefore, ${{\,\textrm{lca}\,}}({{\,\mathrm{\texttt{C}}\,}}(u))=w$. Since $u\in W$ by construction, we have $w\preceq _N u$, and thus, ${{\,\textrm{lca}\,}}({{\,\mathrm{\texttt{C}}\,}}(u))\preceq _N u$. $\square$

Figure 14 shows that the converse is not true, i.e., (CL) does not imply (PCC). Lemma 24 and Lemma 38 imply

#### Proposition 10

For every clustering system $\mathscr {C},$ there is a network *N* with $\mathscr {C}_N = \mathscr {C}$ that satisfies (CL).

Since a normal network is tree-child, it satisfies (PCC) by Corollary 13. This together with Lemma 38 implies

#### Corollary 17

Every normal network *N* satisfies (CL).

Property (CL), however, does not imply that ${{\,\textrm{lca}\,}}$ is well defined for all subsets $A\subseteq X$.

### LCA-networks

#### Definition 22

A network *N* is an *lca-network* if ${{\,\textrm{lca}\,}}(A)$ is well defined, i.e., if $\vert {{\,\textrm{LCA}\,}}(A)\vert =1$ for all non-empty subsets $A\subseteq X$.

From Observation 10 and uniqueness of the least common ancestors, we immediately obtain

#### Observation 14

Every lca-network satisfies (CL). Moreover, if *N* is an lca-network and $A\subseteq B \subseteq X$, then ${{\,\textrm{lca}\,}}(A)\preceq _N{{\,\textrm{lca}\,}}(B)$.

#### Corollary 18

Let *N* be an lca-network on *X* and $\emptyset \ne A\subseteq {{\,\mathrm{\texttt{C}}\,}}_{N}(v)$ for some $v\in V(N)$. Then, it holds ${{\,\textrm{lca}\,}}(A)\preceq _{N} v$.

#### Proof

Since $A \subseteq {{\,\mathrm{\texttt{C}}\,}}_N(v)\subseteq X$, Observation 14 implies that ${{\,\textrm{lca}\,}}(A) \preceq _N {{\,\textrm{lca}\,}}({{\,\mathrm{\texttt{C}}\,}}_N(v))$. Moreover, *N* satisfies (CL). Hence, we can apply Lemma 36 and conclude that ${{\,\textrm{lca}\,}}({{\,\mathrm{\texttt{C}}\,}}_N(v)) \preceq _N v$ and, therefore, ${{\,\textrm{lca}\,}}(A) \preceq _N v$. $\square$

#### Lemma 39

Every lca-network has a closed clustering system.

#### Proof

Let *N* be an lca-network. We show that $\mathscr {C}_N$ is closed, i.e., for all non-empty $A\in 2^X$, it holds ${{\,\textrm{cl}\,}}(A)=A \iff A\in \mathscr {C}_N$. From the definitions of clusters, and the closure operator, we obtain

$$\begin{aligned} A \subseteq \bigcap _{\begin{array}{c} v \in V\\ A\subseteq {{\,\mathrm{\texttt{C}}\,}}(v) \end{array}} {{\,\mathrm{\texttt{C}}\,}}(v) = \bigcap _{\begin{array}{c} C\in \mathscr {C}_N\\ A\subseteq C \end{array}} C = {{\,\textrm{cl}\,}}(A) . \end{aligned}$$

If $A\in \mathscr {C}_N$, then clearly ${{\,\textrm{cl}\,}}(A)=A$. Now, suppose $A\notin \mathscr {C}_N$ and assume, for contradiction, that ${{\,\textrm{cl}\,}}(A)=A$. Thus, we have ${{\,\textrm{cl}\,}}(A)\notin \mathscr {C}_N$. Then, there are at least two distinct inclusion-minimal clusters $C'$ and $C''$ such that $A\subsetneq C',C''$. Clearly, for every cluster $C\in \mathscr {C}_N$, there is a $\preceq _{N}$-minimal vertex *u* with $C={{\,\mathrm{\texttt{C}}\,}}(u)$. In particular, there are $\preceq _{N}$-minimal vertices $u'$ and $u''$ with $C'={{\,\mathrm{\texttt{C}}\,}}(u')$ and $C''={{\,\mathrm{\texttt{C}}\,}}(u'')$. Therefore and by Lemma 17, we obtain, for all $v\prec _{N} u'$, that ${{\,\mathrm{\texttt{C}}\,}}(v)\subsetneq {{\,\mathrm{\texttt{C}}\,}}(u')=C'$ and thus $A\not \subseteq {{\,\mathrm{\texttt{C}}\,}}(v)$ by inclusion-minimality of $C'$. Hence, $u'$ is a least common ancestor of *A*. By analogous arguments, $u''$ is a last common ancestor of *A*. Since $C'\ne C''$, $u'$ and $u''$ are distinct. Together with $\{u',u''\}\subseteq {{\,\textrm{LCA}\,}}(A)$, this contradicts that *N* is an lca-network. Therefore, $A\notin \mathscr {C}_N$ implies $A\subsetneq {{\,\textrm{cl}\,}}(A)$. Isotony of the closure function together with the contraposition of the latter statement shows that $A={{\,\textrm{cl}\,}}(A)\implies A\in \mathscr {C}_N$. In summary, therefore, $\mathscr {C}_N$ is closed. $\square$

#### Lemma 40

If *N* is a network with a closed clustering system $\mathscr {C}_N$ and *N* satisfies (PCC), then it is an lca-network.

#### Proof

Assume, for contradiction, that there is some set *A* with two distinct vertices $u,v\in {{\,\textrm{LCA}\,}}(A)$. Then, *u*, *v* are $\preceq _N$-incomparable and $A\subseteq {{\,\mathrm{\texttt{C}}\,}}(u)$ and $A\subseteq {{\,\mathrm{\texttt{C}}\,}}(v)$. If ${{\,\mathrm{\texttt{C}}\,}}(u)\subseteq {{\,\mathrm{\texttt{C}}\,}}(v)$, then (PCC) implies $u\preceq _N v$ or $v\preceq _N u$, a contradiction. Similarly, ${{\,\mathrm{\texttt{C}}\,}}(v)\subseteq {{\,\mathrm{\texttt{C}}\,}}(u)$ is not possible. Thus, ${{\,\mathrm{\texttt{C}}\,}}(u)$ and ${{\,\mathrm{\texttt{C}}\,}}(v)$ overlap. Since $\mathscr {C}_N$ is closed, there is a cluster ${{\,\mathrm{\texttt{C}}\,}}(w)={{\,\mathrm{\texttt{C}}\,}}(u)\cap {{\,\mathrm{\texttt{C}}\,}}(v)$ for some $w\in V$. Since $A\subseteq {{\,\mathrm{\texttt{C}}\,}}(w)\subsetneq {{\,\mathrm{\texttt{C}}\,}}(u)$ and $A\subseteq {{\,\mathrm{\texttt{C}}\,}}(w)\subsetneq {{\,\mathrm{\texttt{C}}\,}}(v)$, Observation 7 implies $w\prec _N u$ and $w\prec _N v$, contradicting $u,v\in {{\,\textrm{LCA}\,}}(A)$. $\square$

#### Theorem 7

Let *N* be a network satisfying (PCC). Then, *N* is an lca-network if and only if its clustering system $\mathscr {C}_N$ is closed.

#### Proof

Let *N* be an lca-network satisfying (PCC). By Lemma 39, $\mathscr {C}_N$ is closed. Conversely, by Lemma 40, a network satisfying (PCC) with a closed clustering system is an lca-network. $\square$

The example in Fig. 14 shows that (PCC) cannot be omitted in Theorem 7. A trivial consequence of Theorem 7 is

#### Corollary 19

A semi-regular network *N* is an lca-network if and only if its clustering system $\mathscr {C}_N$ is closed.

Since every tree-child network satisfies (PCC) we also obtain

#### Corollary 20

A tree-child network *N* is an lca-network if and only if its clustering system $\mathscr {C}_N$ is closed.

Moreover, we have

#### Proposition 11

A clustering system $\mathscr {C}$ is closed if and only if there is an lca-network *N* with $\mathscr {C}=\mathscr {C}_N$. In this case, the unique regular network and the unique cluster network of $\mathscr {C}$ are lca-networks.

#### Proof

Let $\mathscr {C}$ be the clustering system. By Proposition 2 and Theorem 6, there is a unique regular network *N* and a unique cluster network $N'$ with $\mathscr {C}=\mathscr {C}_N=\mathscr {C}_{N'}$. By Theorem 2 and 4, resp., both networks are semi-regular and thus satisfy (PCC). Suppose $\mathscr {C}$ is closed. Then, Theorem 7 implies that both *N* and $N'$ are lca-networks. In particular, there is an lca-network *N* with $\mathscr {C}=\mathscr {C}_N$. Conversely, suppose there is an lca-network *N* with $\mathscr {C}=\mathscr {C}_N$. By Lemma 39, this implies that $\mathscr {C}$ is closed. $\square$

As in the case of trees, there is a simple connection of the LCA with the closure operator:

#### Lemma 41

Let *N* be an lca-network with clustering system $\mathscr {C}$. Then, the following identity holds:

4 $$\begin{aligned} {{\,\mathrm{\texttt{C}}\,}}({{\,\textrm{lca}\,}}(Y))={{\,\textrm{cl}\,}}(Y) \quad \text { for all } \emptyset \ne Y\subseteq X. \end{aligned}$$

Furthermore, we have

5 $$\begin{aligned} {{\,\mathrm{\texttt{C}}\,}}({{\,\textrm{lca}\,}}(C))={{\,\textrm{cl}\,}}(C)=C \quad \text { for all } C\in \mathscr {C}. \end{aligned}$$

#### Proof

Let $\emptyset \ne Y\subseteq X$. By Proposition 11, $\mathscr {C}$ is closed and thus ${{\,\textrm{cl}\,}}(Y)\in \mathscr {C}$. In particular, ${{\,\textrm{cl}\,}}(Y)$ is the unique inclusion-minimal cluster in $\mathscr {C}$ containing *Y*. Let *u* be a vertex in *N* such that ${{\,\mathrm{\texttt{C}}\,}}(u)={{\,\textrm{cl}\,}}(Y)$. Since $Y\subseteq {{\,\textrm{cl}\,}}(Y)={{\,\mathrm{\texttt{C}}\,}}(u)$, Corollary 18 yields ${{\,\textrm{lca}\,}}(Y)\preceq _{N} u$. By Lemma 17, we have $Y\subseteq {{\,\mathrm{\texttt{C}}\,}}({{\,\textrm{lca}\,}}(Y))\subseteq {{\,\mathrm{\texttt{C}}\,}}(u)={{\,\textrm{cl}\,}}(Y)$. Since ${{\,\textrm{cl}\,}}(Y)$ is the unique inclusion-minimal cluster in $\mathscr {C}$ containing *Y*, this implies ${{\,\mathrm{\texttt{C}}\,}}({{\,\textrm{lca}\,}}(Y))={{\,\textrm{cl}\,}}(Y)$. For every cluster $C\in \mathscr {C}$, we have $C={{\,\textrm{cl}\,}}(C)$ by Eq. (2) and thus Eq. (5) follows immediately. $\square$

Next we show that two sets have the same LCA whenever their LCAs are associated with the same cluster.

#### Lemma 42

Let *N* be an lca-network on *X* and let $Y,Y'\subseteq X$. Then, (i) ${{\,\textrm{lca}\,}}({{\,\mathrm{\texttt{C}}\,}}({{\,\textrm{lca}\,}}(Y)))={{\,\textrm{lca}\,}}(Y)$ and (ii) ${{\,\mathrm{\texttt{C}}\,}}({{\,\textrm{lca}\,}}(Y))={{\,\mathrm{\texttt{C}}\,}}({{\,\textrm{lca}\,}}(Y'))$ imply ${{\,\textrm{lca}\,}}(Y)={{\,\textrm{lca}\,}}(Y')$

#### Proof

Since ${{\,\textrm{cl}\,}}$ is enlarging, i.e., $Y\subseteq {{\,\textrm{cl}\,}}(Y)$, we have $Y\subseteq {{\,\mathrm{\texttt{C}}\,}}({{\,\textrm{lca}\,}}(Y))$ by Lemma 41. Thus, Observation 14 implies ${{\,\textrm{lca}\,}}(Y)\preceq _N{{\,\textrm{lca}\,}}({{\,\mathrm{\texttt{C}}\,}}({{\,\textrm{lca}\,}}(Y)))$. On the other hand, all leaves in ${{\,\mathrm{\texttt{C}}\,}}({{\,\textrm{lca}\,}}(Y))$ are descendants of ${{\,\textrm{lca}\,}}(Y)$, and thus, ${{\,\textrm{lca}\,}}({{\,\mathrm{\texttt{C}}\,}}({{\,\textrm{lca}\,}}(Y)))\preceq _N {{\,\textrm{lca}\,}}(Y)$. Thus, statement (i) holds. Now, suppose ${{\,\mathrm{\texttt{C}}\,}}({{\,\textrm{lca}\,}}(Y))={{\,\mathrm{\texttt{C}}\,}}({{\,\textrm{lca}\,}}(Y'))$. Uniqueness of the LCA implies ${{\,\textrm{lca}\,}}({{\,\mathrm{\texttt{C}}\,}}({{\,\textrm{lca}\,}}(Y)))={{\,\textrm{lca}\,}}({{\,\mathrm{\texttt{C}}\,}}({{\,\textrm{lca}\,}}(Y')))$ and thus statement (i) implies ${{\,\textrm{lca}\,}}(Y)={{\,\textrm{lca}\,}}(Y')$. $\square$

### Strong LCA-networks and weak hierarchies

In this section, we consider an interesting subclass of lca-networks.

#### Definition 23

A network *N* on *X* is a *strong lca-network* if it is an lca-network and, for every non-empty subset $A\subseteq X$, there are $x,y\in A$ such that ${{\,\textrm{lca}\,}}(\{x,y\})={{\,\textrm{lca}\,}}(A)$.

**Fig. 15:**
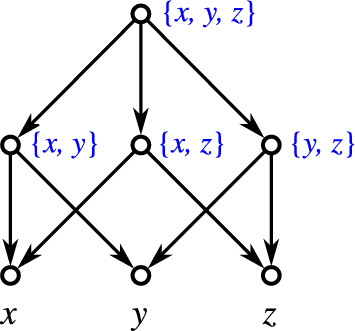
An lca-network with a subset of leaves $A{:}{=}\{x,y,z\}\subseteq X$ in which there are no $x',y'\in A$ such that ${{\,\textrm{lca}\,}}(\{x',y'\})={{\,\textrm{lca}\,}}(A)$

Figure 15 shows an lca-network that is not a strong lca-network.

We shall see below that strong lca-networks are intimately connected with well-studied types of clustering systems.

#### Definition 24

A clustering system $\mathscr {C}$ on *X* is

- a *weak hierarchy* if $C_1\cap C_2\cap C_3 \in \{C_1\cap C_2,C_1\cap C_3, C_2\cap C_3\}$ for all $C_1,C_2,C_3\in \mathscr {C}$; and
- *binary* if it is pre-binary and, for every $C\in \mathscr {C}$, there is a pair of vertices $x,y\in X$ such that *C* is the unique inclusion-minimal cluster containing *x* and *y*.

Weak hierarchies were introduced in Bandelt and Dress (1989) and subsequently have been studied in detail in the context of clustering systems, e.g., in Brucker and Gély (2009); Bertrand and Diatta (2014). Binary clustering system are considered systematically in Barthélemy and Brucker (2008). We first consider the lca-networks with binary clustering systems:

#### Lemma 43

Let *N* be an lca-network. Then, the following conditions are equivalent:

(i) For all $v\in N$, there is $x,y\in {{\,\mathrm{\texttt{C}}\,}}(v)$ such that ${{\,\textrm{lca}\,}}(\{x,y\})={{\,\textrm{lca}\,}}({{\,\mathrm{\texttt{C}}\,}}(v))$.
(ii) $\mathscr {C}_N$ is binary.

#### Proof

Since *N* is an lca-network, it is in particular pre-binary (cf. Observation 13) and satisfies (CL). Property (i) and Lemma 36 imply ${{\,\mathrm{\texttt{C}}\,}}(v)={{\,\mathrm{\texttt{C}}\,}}({{\,\textrm{lca}\,}}({{\,\mathrm{\texttt{C}}\,}}(v)))={{\,\mathrm{\texttt{C}}\,}}({{\,\textrm{lca}\,}}(\{x,y\}))$ for two vertices $x,y\in {{\,\mathrm{\texttt{C}}\,}}(v)$. By Observation 12(ii), ${{\,\mathrm{\texttt{C}}\,}}(v)$ is the unique inclusion-minimal cluster containing *x* and *y*, i.e., $\mathscr {C}_N$ is binary. Conversely, suppose *N* is an lca-network with a binary clustering system. Then for every $v\in N$, there is $x,y\in X$ such that ${{\,\mathrm{\texttt{C}}\,}}(v)$ is the unique inclusion-minimal cluster that contains *x* and *y*. By Observation 12(ii), this implies ${{\,\mathrm{\texttt{C}}\,}}(v)={{\,\mathrm{\texttt{C}}\,}}({{\,\textrm{lca}\,}}(\{x,y\}))$. Hence, we have ${{\,\textrm{lca}\,}}({{\,\mathrm{\texttt{C}}\,}}(v))={{\,\textrm{lca}\,}}({{\,\mathrm{\texttt{C}}\,}}({{\,\textrm{lca}\,}}(\{x,y\})))$, and thus, by Observation 12(iii), ${{\,\textrm{lca}\,}}({{\,\mathrm{\texttt{C}}\,}}(v))={{\,\textrm{lca}\,}}({{\,\mathrm{\texttt{C}}\,}}({{\,\textrm{lca}\,}}(\{x,y\})))={{\,\textrm{lca}\,}}(\{x,y\})$, i.e., property (i) holds. $\square$

In particular, therefore, strong lca-networks give rise to binary clustering systems:

#### Corollary 21

The clustering system of a strong lca-network is binary.

The converse is not true in general, since condition (i) in Lemma 43 requires only that the LCAs of clusters but not necessarily the LCAs of all sets are determined by the LCA of a leaf pair. The latter, stronger condition, is related to weak hierarchies. To investigate this connection, we recall

#### Proposition 12

(Lemma 1 Bandelt and Dress 1989) A clustering system $\mathscr {C}$ on *X* is a weak hierarchy if and only if for every non-empty subset $A\subseteq X$ there exist $x,y\in A$ such that ${{\,\textrm{cl}\,}}(A)={{\,\textrm{cl}\,}}(\{x,y\})$.

#### Proposition 13

Let *N* be an lca-network on *N*. Then, *N* is a strong lca-network if and only of $\mathscr {C}_N$ is a weak hierarchy.

#### Proof

Definition 23 and Eq. (4) imply that for every $\emptyset \ne A\subseteq X$ there is $x,y\in A$ such that ${{\,\textrm{cl}\,}}(A)={{\,\mathrm{\texttt{C}}\,}}({{\,\textrm{lca}\,}}(A))={{\,\mathrm{\texttt{C}}\,}}({{\,\textrm{lca}\,}}(\{x,y\}))={{\,\textrm{cl}\,}}(\{x,y\})$, and thus, $\mathscr {C}_N$ is a weak hierarchy by Proposition 12. Conversely, if *N* is an lca-network such that $\mathscr {C}_N$ is a weak hierarchy, then for all $\emptyset \ne A\subseteq X$ there is $x,y\in A$ such that ${{\,\mathrm{\texttt{C}}\,}}({{\,\textrm{lca}\,}}(A))={{\,\mathrm{\texttt{C}}\,}}({{\,\textrm{lca}\,}}(\{x,y\}))$. By Observation 12 we have ${{\,\textrm{lca}\,}}(A)={{\,\textrm{lca}\,}}({{\,\mathrm{\texttt{C}}\,}}({{\,\textrm{lca}\,}}(A)))={{\,\textrm{lca}\,}}({{\,\mathrm{\texttt{C}}\,}}({{\,\textrm{lca}\,}}(\{x,y\})))={{\,\textrm{lca}\,}}(\{x,y\})$, and thus, *N* is a strong lca-network. $\square$

#### Corollary 22

Let *N* be a network satisfying (PCC). Then, *N* is a strong lca-network if and only if $\mathscr {C}_N$ is a closed weak hierarchy.

#### Proof

Let *N* be a network satisfying (PCC). By Theorem 7, *N* is an lca-network if and only if $\mathscr {C}_N$ is a closed. By Proposition 13, *N* is a strong lca-network precisely if and only if $\mathscr {C}_N$ is a weak hierarchy. $\square$

Furthermore, we can use the same arguments in the proof of Proposition 11 together with Corollary 22 to derive the final result of this section:

#### Proposition 14

A clustering system $\mathscr {C}$ is a closed weak hierarchy if and only if it is the clustering system of a strong lca-network. In this case, the unique regular network and the unique cluster network of $\mathscr {C}$ are strong lca-networks.

## Level-1 networks

### Basic properties

We start by showing that all phylogenetic level-1 networks have the path-cluster-comparability property (PCC).

#### Lemma 44

Every phylogenetic level-1 network satisfies (PCC).

#### Proof

If *N* is a phylogenetic level-1 network, then it is tree-child by Proposition 9, and in turn every phylogenetic tree-child network satisfies (PCC) by Corollary 13. $\square$

We note that “phylogenetic” cannot be dropped in Lemma 44. To see this, consider the level-1 network *N* in Fig. 17B in “Level-1 networks encoded by their cluster multisets” section. There, both parents of the hybrid vertex correspond to cluster $\{a\}$ but they are $\preceq _{N}$-incomparable; a violation of (PCC).

#### Corollary 23

Let *N* be a level-1 network. Then, *N* is least-resolved if and only if *N* is regular.

#### Proof

By Corollary 2 and Theorem 2, resp., least-resolved and regular networks do not contain vertices with outdegree 1, and thus, they are phylogenetic. The statement now follows immediately from Lemma 44 and Theorem 3. $\square$

We emphasize, however, that there can exist least-resolved networks *N* for a given clustering system $\mathscr {C}$ that are not regular, as the example in Fig. 8 shows. In this example, the regular network $N'$ is level-1. Next we show that Lemma 15 does not hold for level-1 networks:

#### Lemma 45

Let *n* be a positive integer. Then, there is no phylogenetic, shortcut-free level-1 network *N* on *n* leaves that is not a tree and where $\mathscr {C}_N$ is a hierarchy.

#### Proof

Let *N* be a phylogenetic, shortcut-free level-1 that is not a tree. By Lemma 44, *N* satisfies (PCC). Since, in addition, *N* is shortcut-free, *N* is semi-regular. Since *N* is not a tree, it must contain a non-trivial block *B*. By Lemma 28, there are at least two vertices *u* and *v* such that ${{\,\mathrm{\texttt{C}}\,}}(u)$ and ${{\,\mathrm{\texttt{C}}\,}}(v)$ overlap. Hence, $\mathscr {C}_N$ is not a hierarchy. $\square$

As an immediate consequence of Theorem 2 and Lemma 44, we also obtain the following

#### Proposition 15

A phylogenetic level-1 network is semi-regular if and only if it is shortcut-free. Furthermore, a level-1 network is regular if and only if it is shortcut-free and has no vertex with outdegree 1.

#### Lemma 46

Let *N* be a phylogenetic level-1 network and *v* be a hybrid vertex of *N*. Then, ${{\,\mathrm{\texttt{C}}\,}}(v)\subsetneq {{\,\mathrm{\texttt{C}}\,}}(u)$ for every $u\in V(N)$ with $v\prec _N u$.

#### Proof

Let *N* be a phylogenetic level-1 network and *v* be a hybrid vertex of *N*. By Lemma 10, *v* and all of its (at least two) parents are contained in a common non-trivial block *B*. Hence, consider first one of the parents $w_1$ of *v* such that $w_1\ne \max B$. By Lemma 17, ${{\,\mathrm{\texttt{C}}\,}}(v)\subseteq {{\,\mathrm{\texttt{C}}\,}}(w_1)$. Assume, for contradiction, that ${{\,\mathrm{\texttt{C}}\,}}(v) = {{\,\mathrm{\texttt{C}}\,}}(w_1)$. Since *v* and $w_1$ are contained in the same non-trivial block *B* and *N* is level-1, $w_1$ cannot be a hybrid vertex and thus, since *N* is phylogenetic, we have ${{\,\textrm{outdeg}\,}}_N(w_1)\ge 2$. Let $w'\ne v$ be another child of $w_1$. Again, by Lemma 17, ${{\,\mathrm{\texttt{C}}\,}}(w')\subseteq {{\,\mathrm{\texttt{C}}\,}}(w_1)$ and thus, ${{\,\mathrm{\texttt{C}}\,}}(w')\subseteq {{\,\mathrm{\texttt{C}}\,}}(v)$. Lemma 44 implies that *N* satisfies (PCC), and thus, *v* and $w'$ are $\preceq _N$-comparable. Hence, we distinguish the two cases (a) $w'\prec _N v$ and (b) $v\prec _{N} w'$.

In Case (a), the arc $(w_1,w')$ must be a shortcut since $w'\prec _N v$ and $v\in {{\,\textrm{child}\,}}_{N}(w_1){\setminus }\{w'\}$. In particular, $w'$ must be a hybrid vertex and there is a directed path from $w_1$ to $w'$ passing through *v*, which together with the arc $(w_1,w')$ forms an undirected cycle. Hence, $w_1$, *v*, and $w'$ are contained in a common block that shares the arc $(w_1,v)$ with *B* and thus equals *B*. But then *B* contains two hybrid vertices *v* and $w'$ that are distinct from $\max B$, a contradiction.

Now, consider Case (b), i.e., $v\prec _{N} w' \prec _{N} w_1$. In this case, *v* has a parent $w_2$ such that $v\prec _N w_2\prec _{N} w_1 \prec _N\max B$. In particular, $w_2$ lies in *B* and Lemma 17 implies ${{\,\mathrm{\texttt{C}}\,}}(v)\subseteq {{\,\mathrm{\texttt{C}}\,}}(w_2) \subseteq {{\,\mathrm{\texttt{C}}\,}}(w_1)$ and thus ${{\,\mathrm{\texttt{C}}\,}}(v)= {{\,\mathrm{\texttt{C}}\,}}(w_2)$. Now, we can repeat the latter arguments for parent $w_2$ and eventually encounter a contradiction as in Case (a) or, if we never obtain such a contradiction, we end in an infinite chain of vertices $w_1\succ _{N} w_2 \succ _{N} \dots$, a contradiction to *V*(*N*) being finite. The latter together with the fact that $w_1\ne \max B$ was chosen arbitrarily implies that ${{\,\mathrm{\texttt{C}}\,}}(v)\subsetneq {{\,\mathrm{\texttt{C}}\,}}(w)$ for every parent *w* of *v* that is distinct from $\max B$. Now, suppose that $w=\max B$ is a parent of *v*. Since *v* is a hybrid vertex, it has another parent $w'$, which is also contained in *B* and satisfies $v\prec _{N} w'\prec _{N} w$. Lemma 17 and the arguments above thus yield ${{\,\mathrm{\texttt{C}}\,}}(v)\subsetneq {{\,\mathrm{\texttt{C}}\,}}(w')\subseteq {{\,\mathrm{\texttt{C}}\,}}(w)$.

Finally note that $v\prec _N u$ if and only if $v\prec _N w\preceq _N u$ where *w* is a parent of *v*. This together with Lemma 17 implies that ${{\,\mathrm{\texttt{C}}\,}}(v)\subsetneq {{\,\mathrm{\texttt{C}}\,}}(w)\subseteq {{\,\mathrm{\texttt{C}}\,}}(u)$ for all $u\in V(N)$ with $v\prec _N w\preceq _N u$ and where *w* is a parent of *v*. $\square$

#### Lemma 47

Let *N* be a level-1 network. Then, every block *B* has a unique $\preceq _N$-minimal vertex $\min B$ and a unique $\preceq _N$-maximal vertex $\max B$. In case *B* is not a single vertex or arc, $\min B$ is the unique properly contained hybrid vertex in *B* and $\max B$ is the unique root of *B*.

#### Proof

The statement is trivial for a block that consists only of a single vertex or arc. Uniqueness of the $\preceq _N$-maximal vertex in *B* follows from Lemma 8. Otherwise, every $v\in V(B)$ lies on an undirected cycle. Since *B* is acyclic, a $\preceq _N$-minimal vertex *u* in *B* does not have an out-neighbor along the cycle, and therefore, *u* has at least two in-neighbors that are contained in *B*. Thus, *u* is a hybrid vertex and, by Lemma 10, *u* is properly contained in *B*. By definition of level-1, there is at most one such vertex in *B*. $\square$

As an immediate consequence, we have

#### Corollary 24

Let *N* be a level-1 network and *B* a block of *N*. For every $v\in B$, it holds $\min B \preceq _N v \preceq _N \max B$ and ${{\,\mathrm{\texttt{C}}\,}}(\min B)\subseteq {{\,\mathrm{\texttt{C}}\,}}(v)\subseteq {{\,\mathrm{\texttt{C}}\,}}(\max B)$.

The fact that every block in a level-1 contains at most one hybrid vertex, implies that $B^0 = B\setminus \{\min B,\max B\}$ for every block *B* (cf. Definition 8). Recall that a block is non-trivial if it is not a single vertex or a single arc. Hence, a block *B* is non-trivial precisely if $B^0\ne \emptyset$. In the absence of shortcuts and in case *B* is non-trivial, the subnetwork induced by $B^0$ is a forest consisting of at least two non-empty trees.

#### Lemma 48

Let $N=(V,E)$ be a level-1 network and suppose $u,v\in V$ are $\preceq _N$-incomparable. Then, *u* and *v* are located in a common block *B* of *N* if and only if ${{\,\mathrm{\texttt{C}}\,}}(u)\cap {{\,\mathrm{\texttt{C}}\,}}(v)\ne \emptyset$. In particular, they share exactly the descendants of the $\preceq _N$-minimal element $\min B$ of *B*, i.e., in this case we have ${{\,\mathrm{\texttt{C}}\,}}(u)\cap {{\,\mathrm{\texttt{C}}\,}}(v)={{\,\mathrm{\texttt{C}}\,}}(\min B)$.

#### Proof

If *u* and *v* are both located in block *B*, then Corollary 24 implies $\emptyset \ne {{\,\mathrm{\texttt{C}}\,}}(\min B)\subseteq {{\,\mathrm{\texttt{C}}\,}}(u)\cap {{\,\mathrm{\texttt{C}}\,}}(v)$. Conversely, if ${{\,\mathrm{\texttt{C}}\,}}(u)\cap {{\,\mathrm{\texttt{C}}\,}}(v)\ne \emptyset$, then Lemma 18 implies that, for every $x\in {{\,\mathrm{\texttt{C}}\,}}(u)\cap {{\,\mathrm{\texttt{C}}\,}}(v)$, *u* and *v* are contained in a common block *B*, and *B* contains, in addition, a hybrid vertex *w* such that $w\prec _N u,v$ and $x\in {{\,\mathrm{\texttt{C}}\,}}(w)$. Since *N* is level-1 and $w\ne \max B$, we have $w=\min B$. Moreover, for all $x\in {{\,\mathrm{\texttt{C}}\,}}(u)\cap {{\,\mathrm{\texttt{C}}\,}}(v)$, the corresponding blocks *B* share *u* and *v* and are therefore identical by Observation 1. Hence, we obtain ${{\,\mathrm{\texttt{C}}\,}}(u)\cap {{\,\mathrm{\texttt{C}}\,}}(v) \subseteq {{\,\mathrm{\texttt{C}}\,}}(\min B)$ and thus ${{\,\mathrm{\texttt{C}}\,}}(u)\cap {{\,\mathrm{\texttt{C}}\,}}(v) = {{\,\mathrm{\texttt{C}}\,}}(\min B)$. $\square$

### Clusters and least common ancestors

An important property of level-1 networks that is not true in general phylogenetic networks is the following.

#### Lemma 49

Every level-1 network is an lca-network.

#### Proof

Let *N* be a level-1 network on *X* and $\emptyset \ne Y\subseteq X$. Suppose for contradiction that there are two distinct such vertices *u* and $u'$ for which $Y\subseteq {{\,\mathrm{\texttt{C}}\,}}(u), {{\,\mathrm{\texttt{C}}\,}}(u')$ and such that $Y\not \subseteq {{\,\mathrm{\texttt{C}}\,}}(v)$ whenever $v\prec _N u,u'$. Clearly, *u* and $u'$ must be $\preceq _N$-incomparable. From Lemma 48 and $\emptyset \ne Y\subseteq {{\,\mathrm{\texttt{C}}\,}}(u)\cap {{\,\mathrm{\texttt{C}}\,}}(u')$, we obtain that *u* and $u'$ are located in the same block *B* and ${{\,\mathrm{\texttt{C}}\,}}(u)\cap {{\,\mathrm{\texttt{C}}\,}}(u')={{\,\mathrm{\texttt{C}}\,}}(\min B)$. In particular, therefore, $Y\subseteq {{\,\mathrm{\texttt{C}}\,}}(\min B)$. Since $\min B\preceq _T u,u'$ by Corollary 24 and *u* and $u'$ are $\preceq _N$-incomparable, we have $u\ne \min B$ and $u'\ne \min B$, and thus, $\min B\prec _N u,u'$, a contradiction to $Y\not \subseteq {{\,\mathrm{\texttt{C}}\,}}(v)$ for all $v\prec _N u,u'$. Therefore, the least common ancestor is unique and ${{\,\textrm{lca}\,}}(u)$ is well defined for all $\emptyset \ne Y\subseteq X$. $\square$

By Lemma 49, every leaf set in a level-1 network *N* has a unique LCA. As a further consequence of Lemmas 35 and 49, the following result, which was stated without proof in Huber and Scholz (2018) for binary level-1 networks, also holds in our more general setting:

#### Corollary 25

Let *N* be a level-1 network on *X* and $\emptyset \ne Y\subseteq X$. Then, there is a unique vertex *u* such that $Y\subseteq {{\,\mathrm{\texttt{C}}\,}}(u)$ but $Y\not \subseteq {{\,\mathrm{\texttt{C}}\,}}(v)$ for all $v\in {{\,\textrm{child}\,}}(u)$. In this case, $u = {{\,\textrm{lca}\,}}(Y)$.

Proposition 1 of Huber and Scholz (2018) also states the following result (without proof) for binary level-1 networks:

#### Lemma 50

Every level-1 network is a strong lca-network.

#### Proof

Let *N* be a level-1 network. By Lemma 49, *N* is an lca-network. Thus, it remains to show that, for every $\emptyset \ne Y\subseteq X$, there are leaves $x,y\in X$ such that ${{\,\textrm{lca}\,}}(Y)={{\,\textrm{lca}\,}}(\{x,y\})$. The statement holds trivially if $Y=\{x\}$, since then ${{\,\textrm{lca}\,}}(\{x,x\})={{\,\textrm{lca}\,}}(Y)$. Hence, suppose now that $\vert Y\vert \ge 2$ and thus that $v{:}{=}{{\,\textrm{lca}\,}}(Y)$ is not a leaf. By Corollary 25, every child $v'\in {{\,\textrm{child}\,}}(v)$ satisfies $Y\not \subseteq {{\,\mathrm{\texttt{C}}\,}}(v')$ and $Y\subseteq {{\,\mathrm{\texttt{C}}\,}}(v)=\bigcup _{v'\in {{\,\textrm{child}\,}}(v)}{{\,\mathrm{\texttt{C}}\,}}(v')$, there are two distinct children $v',v''\in {{\,\textrm{child}\,}}(v)$ such that there is $x\in Y\cap {{\,\mathrm{\texttt{C}}\,}}(v'){\setminus } {{\,\mathrm{\texttt{C}}\,}}(v'')\ne \emptyset$ and $y\in Y\cap {{\,\mathrm{\texttt{C}}\,}}(v''){\setminus } {{\,\mathrm{\texttt{C}}\,}}(v')\ne \emptyset$. Since $\{x,y\}\subseteq Y$, we have ${{\,\textrm{lca}\,}}(\{x,y\})\preceq _N v$ by Lemma 49 and Observation 14. Suppose for contradiction that ${{\,\textrm{lca}\,}}(\{x,y\})\prec _N v$. Contraposition of Corollary 25 and $\{x,y\}\subseteq {{\,\mathrm{\texttt{C}}\,}}(v)$ implies that there is a child $v'''\in {{\,\textrm{child}\,}}(v)$ with $\{x,y\} \subseteq {{\,\mathrm{\texttt{C}}\,}}(v''')$. By the choice of $v'$ and $v'',$ we have $v'''\notin \{v',v''\}$.

We continue by showing that *v*, $v'$, and $v'''$ are located in a common block *B* of *N*. Consider first the case that $v'$ and $v'''$ are $\preceq _N$-comparable. Then, Lemma 17, $y\in {{\,\mathrm{\texttt{C}}\,}}(v''')$, and $y\notin {{\,\mathrm{\texttt{C}}\,}}(v')$ imply $v'\prec _N v'''$. Hence, the three vertices $v,v'$ and $v'''$ lie on an undirected circle formed by the arcs $(v,v')$ and $(v,v''')$ and a directed path from $v'''$ to $v'$. By Observation 1, the vertices *v*, $v'$, and $v'''$ are part of a common block *B*. Assume now that $v'$ and $v'''$ are $\preceq _N$-incomparable, then $x\in {{\,\mathrm{\texttt{C}}\,}}(v')\cap {{\,\mathrm{\texttt{C}}\,}}(v''')$ and Lemma 48 implies that $v'$ and $v'''$ are contained in common non-trivial block *B*. If *v* is not contained in *B*, then *v* and arcs $(v,v')$ and $(v,v''')$ can be added to *B* without losing biconnectivity; contradicting that *B* is a maximal biconnected subgraph. Hence, *v* is also contained in *B*. Similarly, one shows that *v*, $v''$, and $v'''$ are located in a common block $B'$ of *N*. Since *B* and $B'$ share the arc $(v,v''')$, Observation 2 implies $B=B'$. In summary, *v*, $v'$, $v''$ and $v'''$ are all located in a common block *B* of *N*.

Now, suppose again that $v'$ and $v'''$ are $\preceq _N$-comparable. As argued above, we have $v'\prec _N v'''$ and thus there is a directed path *P* from $v'''$ to $v'$. Since *N* is acyclic and $(v,v''')\in E(N)$, all vertices *w* in *P* satisfy $w\prec _N v$. Together with $(v,v')\in E(N)$, this implies that $v'$ has at least indegree 2 and thus, $v'$ is a hybrid vertex of *B*. Since the hybrid vertex in each block of a level-1 network is unique, we have $v'=\min B$. But then we have $x\in {{\,\mathrm{\texttt{C}}\,}}(v')={{\,\mathrm{\texttt{C}}\,}}(\min B)\subseteq {{\,\mathrm{\texttt{C}}\,}}(v'')$ by Corollary 24, a contradiction. Hence, $v'$ and $v'''$ must be $\preceq _N$-incomparable and we can apply Lemma 48 to conclude that ${{\,\mathrm{\texttt{C}}\,}}(v')\cap {{\,\mathrm{\texttt{C}}\,}}(v''')={{\,\mathrm{\texttt{C}}\,}}(\min B)$ and thus $x\in {{\,\mathrm{\texttt{C}}\,}}(\min B)$. Corollary 24 therefore implies $x\in {{\,\mathrm{\texttt{C}}\,}}(\min B)\subseteq {{\,\mathrm{\texttt{C}}\,}}(v'')$, a contradiction. In summary, the case ${{\,\textrm{lca}\,}}(\{x,y\})\prec _N v$ is not possible and hence we must have ${{\,\textrm{lca}\,}}(\{x,y\})= v$. $\square$

As an immediate consequence of Lemma 50 (or alternatively Lemmas 17 and 48) and Proposition 14, we have:

#### Corollary 26

The clustering system of a level-1 network is a closed weak hierarchy.

This result also generalizes (Proposition 1 Gambette and Huber 2012), who showed that $\mathscr {C}_N$ is a weak hierarchy for binary level-1 networks. The next two results provide some more detailed insights into the structure of last common ancestors in level-1 networks.

#### Lemma 51

Let *N* be a shortcut-free phylogenetic level-1 network on *X* and $v\in V(N)$ be a vertex with outdegree at least 2. Then, $v={{\,\textrm{lca}\,}}({{\,\mathrm{\texttt{C}}\,}}_{N}(v))$ and there are two leaves $x,y\in X$ such that ${{\,\textrm{lca}\,}}(x,y) = v$. Moreover, if ${{\,\mathrm{\texttt{C}}\,}}_{N}(u)\cap {{\,\mathrm{\texttt{C}}\,}}_{N}(w)=\emptyset$ for two children $u,w\in {{\,\textrm{child}\,}}_{N}(v)$, then ${{\,\textrm{lca}\,}}(x,y)=v$ for all $x\in {{\,\mathrm{\texttt{C}}\,}}_{N}(u)$ and $y\in {{\,\mathrm{\texttt{C}}\,}}_{N}(w)$.

#### Proof

Suppose that *N* is a shortcut-free phylogenetic level-1 network on *X* and that $v\in V(N)$ is a vertex with outdegree at least 2. Put $Y{:}{=}{{\,\mathrm{\texttt{C}}\,}}_{N}(v)$. By Lemma 44, *N* satisfies (PCC). Thus, it is semi-regular, which allows us to use Corollary 10 and to conclude that ${{\,\mathrm{\texttt{C}}\,}}_{N}(u)\subsetneq Y$ for all children *u* of *v*. Hence, $u\ne {{\,\textrm{lca}\,}}(Y)$ for all children *u* of *v*. Moreover, by Corollary 25, there is a unique vertex $w\in V(N)$ such that $Y \subseteq {{\,\mathrm{\texttt{C}}\,}}_{N}(w)$ but $Y \not \subseteq {{\,\mathrm{\texttt{C}}\,}}_{N}(w')$ for all children $w'$ of *w* in which case, $w={{\,\textrm{lca}\,}}(Y)$. Taking the latter two arguments together yields $v={{\,\textrm{lca}\,}}(Y)$. Moreover, *N* is strong lca-network by Lemma 50. Hence, there are two leaves $x,y\in Y\subseteq X$ such that ${{\,\textrm{lca}\,}}(x,y) = {{\,\textrm{lca}\,}}(Y) = v$.

Now, suppose ${{\,\mathrm{\texttt{C}}\,}}_{N}(u)\cap {{\,\mathrm{\texttt{C}}\,}}_{N}(w)=\emptyset$ for two children $u,w\in {{\,\textrm{child}\,}}_{N}(v)$ and let $x\in {{\,\mathrm{\texttt{C}}\,}}_{N}(u)$ and $y\in {{\,\mathrm{\texttt{C}}\,}}_{N}(w)$. Thus, we have $\{x,y\}\not \subseteq {{\,\mathrm{\texttt{C}}\,}}_{N}(u), {{\,\mathrm{\texttt{C}}\,}}_{N}(w)$ and $\{x,y\}\subseteq {{\,\mathrm{\texttt{C}}\,}}_{N}(v)$. Now, suppose *v* has a child $u'\notin \{u,w\}$ such that $\{x,y\}\subseteq {{\,\mathrm{\texttt{C}}\,}}_{N}(u')$. Since *N* is shortcut-free, *u*, *w*, and $u'$ are pairwise $\preceq _{N}$-incomparable by Observation 3. Now, $x\in {{\,\mathrm{\texttt{C}}\,}}_N(u)\cap {{\,\mathrm{\texttt{C}}\,}}_{N}(u')$ and Lemma 48 imply that *u* and $u'$ are located in a common block *B* and $x\in {{\,\mathrm{\texttt{C}}\,}}_{N}(\min B)={{\,\mathrm{\texttt{C}}\,}}_{N}(u)\cap {{\,\mathrm{\texttt{C}}\,}}_{N}(u')$. In particular, $u'\ne \max B$ since *u* and $u'$ are $\preceq _{N}$-incomparable. By similar arguments, *w* and $u'$ are located in a common block $B'$ with $y\in {{\,\mathrm{\texttt{C}}\,}}_{N}(\min B')={{\,\mathrm{\texttt{C}}\,}}_N(w)\cap {{\,\mathrm{\texttt{C}}\,}}_{N}(u')$ and $u'\ne \max B'$. Using Lemma 9, we conclude that $B=B'$ and thus $x,y\in {{\,\mathrm{\texttt{C}}\,}}_{N}(\min B)\subseteq {{\,\mathrm{\texttt{C}}\,}}_{N}(u)$, a contradiction. Hence, *v* does have a child $u'$ such that $\{x,y\}\subseteq {{\,\mathrm{\texttt{C}}\,}}_{N}(u')$. Therefore, *v* is the unique least common ancestor of $\{x,y\}$. $\square$

#### Lemma 52

Let *N* be a shortcut-free level-1 network on *X* and $v\in V(N)$. Then, ${{\,\mathrm{\texttt{C}}\,}}_{N}(u)\cap {{\,\mathrm{\texttt{C}}\,}}_{N}(w)\ne \emptyset$ for two distinct children *u*, *w* of *v* if and only if *v* is the $\preceq _{N}$-maximal vertex of a cycle in *N*.

#### Proof

Suppose ${{\,\mathrm{\texttt{C}}\,}}_{N}(u)\cap {{\,\mathrm{\texttt{C}}\,}}_{N}(w)\ne \emptyset$ for two distinct children *u*, *w* of *v*. Since *N* is shortcut-free, *u* and *w* must be $\preceq _N$-incomparable by Observation 3. Together with ${{\,\mathrm{\texttt{C}}\,}}_{N}(u)\cap {{\,\mathrm{\texttt{C}}\,}}_{N}(w)\ne \emptyset$ and Lemma 18, this implies that *u* and *w* are connected by an undirected path *P* whose inner vertices *x* satisfy $x\prec _N u$ or $x\prec _N w$. Thus, *P* and the two arcs (*v*, *u*) and (*v*, *w*) form an undirected cycle whose $\preceq _{N}$-maximal vertex is *v*. Conversely, suppose *v* is the $\preceq _{N}$-maximal vertex of a cycle *K* in *N*. Hence, the two vertices $u'$ and $w'$ that are incident with *v* in *K* are children of *v*. In particular, *v*, $u'$, and $w'$ are contained in a common block. Again, $u'$ and $w'$ must be $\preceq _N$-incomparable since *N* is shortcut-free. Therefore, we can apply Lemma 48 to conclude that ${{\,\mathrm{\texttt{C}}\,}}_{N}(u)\cap {{\,\mathrm{\texttt{C}}\,}}_{N}(w)\ne \emptyset$. $\square$

In many applications, vertex- or arc-labeled networks are considered as a scaffold to explain genomic sequence data (Huber and Moulton 2006; Huber et al. 2019a; Huber and Scholz 2018; Hellmuth et al. 2015; Hellmuth and Wieseke 2016; Hellmuth and Scholz 2021; Hellmuth et al. 2019; Bruckmann et al. 2022). In this context, it is of considerable interest to understand the structure of least-resolved networks that still explain the same data and are obtained from the original network by shortcut removal and contraction of arcs (cf. Definition 6). Hence, it is important to keep track of ${{\,\textrm{lca}\,}}$’s after arcs have been

contracted. To this end, we provide the following

#### Proposition 16

Let *N* be a level-1 network with leaf set *X* and $(v',v)$ be an arc such that *v* is neither a hybrid vertex nor a leaf of *N*. Moreover, let $N'$ be the network obtained from *N* by application of ${{\,\mathrm{\textsc {cntr}}\,}}(v',v)$. Then, for all $x,y\in X$, we have ${{\,\textrm{lca}\,}}_{N'}(x,y) = {{\,\textrm{lca}\,}}_N(x,y)$ whenever ${{\,\textrm{lca}\,}}_N(x,y)\ne v'$ and, otherwise, ${{\,\textrm{lca}\,}}_{N'}(x,y) =v$.

#### Proof

Since *v* is not a hybrid vertex, $e = (v',v)$ is not a shortcut. Thus, ${{\,\mathrm{\textsc {cntr}}\,}}(v',v)$ is well defined. Let $x, y \in X$. If $x=y$, then ${{\,\textrm{lca}\,}}_N(x,y) = x = {{\,\textrm{lca}\,}}_{N'}(x,y)$. Hence, assume that $x\ne y$. Note, by Lemma 12, $N'$ remains a level-1 network. By Corollary 25, therefore, ${{\,\textrm{lca}\,}}_N(x,y)$ and ${{\,\textrm{lca}\,}}_{N'}(x,y)$ are well defined and, in particular, correspond to unique vertices in *N* and $N'$, respectively.

Assume first that ${{\,\textrm{lca}\,}}_N(x,y)=v'$. Hence, $x,y\prec _N v'$ and there must be children $c,c'$ of $v'$ such that $x\preceq _N c$ and $y\preceq _N c'$. By construction, each of *c* and $c'$ either equals *v* or becomes a child of *v* in $N'$. This and Lemma 3(1) implies that $x\preceq _{N'} c \preceq _{N'} v$ and $y\preceq _{N'} c' \preceq _{N'} v$. Corollary 18 and $x,y\preceq _{N'} v$ imply $z\preceq _{N'} v$. Assume, for contradiction, that $z\prec _{N'} v$ Since $x,y\preceq _{N'} z$, for *x* and *z* (resp. *y* and *z*) one of the Cases (i) or (ii) as specified in Lemma 3(2) must hold.

Assume that Case (i) holds for both *x* and *z* as well as *y* and *z*, i.e., we have $x,y\preceq _N z$. Note that Lemma 3(2) must hold for *z* and *v* as well. Hence, we have (i) $z\preceq _N v$ or (ii) $z\preceq w'$ for some child $w'\ne v$ of $v'$ in *N*. For both cases, we have $x,y\preceq _N z \prec _N v'$, a contradiction to $v' = {{\,\textrm{lca}\,}}_N(x,y)$.

Assume now that Case (ii) is satisfied for *x* and *z*. In this case, $x\preceq _{N'} z$ implies, in particular, that $v\preceq _N z$. This and Lemma 3(1) implies that $v\preceq _{N'} z$. This together with $z\preceq _{N'} v$ implies $z=v$, a contradiction. By similar arguments, Case (ii) cannot hold for *y* and *z*. Hence, neither of the Cases (i) or (ii) as specified in Lemma 3(2) hold for *x* and *z* (resp. *y* and *z*), a contradiction. Therefore, ${{\,\textrm{lca}\,}}_N(x,y)=v'$ implies that ${{\,\textrm{lca}\,}}_{N'}(x,y) = v$.

Assume now that ${{\,\textrm{lca}\,}}_N(x,y)\ne v'$. Let $z{:}{=}{{\,\textrm{lca}\,}}_N(x,y)$ and $z'{:}{=}{{\,\textrm{lca}\,}}_{N'}(x,y)$. Since $z\ne v'$ and $z'\in V(N')$, we can conclude that $z,z'\in V(N)\cap V(N')$. Lemma 3(1) together with $x,y\prec _N z$ implies $x,y\prec _{N'} z$. Assume, for contradiction, that $z\ne z'$. We distinguish Cases (a) $z \prec _{N} z'$, (b) $z'\prec _{N} z$, and (c) *z* and $z'$ are $\preceq _{N}$-incomparable.

In Case (a), $x,y \prec _{N} z \prec _{N} z'$ and Lemma 3(1) imply $x,y \prec _{N'} z \prec _{N'} z'$, a contradiction to $z'= {{\,\textrm{lca}\,}}_{N'}(x,y)$.

In Case (b), suppose first, for contradiction, that $x\not \preceq _{N} z'$. Together with $x\prec _{N'} z'$, this implies that Case (ii) in Lemma 3(2) must hold, i.e., $v\preceq _{N} z'$ and $x\preceq _{N} w'$ for some $w'\in {{\,\textrm{child}\,}}_{N}(v'){\setminus } \{v\}$. In particular, we have $x\preceq _{N} w'\prec _{N} v'$. Since $v'$ is the only parent of *v* in *N*, the case $v\prec _{N} z'$ is not possible as it would imply $v'\preceq _{N} z'$ and thus $x\preceq _{N} w'\prec _{N} v'\preceq _{N} z'$, a contradiction. Hence, we have $v=z'$. Since $z'\prec _{N} z$, $v'\ne z$, and $v'$ is the only parent of $v=z'$ in *N*, we must have $v'\prec _{N} z$. Now, consider $y\prec _{N'} z'(\prec _{N} v')$ which, by Lemma 3(2), implies (i) $y\preceq _{N} z'$ or (ii) $y\preceq _{N} w'$ for some $w''\in {{\,\textrm{child}\,}}_{N}(v')\setminus \{v\}$. In any of the two cases, it holds $y\prec _{N} v'$. Hence, we have $x,y\prec _{N} v'\prec _{N} z$, a contradiction to $z={{\,\textrm{lca}\,}}_{N}(x,y)$. Therefore, it must hold $x\preceq _{N} z'$. By analogous arguments, it holds $y\preceq _{N} z'$. Hence, we have $x,y \preceq _{N} z' \prec _{N} z$, a contradiction to $z={{\,\textrm{lca}\,}}_N(x,y)$.

In Case (c), *z* and $z'$ are $\preceq _{N}$-incomparable. If $x,y\preceq _{N} z'$, then Corollary 18 implies $z={{\,\textrm{lca}\,}}_{N}(x,y)\preceq _{N} z'$, a contradiction. Hence, suppose w.l.o.g. that $x\not \preceq _{N} z'$. Re-using the arguments from Case (b), this implies $v=z'$ and $x,y\prec _{N} v'$. The latter together with Corollary 18 and $z\ne v'$ implies $z={{\,\textrm{lca}\,}}_{N}(x,y)\prec _{N} v'$. Hence, there is some child $c\in {{\,\textrm{child}\,}}_{N}(v')$ with $z\preceq _{N} c$. Since *z* and $z'=v$ are $\preceq _{N}$-incomparable, it holds $c\in {{\,\textrm{child}\,}}_{N}(v'){\setminus }\{v\}$. By construction, therefore, *c* becomes a child of *v* in $N'$, and thus, $c\prec _{N'} v$. Together with Lemma 3(1), the latter arguments imply $x,y\prec _{N'} z\preceq _{N'} c\prec _{N'} v=z'$, a contradiction to $z'={{\,\textrm{lca}\,}}_{N'}(x,y)$.

In summary, neither of Cases (a), (b), and (c) is possible. Therefore, $z=z'$ must hold. $\square$

### Property (L)

#### Lemma 53

Let *N* be a level-1 network with clustering system $\mathscr {C}$, suppose $C_1,C_2\in \mathscr {C}$ overlap, i.e., $C_1\cap C_2\notin \{C_1,C_2,\emptyset \}$. Then, $C_1\cap C_3\in \{ C_1, C_3, \emptyset , C_1\cap C_2\}$ for all $C_3\in \mathscr {C}$.

#### Proof

Let $u_1, u_2\in V(N)$ be vertices such that $C_1={{\,\mathrm{\texttt{C}}\,}}(u_2)$ and $C_2={{\,\mathrm{\texttt{C}}\,}}(u_2)$. Since $C_1$ and $C_2$ overlap, Lemma 17 implies that $u_1$ and $u_2$ are $\preceq _N$-incomparable, and thus, by Lemma 48, $u_1$ and $u_2$ are located in the same block *B*, and $C_1\cap C_2= {{\,\mathrm{\texttt{C}}\,}}(\min B)$. In particular, we have $u_1\ne \max B$ since otherwise $u_2\preceq _{N} u_1$ (cf. Corollary 24). Now, consider a vertex *w* with $C_3={{\,\mathrm{\texttt{C}}\,}}(w)$. If *w* and $u_1$ are $\preceq _N$-comparable, then $C_3\subseteq C_1$ or $C_1\subseteq C_3$ by Lemma 17, and thus, $C_1\cap C_3\in \{C_1,C_3\}$. Now, consider the case where *w* and *u* are $\preceq _N$-incomparable. If $C_1\cap C_3=\emptyset$, there is nothing to show. Otherwise, by Lemma 48, $u_1$ and *w* are located in a common block $B'$ and $C_1\cap C_3= {{\,\mathrm{\texttt{C}}\,}}(\min B')$. We have $u_1\ne \max B'$ since otherwise $w\preceq _{N} u_1$. Hence, we have $u_1\notin \{\max B, \max B'\}$ and thus $B=B'$ by Lemma 9. Therefore, $C_1\cap C_3= {{\,\mathrm{\texttt{C}}\,}}(\min B)= C_1\cap C_2$. $\square$

Inspection of the proof of Lemma 53 shows that there are overlapping clusters only if *N* contains a non-trivial block and thus a hybrid vertex. In particular, therefore, if *N* is a rooted tree, then $\mathscr {C}_N$ is a hierarchy.

Lemma 53 can be rephrased in a more concise form with help of the following

#### Definition 25

(Property (L)) A clustering system $\mathscr {C}$ satisfies property (L) if $C_1\cap C_2=C_1\cap C_3$ for all $C_1,C_2,C_3\in \mathscr {C}$ where $C_1$ overlaps both $C_2$ and $C_3$.

For later reference, we record an equivalent way of expressing property (L):

#### Corollary 27

A clustering system $\mathscr {C}$ satisfies property (L) if and only if $C_1\cap C_2\in \{\emptyset , C_1, C_2, C_1\cap C\}$ for all $C\in \mathscr {C}$ that overlap with $C_1$.

#### Proof

Let $C_1,C_2$ be chosen arbitrarily from $\mathscr {C}$. If $C_1\subseteq C_2$, $C_2\subseteq C_1$ or $C_1\cap C_2 = \emptyset$, then $C_1\cap C_2\in \{\emptyset , C_1, C_2\}$ and there is nothing to show. If neither of the latter cases is satisfied, then $C_1$ and $C_2$ overlap. Let $\mathscr {C}'\subseteq \mathscr {C}$ be the subset of cluster $C\in \mathscr {C}$ that overlap with $C_1$. By construction, $C_1$ overlaps with all elements in $\mathscr {C}'$ and $C_2\in \mathscr {C}'$. Hence, Property (L) holds if and only if $C_1\cap C_2 = C_1\cap C$ for all $C\in \mathscr {C}'$. $\square$

#### Corollary 28

The clustering system $\mathscr {C}_N$ of every level-1 network *N* satisfies property (L).

#### Proof

Let $C_1,C_2,C_3\in \mathscr {C}$ such that $C_1$ overlaps both $C_2$ and $C_3$. Hence, $C_1\cap C_3\notin \{C_1,C_3,\emptyset \}$. This together with Lemma 53 implies that $C_1\cap C_3\in \{C_1\cap C_2\}$. $\square$

#### Corollary 29

A clustering system $\mathscr {C}$ satisfying Property (L) is a weak hierarchy.

#### Proof

If one of the three sets $C_1$, $C_2,$ and $C_3$ is contained in another one, or if one of three pairwise intersections is empty, then the assertion follows immediately. If $C_1$ overlaps both $C_2$ and $C_3,$ then (L) implies $C_1\cap C_2=C_1\cap C_3=C_1\cap C_2\cap C_3$. $\square$

In order to show that closed clustering systems with property (L) define level-1 networks, we first demonstrate that one can identify the non-trivial blocks directly in a clustering system provided it satisfies (L). We start by introducing subsets of clusters with a given overlap: For a given clustering system $\mathscr {C}$ and a set $C\in \mathscr {C}$, define

6 $$\begin{aligned} \mathcal {B}^0(C) {:}{=}\{C'\in \mathscr {C}\setminus \{C\} \mid \ \text {there is a } C''\in \mathscr {C}\setminus \{C\} \text { s.t. } C'\cap C''=C\}. \end{aligned}$$

Note that the clusters $C'$ and $C''$ appearing Eq.(6) are different from $C'\cap C''$ and thus, must overlap. Furthermore, we observe that $C''\in \mathcal {B}^0(C)$ and $\mathcal {B}^0(C)=\emptyset$ if and only if *C* is not the intersection of two overlapping clusters. In particular, we have

#### Lemma 54

Let $\mathscr {C}$ be a clustering system. Then, $\mathscr {C}$ is closed and $\mathcal {B}^0(C)=\emptyset$ for all $C\in \mathscr {C}$ if and only if there is a phylogenetic tree *T* with $\mathscr {C} = \mathscr {C}_T$.

#### Proof

First, let *T* be phylogenetic tree with $\mathscr {C} = \mathscr {C}_T$. By Corollary 26, $\mathscr {C}$ must be closed. Moreover, since *T* is a tree, we have $C'\cap C'' \in \{C',C'', \emptyset \}$ and thus, $\mathcal {B}^0(C)=\emptyset$ since $C',C''$ must satisfy $C',C''\ne C$.

Assume now that $\mathscr {C}$ is closed and $\mathcal {B}^0(C)=\emptyset$ for all $C\in \mathscr {C}$. Since $\mathscr {C}$ is closed, Lemma 16 implies that $C'\cap C''\in \mathscr {C}$ for all $C',C''\in \mathscr {C}$ whenever $C'\cap C''\ne \emptyset$. Therefore, we have $C'\cap C'' \in \{C',C'', \emptyset \}$ for all $C',C''\in \mathscr {C}$ since otherwise $C'$ and $C''$ overlap and we obtain $C',C'\in \mathcal {B}^0(D)\ne \emptyset$ for $D=C'\cap C''\in \mathscr {C}$, a contradiction. Thus, $\mathscr {C}$ is a hierarchy and, by (Theorem 3.5.2 Semple and Steel 2003), there is a 1-to-1 correspondence between hierarchies and phylogenetic trees *T* such that $\mathscr {C} = \mathscr {C}_T$. $\square$

#### Lemma 55

Let $\mathscr {C}$ be a clustering system satisfying (L), and $C\in \mathscr {C}$ with $\mathcal {B}^0(C)\ne \emptyset$. Then, every cluster $D\in \mathscr {C} \setminus \mathcal {B}^0(C)$ satisfies ones the following alternatives: (i) $D\subseteq C$, (ii) $D\cap C=\emptyset$, or (iii) $C'\subsetneq D$ for all $C'\in \mathcal {B}^0(C)$.

#### Proof

Consider a cluster $D\in \mathscr {C}$ with $D\notin \mathcal {B}^0(C)$. By contraposition, assume that none of the alternatives (i), (ii) and (iii) are satisfied. Hence, suppose $D\not \subseteq C$, i.e., $D{\setminus } C\ne \emptyset$, and $D\cap C\ne \emptyset$, and that there is some set $C'\in \mathcal {B}^0(C)$ such that $C'{\setminus } D\ne \emptyset$. The case $C'=D$ cannot occur since $D\notin \mathcal {B}^0(C)$. By definition, there is $C''\in \mathcal {B}^0(C)$ such that $C'$ and $C''$ overlap with $C'\cap C''=C$. In particular, we have $C\subseteq C'$ and $C\subseteq C''$, and thus, $D\cap C'\ne \emptyset$ and $D\cap C''\ne \emptyset$. From $D\cap C'\ne \emptyset$ and $C'{\setminus } D\ne \emptyset$ we infer that either $D\subseteq C'$ or $C'$ and *D* overlap. If $C'$ and *D* overlap, then (L) implies $C'\cap D=C'\cap C''=C$, and thus, $D\in \mathcal {B}^0(C)$, a contradiction. Thus, we have $D\subseteq C'$ and hence $C''\setminus D\ne \emptyset$. Moreover, $D\subseteq C'$, $C'\cap C''=C$, and $D{\setminus } C\ne \emptyset$ imply that $D{\setminus } C''\ne \emptyset$. Together with $D\cap C''\ne \emptyset$, we obtain that $C''$ overlaps with both $C'$ and *D*. Now, (L) implies $C''\cap D=C''\cap C'=C$, and thus, $D\in \mathcal {B}^0(C)$. $\square$

#### Corollary 30

Let $\mathscr {C}$ be a clustering system satisfying (L), and $C\in \mathscr {C}$ with $\mathcal {B}^0(C)\ne \emptyset$. Then, *C* does not overlap with any cluster in $\mathscr {C}$.

#### Proof

If $D\in \mathscr {C} \setminus \mathcal {B}^0(C)$, then Lemma 55 implies that (i) $D\subseteq C$, (ii) $D\cap C=\emptyset$, or (iii) $C\subsetneq C'\subsetneq D$ for all $C'\in \mathcal {B}^0(C)\ne \emptyset$. Hence, *D* does not overlap with *C* in any of the three cases. If, on the other hand, $D\in \mathcal {B}^0(C)$, then $C\subsetneq D$, and thus, *D* and *C* also do not overlap. $\square$

Alternative (iii) in Lemma 55 can be expressed equivalently with the help of the set

7 $$\begin{aligned} U(C) {:}{=}\bigcup _{C'\in \mathcal {B}^0(C)} C' . \end{aligned}$$

#### Lemma 56

Let $\mathscr {C}$ be a clustering system satisfying (L) and let $C\in \mathscr {C}$ such that $\mathcal {B}^0(C)\ne \emptyset$. Then, $D\in \mathscr {C}$ satisfies $C'\subsetneq D$ for all $C'\in \mathcal {B}^0(C)$ if and only if $U(C)\subseteq D$.

#### Proof

The “only if statement” follows directly from the definition of *U*(*C*). To see the “if” direction, note first that $U(C)\subseteq D$ implies $D\notin \mathcal {B}^0(C)$ since otherwise there would be a $C''\in \mathcal {B}^0(C)$ overlapping *D*, which is impossible because $C''\subseteq U(C)\subseteq D$. Furthermore, we have $C'\subsetneq D$ for all $C'\in \mathcal {B}^0(C)$ since *U*(*C*), and thus also *D*, contains at least one set overlapping $C'$. $\square$

As a consequence we can use the condition $U(C)\subseteq D$ instead of alternative (iii) in Lemma 55. If the clustering system $\mathscr {C}$ is closed, $U(C)\subseteq D$ is equivalent to requiring ${{\,\textrm{cl}\,}}(U(C))\subseteq {{\,\textrm{cl}\,}}(D)=D$. Therefore, we define

8 $$\begin{aligned} {{\,\textrm{Top}\,}}(C){:}{=}{{\,\textrm{cl}\,}}\left( U(C)\right) \end{aligned}$$

Alternative (iii) in Lemma 55 can now be expressed as ${{\,\textrm{Top}\,}}(C)\subseteq D$.

**Fig. 16:**
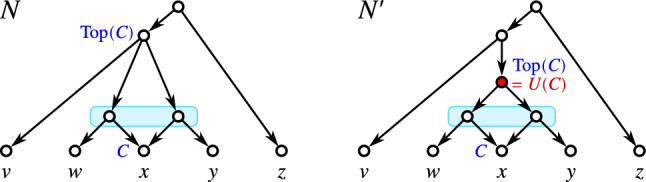
*U*(*C*) is not necessarily a cluster. In both networks $\mathcal {B}(C)$ for $C={{\,\mathrm{\texttt{C}}\,}}\{x\}=\{x\}$ is the only non-trivial block. The vertices with overlapping clusters, i.e., the set $\mathcal {B}^0(C)$ is highlighted in cyan. In both networks $U(C)=\{w,x,y\}$. L.h.s.: $U(C)\subsetneq {{\,\textrm{Top}\,}}(C)$. The additional red vertex r.h.s., ensures that $U(C)={{\,\textrm{Top}\,}}(C)$ is a cluster (color figure online)

In general, $U(C)\notin \mathscr {C}$, see Fig. 16. Nevertheless, ${{\,\textrm{Top}\,}}(C)\in \mathscr {C}$ for all *C* of a closed clustering system $\mathscr {C}$ that satisfy $\mathcal {B}^0(C)$.

#### Lemma 57

Let $\mathscr {C}$ be a closed clustering system. Then, ${{\,\textrm{Top}\,}}(C)\in \mathscr {C}$ and $C\subseteq {{\,\textrm{Top}\,}}(C)$ for all $C\in \mathscr {C}$ with $\mathcal {B}^0(C)\ne \emptyset$.

#### Proof

Let $\mathscr {C}$ be a closed clustering system on *X*. Since $\mathscr {C}$ is closed we have, by definition, ${{\,\textrm{cl}\,}}(A)=A$ if and only if $A\in \mathscr {C}$ for all non-empty sets $A\subseteq 2^X$. Let $C\in \mathscr {C}$ with $\mathcal {B}^0(C)\ne \emptyset$. Hence, $U(C)\ne \emptyset$. Since ${{\,\textrm{cl}\,}}$ is enlarging, we have $U(C)\subseteq {{\,\textrm{cl}\,}}(U(C))={{\,\textrm{Top}\,}}(C)$ and, therefore, ${{\,\textrm{Top}\,}}(C)\ne \emptyset$. In particular, ${{\,\textrm{Top}\,}}(C)\subseteq 2^X$. Since ${{\,\textrm{cl}\,}}$ is idempotent, we have ${{\,\textrm{cl}\,}}({{\,\textrm{Top}\,}}(C)) = {{\,\textrm{cl}\,}}({{\,\textrm{cl}\,}}(U(C))) = {{\,\textrm{cl}\,}}(U(C)) = {{\,\textrm{Top}\,}}(C)$. Taking the latter arguments together, we obtain ${{\,\textrm{Top}\,}}(C)\in \mathscr {C}$. Moreover, if $\mathcal {B}^0(C)\ne \emptyset$, then it is straightforward to verify that $C\subseteq U(C)\subseteq {{\,\textrm{Top}\,}}(C)$. $\square$

Although $U(C)\notin \mathscr {C}$, it has an interesting property:

#### Lemma 58

Let $\mathscr {C}$ be a clustering system satisfying (L), and $C\in \mathscr {C}$ with $\mathcal {B}^0(C)\ne \emptyset$. Then, *U*(*C*) does not overlap any cluster $D\in \mathscr {C}$.

#### Proof

Let $D\in \mathscr {C}$. If $D\in \mathcal {B}^0(C)$, then $D\subseteq U{:}{=}U(C)$ by construction. If $D\notin \mathcal {B}^0(C)$, we consider the three alternatives of Lemma 55. In case (i) we have $D\subseteq C\subseteq U$ and in case (iii) we have $U\subseteq {{\,\textrm{Top}\,}}(C)\subseteq D$. In case (ii) we have $D\cap C=\emptyset$. If $D\cap C'=\emptyset$ for all $C'\in \mathcal {B}^0(C)$, then $D\cap U=\emptyset$. Otherwise $C'\cap D\ne \emptyset$ for some $C'\in \mathcal {B}^0(C)$. Since $C\subseteq C'$ and $D\cap C=\emptyset$, we have $C'\not \subseteq D$. However, $C'$ and *D* cannot overlap since in this case (L) implies $C'\cap D=C$ and thus, $D\in {B}^0(C)$, a contradiction. Therefore, $D\subseteq C'\setminus C$, which implies $D\subsetneq U$. Thus, *D* does not overlap *U*. $\square$

So far, ${{\,\textrm{Top}\,}}(C)$ is defined in terms of non-empty sets $\mathcal {B}^0(C)$. We extend this notion to all clusters of a closed clustering system as follows. Let $\mathscr {C}$ be a closed clustering system. We set ${{\,\textrm{Top}\,}}(X){:}{=}X$. For the remaining clusters $C\in \mathscr {C}$, i.e., those that satisfy $\mathcal {B}^0(C)=\emptyset$ and $C\ne X$, we define ${{\,\textrm{Top}\,}}(C)$ as the unique inclusion-minimal cluster $C'\ne C$ that contains *C*. To see that ${{\,\textrm{Top}\,}}(C)$ is well defined in this case, recall first that $X\in \mathscr {C}$ and hence there is a cluster properly containing $C\ne X$. For uniqueness, suppose there are two distinct inclusion-minimal clusters $C',C''\ne C$ that contain *C*. Clearly, these two supersets overlap with $C\subseteq C^*{:}{=}C'\cap C''$. If $C= C^*$, then $\mathcal {B}^0(C)\ne \emptyset$, a contradiction. If $C\subsetneq C^*$, we have $C^*\in \mathscr {C}$ since $\mathscr {C}$ is closed and thus $C\subsetneq C^*\subsetneq C,C'$ contradicts inclusion-minimality of *C* and $C'$. Now, set

9 $$\begin{aligned} \mathcal {B}(C){:}{=}\mathcal {B}^0(C)\cup \{C,{{\,\textrm{Top}\,}}(C)\} \text { for all } C\in \mathscr {C}. \end{aligned}$$

#### Corollary 31

If $\mathscr {C}$ is a closed clustering system, then $\mathcal {B}(C)\subseteq \mathscr {C}$ for all $C\in \mathscr {C}$.

#### Proof

If $\mathcal {B}^0(C) = \emptyset$, we have by construction, $\mathcal {B}(C){:}{=}\emptyset \cup \{C,C'\} = \{C,C'\}$, where $C'$ is the unique inclusion-minimal element in $\mathscr {C}$ that contains *C*. Hence, $\mathcal {B}(C)\subseteq \mathscr {C}$. The latter covers in particular also the case $C=X$. Otherwise, if $\mathcal {B}^0(C) \ne \emptyset$, then Lemma 57 implies that ${{\,\textrm{Top}\,}}(C)\in \mathscr {C}$. Moreover, by definition, $\mathcal {B}^0(C)\subseteq \mathscr {C}$. Taken the latter together with $C\in \mathscr {C}$ implies $\mathcal {B}(C)\subseteq \mathscr {C}$. $\square$

Lemma 55 then implies the following characterization of $\mathcal {B}(C)$:

#### Corollary 32

Let $\mathscr {C}$ be a closed clustering system satisfying (L). Then, for all $C,D\in \mathscr {C}$ it holds that $D\in \mathcal {B}(C)$ if and only if $C\subseteq D\subseteq {{\,\textrm{Top}\,}}(C)$.

#### Proof

Let $C,D\in \mathscr {C}$ such that $D\in \mathcal {B}(C) = \mathcal {B}^0(C)\cup \{C,{{\,\textrm{Top}\,}}(C)\}$. If $D \in \mathcal {B}^0(C)$ or $D=C$, then $C\subseteq D$ and, by construction, $D \subseteq {{\,\textrm{Top}\,}}(C)$. If $D = {{\,\textrm{Top}\,}}(C)$, we can apply Lemma 57 to conclude that $C\subseteq {{\,\textrm{Top}\,}}(C)=D$. Now, let $C\subseteq D\subseteq {{\,\textrm{Top}\,}}(C)$ and assume, for contradiction, that $D\notin \mathcal {B}(C)$. Thus, $C\subsetneq D\subsetneq {{\,\textrm{Top}\,}}(C)$ and $D\notin \mathcal {B}^0(C)$. From $D\subsetneq {{\,\textrm{Top}\,}}(C)$, we obtain $\mathcal {B}^0(C)\ne \emptyset$. Hence, we can apply Lemma 55 to conclude that the cluster *D* satisfies one of the alternatives: (i) $D\subseteq C$, (ii) $D\cap C=\emptyset$, or (iii) $C'\subsetneq D$ for all $C'\in \mathcal {B}^0(C)$. Based on the latter arguments, only case (iii) can occur, and hence, Lemma 56 and Eq. (8) yield ${{\,\textrm{Top}\,}}(C)\subseteq D$, a contradiction. $\square$

As an immediate consequence of Corollary 32 we have

#### Corollary 33

Let $\mathscr {C}$ be a closed clustering system satisfying (L). Then, for all clusters $C\in \mathscr {C}$, the Hasse diagram ${\mathfrak {H}}[\mathcal {B}(C)]$ is an induced subgraph of the Hasse diagram ${\mathfrak {H}}[\mathscr {C}]$.

Furthermore, the sets $\mathcal {B}^0(C)$ are pairwise disjoint:

#### Lemma 59

Let $\mathscr {C}$ be a closed clustering system satisfying (L) and let $C,C'\in \mathscr {C}$. Then, $C'\in \mathcal {B}^0(C)$ implies $\mathcal {B}^0(C')=\emptyset$. Furthermore, if $\mathcal {B}^0(C)\cap \mathcal {B}^0(C')\ne \emptyset$, then $C=C'$. Consequently, $\mathcal {B}^0(C)\cap \mathcal {B}^0(C') =\emptyset$ for all distinct $C,C'\in \mathscr {C}$.

#### Proof

Suppose that $C'\in \mathcal {B}^0(C)$. Then, there is a set $C''\in \mathscr {C}$ such that $C'$ and $C''$ overlap and $C=C'\cap C''$. Assume for contradiction that $\mathcal {B}^0(C')\ne \emptyset$, i.e., there are two overlapping clusters $D,D'\in \mathscr {C}$ such that $C'=D\cap D'$. Since $C''$ overlaps $C'$ it cannot be contained in both *D* and $D'$ since otherwise $C''\subseteq D\cap D'=C'$ and hence, $C'$ and $C''$ would not overlap. Thus, at least one of $C''{\setminus } D$ and $C''{\setminus } D'$ is non-empty, say $C''\setminus D\ne \emptyset$. Moreover, $C'\subsetneq D$ and $C'$ and $C''$ overlapping each other imply that $D{\setminus } C''\ne \emptyset$ and $D\cap C''\ne \emptyset$. Hence, $C''$ and *D* overlap. By (L), $C = C'\cap C'' = C''\cap D = D'\cap D = C'$ and thus $C'=C\subseteq C''$, a contradiction to the assumption that $C'$ and $C''$ overlap. Hence, $\mathcal {B}^0(C')=\emptyset$ as claimed. Now, assume that $\mathcal {B}^0(C)\cap \mathcal {B}^0(C')\ne \emptyset$, i.e., there is a cluster $C''\in \mathcal {B}^0(C)\cap \mathcal {B}^0(C')$ and clusters $D\in \mathcal {B}^0(C)$ and $D'\in \mathcal {B}^0(C')$, both of which overlap with $C''$, such that $C=C''\cap D$ and $C'=C''\cap D'$. By (L), this implies $C=C'$. $\square$

#### Lemma 60

Let $\mathscr {C}$ be a closed clustering system satisfying (L). Then, $C\in \mathscr {C}$ has indegree greater than one in ${\mathfrak {H}}[\mathscr {C}]$ if and only if $\mathcal {B}^0(C)\ne \emptyset$. In this case, all in-neighbors of *C* in ${\mathfrak {H}}[\mathscr {C}]$ are contained in $\mathcal {B}^0(C)$.

#### Proof

Suppose $C\in \mathscr {C}$ has indegree greater than one in ${\mathfrak {H}}[\mathscr {C}]$. Thus, let $D,D'\in \mathscr {C}$ be two distinct in-neighbors of *C*. Hence, $C\subseteq D\cap D'$ and thus, by closedness of $\mathscr {C}$ and definition of ${\mathfrak {H}}[\mathscr {C}]$, $C= D\cap D'$. In particular, *D* and $D'$ overlap. Therefore, $D,D'\in \mathcal {B}^0(C)\ne \emptyset$. In particular, since the in-neighbors *D* and $D'$ were chosen arbitrarily, all in-neighbors of *C* in ${\mathfrak {H}}[\mathscr {C}]$ are contained in $\mathcal {B}^0(C)$. Now, suppose $C\in \mathscr {C}$ has indegree zero or one in ${\mathfrak {H}}[\mathscr {C}]$. Clearly, *C* has indegree zero if and only if $C=X$, in which case $\mathcal {B}^0(C)=\emptyset$. Suppose *C* has exactly one in-neighbor $C'$ and, for contradiction, that $\mathcal {B}^0(C)\ne \emptyset$. Then, there are two overlapping sets $D,D'\in \mathcal {B}^0(C)$ such that $C=D\cap D'$, and thus directed paths both from *D* and $D'$ to *C*. Both of these paths must pass through $C'$ and thus, $C\subsetneq C'\subseteq D\cap D'$, a contradiction. Hence, the *if*-direction must also hold. $\square$

Lemmas 59 and 60 imply

#### Corollary 34

Let $\mathscr {C}$ be a closed clustering system satisfying (L) and with Hasse diagram ${\mathfrak {H}}$. For every $C\in \mathscr {C}$, the elements $C'\in \mathcal {B}^0(C)$ have a unique in-neighbor in ${\mathfrak {H}}$. In particular, this unique in-neighbor of $C'$ is always contained in $\mathcal {B}(C)$.

#### Proof

The statement is trivially true for all $C\in \mathscr {C}$ with $\mathcal {B}^0(C)=\emptyset$. Thus, consider a set $C\in \mathscr {C}$ with $\mathcal {B}^0(C)\ne \emptyset$. Note that $C\ne X$ must hold. Consider a cluster $C'\in \mathcal {B}^0(C)$. It overlaps with some $C''\in \mathcal {B}^0(C)$ and thus $C',C''\subsetneq {{\,\textrm{Top}\,}}(C)$. Therefore, there is a directed path from ${{\,\textrm{Top}\,}}(C)$ to $C'$ and thus, $C'$ has an in-neighbor $C^*$ that satisfies $C\subsetneq C'\subsetneq C^*\subseteq {{\,\textrm{Top}\,}}(C)$. By Corollary 32, we have $C^*\in \mathcal {B}(C)$. Lemma 59 and $C'\in \mathcal {B}^0(C)$ imply $\mathcal {B}^0(C')=\emptyset$. Hence, $C'$ has indegree 1 by Lemma 60, i.e., $C^*\in \mathcal {B}(C)$ is the unique in-neighbor of *C*. $\square$

#### Lemma 61

Let $\mathscr {C}$ be a closed clustering system satisfying (L). Let $C\in \mathscr {C}$ with $\mathcal {B}^0(C)\ne \emptyset$. Then, the induced subgraph ${\mathfrak {H}}[\mathcal {B}(C)]$ of ${\mathfrak {H}}[\mathscr {C}]$ is biconnected. In particular, ${\mathfrak {H}}[\mathcal {B}(C)]$ is a DAG with unique source ${{\,\textrm{Top}\,}}(C)$ and unique sink *C*.

#### Proof

By Corollary 33, ${\mathfrak {H}}[\mathcal {B}(C)]$ is an induced subgraph of ${\mathfrak {H}}[\mathscr {C}]$. By Lemma 34, all clusters in $\mathcal {B}^0(C)$ have a unique in-neighbor in $\mathcal {B}(C)$. By Corollary 32, $C'\subseteq {{\,\textrm{Top}\,}}(C)$ holds for all $C'\in \mathcal {B}^0(C)$. Therefore, ${{\,\textrm{Top}\,}}(C)$ has indegree 0 in ${\mathfrak {H}}[\mathcal {B}(C)]$ and, moreover, there exists a directed path from ${{\,\textrm{Top}\,}}(C)$ to every cluster $C'\in \mathcal {B}^0(C)$. In particular, by Corollary 32, of the clusters in such paths are again contained in $\mathcal {B}(C)$. Taken together, these arguments imply that the Hasse diagram ${\mathfrak {H}}[\mathcal {B}(C)\setminus \{C\}]$ is a tree with root ${{\,\textrm{Top}\,}}(C)$. Note that this tree is not necessarily phylogenetic, i.e., there may exist clusters with outdegree 1. However, the outdegree of the root ${{\,\textrm{Top}\,}}(C)$ in ${\mathfrak {H}}[\mathcal {B}(C)\setminus \{C\}]$ is at least two. To see this, let $C'$ be a cluster in $\mathcal {B}^0(C)\ne \emptyset$. As argued above, there is a directed path from ${{\,\textrm{Top}\,}}(C)$ to $C'$ and this path only contains clusters in $\mathcal {B}(C)$. Therefore, and since $C'\ne {{\,\textrm{Top}\,}}(C)$, ${{\,\textrm{Top}\,}}(C)$ has a child $D'$ in ${\mathfrak {H}}$ with $C\subsetneq C' \subseteq D' \subsetneq {{\,\textrm{Top}\,}}(C)$. By Corollary 32, we have $D'\in \mathcal {B}(C)$, and thus, $D'\in \mathcal {B}^0(C)$. Hence, there is $C''\in \mathcal {B}^0(C)$ such that $D'$ and $C''$ overlap. By similar argument as before, there is a child $D''\in \mathcal {B}^0(C)$ of ${{\,\textrm{Top}\,}}(C)$ such that $C'' \subseteq D'' \subsetneq {{\,\textrm{Top}\,}}(C)$. Now, $C'' \subseteq D''$ and the fact that $D'$ and $C''$ overlap imply that $D'\ne D''$. Hence, ${{\,\textrm{Top}\,}}(C)$ has at least two children in ${\mathfrak {H}}[\mathcal {B}(C)\setminus \{C\}]$. Using Corollary32, we see that each leaf of the tree induced by $\mathcal {B}(C)\setminus \{C\}$ is an in-neighbor of *C*. It is now easy to verify the graph obtained from (i) a rooted tree whose root has at least two children and (ii) connecting its leaves to an additional vertex is biconnected. Hence, ${\mathfrak {H}}[\mathcal {B}(C)]$ is biconnected. In particular, ${\mathfrak {H}}[\mathcal {B}(C)]$ features at least two internally vertex disjoint directed path connecting ${{\,\textrm{Top}\,}}(C)$ and *C*, and any two vertices lie along a common “undirected” cycle (which necessarily passes through *C*). $\square$

#### Lemma 62

Let $\mathscr {C}$ be a closed clustering system satisfying (L). Let $D\in \mathcal {B}^0(C)$ for some $C\in \mathscr {C}$ and let $D'\notin \mathcal {B}(C)$ be adjacent to *D* in the Hasse diagram ${\mathfrak {H}}$ of $\mathscr {C}$. Then,

(i) *D* is the unique in-neighbor of $D'$ in ${\mathfrak {H}}$ and thus $D'\subsetneq D$,
(ii) $$D'\cap C =\emptyset$$, and
(iii) if $D'$ overlaps with some $D''\in \mathscr {C}$, then there is $C'\in \mathscr {C}$ such that $D'\in \mathcal {B}^0(C')$ and ${{\,\textrm{Top}\,}}(C')=D$.

#### Proof

We start with showing Property (i). By Corollary 34, the unique in-neighbor of *D* is contained in $\mathcal {B}(C)$. Thus, $D'$ must be an out-neighbor of *D*, i.e., $D'\subsetneq D$. If $\mathcal {B}^0(D')\ne \emptyset$, then Lemma 60 implies $D\in \mathcal {B}^0(D')$. Lemma 59 and $D\in \mathcal {B}^0(C)\cap \mathcal {B}^0(D')$ imply $D'=C\in \mathcal {B}(C)$, a contradiction. Hence, $\mathcal {B}^0(D')=\emptyset$ and in particular, by Lemma 60, $D'$ has indegree 1, and thus, *D* is its unique in-neighbor.

We continue with showing Property (ii). Since $\mathcal {B}^0(C)\ne \emptyset$ and $D'\notin \mathcal {B}^0(C)$, Lemma 55 implies that (a) $D'\subseteq C$, (b) $D'\cap C=\emptyset$, or (c) $C'\subsetneq D'$ for all $C'\in \mathcal {B}^0(C)$. In Case (a), $D'\notin \mathcal {B}(C)$ implies $D'\subsetneq C$. Since moreover $D\in \mathcal {B}^0(C)$ and thus $C\subsetneq D$, we have $D'\subsetneq C\subsetneq D$, contradicting that $D'$ and *D* are adjacent in ${\mathfrak {H}}$. In Case (c), we obtain $D\subsetneq D'$ since $D\in \mathcal {B}^0(C)$; contradicting $D'\subsetneq D$. Hence, only Case (b) $D'\cap C=\emptyset$ can hold.

Finally, we show Property (iii). Suppose that $D'$ overlaps $D''$ and set $C'=D'\cap D''$ and thus, $D'\in \mathcal {B}^0(C')$. Since $D'\notin \mathcal {B}^0(C)\subseteq \mathcal {B}(C)$ it must hold that $C'\ne C$ and thus $\mathcal {B}^0(C')\cap \mathcal {B}^0(C)=\emptyset$ by Lemma 59. Since *D* is the unique in-neighbor of $D'$ in ${\mathfrak {H}}$, Corollary 34 implies $D\in \mathcal {B}(C')$ and thus $D'\subsetneq D\subseteq {{\,\textrm{Top}\,}}(C')$. On the other hand, $D\in \mathcal {B}^0(C)$ implies $D\notin \mathcal {B}^0(C')$ and hence $D\not \subsetneq {{\,\textrm{Top}\,}}(C')$; therefore, $D={{\,\textrm{Top}\,}}(C')$. $\square$

#### Lemma 63

Let $\mathscr {C}$ be a closed clustering system satisfying (L). Then, each subgraph ${\mathfrak {H}}[\mathcal {B}(C)]$ with $\mathcal {B}^0(C)\ne \emptyset$ is a non-trivial block of the Hasse diagram ${\mathfrak {H}}$ of $\mathscr {C}$.

#### Proof

By Lemma 61, ${\mathfrak {H}}[\mathcal {B}(C)]$ is biconnected. Therefore, and since $\mathcal {B}^0(C)\ne \emptyset$, the set $\mathcal {B}(C)$ contains at least four clusters, i.e., *C*, ${{\,\textrm{Top}\,}}(C)$, and at least two overlapping clusters in $\mathcal {B}^0(C)$. Thus, it only remains to show that ${\mathfrak {H}}[\mathcal {B}(C)]$ is a maximal biconnected subgraph of ${\mathfrak {H}}$. Since moreover, by Corollary 33, ${\mathfrak {H}}[\mathcal {B}(C)]$ is an induced subgraph of ${\mathfrak {H}}$, ${\mathfrak {H}}[\mathcal {B}(C)]$ is a maximal if and only if there is no undirected cycle in ${\mathfrak {H}}$ that contains an arc of ${\mathfrak {H}}[\mathcal {B}(C)]$ and a vertex not contained in $\mathcal {B}(C)$ (cf. Observation 1). Assume, for contradiction, that such a cycle *K* exists. Since *K* contains at least one arc of ${\mathfrak {H}}[\mathcal {B}(C)]$, we can find a maximal subpath *P* of *K* on at least two vertices and where all vertices of *P* are contained in $\mathcal {B}(C)$. In particular, the two distinct endpoints of *P* are both incident with one cluster in $\mathcal {B}(C)$ and one cluster that is not in $\mathcal {B}(C)$. Clearly, at least one of the endpoints of *P* must be distinct from ${{\,\textrm{Top}\,}}(C)$. Hence, we can pick an endpoint $D\in \mathcal {B}(C) {\setminus }\{{{\,\textrm{Top}\,}}(C)\}$ of *P* that is adjacent to $C'\in \mathcal {B}(C)$ and $D'\notin \mathcal {B}(C)$, where both $C'$ and $D'$ are vertices in *K*. Therefore, it suffices to consider the two mutually exclusive cases (a) $D=C$ and (b) $D\in \mathcal {B}^0(C)$:

- $D=C$. Hence, $C'\ne C$ and thus, by Corollary 32, $C'\in \mathcal {B}(C)\setminus \{C\}$ implies $D=C\subsetneq C'$ and thus, $C'\in \overline{\mathcal {D}}(C)$ (cf. Eq. (3)). Suppose, for contradiction, that *C* overlaps with some cluster $D''\in \mathscr {C}$. Then, since $\mathscr {C}$ is closed, we have $C\in \mathcal {B}^0(E)$ for $E=C\cap C''\in \mathscr {C}$. However, this together with Lemma 59 implies $\mathcal {B}^0(C)\ne \emptyset$, a contradiction. Hence, *C* does not overlap any cluster. Furthermore, by Lemma 60, all in-neighbors of *C* are contained in $\mathcal {B}^0(C)\subsetneq \mathcal {B}(C)$. Therefore, $D'$ must be an out-neighbor of *C* and thus $D'\subsetneq C$, which implies $D'\in \mathcal {D}(C)$. Hence, we can apply Lemma 23 to conclude that there is no cycle *K* containing $D'\in \mathcal {D}(C)$ and $C'\in \overline{\mathcal {D}}(C)$, a contradiction.
- $D\in \mathcal {B}^0(C)$. By Lemma 62, *D* is the unique in-neighbor of $D'$. However, since $D'$ is located on the cycle *K*, it must be adjacent to another vertex $D''\ne D$ in *K*. Since *D* is the unique in-neighbor of $D'$ it follows that $D''$ must be an out-neighbor of $D'$ and thus, $D''\subsetneq D'$. By construction, $D''\in \mathcal {D}(D')$ and $D\in \overline{\mathcal {D}}(D')$. If $D'$ does not overlap any cluster in $\mathscr {C}$, then we can apply Lemma 23 to conclude that there is no cycle *K* in ${\mathfrak {H}}$ containing $D''\in \mathcal {D}(D')$ and $D\in \overline{\mathcal {D}}(D')$, a contradiction. Hence, $D'$ must overlap with some cluster in $\mathscr {C}$. Then, Lemma 62(iii) implies that there is $E\in \mathscr {C}$ such that $D'\in \mathcal {B}^0(E)$ and $D={{\,\textrm{Top}\,}}(E)$. In particular, since $D\ne {{\,\textrm{Top}\,}}(C)$, we have $D\ne E$. Moreover, by Lemma 62(ii), we have $D_1\cap C=\emptyset$ for all children $D_1$ of ${{\,\textrm{Top}\,}}(E)=D\in \mathcal {B}^0(C)$ with $D_1\notin \mathcal {B}^0(C)$. In particular, $C\subsetneq D$, and thus, we have $U{:}{=}U(E)=\bigcup _{D_1\in \mathcal {B}^0(E)}D_1 \subsetneq {{\,\textrm{Top}\,}}(E)=D$. On the other hand, we have $D_1\subsetneq U$ for each of the children of ${{\,\textrm{Top}\,}}(E)$. Since $C\subsetneq D$, *D* has at least one child *F* such that $C\subseteq F$. We continue with showing that $C\cap D_1=\emptyset$ for all $D_1\in \mathcal {B}^0(E)$. Hence, let $D_1\in \mathcal {B}^0(E)$. By Corollary 30, *C* does not overlap with any cluster in $\mathscr {C}$. In particular, this yield $C\ne D_1\in \mathcal {B}^0(E)$ and *C* and $D_1$ do not overlap. The case $C\subsetneq D_1$ is not possible since otherwise $C\subsetneq D_1 \subsetneq {{\,\textrm{Top}\,}}(E)=D\subsetneq {{\,\textrm{Top}\,}}(C)$ and Corollary 32 would imply that $D_1\in \mathcal {B}^0(C)$. Together with Lemma 59, this would imply $C=E$, a contradiction. Now, suppose $D_1\subsetneq C$. Thus, we have $E\subsetneq D_1\subsetneq C\subsetneq D = {{\,\textrm{Top}\,}}(E)$. By Corollary 32, this implies $C\in \mathcal {B}^0(E)$. However, this is not possible because *C* does not overlap with any other cluster. Hence, $C\cap D_1=\emptyset$ must hold for all $D_1\in \mathcal {B}^0(E)$. Therefore, we obtain $U\cap C=\emptyset$. Together with $U\subseteq D$ and $C\subsetneq D$, this implies $U\subsetneq D={{\,\textrm{Top}\,}}(E)$. To summarize, since $\mathscr {C}$ is closed, it holds by definition that ${{\,\textrm{cl}\,}}(U)=U \iff U\in \mathscr {C}$. The latter arguments taken together with ${{\,\textrm{Top}\,}}(E)={{\,\textrm{cl}\,}}(U)$ imply $U\notin \mathscr {C}$. consider $\mathscr {C}^*{:}{=}\mathscr {C}\cup \{U\}$. Clearly, the Hasse diagram ${\mathfrak {H}}^*$ of $\mathscr {C}^*$ is obtained from ${\mathfrak {H}}$ by inserting a extra vertex *U* as child of $D={{\,\textrm{Top}\,}}(E)$ and re-attaching the children $D_1$ of ${{\,\textrm{Top}\,}}(E)$ with $D_1\in \mathcal {B}^0(E)$ in ${\mathfrak {H}}$ as children of *U* in ${\mathfrak {H}}^*$, while the children $D_2$ of ${{\,\textrm{Top}\,}}(C)$ with $D_2\notin \mathcal {B}^0(E)$ remain attached to *D*. In $\mathscr {C}^*$ we therefore have ${{\,\textrm{Top}\,}}(E)=U$. Since *U* does not overlap any set in $\mathscr {C}$ by Lemma 58, $\mathscr {C}^*$ is again a closed clustering system and satisfies (L). Moreover, since $U\subsetneq D$ we have $U\ne X$ and since $\mathcal {B}^0(E)\ne \emptyset$ we have $\vert U\vert >1$. Hence, we can apply Lemma 23 to conclude that *U* is a cut vertex in ${\mathfrak {H}}^*$ and that there is no cycle in ${\mathfrak {H}}^*$ containing both a vertex in $\mathcal {D}(U)$ and in $\overline{\mathcal {D}}(U)$. Since $C'\in \mathcal {B}(C)$, we have $C\subseteq C'$, which together with $U\cap C=\emptyset$ implies that $C'\in \overline{\mathcal {D}}(U)$. Furthermore, $D'\in \mathcal {B}^0(E)$ implies that $D\subsetneq U$ and thus, $D' \in \mathcal {D}(U)$. Taking the latter arguments together, there is no cycle in ${\mathfrak {H}}^*$ that contains both $C'$ and $D'$. Since ${\mathfrak {H}}$ is recovered from ${\mathfrak {H}}^*$ by “contracting” the arc *UD*, there is no cycle in ${\mathfrak {H}}$ that contains both $C'$ and $D'$, a contradiction.

$$\square$$

#### Lemma 64

Let $\mathscr {C}$ be a closed clustering system on *X* satisfying (L). If $C\in \mathscr {C}\setminus \{X\}$, $\mathcal {B}^0(C)=\emptyset$, and $C\notin \mathcal {B}^0(C')$ for all $C'\in \mathscr {C}$, then the arc $({{\,\textrm{Top}\,}}(C),C)$ is a block in ${\mathfrak {H}}[\mathscr {C}]$.

#### Proof

We show that the arc $({{\,\textrm{Top}\,}}(C),C)$ is not contained in any cycle in ${\mathfrak {H}}$. Since $\mathscr {C}$ is closed and $C\notin \mathcal {B}^0(C')$ for all $C'\in \mathscr {C}$, we know that *C* does not overlap any cluster. By Lemma 23, there is no cycle that intersects both $\mathcal {D}(C)$ and $\overline{\mathcal {D}}(C)$. Since $C\subsetneq {{\,\textrm{Top}\,}}(C)$, we have ${{\,\textrm{Top}\,}}(C)\in \overline{\mathcal {D}}(C)$. Furthermore, Lemma 60 and $\mathcal {B}^0(C)=\emptyset$ imply that ${{\,\textrm{Top}\,}}(C)$ is the only in-neighbor of *C* in ${\mathfrak {H}}[\mathscr {C}]$. Therefore, any cycle that contains $({{\,\textrm{Top}\,}}(C),C)$ must contain some child $C'$ of *C*. Clearly, $C'\in \mathcal {D}(C)$ and thus such a cycle cannot exist as it would intersect both $\mathcal {D}(C)$ and $\overline{\mathcal {D}}(C)$. Hence, $({{\,\textrm{Top}\,}}(C),C)$ is a cut arc, and thus a block. $\square$

We summarize Lemmas 63 and 64 in

#### Proposition 17

Let $\mathscr {C}$ be a closed clustering system on *X* satisfying (L) and with Hasse diagram ${\mathfrak {H}}$. Then, *B* is a block of ${\mathfrak {H}}$ if and only if $\vert X\vert =1$ or $\vert X\vert >1$ and $B= {\mathfrak {H}}[\mathcal {B}(C)]$ for some $C\in \mathscr {C}$ that satisfies either (i) $\mathcal {B}^0(C)\ne \emptyset$ or (ii) $C\ne X$ does not overlap any cluster and $\mathcal {B}^0(C)=\emptyset$. If $\vert X\vert =1$ or in Case (ii) *B* is a trivial block and, otherwise, in Case (i) a non-trivial one.

#### Proof

If $\vert X\vert =1$, then $B= {\mathfrak {H}}[\mathcal {B}(C)] = {\mathfrak {H}}$ consists a single vertex only and is, therefore, a block of ${\mathfrak {H}}$. Assume that $\vert X\vert >1$. By Lemma 63 and Lemma 64, each subgraph ${\mathfrak {H}}[\mathcal {B}(C)]$ with $\mathcal {B}^0(C)\ne \emptyset$ is a non-trivial block and ${\mathfrak {H}}[\mathcal {B}(C)]$ for which $C\in \mathscr {C}{\setminus } \{X\}$ does not overlap any cluster and $\mathcal {B}^0(C)=\emptyset$ is a trivial block of the Hasse diagram ${\mathfrak {H}}$.

For the converse, suppose first that *B* is a trivial block of ${\mathfrak {H}}$, i.e., it only consists of the single vertex *C* or the single arc $(C',C)$. In the first case, we have $\vert X\vert =1$. Otherwise, ${\mathfrak {H}}$ consists of $(C',C)$ and hence $\vert X\vert >1$. Moreover, we have $C\subsetneq C'\subseteq X$ and thus $C\in \mathscr {C}{\setminus } \{X\}$. If $\mathcal {B}^0(C)\ne \emptyset$, then, by Lemma 60, $C'\in \mathcal {B}^0(C)\subsetneq \mathcal {B}(C)$. Moreover, ${\mathfrak {H}}[\mathcal {B}(C)]$ is a non-trivial block of ${\mathfrak {H}}$ by Lemma 63. In particular, the arc $(C',C)$ is contained in this block, contradicting that $(C',C)$ forms a trivial block. Hence, we have $\mathcal {B}^0(C) = \emptyset$. Assume, for contradiction, that *C* overlaps with some cluster $C''\in \mathscr {C}$. Then, by closedness of $\mathscr {C}$, $C\in \mathcal {B}^0(D)$ for some $D\in \mathscr {C}$. Then, by Corollary 34, $C'$ is the unique in-neighbor of $C\in \mathcal {B}^0(D)$ in ${\mathfrak {H}}$ and $C'\in \mathcal {B}(D)$. Hence, *C* and $C'$ are contained in ${\mathfrak {H}}[\mathcal {B}(D)]$, which is non-trivial as a consequence of $C\in \mathcal {B}^0(D)$ and Lemma 63. This again contradicts that $(C',C)$ forms a trivial block. In summary, we have $C\in \mathscr {C}{\setminus } \{X\}$, $\mathcal {B}^0(C)=\emptyset$, and *C* does not overlap any cluster. Suppose now that *B* is a non-trivial block of ${\mathfrak {H}}$. Hence, $\vert X\vert >1$ and *B* contains an undirected cycle *K* on at least 3 clusters. Since ${\mathfrak {H}}$ is a DAG, *K* contains at least one cluster *C* with two in-neighbors $C'$ and $C''$ in *K* (and thus in ${\mathfrak {H}}$). By Lemma 60, we have $C',C''\in \mathcal {B}^0(C)$. Therefore, Lemma 63 implies that ${\mathfrak {H}}[\mathcal {B}(C)]$ is a non-trivial block of ${\mathfrak {H}}$. In particular, ${\mathfrak {H}}[\mathcal {B}(C)]$ contains the arcs $C'C$ and $C''C$, which are also arcs in *B*. By Observation 2, we therefore obtain $B={\mathfrak {H}}[\mathcal {B}(C)]$. $\square$

### Characterization of clustering systems of level-1 networks

We start with showing that a regular network is level-1 provided that its clustering is closed and satisfied (L).

#### Proposition 18

Let $\mathscr {C}$ be a closed clustering system on *X* satisfying (L). Then, the Hasse diagram ${\mathfrak {H}}$ of $\mathscr {C}$ is a phylogenetic level-1 network with leaf set $X_{{\mathfrak {H}}}{:}{=}\{ \{x\} \mid x \in X \}$.

#### Proof

By Lemma 22, ${\mathfrak {H}}$ is a phylogenetic network with leaf set $X_{{\mathfrak {H}}}{:}{=}\{ \{x\} \mid x \in X \}$. To show that ${\mathfrak {H}}$ is level-1, we have to demonstrate that each block *B* of ${\mathfrak {H}}$ contains at most one hybrid vertex that is distinct from the unique maximum $\max B$. This holds trivially if *B* is a trivial block consisting of a single arc or, if $\vert X\vert =1$, an isolated vertex. Now, suppose that *B* is a non-trivial block, and thus, by Proposition 17, it contains exactly the clusters in $\mathcal {B}(C)$ for some $C\in \mathscr {C}$ with $\mathcal {B}^0(C)\ne \emptyset$. By Lemma 60, *C* is a hybrid vertex. From Corollary 32 and the construction of the Hasse diagram, we conclude that ${{\,\textrm{Top}\,}}(C)=\max B$. By Corollary 34, none of the clusters in $\mathcal {B}^0(C)$ is a hybrid vertex. Hence, *C* is the only hybrid vertex in $\mathcal {B}(C)=\mathcal {B}^0(C)\cup \{C,{{\,\textrm{Top}\,}}(C)\}$ that is distinct from ${{\,\textrm{Top}\,}}(C)=\max B$. $\square$

#### Corollary 35

For every closed clustering system $\mathscr {C}$ on *X* that satisfies (L), there is a level-1 phylogenetic network *N* such that $\mathscr {C}_N=\mathscr {C}$. In particular, the unique regular network with clustering system $\mathscr {C}$ is level-1 and phylogenetic in this case.

#### Proof

By Proposition 18, the Hasse diagram ${\mathfrak {H}}[\mathscr {C}]$ is a phylogenetic level-1 network. Since ${\mathfrak {H}}[\mathscr {C}]$ is graph isomorphic to the regular network *N* for $\mathscr {C}$, *N* is also a level-1 phylogenetic network. $\square$

We summarize Corollary 26, Corollaries 28 and 35 in the following characterization of clustering systems that can be derived from level-1 phylogenetic networks.

#### Theorem 8

Let $\mathscr {C}$ be a clustering system. Then, there is a level-1 network *N* such that $\mathscr {C}_N=\mathscr {C}$ if and only if $\mathscr {C}$ is closed and satisfies (L).

We emphasize, however, that there is no 1-to-1 correspondence between level-1 networks and clustering systems. Recall that a network *N* is regular if $\varphi :V\rightarrow V({\mathfrak {H}}[\mathscr {C}_N]):v\mapsto {{\,\mathrm{\texttt{C}}\,}}(v)$ is a graph isomorphism. In contrast to the unique regular network ${\mathfrak {H}}[\mathscr {C}]$, a level-1 network might have shortcuts and thus could even be not semi-regular and, therefore, not regular (cf. Proposition 15 and Theorem 2). Nevertheless, a level-1 network *N* can easily be edited into a level-1 network $N'$ that is isomorphic to ${\mathfrak {H}}[\mathscr {C}_N]$ using two simple operations as specified in Proposition 5.

#### Proposition 19

For every level-1 network *N*, the regular network $N'$ with clustering system $\mathscr {C}_{N'} = \mathscr {C}_N$ is level-1 and can be obtained from *N* by repeatedly removing shortcuts and contracting arcs (*u*, *w*) with ${{\,\textrm{outdeg}\,}}(u)=1$. In particular, $N'$ is the unique least-resolved network w.r.t. $\mathscr {C}_N$ that can be obtained from *N* in this way.

#### Proof

Let *N* be a level-1 network. By Theorem 8, $\mathscr {C}_N$ is closed and satisfies (L). By Corollary 35, therefore, the regular network with clustering system $\mathscr {C}_N$ is level-1. Now, let $N'$ be the network obtained from *N* by repeatedly (1) removing a shortcut and (2) applying ${{\,\mathrm{\textsc {cntr}}\,}}(u,w)$ for an arc (*u*, *w*) with ${{\,\textrm{outdeg}\,}}(u)=1$ until neither operation is possible. By construction, $N'$ is phylogenetic, shortcut-free, and contains no vertex with outdegree 1. It is easy to verify that the removal of shortcuts cannot increase the level of the network. This together with Lemma 12 implies that $N'$ is still level-1. By Lemma 44, $N'$ satisfies (PCC), and thus, it is semi-regular. Theorem 2 now implies that $N'$ is regular. Moreover, by Lemma 1 and Lemma 4, we have $\mathscr {C}_{N}=\mathscr {C}_{N'}$. By Proposition 2, $N'$ is the unique regular network with $\mathscr {C}_{N}=\mathscr {C}_{N'}$. The latter, in particular, implies that the order of the operations “shortcut removal” and “contractions” to obtain $N'$ from *N* does not matter. By Corollary 23, $N'$ is least-resolved. Moreover, a network that still contains a shortcut or an arc (*u*, *w*) with ${{\,\textrm{outdeg}\,}}(u)=1$ cannot be least resolved by Lemma 1 and Lemma 4, respectively. Taken together, the latter arguments imply that $N'$ is the unique least-resolved network w.r.t. $\mathscr {C}_N$ that can be obtained from *N* by repeatedly removing shortcuts and contracting arcs (*u*, *w*) with ${{\,\textrm{outdeg}\,}}(u)=1$. $\square$

As a direct consequence of Theorem 8 and Proposition 19 together with the fact that regular networks are phylogenetic, we obtain

#### Corollary 36

Let $\mathscr {C}$ be a clustering system. Then, there is a phylogenetic level-1 network *N* such that $\mathscr {C}_N=\mathscr {C}$ if and only if $\mathscr {C}$ is closed and satisfies (L).

#### Corollary 37

Let $\mathscr {C}$ be a closed clustering system that satisfies (L). Then, there is a unique shortcut-free phylogenetic level-1 network *N* with $\mathscr {C}_N=\mathscr {C}$ that moreover contains no vertex *v* with ${{\,\textrm{outdeg}\,}}_{N}(v)=1$. This network *N* is regular and least-resolved.

#### Proof

By Corollary 35, the regular network *N* with $\mathscr {C}_N=\mathscr {C}$ is level-1. By Theorem 2, *N* is shortcut-free and contains no vertex *v* with ${{\,\textrm{outdeg}\,}}_{N}(v)=1$. Thus, *N* is phylogenetic. Now, let *N* be a shortcut-free phylogenetic level-1 network with $\mathscr {C}_{N}=\mathscr {C}$ that moreover contains no vertex *v* with ${{\,\textrm{outdeg}\,}}_{N}(v)=1$. By Lemma 44 and Theorem 2, *N* is a regular network, which is unique by Proposition 2. By Corollary 23, *N* is least-resolved. $\square$

Most publications on phylogenetic networks assume that leaves always have indegree 1, see, e.g., Huson et al. (2010).

#### Corollary 38

Let $\mathscr {C}$ be a closed clustering system that satisfies (L). Then, there is a unique shortcut-free phylogenetic level-1 network *N* with $\mathscr {C}_N=\mathscr {C}$ such that every leaf has indegree 1 and all vertices *v* with ${{\,\textrm{outdeg}\,}}_{N}(v)=1$ are adjacent to leaves.

#### Proof

By Corollary 37, there is a unique shortcut-free phylogenetic level-1 network $N'$ with $\mathscr {C}_{N'}=\mathscr {C}$ and for which no vertex has outdegree 1. In $N'$, all vertices with outdegree 0 are leaves. Hence, we can simply apply ${{\,\mathrm{\textsc {expd}}\,}}(x)$ for all leaves *x* with ${{\,\textrm{indeg}\,}}_{N'}(x)>1$. We can repeatedly (i.e., in each expansion step) apply Lemma 5 to conclude that the resulting digraph *N* is a phylogenetic network, Corollary 3 to conclude that *N* is shortcut-free, Lemma 13 to conclude that *N* is level-1 and Lemma 5 to conclude that *N* satisfies $\mathscr {C}_{N}=\mathscr {C}_{N'}=\mathscr {C}$. In particular, every leaf in *N* has indegree 1 by construction and all vertices with outdegree 1 must be adjacent to leaves.

It remains to show that *N* is unique w.r.t. these properties. Let ${\tilde{N}}$ be a phylogenetic shortcut-free level-1 network with $\mathscr {C}_{{\tilde{N}}}=\mathscr {C}$ and such that every leaf has indegree 1 and all vertices *v* with ${{\,\textrm{outdeg}\,}}_{{\tilde{N}}}(v)=1$ are adjacent to leaves. Hence, after application of ${{\,\mathrm{\textsc {cntr}}\,}}(v,x)$ to all vertices *v* with outdegree 1, we obtain a phylogenetic level-1 network ${\tilde{N}}'$ that has no vertex with outdegree 1 at all. Proposition 15 implies that ${\tilde{N}}'$ is regular and Lemma 4 implies that $\mathscr {C}_{\tilde{N}'}=\mathscr {C}_{{\tilde{N}}}=\mathscr {C}$. By Corollary 37, ${\tilde{N}}'\simeq N'$. To obtain $N'$ from ${\tilde{N}}$, we applied precisely the “reversed” operation of the operation to obtain *N* from $N'$, which together with ${\tilde{N}}'\simeq N'$ implies that ${\tilde{N}}\simeq N$. Hence, *N* is the unique network with the desired properties. $\square$

As an immediate consequence, we obtain a characterization of the level-1 networks that are completely determined by the least common ancestor function, and equivalently by their clusters.

#### Proposition 20

Let *N* be a level-1 network without shortcuts. Then, the following statements are equivalent:

(i) $${{\,\textrm{outdeg}\,}}(v)\ne 1$$ for all $$v\in V$$.
(ii) For every $v\in V$ there is a pair of leaves $x,y\in X$ such that $v={{\,\textrm{lca}\,}}(\{x,y\})$.

#### Proof

If ${{\,\textrm{outdeg}\,}}(v)\ne 1$ for all $v\in V$, then Proposition 15 implies that *N* is regular, i.e., $\varphi :V\rightarrow V({\mathfrak {H}}[\mathscr {C}_N]):v\mapsto {{\,\mathrm{\texttt{C}}\,}}(v)$ is a graph isomorphism and thus a bijection. Therefore, ${{\,\mathrm{\texttt{C}}\,}}(u)={{\,\mathrm{\texttt{C}}\,}}(u')$ implies $u=u'$ for all $u,u'\in V$. Together with Eq. (5), i.e., the identity ${{\,\mathrm{\texttt{C}}\,}}(v)={{\,\mathrm{\texttt{C}}\,}}({{\,\textrm{lca}\,}}({{\,\mathrm{\texttt{C}}\,}}(v)))$, we obtain, for all $v\in V$, that $v={{\,\textrm{lca}\,}}({{\,\mathrm{\texttt{C}}\,}}(v))$ and thus, by Lemma 50, there is a pair of leaves $x,y\in X$ such that $v={{\,\textrm{lca}\,}}({{\,\mathrm{\texttt{C}}\,}}(v))={{\,\textrm{lca}\,}}(\{x,y\})$. Conversely, suppose there is a vertex $v\in V$ with a unique child *w*. Moreover, assume for contradiction that there leaves $x,y\in X$ such that $v={{\,\textrm{lca}\,}}(\{x,y\})$. Using Observation 5, we have $\{x,y\}\subseteq {{\,\mathrm{\texttt{C}}\,}}(v)={{\,\mathrm{\texttt{C}}\,}}(w)$. Together with $w\prec _N v$, this contradicts $v={{\,\textrm{lca}\,}}(\{x,y\})$. $\square$

Finally, we show that every closed clustering system satisfying (L) is represented by a unique “minimal” separated level-1 network. More precisely, we have

#### Proposition 21

Let $\mathscr {C}$ be a closed clustering system satisfying (L). Then, there is a unique separated phylogenetic shortcut-free level-1 network *N* with $\mathscr {C}=\mathscr {C}_N$. The network *N* is obtained from the unique regular network ${\mathfrak {H}}[\mathscr {C}]$ by applying ${{\,\mathrm{\textsc {expd}}\,}}(v)$ to all hybrid vertices.

#### Proof

By Corollary 37, the unique regular network ${\mathfrak {H}}[\mathscr {C}]$ is a level-1 network. By Theorem 6, there is a unique semi-regular separated phylogenetic network *N* with $\mathscr {C}=\mathscr {C}_N$, and this network is obtained from ${\mathfrak {H}}[\mathscr {C}]$ by applying ${{\,\mathrm{\textsc {expd}}\,}}(v)$ to all hybrid vertices. The latter and Lemma 13 imply that *N* is also level-1. Since *N* is semi-regular, it is shortcut-free. Hence, a network with the desired properties exists. To see that *N* is unique, let $\tilde{N}$ be a separated phylogenetic shortcut-free level-1 network $\tilde{N}$ with $\mathscr {C}=\mathscr {C}_{\tilde{N}}$. By Lemma 44, the shortcut-free network $\tilde{N}$ satisfies (PCC), and thus, it is semi-regular. In summary, $\tilde{N}$ is a semi-regular separated phylogenetic network with clustering system $\mathscr {C}$ which is unique by Theorem 6. $\square$

### Compatibility of clustering systems and intersection closure

A frequent task in phylogenetics is the construction of networks based on partial information of putative networks, e.g., subtrees (Aho et al. 1981; Jansson et al. 2006; Van Iersel et al. 2009; Jansson and Sung 2006; van Iersel and Kelk 2011), subnetworks (Huber et al. 2017; Van Iersel et al. 2017a; Semple and Toft 2021), metrics or full information about clusters (Gambette and Huber 2012). A property or properties of networks can be thought of as a subset $\mathbb {P}$ of the set of all rooted DAGs such that *N* has property $\mathbb {P}$ whenever $N\in \mathbb {P}$. In this case we simply call *N* a $\mathbb {P}$-network. A clustering system $\mathscr {C}\subseteq 2^X$ is *compatible w.r.t.* $\mathbb {P}$-*networks* if there is $\mathbb {P}$-network *N* on *X* such that $\mathscr {C}\subseteq \mathscr {C}_N$.

#### Problem 1

Is a given clustering system $\mathscr {C}\subseteq 2^X$ compatible w.r.t. to (separated, phylogenetic) level-*k* networks?

We show that this question can easily be answered for level-1 networks by computing the so-called intersection closure (Bandelt and Dress 1989). To be more precise, to every clustering system $\mathscr {C}$ one can associate the set $\mathcal {I}(\mathscr {C})$ consisting of all non-empty intersections of an arbitrary subset of clusters in $\mathscr {C}$. Note that $A\in \mathscr {C}$ implies $A\cap A = A\in \mathcal {I}(\mathscr {C})$ and so $\mathscr {C}\subseteq \mathcal {I}(\mathscr {C})$. Recall that a clustering system satisfying (L) is in particular a weak hierarchy (cf. Corollary 29). In this case, only pairwise intersections need to be considered since the intersection of arbitrary subset of clusters coincides with a pairwise intersection. As an immediate consequence, we have

#### Observation 15

Let $\mathscr {C}$ be a clustering system satisfying (L). Then, $\mathcal {I}(\mathscr {C})=\mathscr {C} \cup \{C\cap C' \mid C,C'\in \mathscr {C} \text { overlap}\}$.

Lemma 1 of Bandelt and Dress (1989) asserts that $\mathscr {C}$ is a weak hierarchy if and only if $\mathcal {I}(\mathscr {C})$ is a weak hierarchy. We use this fact to prove an analogous result for property (L).

#### Lemma 65

A clustering system $\mathscr {C}$ satisfies (L) if and only if $\mathcal {I}(\mathscr {C})$ satisfies (L).

#### Proof

Since (L) is a hereditary property, it suffices to show that if $\mathscr {C}$ satisfies (L), then $\mathcal {I}(\mathscr {C})$ also satisfies (L). We show first that the intersection of two overlapping clusters in $\mathscr {C}$ cannot overlap any other cluster of $\mathscr {C}$. To this end, consider $C_1,C_2,C_3\in \mathscr {C}$, and suppose that $C_1$ and $C_2$ overlap and $(C_1\cap C_2)\cap C_3\ne \emptyset$. Then, one easily verifies that either $C_3\subseteq C_1\cap C_2$, $C_1\cup C_2\subseteq C_3$, or $C_3$ overlaps at least one of $C_1$ and $C_2$. In the latter case, (L) implies $C_1\cap C_3=C_1\cap C_2$ or $C_2\cap C_3=C_1\cap C_2$, and thus, $(C_1\cap C_2)\cap C_3= C_1\cap C_2$. That is, the intersection of two overlapping clusters in $\mathscr {C}$ cannot overlap any other cluster of $\mathscr {C}$. It remains to show that the intersection $C_1\cap C_2$ of an overlapping pair of clusters $C_1,C_2\in \mathscr {C}$ also cannot overlap with the intersection $C_3\cap C_4$ of another overlapping pair $C_3,C_4\in \mathscr {C}$. Assume, for contradiction, that $C_1\cap C_2$ and $C_3\cap C_4$ overlap. Hence, we have $(C_1\cap C_2)\cap C_3\ne \emptyset$ and $(C_1\cap C_2)\cap C_4\ne \emptyset$ and also $C_3{\setminus } (C_1\cap C_2)\ne \emptyset$ and $C_4{\setminus } (C_1\cap C_2)\ne \emptyset$. Moreover, $C_1\cap C_2 \subseteq C_3$ and $C_1\cap C_2 \subseteq C_4$ are not possible at the same time since otherwise $C_1\cap C_2 \subseteq C_3 \cap C_4$. Hence, $C_1\cap C_2$ overlaps at least one of $C_3$ and $C_4$, a contradiction. In summary, all overlapping pairs $C',C''\in \mathcal {I}(\mathscr {C})$ are formed by clusters $C',C''\in \mathscr {C}$, and thus, $\mathcal {I}(\mathscr {C})$ also satisfies (L). $\square$

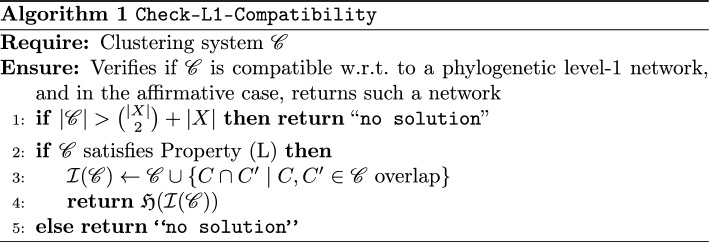



#### Theorem 9

Let $\mathscr {C}\subseteq 2^X$ be a clustering system. Then, Check-L1-Compatibility correctly verifies if there is a (separated, phylogenetic) level-1 network on *X* such that $\mathscr {C}\subseteq \mathscr {C}_N$ and can be implemented to run in $O(\vert \mathscr {C}\vert ^2\vert X\vert )\subseteq O(\vert X\vert ^5)$ time. Moreover, such a network *N* can be constructed in $O(\vert X\vert ^5)$ time.

#### Proof

The proof (in particular, the part concerning the time complexity) is rather lengthy and technical and is, therefore, placed to Sect. 10.3 in “Appendix.” We emphasize, that the proof, however, contains interesting insights for those readers who want to implement algorithm. $\square$

#### Theorem 10

For every clustering system $\mathscr {C}$ the following statements are equivalent:

1. $\mathscr {C}$ is compatible w.r.t. to a (separated, phylogenetic) level-1 network;
2. There is a (separated, phylogenetic) level-1 network with $\mathscr {C}_N = \mathcal {I}(\mathscr {C})$;
3. $$\mathscr {C}$$ satisfies Property (L).
4. ${\mathfrak {H}}[\mathcal {I}(\mathscr {C})]$ is a level-1 network.

#### Proof

If Statement (1) is satisfied, then the network computed with Check-L1-Compatibility is a network with $\mathscr {C}_N = \mathcal {I}(\mathscr {C}),$ and thus, Statement (2) holds. If there is a (separated, phylogenetic) level-1 network with $\mathscr {C}_N = \mathcal {I}(\mathscr {C})$, then Theorem 8 implies that $\mathcal {I}(\mathscr {C})$ satisfies (L). Since (L) is a hereditary property, $\mathscr {C}$ must satisfy (L) as well. Hence, (2) implies (3). Assume that $\mathscr {C}$ satisfies Property (L). By Lemma 65, $\mathcal {I}(\mathscr {C})$ satisfies (L) and, by definition, $\mathcal {I}(\mathscr {C})$ is closed. By Theorem 8 and Proposition 21, there is a (separated, phylogenetic) level-1 network such that $\mathcal {I}(\mathscr {C})=\mathscr {C}_N$. Since $\mathscr {C}\subseteq \mathcal {I}(\mathscr {C})= \mathscr {C}_N$, Item (1) is satisfied. Hence, Statements (1), (2) and (3) are equivalent. Assume that Statement (2) holds. By Theorem 8, $\mathcal {I}(\mathscr {C})$ is closed and satisfies (L). Corollary 37 implies that ${\mathfrak {H}}[\mathcal {I}(\mathscr {C})]$ is a level-1 network and thus Statement (4) holds. Conversely, assume that Statement (4) is satisfied. Again, by Theorem 8, $\mathcal {I}(\mathscr {C})$ is closed and satisfies (L). Proposition 21 implies now Statement (2). In summary, the four statements are equivalent. $\square$

## Special subclasses of level-1 networks

### Galled trees

In level-1 networks, the structure a block *B* is highly constrained if the unique terminal vertex *v* of *B* has only two parents $v_1$ and $v_2$. The absence of additional hybrid vertices implies, in particular, that the two paths from $\max B$ to $v_1$ and $v_2$ are uniquely defined.

#### Observation 16

Let *N* be a level-1 network. Then, every non-trivial block is an (undirected) cycle if and only if every hybrid vertex *v* in *N* satisfies ${{\,\textrm{indeg}\,}}(v)=2$.

**Fig. 17:**
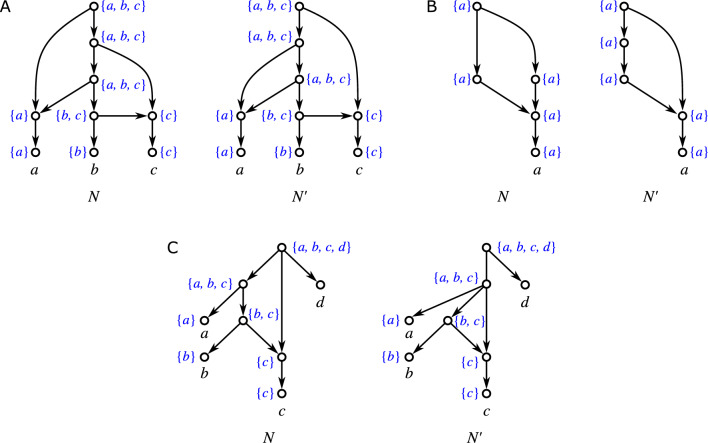
Three pairs of non-isomorphic networks *N* and $N'$ for which $\mathscr {M}_{N} = \mathscr {M}_{N'}$. **A** *N* and $N'$ are binary level-2 tree-child networks. **B** *N* and $N'$ are separated galled trees but not phylogenetic. **C** *N* and $N'$ are separated phylogenetic level-1 networks

We note that a similar result does not hold for level-*k* networks with $k>1$. As an example, Fig. 17A shows two networks whose hybrid vertices have all indegree 2 but whose blocks are not (undirected) cycles.

#### Lemma 66

If *N* is a level-1 network and every hybrid vertex *v* in *N* satisfies ${{\,\textrm{indeg}\,}}(v)=2$, then *N* is outerplanar.

#### Proof

By Observation 16, every non-trivial block on *N* is a cycle. Therefore, the underlying undirected graph of *N* does not contain a subdivision of the graph $K_4$, i.e., the complete graph on 4 vertices, nor of the complete bipartite graph $K_{2,3}$. By Theorem 1 in Chartrand and Harary (1967), *N* is outerplanar. $\square$

In Gusfield et al. (2003), *galled trees* were introduced as phylogenetic networks in which all cycles are vertex disjoint. Here, we consider a more general version, where cycles are allowed to share a cut vertex and the network is not required to be phylogenetic. More constrained types of networks will be discussed in the subsequent sections.

#### Definition 26

A *galled tree* is a network in which every non-trivial block is an (undirected) cycle.

#### Lemma 67

Every galled tree is level-1.

#### Proof

Let *N* be a galled tree. Every trivial block *B* contains at most one hybrid vertex distinct from $\max B$. Thus, consider a non-trivial block *B* and assume, for contradiction, that *B* properly contains two hybrid vertices *h* and $h'$. Since *B* is a cycle, the two vertices in *B* that are adjacent with *h* must exactly be the two in-neighbors of *h*. The same holds for *h*. It is now easy to see that the two path in *B* that connect *h* and $h'$ must each contain a vertex whose two neighbors in the cycle *B* are out-neighbors. Hence, these two distinct vertices are $\preceq _{N}$-maximal in *B*, which contradicts the uniqueness of $\max B$. $\square$

Lemma 67 and Observation 16 imply

#### Corollary 39

A network is a galled tree if and only if it is level-1 and satisfies ${{\,\textrm{indeg}\,}}(v)=2$ for all hybrid vertices *v*.

#### Observation 17

In a galled tree, every non-trivial block consists of two internally vertex disjoint paths connecting $\max B$ and $\min B$. Moreover, every vertex contained in *B* that is distinct from $\max B$ and $\min B$ has precisely one out-neighbor in *B*.

As we shall see below, this implies that its clustering system satisfies the following property:

#### Definition 27

(N3O): $\mathscr {C}$ contains no three distinct pairwise overlapping clusters.

#### Lemma 68

If $\mathscr {C}$ is the clustering system of a galled tree, then $\mathscr {C}$ satisfies (N3O).

#### Proof

Suppose there is a galled tree *N* with $\mathscr {C}_{N}=\mathscr {C}$. In particular, *N* is level-1 by Lemma 67. Now, suppose, for contradiction, that (N3O) is not satisfied. Thus, there are three distinct vertices $u_1,u_2,u_3\in V(N)$ such that $C_1{:}{=}{{\,\mathrm{\texttt{C}}\,}}_N(u_1)$, $C_2{:}{=}{{\,\mathrm{\texttt{C}}\,}}_N(u_2)$, and $C_3{:}{=}{{\,\mathrm{\texttt{C}}\,}}_N(u_3)$ overlap pairwise. By Lemma 17, it must hold that $u_1$, $u_2$, and $u_3$ are pairwise $\preceq _{N}$-incomparable. By Lemma 48, $C_1\cap C_2\ne \emptyset$ implies that $u_1$ and $u_2$ are located in a common block *B*. Clearly, $\preceq _{N}$-incomparability of $u_1$ and $u_2$ implies $u_1\ne \max B$. By similar arguments, $u_1$ and $u_3$ are located in a common block $B'$ and $u_1\ne \max B'$. Since $u_1\notin \{\max B, \max B'\}$, we can apply Lemma 9 to conclude that $B=B'$. Hence, for every $i\in \{1,2,3\}$, there is a directed path $P_i$ in *B* from $u_i$ to $\min B$. Now, consider, for distinct $i,j\in \{1,2,3\}$, the $\preceq _{N}$-maximal vertex *v* in $P_i$ that is also a vertex in $P_j$ (which exists since $\min B$ is a vertex of both paths). We have $v\notin \{u_i, u_j\}$ because $u_i$ and $u_j$ are $\preceq _N$-incomparable. Therefore, the unique parents $v_i$ and $v_j$ of *v* in $P_i$ and $P_j$, resp., must be distinct. Therefore, *v* is a hybrid vertex in *B* and clearly distinct from $\max B$. Hence, it must hold that $v=\min B$. Since *i* and *j* were chosen arbitrarily, the paths $P_1$, $P_2$, and $P_3$ only have vertex $\min B$ in common. This together with the fact that $\min B\notin \{u_1,u_2,u_3\}$ implies that $\min B$ has at least indegree 3. By Observation 16, therefore, *N* has a non-trivial block that is not an undirected cycle. Hence, *N* is not a galled tree, a contradiction. $\square$

The converse of Lemma 68 is not true since, in addition to (N3O), closedness and (L) are required:

#### Theorem 11

$\mathscr {C}$ is the clustering system of a galled tree if and only if $\mathscr {C}$ is closed and satisfies (L) and (N3O). Moreover, in this case, ${\mathfrak {H}}[\mathscr {C}]$ is a phylogenetic galled tree.

#### Proof

Suppose first that *N* is the clustering system of a shortcut-free galled tree. By Lemma 67, *N* is level-1, and thus, it is closed and $\mathscr {C}$ satisfies (L) by Theorem 8. By Lemma 68, $\mathscr {C}$ also satisfies (N3O). Now, suppose, that $\mathscr {C}$ is closed, satisfies (*L*), and does not contain three pairwise overlapping clusters. By Corollary 37, the unique regular network $N{:}{=}{\mathfrak {H}}[\mathscr {C}]$ with clustering system $\mathscr {C}$ is a shortcut-free phylogenetic level-1 network. Hence, it satisfies (PCC) by Lemma 44. Suppose, for contradiction that *N* is not a galled tree, i.e., it contains a non-trivial block, that is not an undirected cycle. By Observation 16, there is a hybrid vertex $w\in V(N)$ with (at least) three distinct in-neighbors $u_1$, $u_2$, and $u_3$. By Observation 3 and since *N* is shortcut-free, $u_1$, $u_2$, and $u_3$ must be pairwise $\preceq _{N}$-incomparable. By (PCC), it therefore holds ${{\,\mathrm{\texttt{C}}\,}}_N(u_i)\not \subseteq {{\,\mathrm{\texttt{C}}\,}}_{N}(u_j)$ for all distinct $i,j\in \{1,2,3\}$. Moreover, it holds $\emptyset \ne {{\,\mathrm{\texttt{C}}\,}}_{N}(w)\subseteq {{\,\mathrm{\texttt{C}}\,}}_{N}(u_i)$ for $i\in \{1,2,3\}$. Taken together, the latter two arguments imply that ${{\,\mathrm{\texttt{C}}\,}}_{N}(u_1)$, ${{\,\mathrm{\texttt{C}}\,}}_N(u_2)$, and ${{\,\mathrm{\texttt{C}}\,}}_{N}(u_3)$ overlap pairwise, a contradiction. Hence, *N* must be a galled tree. $\square$

#### Definition 28

Diday (1986); Bertrand and Diatta (2013) A clustering system $(X,\mathscr {C})$ is *pre-pyramidal* if there exists a total order $\lessdot$ on *X* such that, for every $C\in \mathscr {C}$ and all $x,y\in C$, it holds that $x\lessdot u\lessdot y$ implies $u\in C$. That is, all clusters $C\in \mathscr {C}$ are intervals w.r.t. $\lessdot$.

A necessary condition (Nebeský 1983; Changat et al. 2022) for $\mathscr {C}$ to be pre-pyramidal is

(WP): If $C_1,C_2,C_3\in \mathscr {C}$ have pairwise non-empty intersections, then one of the three sets is contained in the union of the other two.

Taken together, (L) and (WP) imply (N3O). More precisely, we have

#### Lemma 69

Let $\mathscr {C}$ be a pre-pyramidal clustering system satisfying (L). Then, $\mathscr {C}$ satisfies (N3O), i.e., there are no three pairwise overlapping sets.

#### Proof

Assume, for contradiction, that $C_1$, $C_2$, and $C_3$ overlap pairwise. Then, (L) implies that $C_1\cap C_2 = C_2\cap C_3 = C_1\cap C_3 = C_1\cap C_2\cap C_3{=}{:}C\ne \emptyset$. Since $\mathscr {C}$ is pre-pyramidal and the three pairwise intersections are non-empty, (WP) implies that one of the three sets is contained in the union of the other two. W.l.o.g., suppose $C_1\subseteq C_2\cup C_3$. Equivalently, $C_1 = C_1\cap (C_2\cup C_3)=(C_1\cap C_2)\cup (C_1\cap C_3)=C\subsetneq C_1$, a contradiction. $\square$

Pre-pyramidal set systems are also known as “interval hypergraphs.” A characterization in terms of an infinite series of forbidden subhypergraphs has been developed in Tucker (1972); Trotter and Moore (1976); Duchet (1984). It can be used to obtain a simple necessary and condition in the presence of (L).

#### Proposition 22

Let $\mathscr {C}$ be a clustering system satisfying (L). Then, $\mathscr {C}$ is pre-pyramidal if and only if it satisfies (N3O).

#### Proof

Starting from Duchet (1984, Theorem 7.2) one observed that condition (L) excludes all induced forbidden subhypergraphs with a single exception. The remaining configuration, called $M_1$ in (Fig. 11 Duchet 1984), comprises three pairwise overlapping sets that share at least one common point. Thus, if (N3O) holds, no $M_1$-subhypergraph is present in $\mathscr {C}$. Since (L) and (N3O) together exclude all forbidden subhypergraphs and thus $\mathscr {C}$ is pre-pyramidal. Lemma 69 now completes the proof. $\square$

#### Theorem 12

Let *N* be a phylogenetic shortcut-free level-1 network with clustering system $\mathscr {C}$. Then, $\mathscr {C}$ is pre-pyramidal if and only if ${{\,\textrm{indeg}\,}}(v)\le 2$ for all $v\in V$, i.e., if and only if *N* is a galled tree.

#### Proof

($\implies$) Suppose that $\mathscr {C}$ is pre-pyramidal with corresponding total order $\lessdot$ of *X* and, moreover, assume, for contradiction, that *w* is hybrid vertex with ${{\,\textrm{indeg}\,}}_N(w)\ge 3$. Hence, let $u_1,u_2,u_3\in {{\,\textrm{par}\,}}_N(w)$ be pairwise distinct. Since *N* is shortcut-free, Observation 3 implies that $u_1$, $u_2$, and $u_3$ are pairwise $\preceq _{N}$-incomparable. Together with Lemma 44, this implies ${{\,\mathrm{\texttt{C}}\,}}(u_i)\not \subseteq {{\,\mathrm{\texttt{C}}\,}}(u_j)$ for distinct $i,j \in \{1,2,3\}$. Moreover, $u_1,u_2,u_3\in {{\,\textrm{par}\,}}_N(w)$ and Lemma 17 yield $\emptyset \ne {{\,\mathrm{\texttt{C}}\,}}(w)\subseteq {{\,\mathrm{\texttt{C}}\,}}(u_i)$ for all $i \in \{1,2,3\}$, i.e., $\mathscr {C}$ contains three pairwise overlapping clusters. On the other hand, $\mathscr {C}$ satisfies (L) by Corollary 28, thus Lemma 69 implies that $\mathscr {C}$ cannot contain three pairwise overlapping clusters, a contradiction.

$( \Leftarrow )$ Suppose ${{\,\textrm{indeg}\,}}(v)\le 2$ for all $v\in V$. By Observation 16, this holds if and only if *N* is a galled tree. By Lemma 67, Corollary 28, and Lemma 68, $\mathscr {C}$ satisfies (L) and (N3O). Hence, $\mathscr {C}$ is pre-pyramidal by Proposition 22. $\square$

#### Definition 29

Bertrand (2008) A clustering system $\mathscr {C}$ is a *paired hierarchy* if a cluster $C\in \mathscr {C}$ overlaps with at most one other cluster in $\mathscr {C}$.

#### Observation 18

Every hierarchy is a paired hierarchy and every paired hierarchy satisfies (L) and (N30).

#### Proposition 23

Let $\mathscr {C}$ be a closed clustering system. Then, $\mathscr {C}$ is a paired hierarchy if and only if there is a shortcut-free phylogenetic galled tree *N* with $\mathscr {C}_N=\mathscr {C}$ where all non-trivial blocks consist of four vertices.

#### Proof

Suppose first that $\mathscr {C}$ is a paired hierarchy. Since $\mathscr {C}$ satisfies (L) and (N3O) by Observation 18 and is closed, we can apply Theorem 11 to conclude that $N {:}{=}{\mathfrak {H}}[\mathscr {C}]$ is a shortcut-free phylogenetic galled tree with $\mathscr {C}_{N} = \mathscr {C}$. Since *N* is, in particular, a phylogenetic level-1 network (cf. Lemma 67), Lemma 44 implies that *N* satisfies (PCC). By definition, every non-trivial block contains at least 3 vertices. If a block *B* would contain exactly three vertices, then one easily sees that *N* contains the shortcut $(\max B, \min B)$, a contradiction. Hence, every non-trivial block in *N* contains at least 4 vertices. Assume, for contradiction, that *N* contains a non-trivial block *B* with at least $k\ge 5$ vertices. Note, *B* refers to an (undirected) cycle in *N*. Hence, there are two internal vertex disjoint paths in *B* connecting $\max B$ and $\min B$. Since *N* is shortcut-free and $k\ge 5$, we can conclude that one path contains a vertex *v* and the other path contains vertices $u_1,u_2$ that are all distinct from $\max B$ and $\min B$. Since *B* is an undirected cycle, one easily verifies that *v* and $u_1$ as well as *v* and $u_2$ are $\preceq _N$-incomparable. By Lemma 17, we have $\emptyset \ne {{\,\mathrm{\texttt{C}}\,}}(\min B) \subseteq {{\,\mathrm{\texttt{C}}\,}}(v),{{\,\mathrm{\texttt{C}}\,}}(u_1),{{\,\mathrm{\texttt{C}}\,}}(u_2)$. This together with (PCC) and the fact that *v* and $u_1$ as well as *v* and $u_2$ are $\preceq _N$-incomparable implies that ${{\,\mathrm{\texttt{C}}\,}}(v)$ must overlap with both ${{\,\mathrm{\texttt{C}}\,}}(u_1)$ and ${{\,\mathrm{\texttt{C}}\,}}(u_2)$, a contradiction.

Assume now that there is a shortcut-free phylogenetic galled tree *N* with $\mathscr {C}_N=\mathscr {C}$ where all non-trivial blocks consists of four vertices. Suppose, for contradiction, ${{\,\mathrm{\texttt{C}}\,}}(v)\in \mathscr {C}$ overlaps with two distinct clusters ${{\,\mathrm{\texttt{C}}\,}}(u_2)$ and ${{\,\mathrm{\texttt{C}}\,}}(u_2)$ in $\mathscr {C}$. Note, *v*, $u_1$, and $u_2$ must be pairwise distinct. By Lemma 19, we have $v,u_1\in B_1^0$ and $v,u_2\in B_2^0$ for non-trivial blocks $B_1$ and $B_2$ in *N*. In particular, we have $v, u_1\notin \{\min B_1,\max B_1\}$ and $v, u_2\notin \{\min B_2,\max B_2\}$. We can therefore apply Lemma 9 to conclude that $B_1=B_2{=}{:}B$. In particular, *B* contains at least five pairwise distinct vertices $\min B$, $\max B$, *v*, $u_1$, and $u_2$, a contradiction. $\square$

It is worth noting that for paired hierarchies, and in particular also for hierarchies, $\mathscr {C}$ there are not only galled trees but also shortcut-free and phylogenetic level-*k* networks *N* that are not level-$(k-1)$ with $\mathscr {C}_N=\mathscr {C}$. Figure 12A serves as an example.

### Conventional and separated level-1 networks

The literature on phylogenetic networks often stipulates that the leaves $v\in X$ have indegree 1, see, e.g., Huson et al. (2010). Furthermore, level-1 networks are often defined such that every non-trivial block has exactly one hybrid vertex.

#### Definition 30

A network *N* is *conventional* if (i) all leaves have indegree at most 1 and (ii) every hybrid vertex is contained in a unique non-trivial block.

We remark that if $\vert X\vert >1$ all leaves have indegree 1 in a conventional network. In Fig. 4, network *N* is conventional, while $N'$ is not.

#### Proposition 24

Let $\mathscr {C}$ be a closed clustering system on *X* satisfying (L). Then, ${\mathfrak {H}}[\mathscr {C}]$ is conventional if and only if $\mathcal {B}^0(\{x\})=\emptyset$ for all $x\in X$ and $\mathcal {B}^0({{\,\textrm{Top}\,}}(C))=\emptyset$ for all $C\in \mathscr {C}$ with $\mathcal {B}^0(C)\ne \emptyset$.

#### Proof

By Lemma 22, ${\mathfrak {H}}{:}{=}{\mathfrak {H}}[\mathscr {C}]$ is a phylogenetic network with leaf set $X_{{\mathfrak {H}}}{:}{=}\{ \{x\} \mid x \in X \}$. Moreover, Lemma 60 implies that ${{\,\textrm{indeg}\,}}(\{x\})_{{\mathfrak {H}}}\ge 2$ if and only if $\mathcal {B}^0(\{x\})\ne \emptyset$. By Observation 1 and Lemma 9, two distinct non-trivial blocks *B* and $B'$ share at most one vertex $v\in \{\max B,\max B'\}$. Thus, every hybrid vertex is contained in a unique non-trivial block if and only if $\max B\ne \min B'$ for any pair of non-trivial blocks. Since the non-trivial blocks in ${\mathfrak {H}}[\mathscr {C}]$ are exactly the blocks $\mathcal {B}(C)\ne \emptyset$, this is equivalent to requiring that for every $C\in \mathscr {C}$ with $\mathcal {B}(C)\ne \emptyset$ we have ${{\,\textrm{Top}\,}}(C)$ is not the minimum of another non-trivial block, i.e., $\mathcal {B}({{\,\textrm{Top}\,}}(C))=\emptyset$. $\square$

#### Proposition 25

If *N* is separated, then *N* is conventional.

#### Proof

Suppose *N* separated. Then, all leaves in *N* must have indegree at most 1 since they have outdegree 0 and, by definition, hybrid vertices have outdegree 1. By Lemma 9, a vertex *v* that is contained in two non-trivial blocks *B* and $B'$ must be the unique maximal vertex of one of them. In this case, Corollary 4 implies ${{\,\textrm{outdeg}\,}}_{N}(v)\ge 2$. However, since *v* is hybrid vertex in a separated network, we have ${{\,\textrm{outdeg}\,}}(v)=1$. Therefore, such a hybrid vertex *v* that is contained in two non-trivial blocks cannot exist. Hence, *N* is conventional. $\square$

### Binary level-1 networks

Recall that a network is *binary* if it is phylogenetic, separated, and all vertices have in- and outdegree at most 2. Equivalently, in a binary network, every tree vertex is either a leaf or has exactly two children, and every hybrid vertex has exactly two parents and one child. As an immediate consequence of the definition, Proposition 9, Proposition 25, and Corollary 39, we have:

#### Observation 19

Binary level-1 networks are always phylogenetic, separated, conventional, tree-child, galled trees.

#### Lemma 70

Let *N* be a binary level-1 network. Then, ${\mathfrak {H}}[\mathscr {C}_N]\simeq N$ if and only if *N* is a tree.

#### Proof

Let *N* be a binary level-1 network. By definition, *N* is phylogenetic. If *N* is a tree, then ${\mathfrak {H}}[\mathscr {C}_N]\sim N$ (cf. Corollary 9). Assume now that *N* is not a tree and thus, *N* contains hybrid vertices all with outdegree 1 in *N*. However, Proposition 15 implies that ${\mathfrak {H}}[\mathscr {C}]$ does not contain any vertex with outdegree 1. Consequently, ${\mathfrak {H}}[\mathscr {C}_N]\not \sim N$. $\square$

Hence, ${\mathfrak {H}}[\mathscr {C}_N]$ can never be binary in case $\mathscr {C}_N$ contains overlapping clusters.

#### Definition 31

(2-Inc) A clustering system $\mathscr {C}$ has Property (2-Inc) if, for all clusters $C\in \mathscr {C}$, there are at most two inclusion-maximal clusters $A,B\in \mathscr {C}$ with $A,B\subsetneq C$ and at most two inclusion-minimal clusters $A,B\in \mathscr {C}$ with $C\subsetneq A,B$.

#### Lemma 71

Let *N* be a binary network that satisfies (PCC). Then, $\mathscr {C}_N$ satisfies (2-Inc).

#### Proof

Let *N* be a binary network on *X* with clustering system $\mathscr {C}$. Assume, for contradiction, that $\mathscr {C}_N$ does not satisfy Property (2-Inc) for some cluster $C\in \mathscr {C}_N$.

Assume first that there are (at least) three inclusion-minimal clusters $A_1,A_2,A_3\in \mathscr {C}$ that satisfy $C\subsetneq A_1,A_2,A_3$. Hence, $C\ne X$. Since $C\in \mathscr {C}_N$, there is a $\preceq _N$-maximal vertex $v\in V(N)$ with ${{\,\mathrm{\texttt{C}}\,}}(v) = C$. Note, *v* has at least one but at most two parents in *N* since *N* is binary and $v\ne \rho _N$. Let $v_i$ be a vertex in *N* with ${{\,\mathrm{\texttt{C}}\,}}(v_i) = A_i$, $i\in \{1,2,3\}$. By Observation 7, we have $v\prec _N v_1,v_2, v_3$. Therefore, and because *v* has at most two parents, at least two of $v_1,v_2,v_3$ must be ancestors of the same parent *w* of *v* in *N*. W.l.o.g. assume that $w\preceq _N v_1,v_2$. Since *v* is $\preceq _N$-maximal w.r.t. ${{\,\mathrm{\texttt{C}}\,}}(v) = C$, it must hold that ${{\,\mathrm{\texttt{C}}\,}}(v)\subsetneq {{\,\mathrm{\texttt{C}}\,}}(w)$. Lemma 17 implies that ${{\,\mathrm{\texttt{C}}\,}}(w)\subseteq {{\,\mathrm{\texttt{C}}\,}}(v_1),{{\,\mathrm{\texttt{C}}\,}}(v_2)$. Note, however, that ${{\,\mathrm{\texttt{C}}\,}}(w) = {{\,\mathrm{\texttt{C}}\,}}(v_1)$ is not possible, since then $A_1\ne A_2$ and ${{\,\mathrm{\texttt{C}}\,}}(v)\subsetneq {{\,\mathrm{\texttt{C}}\,}}(w)$ imply that ${{\,\mathrm{\texttt{C}}\,}}(v)\subsetneq {{\,\mathrm{\texttt{C}}\,}}(w) = {{\,\mathrm{\texttt{C}}\,}}(v_1) \subsetneq {{\,\mathrm{\texttt{C}}\,}}(v_2)$, a contradiction to the inclusion-minimality of ${{\,\mathrm{\texttt{C}}\,}}(v_2)=A_2$. By similar arguments, ${{\,\mathrm{\texttt{C}}\,}}(w) = {{\,\mathrm{\texttt{C}}\,}}(v_2)$ is not possible. Hence, ${{\,\mathrm{\texttt{C}}\,}}(v)\subsetneq {{\,\mathrm{\texttt{C}}\,}}(w) \subsetneq {{\,\mathrm{\texttt{C}}\,}}(v_1), {{\,\mathrm{\texttt{C}}\,}}(v_2)$ must hold, again a contradiction to the inclusion-minimality of ${{\,\mathrm{\texttt{C}}\,}}(v_1)=A_1$ and ${{\,\mathrm{\texttt{C}}\,}}(v_2)=A_2$.

Assume now that there are (at least) three inclusion-maximal clusters $A_1,A_2,A_3\in \mathscr {C}$ that satisfy $A_1,A_2,A_3\subsetneq C$. Hence, *C* cannot be a singleton. Since $C\in \mathscr {C}_N$, there is a $\preceq _N$-minimal vertex $v\in V(N)$ with ${{\,\mathrm{\texttt{C}}\,}}(v) = C$. Since *C* is not a singleton and *N* is binary, we can conclude that *v* has at least one but at most two children in *N*. Let $v_i$ be a vertex in *N* with ${{\,\mathrm{\texttt{C}}\,}}(v_i) = A_i$, $i\in \{1,2,3\}$. Since *v* has at most two children, at least two of $v_1,v_2,v_3$ must be descendants of the same child *w* of *v* in *N*. Since *v* is $\preceq _N$-minimal w.r.t. ${{\,\mathrm{\texttt{C}}\,}}(v) = C,$ it must hold that ${{\,\mathrm{\texttt{C}}\,}}(w)\subsetneq {{\,\mathrm{\texttt{C}}\,}}(v)$. Now, we can apply similar arguments as in the first case to obtain a contradiction. $\square$

#### Theorem 13

Let $\mathscr {C}$ be a clustering system on *X*. Then, there is a binary level-1 network *N* with $\mathscr {C}_N = \mathscr {C}$ if and only if $\mathscr {C}$ is closed and satisfies Properties (L) and (2-Inc). In this case, the (unique) cluster network with clustering system $\mathscr {C}$ is a binary level-1 network.

#### Proof

Assume first that $\mathscr {C}$ is closed and satisfies Properties (L) and (2-Inc). Taken Theorem 8 and Proposition 19 together, the regular network ${\mathfrak {H}}[\mathscr {C}]$ is a level-1 network. Since $\mathscr {C}$ satisfies (2-Inc), every vertex in ${\mathfrak {H}}[\mathscr {C}]$ must have in- and outdegree at most 2. By Theorem 6, we can uniquely construct a cluster network *N* by applying ${{\,\mathrm{\textsc {expd}}\,}}(v)$ to all hybrid vertices of ${\mathfrak {H}}[\mathscr {C}]$. Hence, *N* is binary. Moreover, since ${\mathfrak {H}}[\mathscr {C}]$ is a level-1 network, Lemma 13 implies that *N* is a level-1 network. Hence, a binary level-1 network *N* with $\mathscr {C}_N = \mathscr {C}$ exists. The latter, in particular, shows that the cluster network *N* is a binary level-1 network. Conversely, suppose that *N* is a binary level-1 network on *X* with $\mathscr {C}_N = \mathscr {C}$. By Theorem 8, $\mathscr {C}$ is closed and satisfies Property (L). By Lemma 44, *N* satisfies (PCC) and thus, by Lemma 71, is also satisfies (2-Inc). $\square$

Since a phylogenetic level-1 network satisfies (PCC) by Lemma 44, it is semi-regular if and only if it is shortcut-free. Theorem 4 therefore yields the following characterization of level-1 cluster networks:

#### Corollary 40

Let *N* be a phylogenetic level-1 network. Then, *N* is a cluster network if and only if it is shortcut-free and separated.

Observation 19 then implies

#### Corollary 41

Let *N* be a binary level-1 network. Then, *N* is a cluster network if and only if it is shortcut-free.

We finally consider the problem as whether a clustering system $\mathscr {C}\subseteq 2^X$ compatible w.r.t. to a binary level-1 network.

#### Theorem 14

A given clustering system $\mathscr {C}\subseteq 2^X$ is compatible w.r.t. to a binary level-1 network if and only if $\mathscr {C}$ satisfies (L) and all hybrid vertices *w* in ${\mathfrak {H}}[\mathcal {I}(\mathscr {C})]$ have ${{\,\textrm{indeg}\,}}_{{\mathfrak {H}}[\mathcal {I}(\mathscr {C})]}(w)=2$.

#### Proof

Assume first that $\mathscr {C}$ is compatible w.r.t. to a binary level-1 network and let *N* be such a network with $\mathscr {C}\subseteq \mathscr {C}_N$. By Theorem 13, $\mathscr {C}$ satisfies (L). Moreover, by Theorem 8, $\mathscr {C}_N$ is closed and thus $\mathcal {I}(\mathscr {C})\subseteq \mathcal {I}(\mathscr {C}_N) = \mathscr {C}_N$. To see that all hybrid vertices *w* in ${\mathfrak {H}}[\mathcal {I}(\mathscr {C})]$ have ${{\,\textrm{indeg}\,}}_{{\mathfrak {H}}[\mathcal {I}(\mathscr {C})]}(w)=2$, suppose for contradiction that there is a vertex *w* in ${\mathfrak {H}}[\mathcal {I}(\mathscr {C})]$ with indegree larger than 2, i.e., there is $C\in \mathcal {I}(\mathscr {C})$ with at least three distinct inclusion-minimal supersets $C_1,C_2,C_3\in \mathcal {I}(\mathscr {C})\subseteq \mathscr {C}_N$. Since $C\subsetneq C_1,C_2,C_3$ and these cluster are inclusion-minimal (and thus not contained in one another), they overlap pairwise. By Observation 19, *N* is a galled tree. Hence, by Theorem 11, $\mathscr {C}_N$ contains no three pairwise overlapping clusters, a contradiction.

Assume now that $\mathscr {C}$ satisfies (L) and that all hybrid vertices *w* in ${\mathfrak {H}}{:}{=}{\mathfrak {H}}[\mathcal {I}(\mathscr {C})]$ have ${{\,\textrm{indeg}\,}}_{{\mathfrak {H}}}(w)=2$. In the following, we use caterpillars $\textrm{CAT}_n$, i.e., binary trees on *n* leaves such that each inner vertex has exactly two children and the subgraph induced by the inner vertices is a directed path with the root $\rho _{\textrm{CAT}_n}$ at one end of this path. By Theorem 10, ${\mathfrak {H}}$ is a level-1 network. This together with Observation 16 implies that every non-trivial block in ${\mathfrak {H}}$ is a cycle and thus ${\mathfrak {H}}$ must be a galled tree. In particular, for every non-trivial block *B*, $\max B$ has exactly two children in *B*. Let *v* be vertex in ${\mathfrak {H}}$ with ${{\,\textrm{outdeg}\,}}_{{\mathfrak {H}}}(v)>2$. We now “resolve” *v* as follows: If ${{\,\textrm{indeg}\,}}_{{\mathfrak {H}}}(v)=2$, then expand *v*. Otherwise, *v* is a tree vertex. In this case, let $\mathcal {B}$ be the set of all non-trivial blocks *B* in ${\mathfrak {H}}$ with $v= \max B$ and $\mathcal {C}$ be the children of *v* that are not contained in some $B\in \mathcal {B}$. We now replace *v* by a caterpillar $\textrm{CAT}_n$ with $n = \vert \mathcal {C}\vert +\vert \mathcal {B}\vert$ and thus, we can find a 1-to-1 correspondence between the *n* leaves of the caterpillar and the elements in $\mathcal {C}{\cup \!\!\!\cdot }\,\,\mathcal {B}$. The elements in $\mathcal {C}$ are now identified with their corresponding leaves. Observe that $\vert \mathcal {B}\vert >1$ is possible, i.e., $v=\max B$ for more than one non-trivial block *B*. We therefore re-attach, for each block $B\in \mathcal {B}$, the two children of $v=\max B$ that are contained in *B* as children of the leaf of the caterpillar that corresponds to *B*. Note that this construction is well defined since, by Lemma 9, no two such children can be children of two distinct blocks $B,B'\in \mathcal {B}$. Since we do not change the structure of non-trivial blocks, the network $N'$ obtained in this way remains a level-1 network whose hybrid vertices still have indegree 2. Moreover, it is an easy task to verify that $\mathscr {C}\subseteq \mathscr {C}_{{\mathfrak {H}}}\subseteq \mathscr {C}_{N'}$. Repeated application of the latter steps to all vertices eventually results in a binary level-1 network *N* with $\mathscr {C}\subseteq \mathscr {C}_{{\mathfrak {H}}} \subseteq \mathscr {C}_N$. $\square$

Using Check-L1-Compatibility and the results in Theorem 9, we obtain

#### Corollary 42

Determining if a clustering system $\mathscr {C}\subseteq 2^X$ is compatible w.r.t. to a binary level-1 network and, in the affirmative case, the construction of such a network can be done in $O(\vert X\vert ^5)$ time.

#### Corollary 43

For all phylogenetic level-1 networks *N* whose hybrid vertices *w* have ${{\,\textrm{indeg}\,}}_{N}(w)=2$, there is a binary level-1 network $N'$ with $\mathscr {C}_N\subseteq \mathscr {C}_{N'}$.

#### Proof

If all hybrid vertices *w* in *N* have ${{\,\textrm{indeg}\,}}_{N}(w)=2$, then *N* is a galled tree by Observation 16. By Theorem 11, $\mathscr {C}_N$ is closed (i.e., $\mathscr {C}_N=\mathcal {I}(\mathscr {C})$) and satisfies (L) and, moreover, ${\mathfrak {H}}[\mathscr {C}]= {\mathfrak {H}}[\mathcal {I}(\mathscr {C})]$ is a galled tree. Applying Observation 16 again to ${\mathfrak {H}}[\mathcal {I}(\mathscr {C})]$ yields that all hybrid vertices *w* in ${\mathfrak {H}}[\mathcal {I}(\mathscr {C})]$ have ${{\,\textrm{indeg}\,}}_{{\mathfrak {H}}[\mathcal {I}(\mathscr {C})]}(w)=2$. Now, apply Theorem 14. $\square$

The converse of Corollary 43 is not true, i.e., a phylogenetic level-1 networks *N* for which there is a binary level-1 network $N'$ with $\mathscr {C}_N\subseteq \mathscr {C}_{N'}$ may contain a hybrid vertex *w* with ${{\,\textrm{indeg}\,}}_{N}(w)>2$. To see this, consider a binary level-1 network $N'$ with a non-trivial block *B* such that $(\max B,\min B)\notin E(N')$ and the non-binary network *N* that is obtained from $N'$ by adding the shortcut $(\max B, \min B)$. Clearly, *N* is still phylogenetic and level-1 and by Lemma 1 satisfies ${{\,\mathrm{\texttt{C}}\,}}_{N}={{\,\mathrm{\texttt{C}}\,}}_{N'}$ but the vertex $\min B$ has indegree 3.

### Level-1 networks encoded by their cluster multisets

In this part, we focus on a particular subclass of networks:

#### Definition 32

A network *N* is a *quasi-binary* if ${{\,\textrm{indeg}\,}}_N(w)=2$ and ${{\,\textrm{outdeg}\,}}_{N}(w)=1$ for every hybrid vertex $w\in V(N)$ and, additionally, ${{\,\textrm{outdeg}\,}}_N(\max B) = 2$ for every non-trivial block *B* in *N*.

We note that, in particular, all binary networks are quasi-binary. Moreover, quasi-binary networks are separated. Therefore and by Corollary 39, we obtain

#### Observation 20

Quasi-binary level-1 networks are separated galled trees.

Theorem 5 shows that all semi-regular networks are encoded by their multisets of clusters. None of the conditions (PCC) or shortcut-free can be omitted, as shown be the examples in Fig. 9. Nevertheless, replacing some of these conditions by a different one might be possible. In the following, we replace shortcut-freeness by requiring that *N* is a phylogenetic quasi-binary level-1 network. In this case, we obtain Property (PCC) as an immediate consequence of Lemma 44. Again, we observe that neither of the properties phylogenetic or quasi-binary or level-1 can be dropped: Fig. 17A shows two phylogenetic quasi-binary networks that are not level-1; Fig. 17B shows two quasi-binary level-1 networks that are not phylogenetic; and Fig. 17C shows two phylogenetic level-1 networks that are not quasi-binary where, in all three examples, the respective networks have the same multisets of clusters but are not isomorphic. As a by-product, we obtain the following result:

#### Observation 21

Let $\mathbb {P}$ denote the class of *all* networks *N* for which either precisely one or at least one of the following conditions hold:

1. *N* is level-*k*, $k\ge 2$ but not level-1 and contains at least three leaves.
2. *N* does not satisfy (PCC);
3. *N* is not quasi-binary;
4. *N* is not shortcut-free;
5. *N* is not phylogenetic.

Then, no network $N\in \mathbb {P}$ is encoded (w.r.t. $\mathbb {P}$) by its multiset $\mathscr {M}_N$ of clusters and thus, by its set $\mathscr {C}_N$ of clusters.

#### Theorem 15

Let *N* be a phylogenetic quasi-binary level-1 network. Then, *N* is the unique phylogenetic quasi-binary level-1 network tree whose cluster multiset is $\mathscr {M}_N$.

#### Proof

Suppose that both *N* and $\tilde{N}$ are phylogenetic quasi-binary level-1 networks and $\mathscr {M}_N= \mathscr {M}_{\tilde{N}}$. Lemma 44 implies that both *N* and $\tilde{N}$ satisfy (PCC). We show that $\varphi {:}{=}\varphi _{PCC}:V(N) \rightarrow V(\tilde{N})$ is a graph isomorphism. By Lemma 31, $\varphi$ is a bijection between *V*(*N*) and $V(\tilde{N})$ that is the identity on the common leaf set *X*. In the following, we write $\tilde{v}{:}{=}\varphi (v)$ for all $v\in V(N)$, and make free use of the facts that, by Lemma 31, ${{\,\mathrm{\texttt{C}}\,}}_{N}(v)={{\,\mathrm{\texttt{C}}\,}}_{\tilde{N}}(\tilde{v})$ and *v* is a leaf if and only if $\tilde{v}$ is a leaf, and moreover, $u\prec _{N} v$ if and only if $\tilde{u}\prec _{\tilde{N}} \tilde{v}$ for all $u,v\in V(N)$. In the following, we will make frequent use of the fact that both *N* and $\tilde{N}$ are galled trees (cf. Observation 20).

It remains to show that, for all $u,v\in V(N)$, it holds $(v,u)\in E(N)$ if and only if $(\tilde{v},\tilde{u})\in E(\tilde{N})$. To this end, suppose $(v,u)\in E(N)$ and, for contradiction, that $(\tilde{v},\tilde{u})\notin E(\tilde{N})$. Since *N* is acyclic and finite, we can assume w.l.o.g. that $(v,u)\in E(N)$ is a $\preceq _{N}$-minimal arc that is “missing” in $\tilde{N}$, i.e., there is no arc $(v',u')\in E(N)$ with $u'\prec _{N} u$ and $(\tilde{v}',\tilde{u}')\notin E(\tilde{N})$. We have $u\prec _{N} v$ and thus also $\tilde{u}\prec _{\tilde{N}} \tilde{v}$. The latter together with $(\tilde{v},\tilde{u})\notin E(\tilde{N})$ implies that there is $\tilde{w}\in V(\tilde{N})$ such that $\tilde{u}\prec _{\tilde{N}} \tilde{w} \prec _{\tilde{N}} \tilde{v}$. This in turn implies $u\prec _{N} w \prec _{N} v$, and thus, (*v*, *u*) is a shortcut in *N*. In particular, *u* is a hybrid vertex and, since by definition the non-trivial blocks in the galled tree *N* correspond to the undirected cycles, $v=\max B$ for some non-trivial block *B* of *N* whose unique hybrid vertex is $u=\min B$.

We continue with showing that $\tilde{u}$ is also a hybrid vertex. Since *N* is quasi-binary, the hybrid vertex *u* has a unique child *c*. By the $\preceq _{N}$-minimal choice of $(v,u)\in E(N)$, $\tilde{c}$ must be a child of $\tilde{u}$ in $\tilde{N}$. Suppose, for contradiction, that $\tilde{u}$ is a tree vertex. Hence, since $\tilde{N}$ is phylogenetic, it must have a second child $\tilde{c}'\in {{\,\textrm{child}\,}}_{\tilde{N}}(\tilde{u}) {\setminus } \{\tilde{c}\}$. Now, $\tilde{c}'\prec _{\tilde{N}} \tilde{u}$ implies that $c'\prec _{N} u$. Therefore and by the choice of (*v*, *u*), there is an arc $(p,c')\in V(N)$ (where $p\ne u$ since *c* is the only child of *u*) such that $(\tilde{p},\tilde{c}')$ is also an arc in $\tilde{N}$. Thus, $\tilde{c}'$ is a hybrid vertex with distinct parents $\tilde{u}$ and $\tilde{p}$. Hence, neither of $c'$ and $\tilde{c}'$ is a leaf. Therefore, and because *N* is phylogenetic, $c'$ either has a second parent $p'$ (which is also distinct from *u*), or at least two children. By the choice of (*v*, *u*), the images of these vertices are adjacent with $\tilde{c}'$ in both cases. Hence, if $c'$ has a second parent $p'$, then $\tilde{c}'$ has three distinct parents $\tilde{u}$, $\tilde{p}$, and $\tilde{p}'$. If on the other hand $c'$ has at least two children, then the hybrid vertex $\tilde{c}'$ also has at least two children. Both cases, therefore, contradict that $\tilde{N}$ is quasi-binary. Hence, $\tilde{u}$ must be a hybrid vertex.

Let *z* be the parent of the hybrid vertex *u* that is not *v* and observe that $z\preceq _{N} w\prec _{N}\max B=v$. Moreover, $u\prec _{N} z$ implies $\tilde{u}\prec _{\tilde{N}} \tilde{z}$. Suppose, for contradiction, that $(\tilde{z}, \tilde{u})\notin E(\tilde{N})$. In this case, $\tilde{u}\prec _{\tilde{N}} \tilde{z}$ implies that there is $\tilde{z}'$ with $\tilde{u}\prec _{\tilde{N}} \tilde{z}'\prec _{\tilde{N}} \tilde{z}$ and thus $u\prec _{N} z'\prec _{N} z$. Hence, *u* must have a parent $z''$ with $z''\preceq _{N} z'\prec _{N} z(\prec _{N} v)$. Since *N* is acyclic, it holds that $z'\ne v$ and thus, $z''\ne z,v$. This contradicts the fact that *z* and *v* are the only two parents of *u* in *N*. Therefore, $\tilde{z}$ must be one of the two parents of $\tilde{u}$ in $\tilde{N}$. In particular, it holds $\tilde{u}\prec _{\tilde{N}}\tilde{z}\prec _{\tilde{N}}\tilde{v}$.

Now, let $\tilde{q}$ be the parent of the hybrid vertex $\tilde{u}$ that is not $\tilde{z}$. Since we assumed that $(\tilde{v},\tilde{u})\notin E(\tilde{N})$, it holds that $\tilde{q}\ne \tilde{v}$ (and thus $q\ne z$). We have $\tilde{u}\prec _{\tilde{N}} \tilde{q}$ and thus $u\prec _{N} q$. Therefore, and since $q\notin \{v,z\}={{\,\textrm{par}\,}}_{N}(u)$, we must have $z\prec _{N} q$ or $v \prec _{N} q$. Since $z\prec _{N}\max B = v$, we have, in both of the latter two cases, that $z\prec _{N} q$. Moreover, since the non-trivial block *B* consists of two paths that have only *v* and *u* in common and which do not contain additional hybrid vertices and since *v* and *z* are the unique two parents of *u*, it holds that $z\prec _{N} q$ and (a) $q\prec _{N} v$ or (b) $v\prec _{N} q$. In particular, we also have $(\tilde{u}\prec _{\tilde{N}}) \; \tilde{z}\prec _{\tilde{N}} \tilde{q}$, which implies that $(\tilde{q},\tilde{u})$ is a shortcut.

**Fig. 18:**
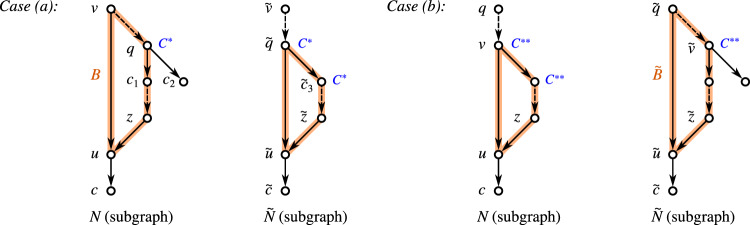
Illustration of Cases (**a**) and (**b**) in the proof of Theorem 15. Dashed arrows indicate directed paths (possibly consisting of a single vertex)

*Case (a)* $q\prec _{N} v$. The situation that we will obtain in the following up to the final contradiction is illustrated in Fig. 18. Together with $z\prec _{N} q$ and Lemma 7, $q\prec _{N} v$ implies that *q* is also contained in *B*. In particular, $\min B=u \prec _{N} q$ and thus *q* is a tree vertex. Since *N* is a galled tree, *B* is exactly the undirected cycle that is formed by the shortcut (*v*, *u*) and the directed path from $v=\max B$ to $u=\min B$ (which contains *q* as an inner vertex). Hence, *q* has exactly one child $c_1$ in *B* and, since *N* is phylogenetic, at least one child that is not in *B*. Consider an arbitrary such child $c_2$ that is not in *B*. Since all vertices of *B* lie on a directed path and $z\prec _{N} q$, we must have $z\preceq _{N} c_1 \prec _{N} \max B$. In particular, $c_1$ is also a tree vertex since *N* is level-1. The vertices $c_1$ and $c_2$ must be $\preceq _{N}$-incomparable. To see this, suppose $c_1\prec _{N} c_2$. Then, $(q,c_1)$ is a shortcut, and thus, because *N* is a level-1 network whose non-trivial blocks correspond to undirected cycles, *N* contains a non-trivial block $B'$ that is formed be the shortcut $(q,c_1)$ and a directed path from *q* to $c_1$ that passes through $c_2$. In particular, $q=\max B'$ and both of $c_1$ and $c_2$ are contained in $B'$. Therefore, *B* and $B'$ share the two vertices *q* and $c_1$ and we have $B=B'$ by Observation 1, a contradiction to $c_2\notin V(B)$. Similarly, $c_2\prec _{N} c_1$ is not possible and thus $c_1$ and $c_2$ are $\preceq _{N}$-incomparable. Suppose, for contradiction, that there is a vertex $x\in {{\,\mathrm{\texttt{C}}\,}}_{N}(c_1)\cap {{\,\mathrm{\texttt{C}}\,}}_{N}(c_2)$. Then, Lemma 18 implies that $c_1$ and $c_2$ are contained in some non-trivial block $B'$ of *N*. Since they are $\preceq _{N}$-incomparable, it holds $c_1\ne \max B'$, and thus, the unique parent *q* of the tree vertex $c_1$ must also be contained in $B'$. Similar as before, we therefore obtain $B=B'$, a contradiction to $c_2\notin V(B)$. Hence, ${{\,\mathrm{\texttt{C}}\,}}_{N}(c_1)$ and ${{\,\mathrm{\texttt{C}}\,}}_{N}(c_2)$ are disjoint. In particular, we have ${{\,\mathrm{\texttt{C}}\,}}_{N}(c_1) {\cup \!\!\!\cdot }\,\,{{\,\mathrm{\texttt{C}}\,}}_{N}(c_2)\subseteq {{\,\mathrm{\texttt{C}}\,}}_{N}(q)$, and, because both of ${{\,\mathrm{\texttt{C}}\,}}_{N}(c_1)$ and ${{\,\mathrm{\texttt{C}}\,}}_{N}(c_2)$ are non-empty, we obtain ${{\,\mathrm{\texttt{C}}\,}}_{N}(c_i)\subsetneq {{\,\mathrm{\texttt{C}}\,}}_{N}(q)$, $i=1,2$. Since $c_2$ was chosen arbitrarily, we have ${{\,\mathrm{\texttt{C}}\,}}_{N}(c_i)\subsetneq {{\,\mathrm{\texttt{C}}\,}}_{N}(q)$ for all children $c_i\in {{\,\textrm{child}\,}}_{N}(q)$. Together with Lemma 17 and the fact that every vertex $w'$ with $w'\prec _{N} q$ satisfies $w'\preceq _{N} c_i$ for some $c_i\in {{\,\textrm{child}\,}}_{N}(q)$, these inclusions imply that ${{\,\mathrm{\texttt{C}}\,}}_{N}(w')\subsetneq {{\,\mathrm{\texttt{C}}\,}}_{N}(q)$. Hence, *q* is a $\preceq _{N}$-minimal vertex with cluster $C^*{:}{=}{{\,\mathrm{\texttt{C}}\,}}_{N}(q) ( = {{\,\mathrm{\texttt{C}}\,}}_{\tilde{N}}(\tilde{q}))$. Since $(\tilde{q},\tilde{u})$ is a shortcut, $\tilde{q} = \max \tilde{B}$ of some non-trivial block $\tilde{B}$ in $\tilde{N}$. Since $\tilde{N}$ is quasi-binary, $\tilde{q}$ has precisely two children $\tilde{u}$ and $\tilde{c}_3$. Since $(\tilde{q},\tilde{u})$ is a shortcut and the non-trivial blocks of *N* correspond to undirected cycles, we have $\tilde{u} \prec _{\tilde{N}} \tilde{c}_3$ and thus ${{\,\mathrm{\texttt{C}}\,}}_{\tilde{N}}(\tilde{u}) \subseteq {{\,\mathrm{\texttt{C}}\,}}_{\tilde{N}}(\tilde{c}_3)$ by Lemma 17. Therefore, we obtain ${{\,\mathrm{\texttt{C}}\,}}_{\tilde{N}}(\tilde{q}) = {{\,\mathrm{\texttt{C}}\,}}_{\tilde{N}}(\tilde{u}) \cup {{\,\mathrm{\texttt{C}}\,}}_{\tilde{N}}(\tilde{c}_3)= {{\,\mathrm{\texttt{C}}\,}}_{\tilde{N}}(\tilde{c}_3)$. Together with $\tilde{c}_3 \prec _{N} \tilde{q}$, this implies that $\tilde{q}$ is not a $\preceq _{\tilde{N}}$-minimal vertex with cluster $C^*$ in $\tilde{N}$ (as opposed to *q* in *N*), a contradiction to the construction of $\varphi$. In summary, therefore, Case (a) cannot occur.

*Case (b)* $v\prec _{N} q$. This implies $\tilde{v}\prec _{\tilde{N}} \tilde{q}$. Since $(\tilde{q},\tilde{u})$ is a shortcut, it holds $\tilde{q}= \max \tilde{B}$ and $\tilde{u}= \min \tilde{B}$ for some non-trivial block $\tilde{B}$ in $\tilde{N}$. Lemma 7, together with $\tilde{v}\prec _{\tilde{N}} \tilde{q}$ and $\tilde{u}\prec _{\tilde{N}} \tilde{v}$, implies that $\tilde{v}$ is also contained in $\tilde{B}$. We can now apply similar arguments as in Case (a), where the roles of *N* and $\tilde{N}$ are interchanged, to conclude that Case (b) is also impossible. The situation up to the final contradiction is again illustrated in Fig. 18.

In summary, therefore, $(\tilde{v},\tilde{u})\in E(\tilde{N})$ must hold. By analogous arguments, $(\tilde{v},\tilde{u})\in E(\tilde{N})$ implies $(v,u)\in E(N)$. Hence, $\varphi$ is a graph isomorphism that is the identity on *X* and thus $N\simeq \tilde{N}$. Therefore, *N* is the unique phylogenetic quasi-binary level-1 network whose cluster multiset is $\mathscr {M}_N$. $\square$

Our colleagues Simone Linz and Kristina Wicke drew our attention to an alternative proof for Theorem 15 that proceeds by induction on the size of the leaf set of *N*. It utilizes the concepts of cherries and reticulated cherries that have been used extensively in the literature, see, e.g., Bordewich and Semple (2016); Murakami et al. (2019); Semple and Toft (2021). We opted for a non-inductive proof that remains closer to the construction utilized throughout this contribution.

#### Corollary 44

Let *N* be a binary level-1 network. Then, *N* is the unique binary level-1 network whose cluster multiset is $\mathscr {M}_N$.

Again, we can observe that neither of the properties binary or level-1 in Corollary 44 can be dropped: Fig. 9A shows two level-1 networks (where one is not binary), and Fig. 17A shows two binary level-2 networks (that are not level-1) where, in both examples, the respective networks have the same multisets of clusters but are not isomorphic.

**Table 3:**
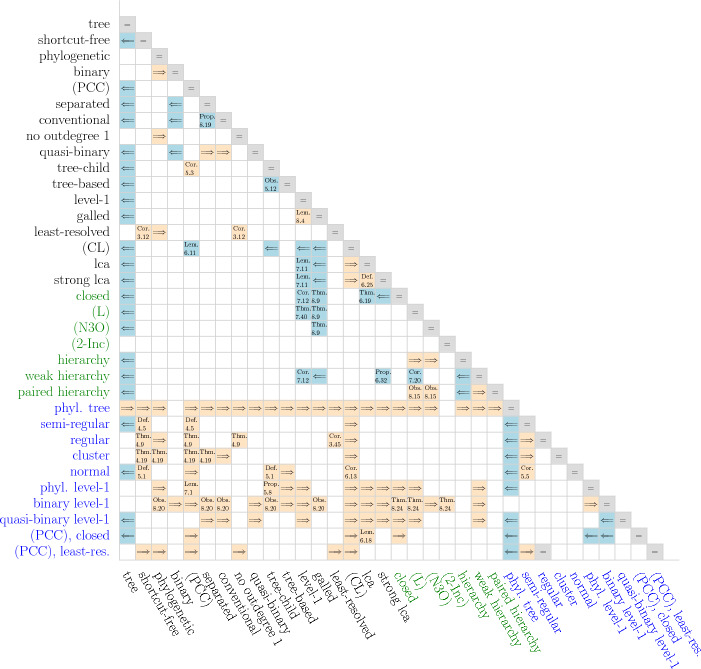
Summary of main results: Mutual dependencies between the types of networks and clustering systems considered in this paper (color figure online)

The properties highlighted in black and green refer to properties of networks and clustering systems, respectively. Properties in blue text are combinations of “basic” properties of networks. An entry at position (*i*, *j*) in the matrix is colored orange, turquoise, and white, if the property at pos. *i* implies *j*, is implied by *j*, or does have a non-empty overlap with *j*, respectively. Gray colored entries refer to equality. References within the matrix indicate the result where the respective dependencies are shown. All other colors in the matrix are either trivial observations or were derived by computing the transitive closure over the proven implications

**Fig. 19:**
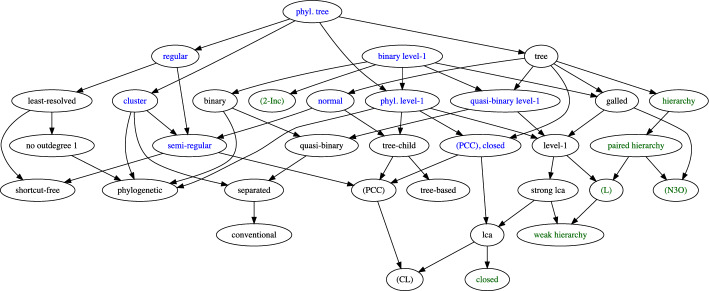
Summary of main results: Transitive reduction of the implication graph defined in Table 3 (see there for explanation of the colors) (color figure online)

## Summary

In this contribution, we investigated the mutual dependencies between the different concept of networks in the literature and their connection to clustering systems. Most of our findings are summarized in Table 3 and Fig. 19. As one of the main results, level-1 networks as well as some of their subclasses, such as galled trees or binary phylogenetic level-1 networks, are characterized by the structure of their clustering system $\mathscr {C}$. Moreover, we showed that semi-regular networks and phylogenetic quasi-binary level-1 (and thus, binary level-1 networks) are uniquely determined by their multisets of clusters. Furthermore, regular and cluster networks (and their subclasses as, e.g., phylogenetic trees) are uniquely determined by their clustering system. We provided a plethora of examples that show, however, that most classes of networks cannot be encoded in such way if there are not sufficiently many extra restrictions placed on such networks. In addition, we showed that it is possible to determine in polynomial time whether a clustering system is compatible with a level-1 network and to construct such a network in the affirmative case.

It remains an open question as whether general level-*k* networks can be characterized by their clustering systems. Moreover, under which conditions is a clustering system compatible with other specified networks and what is the computational complexity to determine them? While we have shown that some types of networks can be encoded by the multisets of clusters, a characterization of multisets that encode the underlying networks as well as reconstruction algorithms are part of future research.

From the point of view of clustering systems, phylogenetic networks suggest properties that may also be of relevance in practical data analysis beyond applications in phylogenetics. Since clustering systems that satisfy property (L) are between hierarchies and weak hierarchies (Bertrand and Diatta 2014), they appear as an attractive alternative, e.g., to pyramidal clustering (Bertrand and Diatta 2013) for data that are not naturally linearly ordered.

## Appendix 1: Additional results and proofs

### Appendix 1.1: Expansion, contraction, and blocks

We first provide the proof of

#### Lemma

**3** Let *N* be a network and $(u,w) \in E(N)$ be an arc that is not a shortcut. Then, ${{\,\mathrm{\textsc {cntr}}\,}}(u,w)$ applied on *N* results in a network $N'$ with leaf set *X* or $X\setminus \{w\}$ and $V(N')=V(N)\setminus \{u\}$. Moreover, for all $v,v'\in V(N')$,

1. $$v\preceq _N v'$$ implies $$v \preceq _{N'} v'$$, and
2. $v\preceq _{N'} v'$ implies (i) $v \preceq _{N} v'$ or (ii) $w\preceq _N v'$ and $v\preceq _N w'$ for some $w'\in {{\,\textrm{child}\,}}_N(u)\setminus \{w\}$ that is $\preceq _{N}$-incomparable with *w*.

In particular, $v\prec _{N'} v'$ always implies $v\prec _{N} v'$ or *v* and $v'$ are $\preceq _{N}$-incomparable.

#### Proof

Assume that (*u*, *w*) is not a shortcut in *N* and recall that ${{\,\mathrm{\textsc {cntr}}\,}}(u,w)$ consists in replacing arcs (*v*, *u*) by (*v*, *w*) for all $v\in {{\,\textrm{par}\,}}_N (u)$, replacing arcs (*u*, *v*) by (*w*, *v*) for all $v\in {{\,\textrm{child}\,}}_N (u)\setminus \{w\}$, and deleting (*u*, *w*) and *u*. Observe that only the arcs incident with *u* are removed and all inserted arcs are incident with *w*. While *u* does not exist in $N'$ anymore, both the in- and out-neighborhood of *w* may change in such a way that *w* may get additional in/out-neighbors of *u*.

We show first that $N'$ has a single vertex with indegree 0. Suppose first $u\ne \rho _N$, and thus, $\rho _N\in V(N')$. Since clearly $\rho _{N}\notin {{\,\textrm{child}\,}}_N(u)$, we did not insert the arc $(w, \rho _N)$. Together with the fact that all inserted arcs are incident with *w*, this implies that $\rho _N$ still has indegree 0 in $N'$. Since $u\ne \rho _N$, vertex *u* has at least one in-neighbor, which becomes an in-neighbor of *w* if it was not already an in-neighbor of *w* in *N*. Now, suppose, for contradiction, that there is a vertex $v\in V(N'){\setminus }\{\rho _N, w\}$ with ${{\,\textrm{indeg}\,}}_{N'}(v)=0$. Since $v\ne \rho _N$, there must be an arc $(u',v)$ in *N* which is no longer contained in $N'$. By construction and since $v\ne u$, we must have $u'=u$. But then $(u,v)=(u',v)\in E(N)$ implies that (*w*, *v*) is an arc in $N'$, contradicting that ${{\,\textrm{indeg}\,}}_{N'}(v)=0$. Now, consider the case $u= \rho _N$. Then, *u* is the unique in-neighbor of *w* in *N*. To see this, assume, for contradiction, that there is $v\in {{\,\textrm{par}\,}}_N(w){\setminus }\{u\}$ and thus $w\prec _N v$. Since $u=\rho _N$ is the unique root, there is a *uv*-path. Since $v\ne u$, this path passes through some child $w'$ of *u*. Since $w\prec _N v \preceq _{N} w'$, we must have $w\ne w'$ and thus $w\prec _N w'\prec _N u$, i.e., (*u*, *w*) is a shortcut, a contradiction. Therefore, *u* is the unique in-neighbor of *w*. Moreover, since ${{\,\textrm{par}\,}}_N(u)=\emptyset$, *w* does not get any new in-neighbors. After deletion of *u*, *w* has outdegree 0 in $N'$. Re-using the arguments from the case $u\ne \rho _N$, there is no vertex $v\in V(N'){\setminus }\{\rho _N, w\}$ with ${{\,\textrm{indeg}\,}}_{N'}(v)=0$. Hence, $N'$ is a directed graph that has a unique vertex with indegree 0, i.e., a unique root $\rho _{N'}$, in both cases.

We continue by showing that $N'$ is a DAG. Assume, for contradiction, that $N'$ contains a directed cycle *K* comprising the vertices $v_1, v_2, \dots , v_k$, $k\ge 2$, in this order, i.e., $(v_i,v_{i+1})$, $1\le i\le k-1$ and $(v_k,v_1)$ are arcs in $N'$. If all arcs along *K* are arcs of *N*, then *K* is a directed cycle in *N*. a contradiction. Hence, at least one arc *e* in *K* cannot be contained in *N*. By construction, *e* must be of the form (*v*, *w*) with $v\in {{\,\textrm{par}\,}}_N(u){\setminus }{{\,\textrm{par}\,}}_N(w)$ or of the form (*w*, *v*) with $v\in {{\,\textrm{child}\,}}_N(u){\setminus }{{\,\textrm{child}\,}}_N(w)$. Since *w* can appear in at most two arcs of this form, all other arcs in *K* must also be arcs in *N*. Assume w.l.o.g. that $v_1=w$. Then, the following cases have to be considered:

- $(w,v_2)=(v_1, v_2)\notin E(N)$ but all other arcs are contained in *N*,
- $(v_k,w)=(v_k, v_1)\notin E(N)$ but all other arcs are contained in *N*, and
- exactly the arcs $(w,v_2)=(v_1, v_2)$ and $(v_k,w)=(v_k, v_1)$ are not contained in *N*.

In case (a), we must have $(u,v_2)\in V(N)$ by construction, i.e., $v_2\in {{\,\textrm{child}\,}}_N(u)$. Since all arcs except $(v_1,v_2)$ exist in *N* and $w=v_1\ne v_2$, there is a $v_2 w$-path in *N*, i.e., $w\prec _N v_2$. This together with $w, v_2\in {{\,\textrm{child}\,}}_N(u)$ implies that (*u*, *w*) is a shortcut, a contradiction. In case (b), we must have $(v_k,u)\in V(N)$ by construction. Then, *N* contains a directed cycle along $v_0{:}{=}u, v_1, v_2, \dots , v_k$ in this order, i.e., $(v_i,v_{i+1})$, $0\le i\le k-1$ and $(v_k,v_0)=(v,u)$ are arcs in *N*, a contradiction. In case (c), we must have $(v_k,u),(u,v_2)\in V(N)$ by construction. Replacing $v_1$ and its incident arcs in the cycle *K* by vertex *u* (which is not already in $V(K)\subseteq V(N')$) and arcs $(v_k,u)$ and $(u,v_2)$ thus yields a directed cycle $K'$ in *N*, a contradiction. In summary, neither of the three cases is possible and thus $N'$ is acyclic. Since $N'$ has a unique root and acyclicity is preserved as well, $N'$ is a rooted network.

Since ${{\,\textrm{outdeg}\,}}_{N}(u)>1$, we do not delete any leaf of *N*, i.e., $X\subseteq V(N')$. Moreover, the only vertices whose out-neighborhood changes are *w* and the vertices in ${{\,\textrm{par}\,}}_N(u)$. Since all vertices in ${{\,\textrm{par}\,}}_N(u)$ have *w* as out-neighbor in $N'$, they do not become leaves. Hence, the leaf set of $N'$ is either *X* or $X\setminus \{w\}$.

We continue with showing that $v\preceq _N v'$ implies $v \preceq _{N'} v'$ for all $v,v'\in V(N')$. Thus, assume that $v\preceq _N v',$ i.e., there is a $v'v$-path $P{:}{=}(v'=v_1, \dots , v=v_k)$ in *N*. If *P* does not contain *u*, then *P* is also a $v'v$-path in $N'$ since only arcs that are incident with *u* are removed. Now, suppose *P* contains *u*. Then, clearly $u=v_i$ for some $1<i<k$. Since *u* appears in *P* at most once and only arcs incident with *u* were removed, all arcs in *P* except $(v_{i-1},u)$ and $(u,v_{i+1})$ are also arcs in $N'$. Observe that $(v_{i-1},w)\in E(N')$ holds by construction. If $v_{i+1}=w$, then $(v_{i-1}, v_{i+1})$ is an arc in $N'$ and thus $\tilde{P}=(v_1,\dots ,v_{i-1}, v_{i+1}, \dots , v_k)$ is a $v'v$-path in $N'$. Otherwise, we have $v_{i+1}\in {{\,\textrm{child}\,}}_N(u)\setminus \{w\}$ and thus $(w,v_{i+1})$ is an arc in $N'$. The vertex *w* is not contained in *P*. To see this, observe first that $w\ne v_{i+1}$ and $w\in {{\,\textrm{child}\,}}_N(u=v_i)$ imply $w\prec _N v_j$ for $1\le j\le i$. If $w=v_j$ for some $i+1<j\le k$, then $w\prec _N v_{i+1}$ which implies that (*u*, *w*) is a shortcut, a contradiction. Taken together, these arguments imply that $\tilde{P}=(v_1,\dots ,v_{i-1}, w, v_{i+1}, \dots , v_k)$ is a $v'v$-path in $N'$. Hence, we have $v\preceq _{N'} v'$ is all cases.

Conversely, suppose $v \preceq _{N'} v'$, i.e., there is a $v'v$-path $P'{:}{=}(v'=v_1, \dots , v=v_k)$ in $N'$. If all arcs in $P'$ are arcs in *N*, then $P'$ is a $v'v$-path in *N* and thus $v \preceq _{N} v'$. Now, suppose that $P'$ contains at least one arc that is not in *N*. By construction, any such arc must be incident with *w* and thus $P'$ contains at most two arcs that are not in *N*. These are then consecutive in $P'$. We therefore have to consider three cases:

(a’): $(w,v_{i+1})=(v_{i}, v_{i+1})\notin E(N)$ for some $1\le i<k$ but all other arcs in $P'$ are contained in *N*,
(b’): $(v_{i-1},w)=(v_{i-1}, v_{i})\notin E(N)$ for some $1< i\le k$ but all other arcs in $P'$ are contained in *N*, and
(c’): exactly the arcs $(v_{i-1},w)=(v_{i-1}, v_{i})$ and $(w,v_{i+1})=(v_{i}, v_{i+1})$ with $1< i< k$ in $P'$ are not contained in *N*.

In case (a’), we have by construction that $(u,v_{i+1})\in E(N)$, i.e., $v_{i+1}\in {{\,\textrm{child}\,}}_N(u)$. Since all other arcs of $P'$ are arcs in *N*, the subpath of $P'$ from $v'$ to *w* is a $v'w$-path in *N* and thus $w\preceq _{N} v'$. Similarly, the subpath from $v_{i+1} (\ne w)$ to *v* is a $v_{i+1} v$-path in *N* implying that $v\preceq _N v_{i+1}$ with $v_{i+1}\in {{\,\textrm{child}\,}}_N(u){\setminus } \{w\}$. Clearly $w\prec _N v_{i+1}$ is not possible since otherwise (*u*, *w*) would be a shortcut in *N*. Hence, we have either $v\preceq _N v_{i+1} \prec _N w \preceq _N v'$ or *w* and $v_{i+1}$ are $\preceq _{N}$-incomparable children of *u*.

In case (b’), we have by construction that $(v_{i-1},u)\in E(N)$, i.e., $v_{i-1}\in {{\,\textrm{par}\,}}_N(u)$. Since all arcs of $P'$ except $(v_{i-1},v_{i})$ are also in *N*, we have $v\preceq _{N} v_{i}=w\prec _N u \prec _N v_{i-1} \preceq _N v'$.

In case (c’), we have by construction that $(v_{i-1},u), (u, v_{i+1})\in E(N)$. Since all arcs of $P'$ except $(v_{i-1}, v_{i})$ and $(v_{i}, v_{i+1})$ are also arcs in *N*, we obtain $v\preceq _{N} v_{i+1}\prec _N u \prec _N v_{i-1} \preceq _N v'$. In summary, in all cases it holds that at least one of (i) $v\preceq _N v'$ or (ii) $w\preceq _N v'$ and $v\preceq _N w'$ for some $w'\in {{\,\textrm{child}\,}}_N(u)\setminus \{w\}$ that is $\preceq _{N}$-incomparable with *w* is true.

For the final statement, assume that $v\prec _{N'} v'$. If $v'\preceq _{N} v$ would, then (1) implies $v'\preceq _{N'} v$, a contradiction. Consequently, we have $v\prec _{N} v'$ or *v* and $v'$ are $\preceq _{N}$-incomparable. $\square$

In the following, we show that the operation ${{\,\mathrm{\textsc {expd}}\,}}(w)$ does not introduce shortcuts and that neither ${{\,\mathrm{\textsc {expd}}\,}}(w)$ nor ${{\,\mathrm{\textsc {cntr}}\,}}(w',w)$ increases the level of a network. Besides providing additional results, we give here the proofs for statements that were omitted in the main text.

#### Lemma 72

Let $N_1$ be a network, $N_2$ be the network obtained from $N_1$ by applying ${{\,\mathrm{\textsc {expd}}\,}}(w_1)$ for some $w_1\in V(N_1)$, and $w_2$ be the unique vertex in $V(N_2)\setminus V(N_1)$. Then, for every shortcut $(u,v) \in E(N_i)$, it holds either (i) $v\ne w_i$ and (*u*, *v*) is a shortcut in $N_j$ or (ii) $v= w_i$ and $(u,w_j)$ is a shortcut in $N_j$ such that $i,j\in \{1,2\}$ are distinct.

#### Proof

In the following, we put $N=N_1$, $N'=N_2$, $w=w_1$ and $w'=w_2$. Recall that by Lemma 5, *N* and $N'$ are $(N',N)$-ancestor-preserving.

Suppose first that $(u,v) \in E(N')$ is a shortcut in $N'$, i.e., there is a $v'\in {{\,\textrm{child}\,}}_{N'}(u)\setminus \{v\}$ such that $v\prec _{N'} v'$. The fact that $N'$ is acyclic, $v\prec _{N'} v'\prec _{N'} u$ and $(u,v) \in E(N')$ together imply that ${{\,\textrm{indeg}\,}}_{N'}(v)\ge 2$. By construction, it holds ${{\,\textrm{par}\,}}_{N'}(w)=\{w'\}$ and ${{\,\textrm{child}\,}}_{N'}(w')=\{w\}$ which yield $v\ne w$ and $u\ne w'$, respectively.

(i) Suppose first that $v\ne w'$. Then, (*u*, *v*) is also an arc in *N* since moreover $u\ne w'$ and all newly inserted arcs are incident with $w'$. Similarly, if in addition $v'\ne w'$, then $(u,v')\in E(N)$ and moreover $v\prec _{N'} v'$ implies $v\prec _{N} v'$. Hence, (*u*, *v*) is a shortcut in *N*. If on the other hand $v'=w'$, then $w\in {{\,\textrm{child}\,}}_N(u)$ and $v\prec _{N'} v'=w'$ implies that there is a $w'v$-path in $N'$. Since *w* is the unique child of $w'$ and $v\ne w'$, this path must pass through *w* and thus $v\prec _{N'} w$ which together with $v,w\in V(N)$ implies $v\prec _{N} w$. Hence, (*u*, *v*) is a shortcut in *N*.
(ii) Suppose now that $v= w'$. Then, by construction, $(u, w)\in V(N)$. Moreover, we have $w\prec _{N'} w'\preceq _{N'} v'$. In particular, *u*, *w*, and $v'$ are all vertices in $N'$. Hence, $w\preceq _{N'} v'$ implies $w\preceq _{N} v'$ and since all newly inserted arcs are incident with $w'$, $(u,v')\in V(N')$ is also an arc in *N*. In summary, therefore, $(u, w)\in V(N)$ and $w\preceq _{N} v'$ for $v'\in {{\,\textrm{child}\,}}_N(u){\setminus }\{w\}$, i.e., (*u*, *w*) is a shortcut in *N*.

Suppose now that $(u,v) \in E(N)$ is a shortcut in *N*, i.e., there is a $v'\in {{\,\textrm{child}\,}}_{N}(u)\setminus \{v\}$ such that $v\prec _{N} v'$.

(i’) Suppose $v\ne w$. By construction, the arc (*u*, *v*) still exists in $N'$. Since moreover $u,v,v'\in V(N)\subset V(N')$, $v\prec _{N} v' \prec _{N} u$ and *N* and $N'$ are $(N',N)$-ancestor-preserving (cf. Lemma 5), we have $v\prec _{N'} v' \prec _{N'} u$. Hence, (*u*, *v*) must be a shortcut in $N'$.

(ii’) Finally, suppose $v= w$. Observe that $u'\preceq _{N} v'(\prec _{N} u)$ for some $u'\in {{\,\textrm{par}\,}}_{N}(v){\setminus }\{u\}$ and that $u, u',v'\in V(N)$. In particular, it holds $u'\prec _{N'} u$ since *N* and $N'$ are $(N',N)$-ancestor-preserving (cf. Lemma 5). By construction, we have $(u,w'),(u',w')\in V(N')$ and thus $w'\prec _{N'} u'$. In summary, we have $w'\prec _{N'} u' \prec _{N'} u$, which implies that $(u,w')$ is a shortcut in $N'$. $\square$

#### Lemma 73

Let *N* be a network, $(w',w) \in E(N)$ be an arc that is not a shortcut, and $N'$ be the network obtained from *N* by applying ${{\,\mathrm{\textsc {cntr}}\,}}(w',w)$. If two distinct vertices $u,v\in V(N')$ are in a common non-trivial block of $N'$, then either (i) $w\notin \{u,v\}$ and $u,v\in V(N)$ are in a common non-trivial block of *N* or (ii) $w\in \{u,v\}$ and the unique element in $\{u,v\}\setminus \{w\}$ and $w'$ are in a common block in *N*.

#### Proof

Suppose two distinct vertices $u,v\in V(N')=V(N)\setminus \{w'\}$ are in a common non-trivial block of $N'$. Thus, *u* and *v* are contained in an undirected cycle $K'$ in $N'$. If (the directed versions of) all arcs in $K'$ are arcs of *N*, then $K'$ is an undirected cycle in *N*, and thus, *u* and *v* are contained in a non-trivial block of *N*. Now, suppose that at least one arc *e* in $K'$ is not contained in *N*. By construction, *e* must be of the form (*p*, *w*) with $p\in {{\,\textrm{par}\,}}_N(w'){\setminus }{{\,\textrm{par}\,}}_N(w)$ or of the form (*w*, *c*) with $c\in {{\,\textrm{child}\,}}_N(w'){\setminus }{{\,\textrm{child}\,}}_N(w)$. Since such an arc is incident with *w*, the only arcs of $N'$ that possibly are not contained in *N* are the two arcs incident with *w* in $K'$. Hence, we have to consider the following cases:

1. both of the incident arcs of *w* in $K'$ are not contained in *N* but all other arcs are contained in *N*,
  - *w* is incident in $K'$ with two distinct parents $p_1, p_2\in {{\,\textrm{par}\,}}_N(w'){\setminus }{{\,\textrm{par}\,}}_N(w)$,
  - *w* is incident in $K'$ with two distinct children $c_1, c_2\in {{\,\textrm{child}\,}}_N(w'){\setminus }{{\,\textrm{child}\,}}_N(w)$, or
  - *w* is incident in $K'$ with a parent $p\in {{\,\textrm{par}\,}}_N(w'){\setminus }{{\,\textrm{par}\,}}_N(w)$ and a child $c\in {{\,\textrm{child}\,}}_N(w'){\setminus }{{\,\textrm{child}\,}}_N(w)$.
2. exactly one of the arcs incident with *w* in $K'$ is not contained in *N*, while all other arcs of $N'$ are contained in *N*.
  - *w* is incident in $K'$ with a parent $p\in {{\,\textrm{par}\,}}_N(w'){\setminus }{{\,\textrm{par}\,}}_N(w)$, or
  - *w* is incident in $K'$ with a child $c\in {{\,\textrm{child}\,}}_N(w'){\setminus }{{\,\textrm{child}\,}}_N(w)$.

Observe that $w'\notin V(N')$ and thus $w'$ is not contained in $K'$. One therefore easily verifies that, in each of cases **(1a)**, **(1b)**, and **(1c)**, replacing *w* by $w'$ in $K'$ yields an undirected cycle *K* in *N*. If $w\notin \{u,v\}$, then *u* and *v* are contained in *K* and thus contained in a common non-trivial block of *N*. If on the other hand $w\in \{u,v\}$, then the unique element in $\{u,v\}\setminus \{w\}$ and $w'$ are contained in *K* and thus contained in a common non-trivial block of *N*. In case **(2a)**, $K'$ contains the arc (*p*, *w*) by construction of $N'$. Replacing this arc by vertex $w'$ and the arcs $(p,w'),(w',w)\in V(N)$ therefore yields an undirected cycle *K* in *N* that contains all vertices in $K'$. In particular, therefore, $u,v\in V(K')$ are contained in a common non-trivial block in *N*. The latter is also true in case **(2b)** by similar arguments. $\square$

From Lemma 8, we obtain

#### Corollary 45

Let *N* be a network and $u,v,w\in V(N)$ three distinct vertices. If *u* and *v* are contained in block $B_{uv}$, *u* and *w* are contained in block $B_{uw}$, and *v* and *w* are contained in block $B_{vw}$, then $B_{uv}=B_{uw}=B_{vw}$.

#### Proof

If $u\notin \{\max B_{uv}, \max B_{uw}\}$, then Lemma 9 immediately implies that $B_{uv}= B_{uw}$. Thus, suppose (a) $u= \max B_{uv}$ or (b) $u= \max B_{uw}$. In Case (a), we have $v\prec _N u$ and $v\ne \max B_{uv}$. If $v\ne \max B_{vw}$, then $B_{uv}= B_{vw}$ by Lemma 9. Now, assume $v=\max B_{vw}$ which implies $w\prec _N v$ and $w\ne \max B_{vw}$. If $w\ne \max B_{uw}$, then $B_{uw}= B_{vw}$ by Lemma 9. The case $w=\max B_{uw}$ is not possible since otherwise $u\prec _N w$, and thus, $u\prec _N w\prec _N v\prec _N u$, a contradiction. One argues similarly in Case (b). Hence, in all possible case, *u*, *v*, and *w* are contained in a common non-trivial block *B*. Since each of $B_{uv}$, $B_{uw}$, and $B_{vw}$ shares two vertices with *B*, it holds $B=B_{uv}=B_{uw}=B_{vw}$. $\square$

#### Lemma 74

Let *N* be a network, $(w',w) \in E(N)$ be an arc that is not a shortcut, and $N'$ be the network obtained from *N* by applying ${{\,\mathrm{\textsc {cntr}}\,}}(w',w)$. Let $B'$ be a non-trivial block of $N'$. Then, there is a non-trivial block *B* of *N* with $V(B'){\setminus } \{w\} \subseteq V(B)$. Moreover, $w\in V(B')$ and $w\notin V(B)$ imply $w'\in V(B)$.

If $v\in V(B')\setminus \{w\}$ is a hybrid vertex and properly contained in $B'$, then *v* is a properly contained hybrid vertex in *B*. If *w* is a properly contained hybrid vertex in $B'$, then at least one of *w* and $w'$ is a properly contained hybrid vertex in *B*.

#### Proof

Suppose $B'$ is a non-trivial block of $N',$ and thus, it contains at least three vertices. In particular, we can find two distinct vertices in *u*, *v* such that $w\notin \{u,v\}$. By Lemma 73, $u,v\in V(N)$ are in a common non-trivial block $B{:}{=}B_{uv}$ of *N*. Now, suppose there is $u'\in V(B')\setminus \{w,u,v\}$. By Lemma 73, $u,u'\in V(N)$ and $v,u'\in V(N)$, resp., are in common non-trivial blocks $B_{uu'}$ and $B_{vu'}$ of *N*. By Corollary 45, *u*, *v*, and $u'$ are contained in $B=B_{uv}=B_{uu'}=B_{vu'}$ of *N*. Since $u'\in V(B')\setminus \{w,u,v\}$ was chosen arbitrarily and blocks that share two vertices are equal by Observation 1, we have $V(B')\setminus \{w\}\subseteq V(B)$.

Now, suppose $w\in V(B')$ and $w\notin V(B)$. By Lemma 75(ii), $u,w'\in V(N)$ and $v,w'\in V(N)$, resp., are in common non-trivial blocks $B_{uw'}$ and $B_{vw'}$ of *N*. By Corollary 45, $w'$ is contained in $B_{uw'}=B_{vw'}=B_{uv}=B$.

Suppose $v\in V(B')\setminus \{w\}$ is a hybrid vertex and properly contained in $B'$. By Lemma 11, all of the at least two vertices in ${{\,\textrm{par}\,}}_{N'}(v)$ are contained in $B'$. Hence, let $p, p'\in {{\,\textrm{par}\,}}_{N'}(v)$ be two distinct parents, and assume w.l.o.g. that $p\ne w$. Note that $v,p \in V(B'){\setminus } \{w\}\subseteq V(B)$. Moreover, since all newly inserted arcs to obtain $N'$ from *N* are incident with *w*, the arc $(p,v)\in E(N')$ is also an arc in *N*. Now, consider $p'$. If $(p',v)\in E(N)$, then *v* is a hybrid vertex in *N*. If $(p',v)\notin E(N)$, then, by construction of $N'$, we must have $p'=w$ and $v\in {{\,\textrm{child}\,}}_{N}(w'){\setminus } {{\,\textrm{child}\,}}_{N}(w)$. Hence, we have $(w',v)\in E(N)$. Together with $w'\ne p$ (since $p\in V(N')=V(N){\setminus }\{w'\}$) this implies that *v* is a hybrid vertex in *N* also in this case. Therefore, and since $p\in {{\,\textrm{par}\,}}_N(v)$ and $v,p\in V(B)$, Lemma 11 implies that *v* is properly contained in *B*.

Suppose *w* is a properly contained hybrid vertex in $B'$. By Lemma 11, all of the at least two vertices in ${{\,\textrm{par}\,}}_{N'}(w)$ are contained in $B'$. We distinguish cases (a) $w\in V(B)$ and (b) $w\notin V(B)$.

*Case (a):* $w\in V(B)$. Suppose first that there is $p\in {{\,\textrm{par}\,}}_{N'}(w) \cap {{\,\textrm{par}\,}}_{N}(w)$. We have $p\in V(B'){\setminus }\{w\} \subseteq V(B)$. Moreover, *w* has at least the two distinct parents $w'$ and *p* in *N*, i.e., *w* is a hybrid vertex in *N*. By Lemma 10 and since *w* and its parent *p* are both contained in *B*, *w* is properly contained in *B*. Suppose now that ${{\,\textrm{par}\,}}_{N'}(w) \cap {{\,\textrm{par}\,}}_{N}(w)=\emptyset$. By construction of $N'$, this implies that all of the at least two vertices in ${{\,\textrm{par}\,}}_{N'}(w)$ must be vertices in ${{\,\textrm{par}\,}}_{N}(w')$. Hence, $w'$ is a hybrid vertex in *N* with at least two parents *p* and $p'$ that are parents of *w* in $N'$ and thus contained in $V(B')\setminus \{w\} \subseteq V(B)$. By Lemma 10, $\{w',p,p'\}\in V(\tilde{B})$ for some block $\tilde{B}$ of *N*. Since *B* and $\tilde{B}$ are blocks in *N* that share the two vertices *p* and $p'$, we conclude $B=\tilde{B}$. In particular, $w'$ is a properly contained hybrid vertex in *B*.

*Case (b):* $w\notin V(B)$. We have already seen that this implies that $w'\in V(B)$. Since *w* is properly contained in $B'$, all of its at least two parents in $N'$ are also contained in $B'$. Let $p\in {{\,\textrm{par}\,}}_{N'}(w)$. Suppose, for contradiction, that $p\in {{\,\textrm{par}\,}}_{N}(w)$. Then, *w* has at least the two distinct parents $w'$ and *p* in *N*, i.e., *w* is a hybrid vertex in *N*. By Lemma 10, $\{w,w',p\}\in V(\tilde{B})$ for some block $\tilde{B}$ of *N*. Since *B* and $\tilde{B}$ are blocks in *N* that share the two vertices $w'$ and *p*, we conclude $B=\tilde{B}$ and thus $w\in V(B)$, a contradiction. Hence, $p\notin {{\,\textrm{par}\,}}_{N}(w)$. Together with $p\in {{\,\textrm{par}\,}}_{N'}(w)$, this implies $p\in {{\,\textrm{par}\,}}_{N}(w')$. Since the latter is true for all of the at least two vertices $p'\in {{\,\textrm{par}\,}}_{N'}(w)$, $w'$ must be a hybrid vertex in *N*. Therefore, and because $p\in {{\,\textrm{par}\,}}_N(w')$ and $w',p\in V(B)$, Lemma 11 implies that $w'$ is properly contained in *B*. $\square$

We are now in the position to prove Lemma 12. To recall, Lemma 12 states that ${{\,\mathrm{\textsc {cntr}}\,}}(w',w)$ of a non-shortcut arc $(w',w)$ in a level-*k* network *N* preserves the property of the resulting network $N'$ to be level-*k*.

#### Proof of Lemma 12

Suppose, for contraposition, that $N'$ is not level-*k*. Hence, there is a block $B'$ in $N'$ that properly contains at least $k+1$ hybrid vertices. Denote the set of these vertices by *A*. By Lemma 74, it holds $V(B'){\setminus } \{w\} \subseteq V(B)$ for some non-trivial block *B* of *N*, and in particular, all vertices in $A\setminus \{w\}$ are properly contained hybrid vertices in *B*. If $w\notin A$, then *B* properly contains at least $k+1$ hybrid vertices. Otherwise, *w* is a properly contained hybrid vertex in $B'$ and *B* properly contains at least *k* hybrid vertices in $A\setminus \{w\}$, and, by Lemma 74, at least on of $w,w'\notin A{\setminus } \{w\}$ is an additional properly contained hybrid vertex in *B*. Therefore, the block *B* in *N* properly contains at least $k+1$ hybrid vertices in both cases, and thus, *N* is not level-*k*. $\square$

#### Lemma 75

Let *N* be a network, $N'$ be the network obtained from *N* by applying ${{\,\mathrm{\textsc {expd}}\,}}(w)$ for some $w\in V(N)$, and $w'$ be the unique vertex in $V(N')\setminus V(N)$. If two distinct vertices $u,v\in V(N')$ are in a common non-trivial block of $N'$, then either (i) $w'\notin \{u,v\}$ and $u,v\in V(N)$ are in a common non-trivial block of *N* or (ii) $w'\in \{u,v\}$ and *w* and the element in $\{u,v\}\setminus \{w'\}$ are in a common block in *N*.

#### Proof

Suppose two distinct vertices $u,v\in V(N')$ are in a common non-trivial block $B'$ of $N'$. Then, *u* and *v* lie in an undirected cycle $K'$ in $N'$.

(i) Assume first that $w'\notin \{u,v\}$, and thus, $u,v\in V(N)$. If $w'$ is not contained in $K'$, then all arcs in $K'$ are also arcs in *N* since all newly inserted arcs are incident with $w'$. Hence, *u* and *v* lie on an undirected cycle in *N* and are thus contained in a common non-trivial block *B* in this case. Now, suppose that $K'$ contains $w'$. Assume that $w'$ is incident with its unique child *w* in $K'$. Then, by construction of $N'$, the second vertex that is incident with $w'$ in $K'$ must be a vertex $p\in {{\,\textrm{par}\,}}_{N'}(w')={{\,\textrm{par}\,}}_N(w)$. In particular, we have $(p,w)\in E(N)$ and $(p,w),(w,p)\notin E(N')$. The latter implies that *p* and *w* are not incident in $K'$ and therefore $K'$ contains at least one additional vertex $x\notin \{p,w',w\}$. Together with the fact that *N* contains all arcs in $K'$ except $(p,w')$ and $(w',w)$, the latter arguments imply that there is an undirected cycle *K* in *N* formed by the vertices in $V(K')\setminus \{w'\}$ and the arcs in $(E(K'){\setminus }\{(p,w'),(w',w)\})\cup \{(p,w)\}$. Hence, $u,v\in V(K'){\setminus } \{w'\}$ lie in a common non-trivial block of *N*. Now, suppose $w'$ is not incident with its unique child *w* in $K'$, and thus, the two incident vertices in $K'$ must be two distinct element $p_1,p_2\in {{\,\textrm{par}\,}}_{N'}(w')={{\,\textrm{par}\,}}_N(w)$. Thus, we have $(p_1,w),(p_2,w)\in E(N)$. Since $(p_1,w')$ and $(p_2,w')$ are the only arcs incident with $w'$ in $K'$, all other arcs in $K'$ are also arcs in *N*. Thus, consider the (not necessarily induced) subgraph *K* of *N* formed by the vertices $(V(K'){\setminus } \{w'\})\cup \{w\}$ and arcs $(E(K')\setminus \{(p_1,w'),(p_2,w')\})\cup \{(p_1,w),(p_2,w)\}$. If $w\notin V(K')$, then one easily verifies that *K* is an undirected cycle in *N* that contains *u* and *v* and thus they are contained in a common block *B* of *N*. On the other hand, if $w\in V(K')$, then its two incident vertices in $K'$ are two distinct elements $c_1,c_2\in {{\,\textrm{child}\,}}_{N'}(w)={{\,\textrm{child}\,}}_{N}(w)$ since, by assumption, *w* is not incident in $K'$ with its unique parent $w'$. In this case, one easily verifies that *K* consists of two undirected cycles that share only the vertex *w* and each of the two cycles contains exactly one of $p_1,p_2\in {{\,\textrm{par}\,}}_N(w)$ and one of $c_1,c_2\in {{\,\textrm{child}\,}}_{N}(w)$. If *u* and *v* are contained in the same of these two cycles, then they are contained in a common non-trivial block of *N*. Otherwise, they are contained in non-trivial blocks *B* and $B'$, resp., that each contain *w* and one of its parents. The latter implies that $w\notin \{\max B, \max B'\}$. Hence, we have $B=B'$ by Lemma 9.
(ii) Suppose now that $w'\in \{u,v\}$, say $v=w'$. We can essentially reuse the arguments from case (i), with exception of the case that $w'$ is not contained in $K'$ (which is impossible since $w'=v\in V(K')$), since we always have constructed an undirected cycle in *N* that contains $u,w\in V(N)$. Hence, they are contained in a non-trivial block of *N*.

$$\square$$

#### Lemma 76

Let *N* be a network, $N'$ be the network obtained from *N* by applying ${{\,\mathrm{\textsc {expd}}\,}}(w)$ for some $w\in V(N)$, and $w'$ be the unique vertex in $V(N')\setminus V(N)$. Let $B'$ be a non-trivial block of $N'$. Then, there is a non-trivial block *B* of *N* with $V(B')\setminus \{w'\} \subseteq V(B)$. Moreover, $w'\in V(B')$ implies $w\in V(B)$.

If $v\in V(B')\setminus \{w'\}$ is a hybrid vertex and properly contained in $B'$, then *v* is a properly contained hybrid vertex in *B*. If $w'\in V(B')$ and $w'$ is a hybrid vertex, then *w* is a properly contained hybrid vertex in *B*.

#### Proof

Suppose $B'$ is a non-trivial block of $N',$ and thus, it contains at least three vertices. In particular, we can find two distinct vertices in *u*, *v* such that $w'\notin \{u,v\}$. By Lemma 75, $u,v\in V(N)$ are in a common non-trivial block $B{:}{=}B_{uv}$ of *N*. Now, suppose there is $u'\in V(B')\setminus \{w',u,v\}$. By Lemma 75, $u,u'\in V(N)$ and $v,u'\in V(N)$, resp., are in common non-trivial blocks $B_{uu'}$ and $B_{vu'}$ of *N*. By Corollary 45, *u*, *v*, and $u'$ are contained in $B= B_{uv}$. Since $u'\in V(B'){\setminus } \{w',u,v\}$ was chosen arbitrarily and blocks that share two vertices are equal by Observation 1, we have $V(B')\setminus \{w'\}\subseteq V(B)$.

Now, suppose $w'\in V(B')$. If $w\in V(B')$, then we have already seen that $w\in V(B)$. Hence, suppose $w\notin V(B')$. By Lemma 75, $u,w\in V(N)$ and $v,w\in V(N)$, resp., are in common non-trivial blocks $B_{uw}$ and $B_{vw}$ of *N*. By Corollary 45, *w* must also be contained in *B*.

Suppose $v\in V(B')\setminus \{w'\}$ is a hybrid vertex and properly contained in $B'$. Since *w* has a unique parent in $N'$, we have $v\ne w$ and thus ${{\,\textrm{par}\,}}_{N'}(v)={{\,\textrm{par}\,}}_N(v)$. Moreover, since *v* is properly contained in $B'$, we have ${{\,\textrm{par}\,}}_{N'}(v)\in V(B')$ and thus ${{\,\textrm{par}\,}}_{N'}(v)\in V(B)$. Taken together, the latter arguments imply that $v\ne \max B$, i.e., *v* is properly contained in *B*.

Suppose $w'\in V(B')$ and $w'$ is a hybrid vertex. Since $w'$ has a unique child and must lie on an undirected cycle in $N'$, $w'$ must be properly contained in $B'$. Therefore, all of its at least two parent are also contained in $B'$ and thus in *B*. Together with the fact that $w\in V(B)$ and ${{\,\textrm{par}\,}}_{N'}(w')={{\,\textrm{par}\,}}_N(w)$, we obtain that *w* is a hybrid vertex and properly contained in *B*. $\square$

We now prove Lemma 13. To recall, Lemma 13 states that two networks *N* and $N'$ are always level-*k* whenever $N'$ is obtained from *N* by applying ${{\,\mathrm{\textsc {expd}}\,}}(w)$ for some $w\in V(N)$ and at least one of them is level-*k*.

#### Proof of Lemma 13

Denote by $w'$ the unique vertex in $V(N')\setminus V(N)$. Suppose that $N'$ is not level-*k*. Hence, there is a block $B'$ in $N'$ that properly contains at least $k+1$ hybrid vertices. Denote the set of these vertices by *A*. Observe that $w\notin A$ since it has a single parent *w* in $N'$. By Lemma 76, it holds $V(B'){\setminus } \{w'\} \subseteq V(B)$ for some non-trivial block *B* of *N*, and in particular, all vertices $A\setminus \{w'\}$ are properly contained hybrid vertices in *B*. If $w'\notin A$, then *B* properly contains at least $k+1$ hybrid vertices. Otherwise, *B* properly contains at least *k* hybrid vertices in $A\setminus \{w'\}$ and, by Lemma 76, additionally the hybrid vertex $w\notin A$. Therefore, the block *B* in *N* properly contains at least $k+1$ hybrid vertices in both cases, and thus, *N* is not level-*k*.

Conversely, suppose that $N'$ is level-*k*. Observe that *N* is recovered from $N'$ by applying ${{\,\mathrm{\textsc {cntr}}\,}}(w',w)$. By Lemma 12, therefore, *N* is also level-*k*. $\square$

### Appendix 1.2: Closed clustering systems

As promised, we provide here a short proof of Lemma 16, which states that a clustering system $\mathscr {C}$ is closed if and only if $A,B \in \mathscr {C}$ and $A\cap B\ne \emptyset$ implies $A\cap B\in \mathscr {C}$.

#### Proof of Lemma 16

Let $\mathscr {C}$ be closed and let $A,B\in \mathscr {C}$. Since ${{\,\textrm{cl}\,}}$ is enlarging, we have $A\cap B\subseteq {{\,\textrm{cl}\,}}(A\cap B)$. Moreover, since $A\cap B\subseteq A,B$, it holds by Eq. (2) that ${{\,\textrm{cl}\,}}(A\cap B)\subseteq A\cap B$. Hence, ${{\,\textrm{cl}\,}}(A\cap B)=A\cap B$ and the definition of “closed” implies $A\cap B \in \mathscr {C}$. Assume now that $C\cap C'\in \mathscr {C}$ for all $C,C'\in \mathscr {C}$. Equation (2) implies ${{\,\textrm{cl}\,}}(A)=A$ for all $A\in \mathscr {C}$. It remains to show that, for $A\in 2^X$, ${{\,\textrm{cl}\,}}(A)=A$ implies $A\in \mathscr {C}$. By Eq. (2), ${{\,\textrm{cl}\,}}(A)$ can be written as the intersection ${{\,\textrm{cl}\,}}(A)=\bigcap _{i=1}^k C_i$ of a finite number *k* of clusters $C_i\in \mathscr {C}$ with $A\subseteq C_i$. Since $A\subseteq X\in \mathscr {C}$ we have $k\ge 1$. If $k\in \{1,2\}$, ${{\,\textrm{cl}\,}}(A) = A \in \mathscr {C}$ follows immediately from the assumption that $C\cap C'\in \mathscr {C}$ for all $C,C'\in \mathscr {C}$. Otherwise, we can construct a series of intersections $C'_i$, $1\le i\le k$, by setting $C'_1{:}{=}C_1$ and $C'_j{:}{=}C'_{j-1}\cap C_{j}$ for $2\le j\le k$. By definition, $C'_1=C_1\in \mathscr {C}$. Moreover, if $C'_{j-1} \in \mathscr {C}$, then $C'_{j} \in \mathscr {C}$ holds by the assumption and the fact that $C_j\in \mathscr {C}$ for all $2\le j\le k$. By induction, therefore, we obtain $C'_k\in \mathscr {C}$. We have $C'_k={{\,\textrm{cl}\,}}(A)$ by construction and thus ${{\,\textrm{cl}\,}}(A) = A \in \mathscr {C}$. $\square$

### Appendix 1.3: Algorithmic details

We show here the correctness and runtime results for Check-L1-Compatibility.

#### Proof of Theorem 9

Let $\mathscr {C}\subseteq 2^X$ be a clustering system. Suppose first that Check-L1-Compatibility returns a network. In particular, $\mathscr {C}$ satisfies (L) in this case. By Observation 15 and Lemma 65, $\mathcal {I}(\mathscr {C})$ is correctly computed and satisfies (L). By definition, $\mathcal {I}(\mathscr {C})$ is closed. The latter two arguments together with Proposition 21 imply that there is a (separated, phylogenetic) level-1 network on *X* such that $\mathscr {C}\subseteq \mathcal {I}(\mathscr {C})= \mathscr {C}_N$.

Conversely, suppose that Check-L1-Compatibility returns “no solution”. Assume for contradiction that there is a level-1 network *N* with $\mathscr {C}\subseteq \mathscr {C}_N$. By Theorem 8, $\mathscr {C}_N$ is closed and satisfies (L). By Corollary 29, Property (L) implies that $\mathscr {C}$ is weak hierarchy. As shown in Bandelt and Dress (1989) (right below Lemma 1), this in turn implies that $$\vert \mathscr {C}\vert \le \vert \mathscr {C}_N\vert \le \left( {\begin{array}{c}\vert X\vert +1\\ 2\end{array}}\right) = \left( {\begin{array}{c}\vert X\vert \\ 2\end{array}}\right) +\vert X\vert$$. Inputs larger than this bound are therefore correctly rejected immediately. If, on the other hand, Property (L) is not satisfied for $\mathscr {C}$, then by the definition of Property (L), its superset $\mathscr {C}_N$ also violates (L), a contradiction. Therefore, such a network *N* cannot exist and Check-L1-Compatibility correctly exits with a negative answer.

We now proceed to show that the algorithm can be implemented to run in $O(\vert \mathscr {C}\vert ^2\vert X \vert )\subseteq O(\vert X\vert ^5)$ time. To this end, we first enumerate the elements in *X* from 1 to $\vert X\vert$. We then initialize a list $\mathcal {L}$ containing a bitvector $b_i$ of size $\vert X\vert$ for each $C_i\in \mathscr {C}$ that has a 1-entry at position *j* if and only if the *j*th element of *X* is contained in $C_j$, and a 0-entry otherwise requiring a total effort of $O(\vert \mathscr {C}\vert \vert X\vert )$ time. Moreover, we create an initially arcless auxiliary graph *G* whose vertices are (unique identifiers of) the clusters in $\mathscr {C}$. In the end, two clusters $C_i,C_j\in \mathscr {C}$ will be connected by an arc in *G* precisely if they overlap. Moreover, every arc $\{C_i,C_j\}$ will be associated with a pointer to a bitvector that represents the intersection $C_i\cap C_j$.

To achieve this, we proceed as follows for every pair $C_i, C_j\in \mathscr {C}$. We compute the bitvector *b* corresponding to the intersection $C_i\cap C_j$ as $b\leftarrow b_i \wedge b_j$. The clusters $C_i$ and $C_j$ overlap if and only if the number of 1-entries in *b* is greater than 0 but less than $\vert C_i\vert$ and $\vert C_j\vert$ (the latter cardinalities can be pre-computed for all clusters in $\mathscr {C}$). If the clusters do overlap, then we continue as follows. If $C_i$ is already adjacent with some other cluster in $\mathscr {C}\setminus \{C_i, C_j\}$, then we pick $C_k$ among them arbitrarily. Let $b'$ be the bitvector associated with the arc $\{C_i,C_k\}$ in *G*. By (L), *b* and $b'$ must be equal, which can be checked in $O(\vert X\vert )$. If they are not equal, we can exit “no solution.” If existent, we proceed analogously with some neighbor $C_l\in \mathscr {C}{\setminus }\{C_i, C_j\}$ of $C_j$ in *G* with associated bitvector $b''$. We add the arc $\{C_i,C_j\}$ to *G*. If at least one of $C_i$ and $C_j$ previously had a neighbor $C_k$ or $C_j$, respectively, then we associate the bitvector $b'$ or $b''$, respectively, with the new arc $\{C_i,C_j\}$ and discard *b*. Otherwise, we associate *b* with $\{C_i,C_j\}$ and add *b* to $\mathcal {L}$. In summary, for a pair $C_i,C_j\in \mathscr {C}$, we only perform a constant number of operations, all of which require at most $O(\vert X\vert )$ time. Hence, we obtain a total effort of $O(\vert \mathscr {C}\vert ^2 \vert X\vert )$ time for processing all pairs of clusters in $\mathscr {C}$.

One easily verifies that $\mathscr {C}$ satisfies (L) if the algorithms did not exit at this point because all overlaps are represented by arcs in *G* and, for each $C\in \mathscr {C}$, the intersections with its overlapping clusters are stepwisely added and compared to the overlaps that where already computed. Moreover, we have added at most $\lfloor \vert \mathscr {C}\vert / 2 \rfloor$ bitvectors to $\mathcal {L}$. To see this, recall that we only added a new bitvector *b* (associated with arc $\{C_i,C_j\}$) if neither of $C_i$ and $C_j$ had any other neighbors at that point. Since each arc is incident with two clusters, we can clearly do this at most $\lfloor \vert \mathscr {C}\vert / 2 \rfloor$ times until all but possible one clusters are adjacent with some other cluster. Hence, $\mathcal {L}$ still contains only $O(\vert \mathscr {C}\vert )$ bitvectors. In particular, $\mathcal {L}$ contains all clusters in $\mathcal {I}(\mathscr {C})=\mathscr {C} \cup \{C\cap C' \mid C,C'\in \mathscr {C}\text { overlap}\}$ (represented by their bitvectors) at least once.

We now sort $\mathcal {L}$ lexicographically in $O( \vert X\vert \vert \mathscr {C}\vert \log \vert \mathscr {C}\vert ) = O( \vert X\vert \, \vert \mathscr {C}\vert \log \vert X\vert )$, where the additional factor $\vert X\vert$ originates from the fact that each comparison of bitvectors requires $O(\vert X\vert )$ comparisons of their entries. Removal of all duplicates in $\mathcal {L}$ now takes $O(\vert \mathscr {C}\vert \vert X \vert )$ time, e.g., by iterating through the list and comparing each vector to the last added bitvector in a newly constructed list. The bitvectors in this final list $\mathcal {L}$ are thus in a 1-to-1 correspondence with the clusters in $\mathcal {I}(\mathscr {C})$.

If we are only interested in the existence of a (separated, phylogenetic) level-1 network *N* such that $\mathscr {C}\subseteq \mathscr {C}_{N}$ and the clustering system $\mathscr {C}_{N}$, then we can stop here after a total effort of $O(\vert \mathscr {C}\vert ^2 \vert X\vert )\subseteq O(\vert X\vert ^5)$ time.

Otherwise we continue with the construction of the inclusion order, i.e., the Hasse diagram of $\mathcal {I}(\mathscr {C})$. In the following, the cluster $C_i\in \mathcal {I}(\mathscr {C})$ corresponds to the *i*th bitvector in $\mathcal {L}$. We initialize a $\vert \mathcal {I}(\mathscr {C})\vert \times \vert \mathcal {I}(\mathscr {C})\vert$-matrix *M* with all zero entries (requiring $O(\vert \mathscr {C}\vert ^2)$ time and space). In the end, we will have $M_{i,j}=1$ if and only if $C_i\subsetneq C_j$. Since $\mathcal {L}$ is still sorted lexicographically, observe that if $C_i\subsetneq C_j$, then $i<j$. Hence, it suffices to check for all $1\le i < j \le \vert \mathcal {I}(\mathscr {C})\vert$ whether all 1-entries in $b_i$ are also 1-entries in $b_j$ and, if so, set $M_{i,j}=1$. Finally, the adjacency matrix of ${\mathfrak {H}}[\mathcal {I}(\mathscr {C})]$ is obtained from *M* by Transitive Reduction. As shown in Aho et al. (1972) for DAGs, this task has the same complexity as Transitive Closure, which, in our setting, is bounded $O(\vert X\vert ^{2\omega })$. Here, $\omega \le 2.3729$ is the “matrix multiplication constant.” Thus, ${\mathfrak {H}}[\mathcal {I}(\mathscr {C})]$ can also be constructed in quintic time. Thus, we obtain a regular (and thus phylogenetic) level-1 network $N\sim {\mathfrak {H}}[\mathcal {I}(\mathscr {C})]$ in $O(\vert X\vert ^5)$ time. This network can be modified to be separated by arc expansion at all hybrid vertices *v* with ${{\,\textrm{outdeg}\,}}(v)$. Since ${\mathfrak {H}}[\mathcal {I}(\mathscr {C})]$ has $O(\vert X\vert ^2)$ vertices, for each vertex, one checks in constant time whether ${{\,\textrm{indeg}\,}}(v)>1$ and ${{\,\textrm{outdeg}\,}}(v)>1$, and ${{\,\mathrm{\textsc {expd}}\,}}(v)$ requires constant effort for moving the list of out-neighbors from *v* to the newly inserted vertex, the total effort for the modification of the Hasse diagram is bounded by $O(\vert X\vert ^2)$. $\square$
